# The role of intestinal immune cells and matrix metalloproteinases in inflammatory bowel disease

**DOI:** 10.3389/fimmu.2022.1067950

**Published:** 2023-01-17

**Authors:** Kun Mei, Zilu Chen, Qin Wang, Yi Luo, Yan Huang, Bin Wang, Renjun Gu

**Affiliations:** ^1^ Nanjing University of Chinese Medicine, Nanjing, China; ^2^ Department of Cardiothoracic Surgery, The Third Affiliated Hospital of Soochow University, Changzhou, China; ^3^ Department of Oncology, Jiangsu Province Hospital on Integration of Chinese and Western Medicine, Nanjing, Jiangsu, China; ^4^ Department of Oncology, Affiliated Hospital of Integrated Traditional Chinese and Western Medicine, Nanjing University of Chinese Medicine, Nanjing, Jiangsu, China; ^5^ Department of Ultrasound, Nanjing Hospital of Chinese Medicine Affiliated to Nanjing University of Chinese Medicine, Nanjing, China; ^6^ School of Chinese Medicine & School of Integrated Chinese and Western Medicine, Nanjing University of Chinese Medicine, Nanjing, China; ^7^ Department of Gastroenterology and Hepatology, Jinling Hospital, Medical School of Nanjing University, Nanjing, Jiangsu, China

**Keywords:** inflammatory bowel disease, matrix metalloproteinases, immune microenvironment, biomarker, gene expression omnibus

## Abstract

Inflammatory bowel disease (IBD) has become globally intractable. MMPs play a key role in many inflammatory diseases. However, little is known about the role of MMPs in IBD. In this study, IBD expression profiles were screened from public Gene Expression Omnibus datasets. Functional enrichment analysis revealed that IBD-related specific functions were associated with immune pathways. Five MMPS-related disease markers, namely MMP-9, CD160, PTGDS, SLC26A8, and TLR5, were selected by machine learning and the correlation between each marker and immune cells was evaluated. We then induced colitis in C57 mice using sodium dextran sulfate and validated model construction through HE staining of the mouse colon. WB and immunofluorescence experiments confirmed that the expression levels of MMP-9, PTGDS, SLC26A8, and CD160 in colitis were significantly increased, whereas that of TLR5 were decreased. Flow cytometry analysis revealed that MMPs regulate intestinal inflammation and immunity mainly through CD8 in colitis. Our findings reveal that MMPs play a crucial role in the pathogenesis of IBD and are related to the infiltration of immune cells, suggesting that MMPs may promote the development of IBD by activating immune infiltration and the immune response. This study provides insights for further studies on the occurrence and development of IBD.

## 1 Introduction

The global incidence of inflammatory bowel disease (IBD) is increasing yearly (1). IBD includes intestinal autoimmune diseases, inflammation, stimulation of inflammatory cells, with the release of inflammatory cytokines, activation of immune cells, and abnormal changes in intestinal microvascular endothelial cell function, which affect immune cell function and the stability of the intestinal environment and lead to cell and tissue damage (2). In recent years, it is generally believed that a part of colorectal cancer can progress from IBD (3, 4). The inflammatory response of the colon is a major factor in the development of colorectal cancer (5). Studies on the treatment of IBD provide insights for the prevention and treatment of colorectal cancer. However, due to the increasing incidence, long course, and delayed healing in IBD, its diagnosis, treatment, and prognosis have become a challenge (6).

Although IBD can be triggered by various factors, the immune response appears critical for the onset of IBD (7, 8). As the largest immune organ of mammals, the gut contains many types of immune cells, including B cells, T cells, dendritic cells, macrophages, eosinophils, and mast cells (7, 9). When the intestinal barrier is damaged, bacterial infection occurs, which affects the process of IBD (10). A focus of our research includes identifying the immune cells that play a role in the pathogenesis of IBD. At present, therapy for IBD mostly involves inhibiting intestinal inflammation (11). Immunotherapy that can regulate the intestinal barrier also provides a new idea for treating IBD.

MMPs are enzymes with specific biological activities (12). MMPs participate in many activities related to maintaining their own stability and play a wide range of roles in the development of disease (13). MMPs can regulate inflammation at all levels. They can regulate the migration of inflammatory cells from the artery to the inflammatory zone and process ECM components, growth factors, cytokines, and chemokines, thus regulating the uptake of inflammatory cells and access to the inflammatory zone (14–16). The inflammatory marker role of metalloproteinases can help in the diagnosis and treatment of some inflammatory diseases (17), especially rheumatoid arthritis (18, 19); however, the role of MMPs in IBD has not been elucidated, and the value of MMPs in the immunotherapy of IBD is rarely demonstrated. Notably, the relationship between MMPs and immune cells may be much more complex than understood (20). Studies have demonstrated high expression of MMP-3 (21), MMP-9 (22, 23), and MMP-13 (24) in damaged colonic mucosa. This gives us confidence to further demonstrate the relationship between other MMPs and IBD.

To evaluate the potential impact of MMPs on IBD, machine learning was used to identify five MMP-related disease markers. The expression of MMP-9, PTGDS, SLC26A8, and CD160 in colitis was increased in the IBD mouse model, whereas the expression of TLR5 was downregulated. In addition, the findings revealed that MMPs regulated the occurrence and development of IBD through CD8.

## 2 Materials and methods

### 2.1 Data sources and processing

The GSE94648 and GSE119600 microarray data sets were downloaded from the Gene Expression Omnibus (GEO) datasets. The GSE94648 profile includes samples from 75 patients with IBD and 22 healthy controls, whereas GSE119600 contains samples from 188 patients with IBD and 47 healthy controls. The platforms are GPL19109 and GPL10558, respectively. Finally, the batch effect was eliminated using the “SVA” package in R, and the two datasets were subsequently merged. Metalloproteinase-related genes were retrieved from Gene Cards (https://www.genecards.org/). The cut-offs were set as Relevance Score > 0.2 (**Supplementary Table 1**).

### 2.2 Analysis of differentially expressed genes

The “limma” package in R is used to identify various genes. Genes with *P*-value < 0.05 and absolute log2FC > 0.6 were considered differentially expressed genes (DEGs). Volcano plots and heatmaps were constructed using “heatmap” and “ggplot2” packages in R, respectively.

### 2.3 Gene set enrichment analysis

Gene ontology (GO) enrichment analysis, KEGG pathway analysis, and DO method combined with “cluster Profiler” in R and the DOSE program were used to study DEGs. The GSEA technique allows for the identification of the most important functional terms in patients with IBD and control groups. “c2.cp.kegg.v7.0.symbols.gm t” is a criterion used for mymbols.gm t. The gene cluster is considered significantly aggregated if *P* < 0.05 or false issue rate < 0.025.

### 2.4 Candidate diagnostic biomarker screening

Three machine learning methods are used to predict patients’ conditions to identify the main prognostic variables. The least absolute compression and selection operation (LASSO) is a new approach that uses regularization methods to improve forecast accuracy. In R, the LASSO regression algorithm uses “glmnet” grouping to identify genetic factors that are significantly associated with IBD and control samples. Support vector machine (SVM) is one of the most widely used supervised machine learning methods. The metadata sequences are optimized using recursive feature elimination (RFE) methods to prevent duplicate screening. SVM-RFE was used to screen for suitable features to identify the set of gene pools with the highest discrimination power. Then “randomForest” in R was used to implement the random tree algorithm. Finally, the intersection was obtained through the Venn diagram package.

### 2.5 Discovery of immune cell subtypes

To quantify the rate of invasive immune cells in the gene expression profile of IBD, a bioinformatics algorithm called CIBERSORT (https://cibersortx.stanford.edu/) was used to estimate the invasion rate of the immune system. The number of an immune cell type was estimated using a reference system containing 22 isoforms (LM22) for 1000 permutations. A total of 22 infiltrating immune cell types were correlated using “corrplot” in R. Violin charts were used to represent the infiltration of immune cells in IBD and control samples using “vioplot” in R.

### 2.6 Correlation analysis between identified genes and infiltrating immune cells

The relationship between identified genetic markers and invading immune cells was evaluated using Spearman’s hierarchical correlation in R. The correlations were generated using the graphical technique in the “ggplot2” suite.

### 2.7 Construction of the mouse model of chronic colitis and experimental design

Male C57 mice (22–24 g), aged ~6 weeks, were purchased from Ltd. in Jiangsu, China. After 1 week of acclimatization, the rats were randomly divided into two groups (n = 6 per group): normal control and dextran sulfate (DSS) groups. For the DSS group, 1.5% (w/v) DSS (36000–50000 Da, China Eason Biochemical Technology Co., Ltd.) was prepared by dissolving in sterilized water, filtered through a 0.22-μm filter, and provided to the rats in the DSS group. The DSS solution was reconfigured every other day. Rats in the control group drank sterilized fresh water and bred in the same facility. A mouse model of chronic colitis lasted for three weeks per period, and after three periods, the mice were euthanized. The mice was weighed every two days, and the blood in the feces and changes in character were evaluated. All mice were euthanized by cervical dislocation. The colon was collected, and its length and thickness were measured.

### 2.8 HE staining and histological evaluation of colonic damage

Colonic tissue was fixed in 4% methylal solution and left overnight. Then the tissue was fixed and embedded in paraffin and cut into slices with a thickness of ~3 mm. Then after dewaxing, dehydrating, HE staining, dehydrating, and making the samples transparent, the slides were covered with a cover slip coated with neutral gum and sealed. The lesions were observed and recorded under an optical microscope. Lesions and inflammatory cell infiltration were evaluated in colon tissue. Then colon damage was determined histologically according to the scoring criteria of the histological examination provided in **Supplementary Table 2**.

### 2.9 Immunofluorescence

Immunofluorescence for MMP-9, PTGDS, the activating NK cell receptor CD160 and TLR5 and SLC26A8 on colonic tissue was performed using standard methods. Colonic sections were deparaffinized and rehydrated. Then antigen retrieval was performed by continuous heating with citrate buffer in a pressure cooker at 98°C for 10 min. The sections were then blocked-in normal serum and labeled with primary antibodies in blocking solution overnight at 4°C. After washing with PBS, the sheet was ligated to Alexa Fluor-488 or Cy-3. The sections were then examined with a fluorescence microscope (Olympus DP72 Microscopic imaging system).

### 2.10 Flow cytometry

The spleen was cut into pieces with sterilized surgical scissors and put into centrifuge tubes, digested with an appropriate amount of trypsin, and centrifuged at 1000 rpm for 5 min. Then 1 × 10^6^ cells were collected and resuspended with appropriate amount of flow staining buffer, and 5 μL of each antibody was added to the final reaction volume of 100 μL. Anti-CD16/CD32, -CD8, -CD25, and -CD56 antibodies were purchased from Multisciences. The cells were mixed by shaking and incubated for 20 min in the dark at room temperature. Then 1 mL of flow staining buffer was added to each tube. The tube was centrifuged at 300 × *g* for 10 min and the supernatant was discarded. Then 500 μL of flow staining buffer was added to the tube to resuspend the cells and a flow cytometer (Beckman Coulter, Inc.) was used for detection.

### 2.11 Western blotting

Colonic tissue was cut and placed in EP tubes, and RIPA lysis buffer (Epizyme Biomedical Technology, Shanghai, China) was added at 100 mg/mL (containing 1% phosphatase inhibitor and 1% PMSF). The sample was homogenized with a tissue homogenizer until no visible pieces remained. Then lysis was performed on ice for 30 min. The supernatant was collected and total protein concentration was evaluated using a bicinchoninic acid kit (TransGen Biotech, Beijing, China). The samples were run using 12% SDS-PAGE and transferred to polyvinylidene difluoride membranes. Then, the membranes were blocked with QuickBlock Blocking Buffer (Beyotime Biotechnology in Shanghai, China) for 15 min, incubated with diluted primary antibodies overnight at 4°C, washed three times with TBST, and incubated for 1 h with secondary antibodies. TLR5 (19810-1-AP) and SLC26A8 (12776-1-AP) were purchased from Proteintech (Wuhan, China). MMP-9 (TA5228S) was purchased from Abmart Technology (Shanghai, China). PTGDS was purchased from Solarbio Life Science (Beijing, China) and CD160 from Affinity Biosciences (Suzhou, China). Goat anti-rabbit IgG (H+L) HRP (BL003A) was purchased from Biosharp (Hefei, China).

## 3 Results

### 3.1 Identification of DEGs in IBD

**Figure 1:**
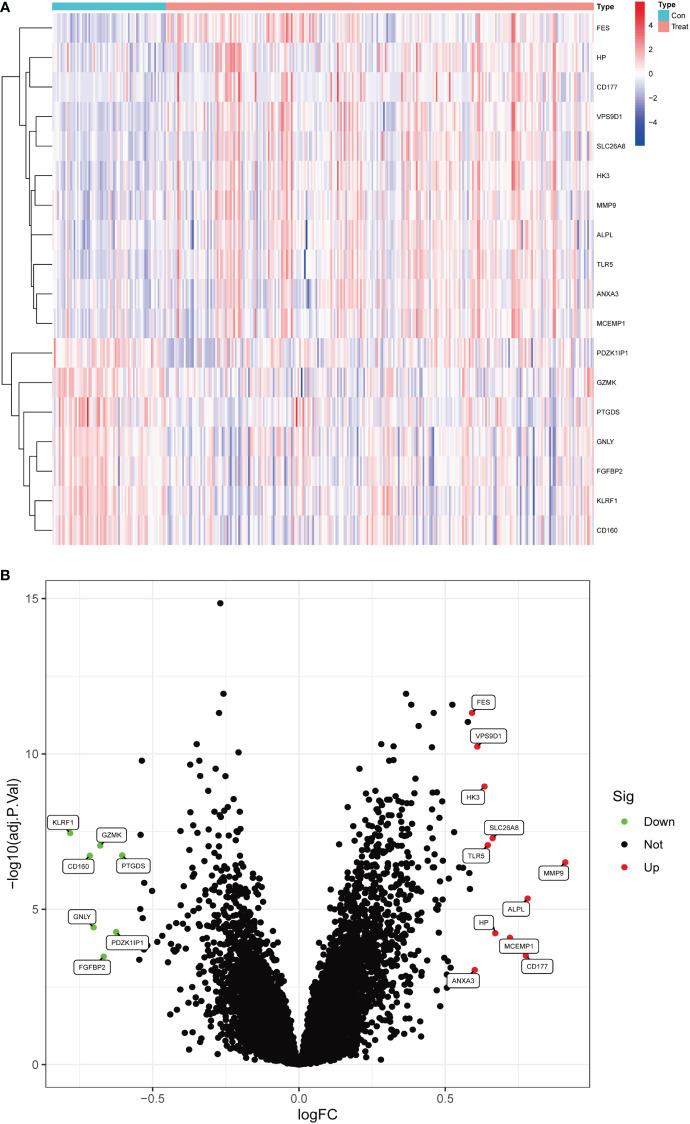
Differentially expressed genes in normal and inflammatory bowel disease (IBD). **(A)** Volcano plot of differentially expressed genes. Red dots represent significantly upregulated genes and green dots indicate significantly downregulated genes. **(B)** Heatmap of differentially expressed genes.

In this study, data were collected from three GEO data sets, namely GSE94648 and GSE119600, for 263 IBD and 69 control samples, respectively. After correcting for batch processing, metadata DEGs were parsed using the limma software. The results showed that 18 DEGs could be obtained by this method. Furthermore, 11 genes were significantly upregulated and 7 were significantly downregulated (**Figures 1A, B**). Subsequently, the DEGs were intersected with 3970 metalloproteinase-related genes, and 9 differentially expressed metalloproteinase-related genes (MRDEGs) were finally obtained.

### 3.2 Functional correlation analysis

**Figure 2:**
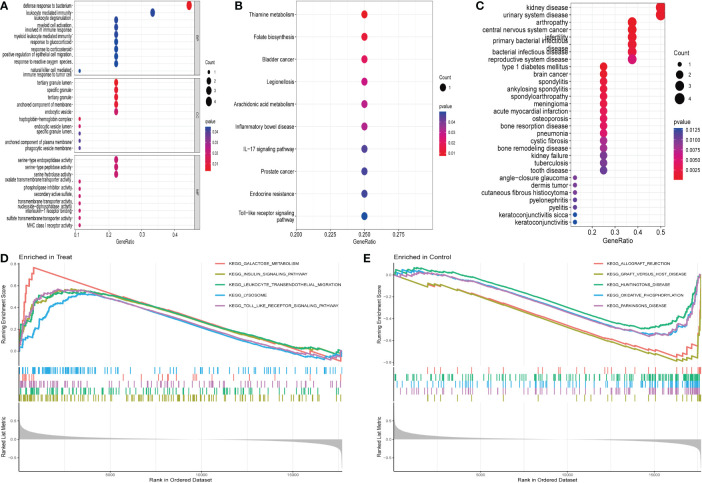
Enrichment analysis of differentially expressed genes: **(A)** GO enrichment analysis. **(B)** KEGG enrichment analysis. **(C)** Disease ontology enrichment analysis. **(D, E)** GSEA enrichment analysis.

The MRDEGs were initially evaluated using GO enrichment, KEGG pathway analysis, and DO pathway enrichment analysis. The results revealed that 205 biological processes, 10 signaling pathways, and 127 diseases were significantly enriched. The biological processes were enriched for positive control of defense response to bacterium, leukocyte mediated immunity, leukocyte degranulation, and myeloid cell activation involved in immune response. In the cellular component, tertiary granule lumen, specific granule, tertiary granule, and anchored component of membrane were enriched. In addition, a significant enrichment of serine-type endopeptidase activity, serine-type peptidase activity, serine hydrolase activity, and oxalate transmembrane transporter activity in molecular function (**Figure 2A**) was observed. KEGG enrichment analysis showed that thiamine metabolism, folate biosynthesis, bladder cancer, legionellosis, arachidonic acid metabolism, and IBD were enriched (**Figure 2B**). DO analysis revealed that kidney disease, urinary system disease, arthropathy, central nervous system cancer, infertility, and primary bacterial infectious disease were significantly enriched (**Figure 2C**). In addition, enrichment differences were further investigated between IBD and control groups using GSEA. In IBD, Galactose metabolism, insulin signaling pathway, leukocyte transendothelial migration, lysosome, the toll-type receptor signaling pathway is an important area of aggregation. In the control group, graft rejection, graft-versus-host disease, Huntington’s disease, oxidative phosphorylation, and Parkinson’s disease were significantly enriched (**Figures 2D, E**).

### 3.3 Identification and validation of diagnostic feature biomarkers

**Figure 3:**
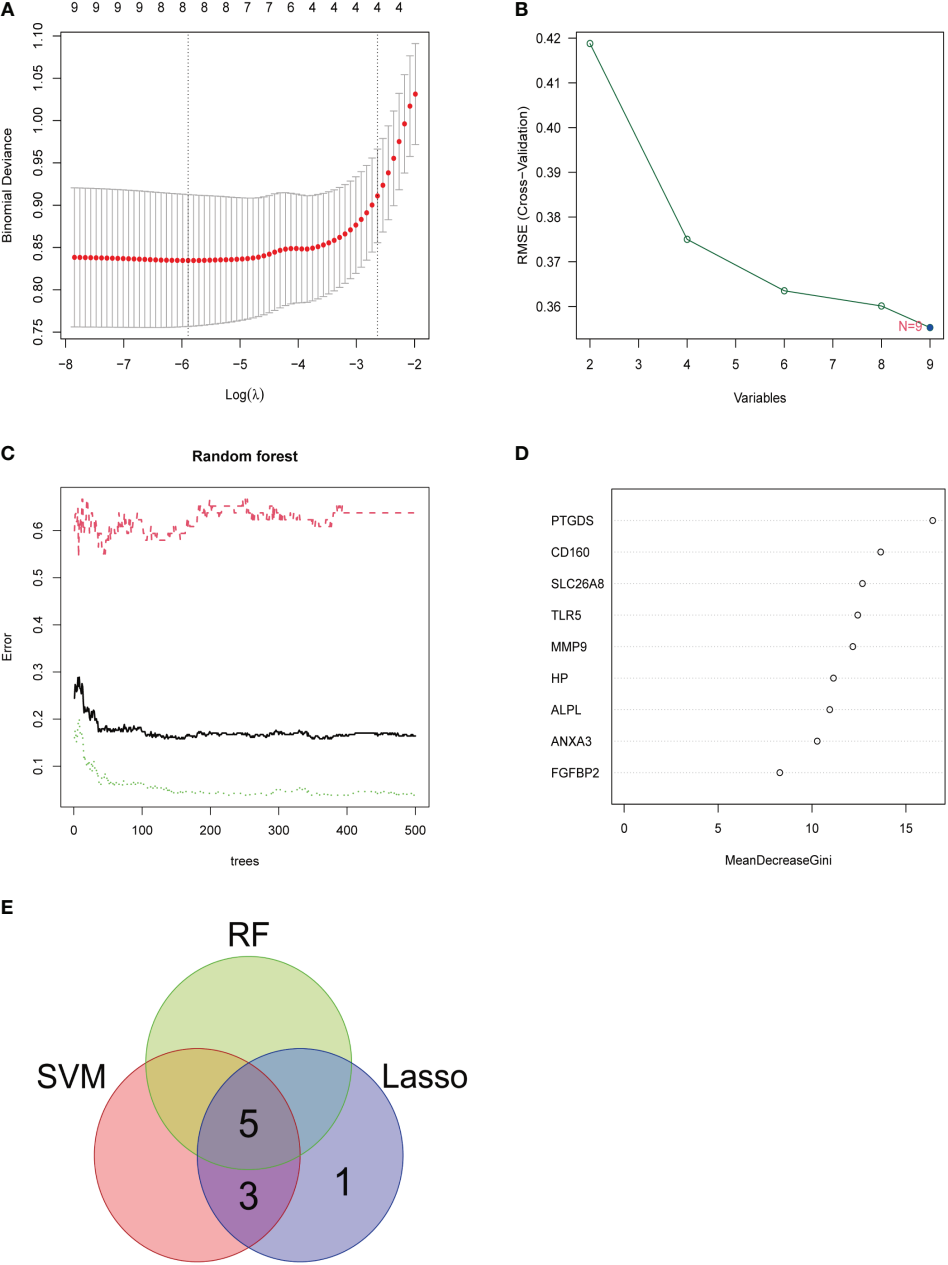
LASSO, SVM-RFE, and random forest were used for feature selection. **(A)** LASSO coefficient profiles of eight genes that initially met the prognostic criteria. **(B)** Biomarker selection map based on the support vector machine recursive feature elimination (SVM-RFE) algorithm. **(C)** Random forest model. **(D)** Random forest MeanDecreaseGini assessment. **(E)** Venn diagram shows the five diagnostic markers shared by the three algorithms.

**Figure 4:**
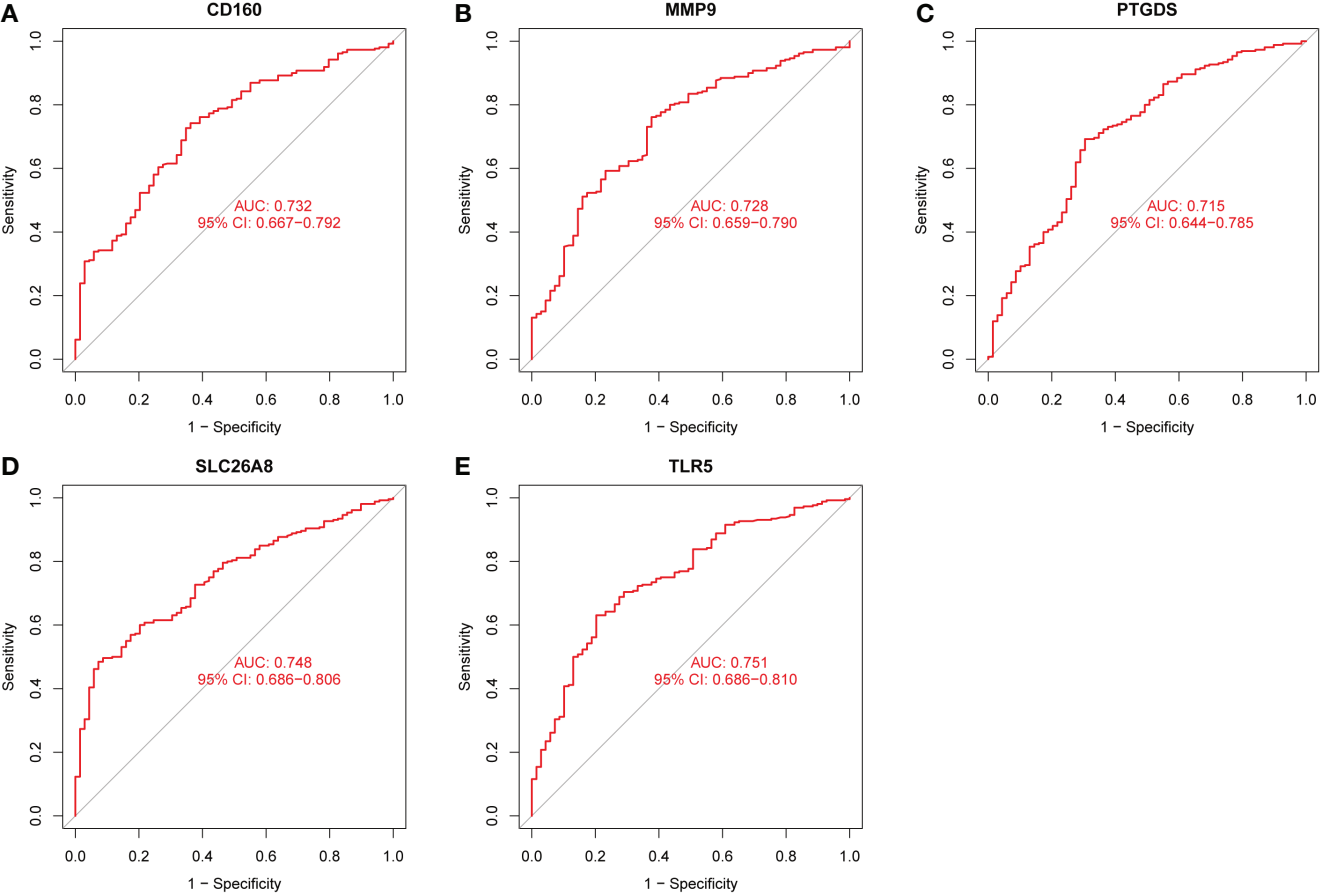
The receiver operating characteristic (ROC) curve of the diagnostic effectiveness of the five diagnostic markers. **(A)** ROC curve of CD160. **(B)** ROC curve of MMP9. **(C)** ROC curve of PTGDS. **(D)** ROC curve of SLC26A8. **(E)** ROC curve of TLR5.

Three methods were used for detecting possible biological markers. The application of MRDEGs was investigated using the LASSO regression method and eight biochemical indicators were identified that could be used for the diagnosis of IBD (**Figure 3A**). MRDEGs were identified using the SVM-RFE method for four feature points (**Figure 3B**). In addition, the features of the top 5 were obtained by the random forest algorithm (**Figures 3C, D**). Finally, the intersection of the three algorithms was used to obtain five related metalloproteinases (**Figure 3E**). These included CD160, MMP-9, PTGDS, SLC26A8, and TLR5. Subsequently, the accuracy of the relevant genes was evaluated by machine learning as disease diagnostic genes using the ROC curve (**Figures 4A–E**). The AUC values of the five related genes were 0.732, 0.728, 0.715, 0.748, and 0.751, respectively, showing high sensitivity and specificity.

### 3.4 Immune cell infiltration

**Figure 5:**
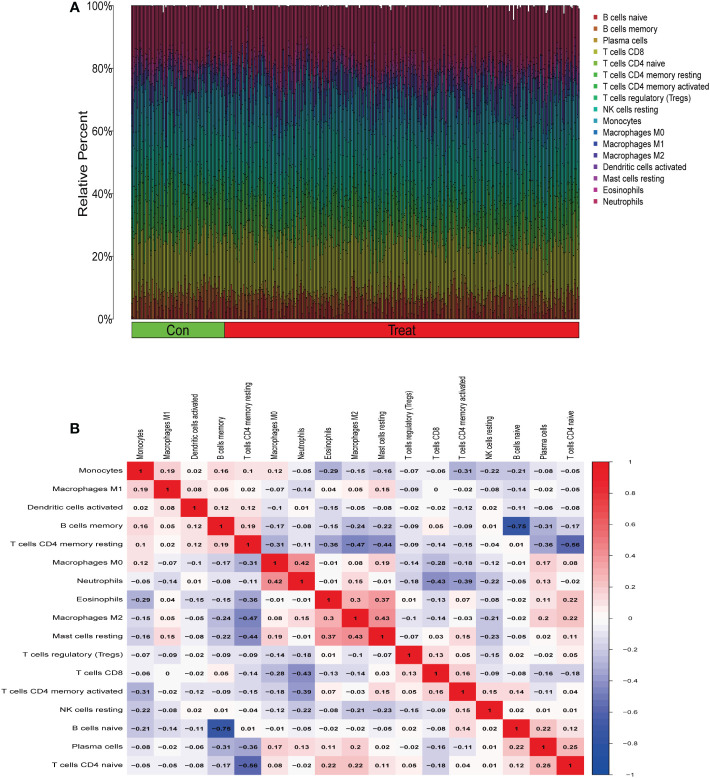
Immune infiltration analysis. **(A)** Immune cell distribution map. **(B)** Correlation of immune cells between patients with inflammatory bowel disease (IBD) and normal patients.

**Figure 6:**
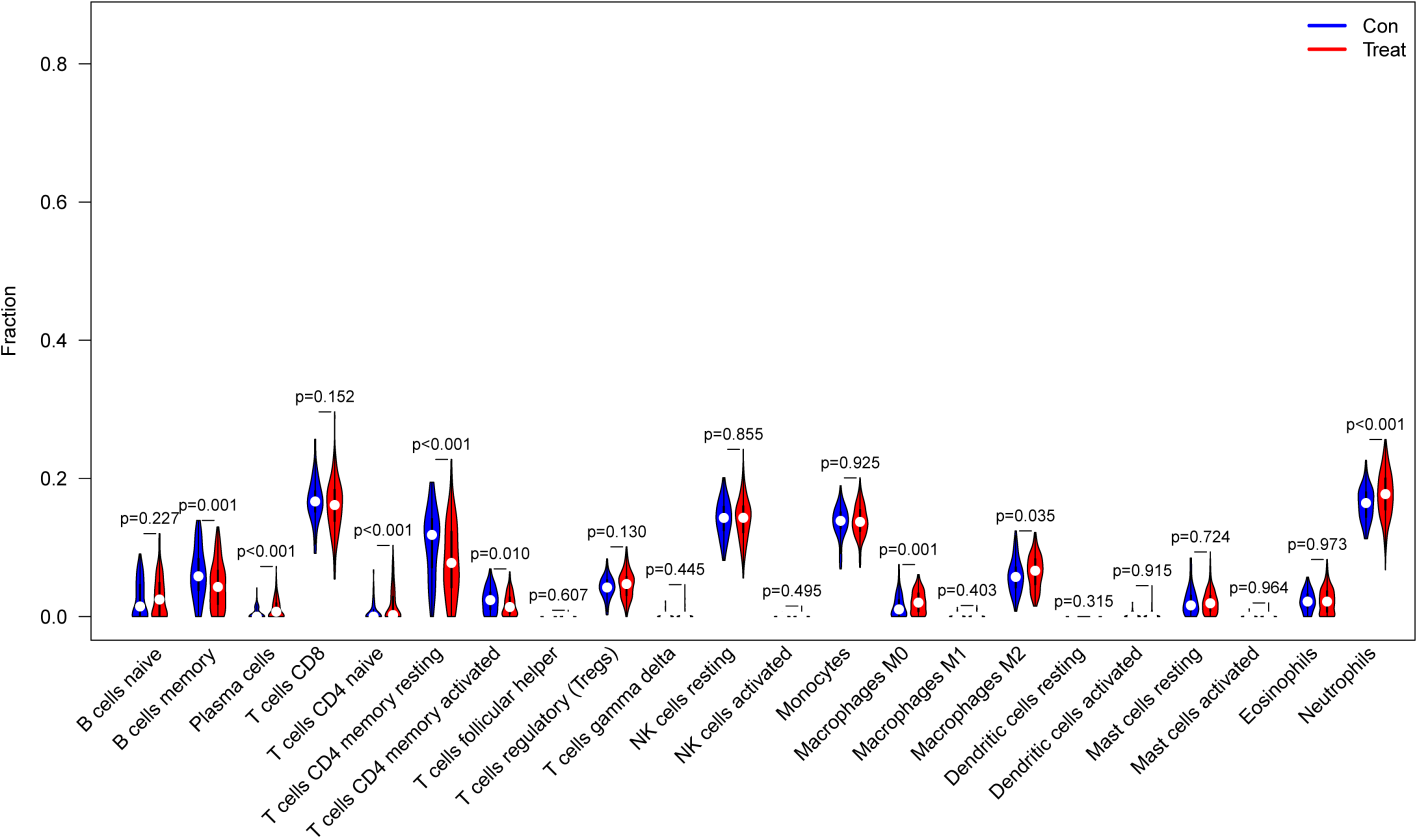
Expression of immune cells between patients with inflammatory bowel disease (IBD) and normal patients.

From the results of the analysis, a map was made that showed how immune cells are distributed (**Figure 5A**) and then a preliminary discussion of the immune cell component of IBD was provided. To investigate the correlation between immune cell expression, correlation analysis was performed. The results showed the relationship between the expression of immune cells in the combined data (**Figure 5B**). Immune cell differential analysis revealed that in IBD, the expression of plasma cells, T cells CD4 naïve, T cells CD4 memory resting, and neutrophils was higher and B cells memory, T cells CD4 memory activated, macrophages M0, and macrophages M2 was lower than that in the control group (**Figure 6**).

### 3.5 Correlation analysis between the five biomarkers and infiltrating immune cells

**Figure 7:**
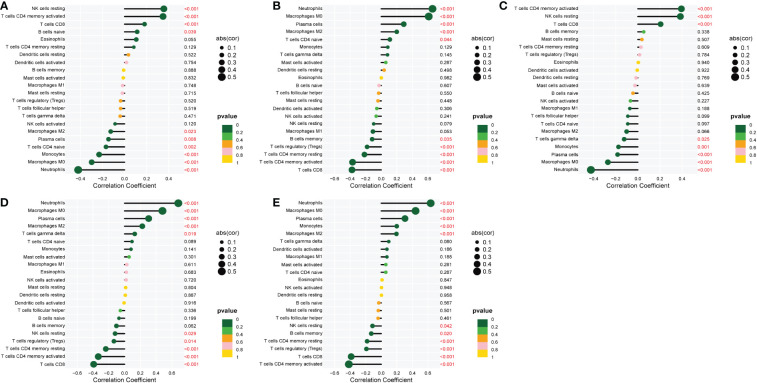
Correlation between five metalloproteinase-related genes and infiltrating immune cells. **(A)** Correlation between CD160 and infiltrating immune cells. **(B)** Correlation between MMP-9 and infiltrating immune cells. **(C)** Correlation between PTGDS and infiltrating immune cells. **(D)** Correlation between SLC26A8 and infiltrating immune cells. **(E)** Correlation between TLR5 and infiltrating immune cells.

The correlation of the five biological markers with each immune cell type was investigated (Appendix 5). The five biomarkers were positively correlated with NK cells resting, Neutrophils, and T cells CD4 memory activated, respectively, while negatively correlated with Neutrophils, T cells CD8 and T cells CD4 memory activated (**Figures 7A–E**).

### 3.6 Chronic colitis model evaluation and histological identification

**Figure 8:**
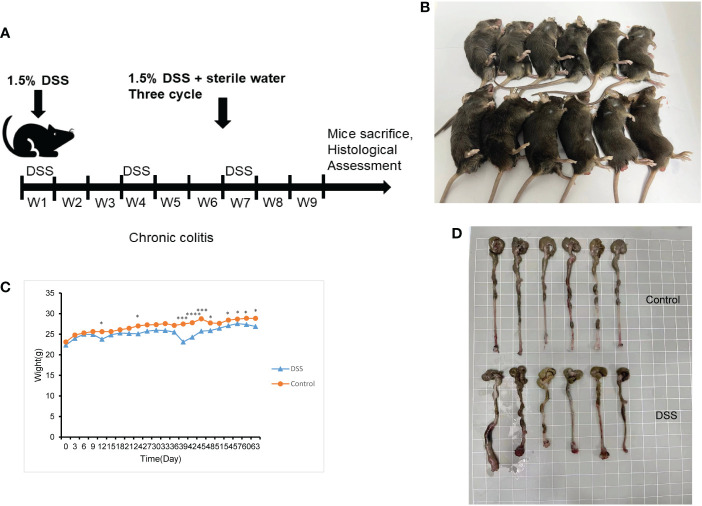
The construction of the inflammatory bowel disease (IBD) model. **(A)** Flow chart for mice feeding. **(B)** Irregular perianal prolapse in mice. **(C)** Body weight changes in mice. **(D)** Comparison of the length of colon. ^*^ *P* < 0.05, ^***^ *P* < 0.001, ****P < 0.0001. .

**Figure 9:**
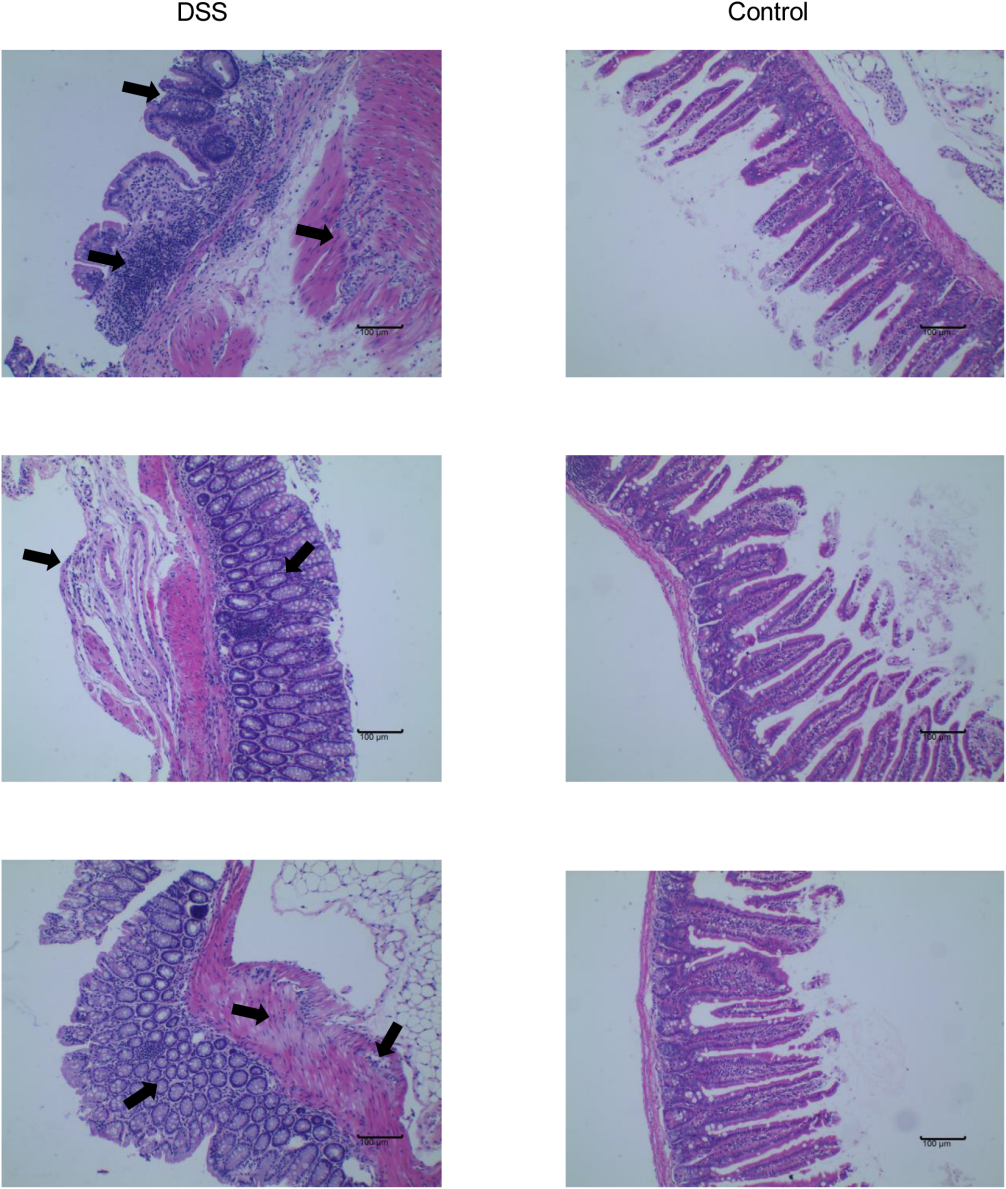
Comparison of HE staining between the DSS and control groups.

The feeding process of mice is shown in **Figure 8A**. The mice in the DSS group had redness and swelling around the anus. Due to the proliferation and swelling of the perianal mucosa, some mice showed adenomatous changes, with obvious redness and swelling and irregular prolapse of the anus (**Figure 8B**). Analysis of change in body weight revealed that the body weights of the mice decreased at the initial stage of administration, and in the subsequent modeling, the body weights of the mice in the DSS group increased at a lower rate than that in the control group (**Figure 8C**). After the mice were euthanized, the cecum was dissected to the anal hilum, stretched and spread spontaneously, and placed flat on A4 paper. When the length of each colon was measured, the intestinal length of the DSS group was found to be shortened (**Figure 8D**). The control group showed normal colonic structure with smooth mucosal surface and absence of annular folds and villi. The DSS group mainly showed typical manifestations of inflammatory mucosa, including infiltration of a large number of inflammatory cells in the mucosa, submucosa, and muscle layer; atypical gland hyperplasia; structural disorder; submucosal hemorrhage; and edema (**Figure 9**).

### 3.7 Expression of immune cells in the spleen

**Figure 10:**
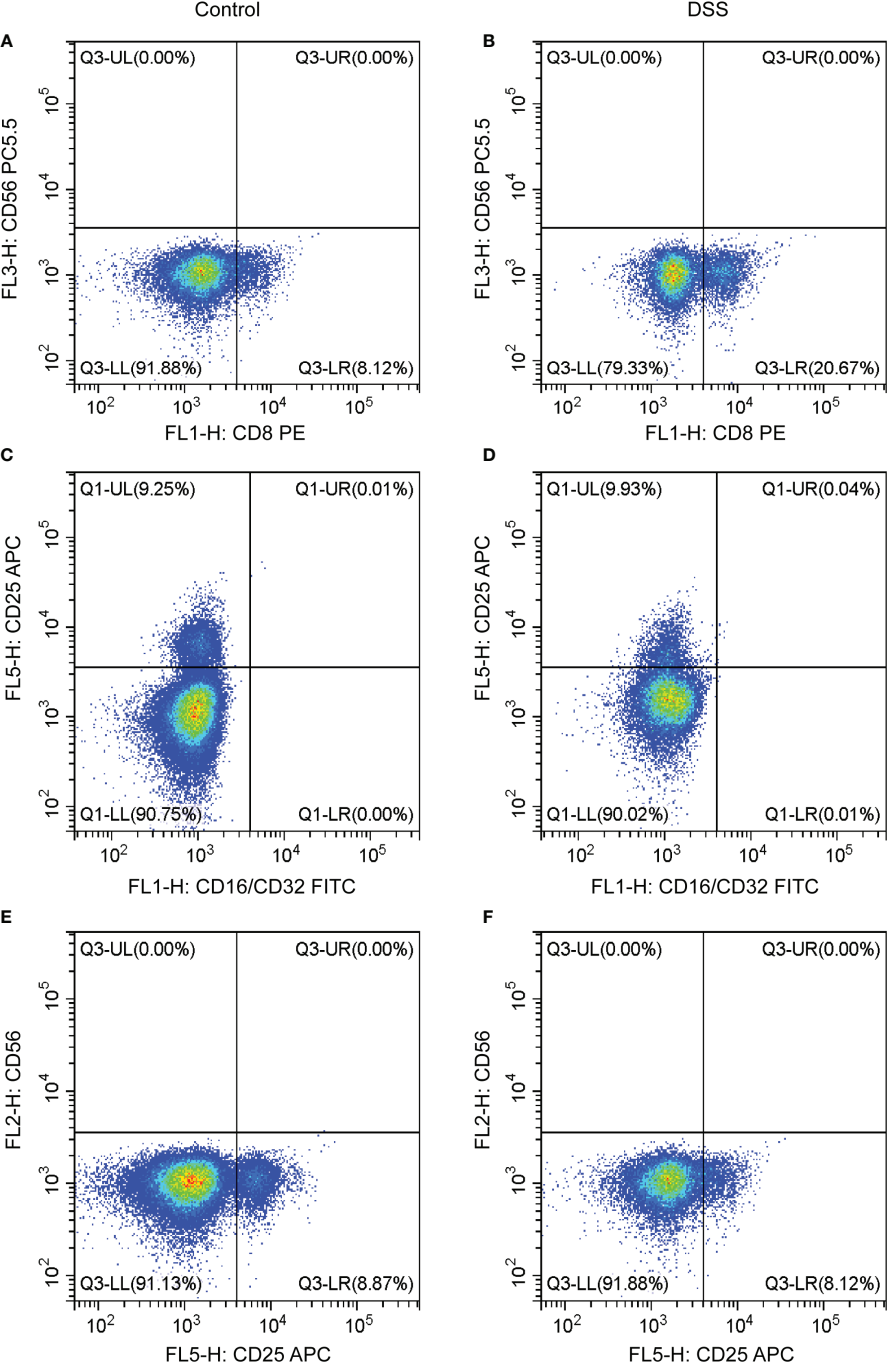
Effects of chronic inflammatory bowel disease (IBD) on immune cells in the spleen of mice. **(A–F)** The strategy of CD8, CD56, CD16/CD32, and CD25 in the DSS and control groups.

The results of flow cytometry showed that the expression of the T cell CD8 marker (CD8 antibody) was different between the DSS and control groups, whereas that of other neutrophil markers (CD16/32) and T cells CD4 memory activated markers (CD25 and CD65) were not different (**Figures 10A–F**). CD8 was negatively correlated with MMP-9 and SLC26A8.

### 3.8 Validation of inflammatory factors and MMPs associated with IBD

**Figure 11:**
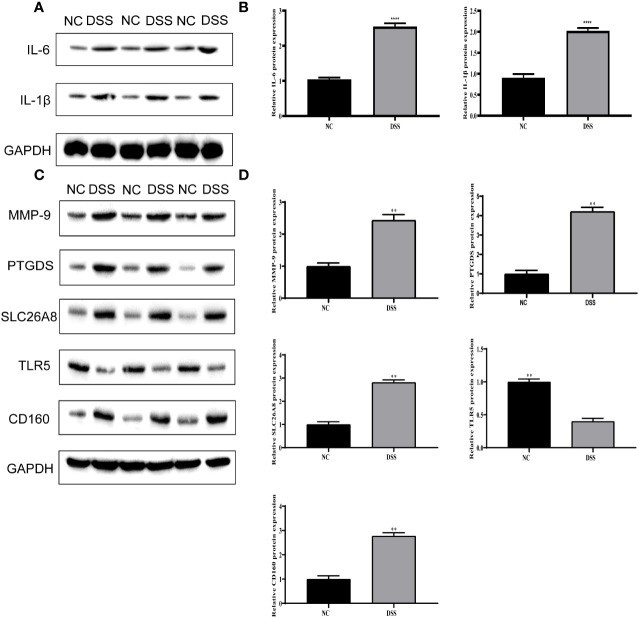
Validation of differential expression of inflammatory factors and five biomarkers. **(A)** Protein expression of IL-6 and IL-1β. **(B)** Protein statistics of IL-6 and IL-1β. **(C)** Protein expression of five biomarkers. **(D)** Protein statistics of five biomarkers. **P < 0.01, ****P < 0.0001.

**Figure 12:**
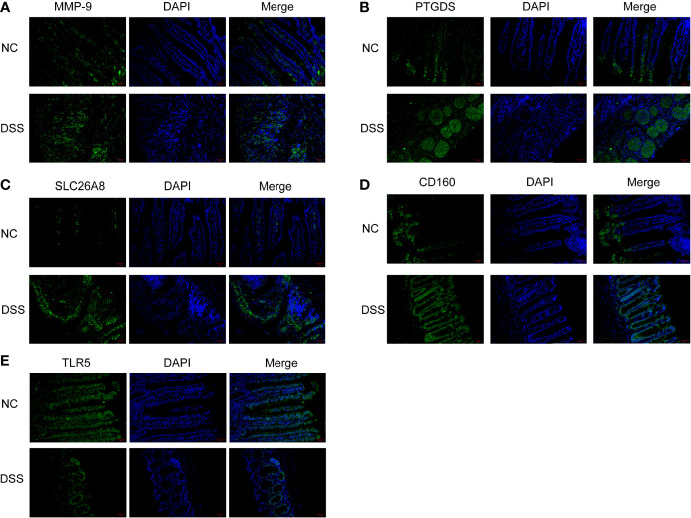
Colonic tissue immunofluorescence in mice. **(A–E)** Immunofluorescence of CD160, MMP-9, PTGDS, SLC26A8, and TLR5.

We verified the increased expression of inflammatory factors (IL-6 and IL-1β) in DSS group, which indicate that MMPs are closely related to inflammatory response (**Figures 11A, B**). Changes in the levels of MMP-related genes were evaluated in chronic IBD models. Compared with the control group, the expression levels of MMP-9, PTGDS, SLC26A8, and CD160 significantly increased, whereas that of TLR5 decreased in the DSS group, suggesting that the MMPs play a role in the development of the chronic inflammatory intestinal disease mouse model (**Figures 11C, D**). The results of immunofluorescence assay showed that compared with the control group, the fluorescence intensity of MMP-9, PTGDS, SLC26A8, and CD160 increased, whereas that of TLR5 decreased (**Figures 12A–E**) in the DSS group, which is consistent with the results of western blotting.

## 4 Discussion

IBD is an autoimmune disease of the intestine and includes ulcerative colitis and Crohn’s disease (25). As an important causative factor of colorectal cancer, IBD has attracted global attention. However, due to the increasing incidence, long course, and delayed healing in IBD, its diagnosis, treatment, and prognosis have become a challenge. The main feature of IBD is the chronic inflammatory reaction in the intestine (26), which induces immune stimulation of the mucosa and makes the intestinal environment abnormal (27, 28). Therefore, the immune function of the intestinal barrier is crucial for the prevention and resolution of intestinal inflammatory diseases.

MMPs are a group of proteolytic enzymes containing active zinc. MMPs can be divided into groups based on the structure of the catalytic region (29). They are classified according to substrate and fragment homology, such as collagenases, gelatinases, stromelysins, elastases, and membrane-type MMPs (30). Dysregulation of MMP expression can cause tissue damage and persistent inflammation. Some studies have demonstrated the antitumor effects of MMPs. Various MMPs are associated with the poor prognosis of tumors (31). Cancer cells can evade the immune system by using MMPs to ensure the survival of metastatic cells (32). In addition to the regulatory role of MMPs in cancer, MMPs are also involved in the inflammatory response of many inflammatory diseases, such as sepsis (33), atherosclerosis (34, 35), and arthritis (36).

In this study, a mouse model of chronic IBD induced by DSS was constructed to evaluate the relationship between immune cells in IBD and MMPs. Histologically, chronic colitis is characterized by shortened colon length and loss of goblet cells and crypts. Additionally, some mice may have adenomatous polyps and tumor-like changes. During the 9 weeks of modeling, mice showed weight loss and mucus and blood in stool. Some mice showed adenomatous changes due to the proliferation and swelling of the perianal mucosa, and obvious redness, swelling, and irregular prolapse in the anus. The histological score was statistically different from that of the control group. Thus, the model of chronic IBD was validated.

In this study, a clustering approach was used to identify the types of differentially expressed metalloproteinase-related genes. Toll-type receptors (TLRs) are an important source of IBD. TLR is a natural immune system receptor that contributes to the pathogenic mechanisms of IBD, including immune response, genetics, and microbiology (37). The TLR signal transduction pathway can induce various factors involved in defense, such as inflammatory factors, chemokines, and antigen presenting factors (38, 39). Inflammatory cytokines play a crucial role in multiple processes of IBD development, when TLR pathway is activated, immune cells will produce a large number of pro-inflammatory factors, such as IL-1β, IL-6.This was also verified in our experiment. According to the results of Western Blot, the expression of inflammatory cytokines in the DSS group was significantly higher than that in the other.

On this basis, CD160, MMP-9, PTGDS, SLC26A8, and TLR5 were selected as indicators of tumor detection. A survey by Marônek et al. (40) showed that reduced MMP-9 in patients with IBD is a risk factor in patients with IBD and infection (41). Liu et al. studied MMP-9 in excreta using nanoparticles (42). A study reported that TLR5 plays a key role in the inflammatory response in the intestine through animal tests on TLR5 and that disruption of TLR5 signaling triggers the TNFR2 signaling pathway, which leads to an inflammatory response in the intestine (43). CD160 activates natural killer cells with specific domains, making it a novel therapeutic target in the fight against atherosclerosis, autoimmune diseases, and many cancers (44). In recent years, researchers have shown that CD160 is associated with the recovery of COVID-19 patients (45). PTGDS have been shown to be selectively expressed in cancers, including ovarian cancer (46) and melanoma (47) with overexpression, gastric cancer (48) and lung cancer (49) with low expression. This selective expression also indicates that PTGDS has a more complex mechanism and potential research value. A study has shown that SLC26A8 is a susceptibility gene for hereditary non-polyposis colorectal cancer (50), which also provides reference for our subsequent research on the development of IBD for colorectal cancer. In this study, high levels of MMP-9, PTGDS, SLC26A8, and CD160 and reduced levels of TLR5 protein in patients with IBD (identified using WB and immunofluorescence analysis) suggest that these are clinically significant in IBD.

In the pathogenesis of many chronic diseases, both innate and adaptive immune functions are affected to some extent. A comparison of different cell types identified high levels of plasma cells, T-cell CD4 naive, T-cell CD4 memory, and eosinophils. Mitsialis et al. (51) reported that the abundance of HLA-DR+CD38+ T lymphocytes increased in colonic mucosal samples from patients with IBD, where T-regulatory cells were also present. Based on the relationship of the five antigens different cell types, we suggest that there are differences in the function of CD4 of neutrophils and T cells in the pathogenesis of IBD, which is related to the non-homogeneity of cells. However, all but CD8 were ineffective at elevated expression levels in IBD. Recent data show that CD8+ T lymphocytes (Tc1) and CD8+ (Tc17) play an important role in the development of IBD (52).

In conclusion, the findings of this study show that MMPS-related genes, namely MMP-9, CD160, PTGDS, SLC26A8, and TLR5, regulate the occurrence and development of IBD. These findings provide insights into future research on the mechanism of IBD. In addition, bioinformatics and mouse model studies revealed that MMP-related genes can participate in the progression of IBD by regulating CD8 cells. However, the detailed mechanism of action of MMPs in IBD is unknown and requires further investigation.

## 5 Conclusion

The findings of this study show that patients with IBD and healthy controls have significantly different gene expression, identifying the role of metalloproteinases in IBD. Moreover, the results provide insights into immune cell activation through metalloproteinases in IBD. However, more in-depth research is needed in future studies.

## Data availability statement

The original contributions presented in the study are included in the article/**Supplementary Material**. Further inquiries can be directed to the corresponding authors.

## Ethics statement

The studies involving human participants were reviewed and approved by The Committee on the Ethics of Nanjing Normal University, Nanjing Normal University. The ethics committee waived the requirement of written informed consent for participation. The animal study was reviewed and approved by All in vivo animal experiments were approved by the Committee on the Ethics of Animal Experiments of Nanjing Normal University (IRB#2020–0047), Nanjing Normal University.

## Author contributions

All authors made a significant contribution to the work reported, whether that is in the conception, study design, execution, acquisition of data, analysis and interpretation, or in all these areas; took part in drafting, revising or critically reviewing the article; gave final approval of the version to be published; have agreed on the journal to which the article has been submitted; and agree to be accountable for all aspects of the work.

## Supplementary material

Supplementary Table 1 Metalloproteinase-related genes.

Supplementary Table 2 Scoring criteria for the histological examination of mice.
