# Supplementary material for: The role of intestinal immune cells and matrix metalloproteinases in inflammatory bowel disease
Source: Front Immunol. 2023 Jan 17;13:1067950. doi: 10.3389/fimmu.2022.1067950 (PMC9888429; doi:10.3389/fimmu.2022.1067950)
Supplement: Supplementary Table 1 — Metalloproteinase-related genes. [file Table_1.docx]

Supplementary Material

Table 1 Metalloproteinase related genes

| Gene Symbol | Description | Category | Gifts | GC Id | Relevance score |
| --- | --- | --- | --- | --- | --- |
| MMP9 | Matrix Metallopeptidase 9 | Protein Coding | 56 | GC20P046008 | 58.14687347 |
| MMP2 | Matrix Metallopeptidase 2 | Protein Coding | 55 | GC16P055390 | 51.87375259 |
| MMP1 | Matrix Metallopeptidase 1 | Protein Coding | 54 | GC11M102810 | 45.64362335 |
| TIMP1 | TIMP Metallopeptidase Inhibitor 1 | Protein Coding | 47 | GC0XP047647 | 44.55200958 |
| MMP3 | Matrix Metallopeptidase 3 | Protein Coding | 53 | GC11M102835 | 39.42826843 |
| TIMP2 | TIMP Metallopeptidase Inhibitor 2 | Protein Coding | 45 | GC17M078852 | 38.82058716 |
| MMP14 | Matrix Metallopeptidase 14 | Protein Coding | 53 | GC14P033595 | 35.18500137 |
| MMP13 | Matrix Metallopeptidase 13 | Protein Coding | 54 | GC11M102942 | 29.98983002 |
| MMP7 | Matrix Metallopeptidase 7 | Protein Coding | 50 | GC11M102425 | 29.09084892 |
| TIMP3 | TIMP Metallopeptidase Inhibitor 3 | Protein Coding | 46 | GC22P041082 | 28.08011055 |
| MMP8 | Matrix Metallopeptidase 8 | Protein Coding | 51 | GC11M102617 | 25.94309998 |
| MMP12 | Matrix Metallopeptidase 12 | Protein Coding | 50 | GC11M102862 | 22.24446297 |
| TIMP4 | TIMP Metallopeptidase Inhibitor 4 | Protein Coding | 43 | GC03M012153 | 20.02807617 |
| MMP10 | Matrix Metallopeptidase 10 | Protein Coding | 47 | GC11M102770 | 18.57862091 |
| MMP11 | Matrix Metallopeptidase 11 | Protein Coding | 47 | GC22P023768 | 17.68581963 |
| MMP16 | Matrix Metallopeptidase 16 | Protein Coding | 47 | GC08M088032 | 17.63358879 |
| BSG | Basigin (Ok Blood Group) | Protein Coding | 47 | GC19P000571 | 17.49012566 |
| MMP15 | Matrix Metallopeptidase 15 | Protein Coding | 48 | GC16P058025 | 17.37824631 |
| MMP19 | Matrix Metallopeptidase 19 | Protein Coding | 51 | GC12M055835 | 17.00338936 |
| MMP20 | Matrix Metallopeptidase 20 | Protein Coding | 47 | GC11M102576 | 16.06633759 |
| MMP24 | Matrix Metallopeptidase 24 | Protein Coding | 44 | GC20P035226 | 15.69730282 |
| MMP17 | Matrix Metallopeptidase 17 | Protein Coding | 47 | GC12P131828 | 15.19532394 |
| TNF | Tumor Necrosis Factor | Protein Coding | 54 | GC06P087731 | 14.39928436 |
| MMP25 | Matrix Metallopeptidase 25 | Protein Coding | 45 | GC16P012724 | 14.1684494 |
| MMP26 | Matrix Metallopeptidase 26 | Protein Coding | 40 | GC11P004706 | 14.0401516 |
| ADAM17 | ADAM Metallopeptidase Domain 17 | Protein Coding | 54 | GC02M009488 | 13.0700655 |
| MMP23B | Matrix Metallopeptidase 23B | Protein Coding | 41 | GC01P004137 | 12.78553963 |
| VEGFA | Vascular Endothelial Growth Factor A | Protein Coding | 51 | GC06P043770 | 12.09292412 |
| MMP28 | Matrix Metallopeptidase 28 | Protein Coding | 40 | GC17M035756 | 11.41615295 |
| ADAM15 | ADAM Metallopeptidase Domain 15 | Protein Coding | 47 | GC01P155050 | 11.37087345 |
| MMP21 | Matrix Metallopeptidase 21 | Protein Coding | 43 | GC10M125756 | 11.31661224 |
| ADAMTS5 | ADAM Metallopeptidase With Thrombospondin Type 1 Motif 5 | Protein Coding | 47 | GC21M026918 | 11.25030327 |
| RECK | Reversion Inducing Cysteine Rich Protein With Kazal Motifs | Protein Coding | 43 | GC09P036036 | 11.22923088 |
| MMP27 | Matrix Metallopeptidase 27 | Protein Coding | 40 | GC11M102596 | 10.91517162 |
| IL1B | Interleukin 1 Beta | Protein Coding | 50 | GC02M112829 | 10.67175865 |
| CD36 | CD36 Molecule | Protein Coding | 52 | GC07P080369 | 10.62501717 |
| ZMPSTE24 | Zinc Metallopeptidase STE24 | Protein Coding | 44 | GC01P040258 | 10.18413353 |
| ADAM10 | ADAM Metallopeptidase Domain 10 | Protein Coding | 55 | GC15M058588 | 10.16320229 |
| LOC100131553 | Disintegrin And Metalloproteinase Domain-Containing Protein 21-Like | Pseudogene | 2 | GC04P175016 | 10.12498093 |
| LOC102723815 | Disintegrin And Metalloproteinase Domain-Containing Protein 29-Like | Pseudogene | 2 | GC16M8D1305 | 10.12498093 |
| LOC283804 | Disintegrin And Metalloproteinase Domain-Containing Protein 21-Like | Pseudogene | 2 | GC15M021053 | 10.12498093 |
| LOC646071 | Disintegrin And Metalloproteinase Domain-Containing Protein 21-Like | Pseudogene | 2 | GC15M020046 | 10.12498093 |
| LOC124905509 | Disintegrin And Metalloproteinase Domain-Containing Protein 29-Like | Protein Coding | 1 | GC15Pr02835 | 10.12498093 |
| ADAMTS1 | ADAM Metallopeptidase With Thrombospondin Type 1 Motif 1 | Protein Coding | 48 | GC21M026835 | 9.714985847 |
| TGFB1 | Transforming Growth Factor Beta 1 | Protein Coding | 55 | GC19M041301 | 9.692890167 |
| ADAMTS4 | ADAM Metallopeptidase With Thrombospondin Type 1 Motif 4 | Protein Coding | 47 | GC01M161184 | 9.467726707 |
| MMP23A | Matrix Metallopeptidase 23A (Pseudogene) | Pseudogene | 19 | GC01P001699 | 9.459448814 |
| ADAM12 | ADAM Metallopeptidase Domain 12 | Protein Coding | 48 | GC10M126012 | 9.332700729 |
| PLAU | Plasminogen Activator, Urokinase | Protein Coding | 55 | GC10P073909 | 9.270526886 |
| IL6 | Interleukin 6 | Protein Coding | 53 | GC07P022725 | 8.738050461 |
| JUN | Jun Proto-Oncogene, AP-1 Transcription Factor Subunit | Protein Coding | 50 | GC01M058780 | 8.676464081 |
| ADAM9 | ADAM Metallopeptidase Domain 9 | Protein Coding | 51 | GC08P038996 | 8.538584709 |
| MAPK1 | Mitogen-Activated Protein Kinase 1 | Protein Coding | 54 | GC22M021759 | 8.128573418 |
| ADAM8 | ADAM Metallopeptidase Domain 8 | Protein Coding | 46 | GC10M133262 | 7.594799042 |
| ADAM33 | ADAM Metallopeptidase Domain 33 | Protein Coding | 43 | GC20M003669 | 7.547424316 |
| PTGS2 | Prostaglandin-Endoperoxide Synthase 2 | Protein Coding | 51 | GC01M186640 | 7.544478416 |
| CDH1 | Cadherin 1 | Protein Coding | 52 | GC16P068737 | 7.484108925 |
| ELN | Elastin | Protein Coding | 45 | GC07P074027 | 7.348781109 |
| ELANE | Elastase, Neutrophil Expressed | Protein Coding | 53 | GC19P003232 | 7.298573494 |
| ADAM28 | ADAM Metallopeptidase Domain 28 | Protein Coding | 44 | GC08P024294 | 7.265285492 |
| ADAMTS13 | ADAM Metallopeptidase With Thrombospondin Type 1 Motif 13 | Protein Coding | 51 | GC09P133414 | 7.264689922 |
| FURIN | Furin, Paired Basic Amino Acid Cleaving Enzyme | Protein Coding | 50 | GC15P090868 | 7.112979889 |
| EGFR | Epidermal Growth Factor Receptor | Protein Coding | 58 | GC07P055019 | 7.030836105 |
| CXCL8 | C-X-C Motif Chemokine Ligand 8 | Protein Coding | 44 | GC04P073740 | 6.998610973 |
| ADAM19 | ADAM Metallopeptidase Domain 19 | Protein Coding | 45 | GC05M157395 | 6.995690346 |
| ADAM11 | ADAM Metallopeptidase Domain 11 | Protein Coding | 43 | GC17P058668 | 6.819135189 |
| ADAMTS12 | ADAM Metallopeptidase With Thrombospondin Type 1 Motif 12 | Protein Coding | 43 | GC05M033524 | 6.818393707 |
| MAPK14 | Mitogen-Activated Protein Kinase 14 | Protein Coding | 54 | GC06P087896 | 6.725907803 |
| ADAMTS15 | ADAM Metallopeptidase With Thrombospondin Type 1 Motif 15 | Protein Coding | 38 | GC11P130448 | 6.378013611 |
| ADAMTS7 | ADAM Metallopeptidase With Thrombospondin Type 1 Motif 7 | Protein Coding | 43 | GC15M078759 | 6.29360342 |
| ADAMTS2 | ADAM Metallopeptidase With Thrombospondin Type 1 Motif 2 | Protein Coding | 47 | GC05M179110 | 6.2421875 |
| ADAM22 | ADAM Metallopeptidase Domain 22 | Protein Coding | 45 | GC07P087934 | 6.127068996 |
| ADAMTS17 | ADAM Metallopeptidase With Thrombospondin Type 1 Motif 17 | Protein Coding | 44 | GC15M099971 | 5.943881989 |
| EGF | Epidermal Growth Factor | Protein Coding | 54 | GC04P109912 | 5.937444687 |
| ADAMTS18 | ADAM Metallopeptidase With Thrombospondin Type 1 Motif 18 | Protein Coding | 45 | GC16M077316 | 5.902903557 |
| CD44 | CD44 Molecule (Indian Blood Group) | Protein Coding | 50 | GC11P035139 | 5.876925468 |
| ADAM23 | ADAM Metallopeptidase Domain 23 | Protein Coding | 44 | GC02P206443 | 5.863022804 |
| CCL2 | C-C Motif Chemokine Ligand 2 | Protein Coding | 51 | GC17P034255 | 5.801646709 |
| MAPK8 | Mitogen-Activated Protein Kinase 8 | Protein Coding | 52 | GC10P048306 | 5.798877716 |
| LCN2 | Lipocalin 2 | Protein Coding | 48 | GC09P128149 | 5.784149647 |
| ADAMTS14 | ADAM Metallopeptidase With Thrombospondin Type 1 Motif 14 | Protein Coding | 41 | GC10P070672 | 5.719256878 |
| IL17A | Interleukin 17A | Protein Coding | 47 | GC06P052186 | 5.663418293 |
| ETS1 | ETS Proto-Oncogene 1, Transcription Factor | Protein Coding | 50 | GC11M128458 | 5.592517376 |
| ETV4 | ETS Variant Transcription Factor 4 | Protein Coding | 45 | GC17M043527 | 5.570017338 |
| IL1A | Interleukin 1 Alpha | Protein Coding | 47 | GC02M112773 | 5.487671852 |
| SPOCK3 | SPARC (Osteonectin), Cwcv And Kazal Like Domains Proteoglycan 3 | Protein Coding | 38 | GC04M166733 | 5.418774128 |
| PLAUR | Plasminogen Activator, Urokinase Receptor | Protein Coding | 47 | GC19M043646 | 5.368176937 |
| IL13 | Interleukin 13 | Protein Coding | 48 | GC05P132656 | 5.363390446 |
| ADAMTS16 | ADAM Metallopeptidase With Thrombospondin Type 1 Motif 16 | Protein Coding | 41 | GC05P005140 | 5.345736504 |
| ADAMTS3 | ADAM Metallopeptidase With Thrombospondin Type 1 Motif 3 | Protein Coding | 43 | GC04M072280 | 5.324725628 |
| ADAMTS9 | ADAM Metallopeptidase With Thrombospondin Type 1 Motif 9 | Protein Coding | 41 | GC03M064501 | 5.299728394 |
| LEP | Leptin | Protein Coding | 49 | GC07P128241 | 5.276669502 |
| NFKB1 | Nuclear Factor Kappa B Subunit 1 | Protein Coding | 55 | GC04P102501 | 5.247723103 |
| HLA-G | Major Histocompatibility Complex, Class I, G | Protein Coding | 46 | GC06P087672 | 5.245210171 |
| FGF2 | Fibroblast Growth Factor 2 | Protein Coding | 48 | GC04P122826 | 5.194424152 |
| FN1 | Fibronectin 1 | Protein Coding | 52 | GC02M215360 | 5.179747105 |
| FOS | Fos Proto-Oncogene, AP-1 Transcription Factor Subunit | Protein Coding | 52 | GC14P075278 | 5.142933846 |
| CRP | C-Reactive Protein | Protein Coding | 48 | GC01M159728 | 5.10948658 |
| STAT3 | Signal Transducer And Activator Of Transcription 3 | Protein Coding | 56 | GC17M042313 | 5.103445053 |
| SERPINE1 | Serpin Family E Member 1 | Protein Coding | 52 | GC07P101127 | 5.059680462 |
| MAPK3 | Mitogen-Activated Protein Kinase 3 | Protein Coding | 51 | GC16M037779 | 5.058074951 |
| BMP6 | Bone Morphogenetic Protein 6 | Protein Coding | 46 | GC06P007726 | 5.024014473 |
| ADAMTS6 | ADAM Metallopeptidase With Thrombospondin Type 1 Motif 6 | Protein Coding | 41 | GC05M065148 | 5.018091679 |
| PTK2 | Protein Tyrosine Kinase 2 | Protein Coding | 50 | GC08M140657 | 5.011361122 |
| PITRM1 | Pitrilysin Metallopeptidase 1 | Protein Coding | 43 | GC10M003138 | 4.996874809 |
| ADAM30 | ADAM Metallopeptidase Domain 30 | Protein Coding | 40 | GC01M119893 | 4.921041965 |
| ADAM21 | ADAM Metallopeptidase Domain 21 | Protein Coding | 38 | GC14P070418 | 4.921041965 |
| ADAM20 | ADAM Metallopeptidase Domain 20 | Protein Coding | 37 | GC14M070522 | 4.902786255 |
| BMP1 | Bone Morphogenetic Protein 1 | Protein Coding | 51 | GC08P022164 | 4.892724037 |
| CXCL12 | C-X-C Motif Chemokine Ligand 12 | Protein Coding | 46 | GC10M044370 | 4.882019043 |
| ADAMTS19 | ADAM Metallopeptidase With Thrombospondin Type 1 Motif 19 | Protein Coding | 40 | GC05P129460 | 4.860230446 |
| IL10 | Interleukin 10 | Protein Coding | 50 | GC01M206767 | 4.835211754 |
| ITGAV | Integrin Subunit Alpha V | Protein Coding | 51 | GC02P186589 | 4.831234932 |
| ACAN | Aggrecan | Protein Coding | 48 | GC15P121030 | 4.831092834 |
| ADAMTS8 | ADAM Metallopeptidase With Thrombospondin Type 1 Motif 8 | Protein Coding | 44 | GC11M130404 | 4.823261738 |
| PLG | Plasminogen | Protein Coding | 52 | GC06P160702 | 4.781954288 |
| PAPPA | Pappalysin 1 | Protein Coding | 45 | GC09P119285 | 4.770225048 |
| ADAM29 | ADAM Metallopeptidase Domain 29 | Protein Coding | 40 | GC04P174831 | 4.754012108 |
| DDR1 | Discoidin Domain Receptor Tyrosine Kinase 1 | Protein Coding | 50 | GC06P087709 | 4.747328281 |
| OSM | Oncostatin M | Protein Coding | 46 | GC22M030262 | 4.745146751 |
| CXCR4 | C-X-C Motif Chemokine Receptor 4 | Protein Coding | 55 | GC02M136114 | 4.723081112 |
| ALOX5 | Arachidonate 5-Lipoxygenase | Protein Coding | 50 | GC10P045374 | 4.583646297 |
| AKT1 | AKT Serine/Threonine Kinase 1 | Protein Coding | 56 | GC14M104769 | 4.536396027 |
| ADRB2 | Adrenoceptor Beta 2 | Protein Coding | 52 | GC05P148825 | 4.534704685 |
| SRC | SRC Proto-Oncogene, Non-Receptor Tyrosine Kinase | Protein Coding | 54 | GC20P037344 | 4.5133605 |
| PIK3CG | Phosphatidylinositol-4,5-Bisphosphate 3-Kinase Catalytic Subunit Gamma | Protein Coding | 50 | GC07P106865 | 4.508783817 |
| PPARG | Peroxisome Proliferator Activated Receptor Gamma | Protein Coding | 55 | GC03P012287 | 4.494004726 |
| ADAM32 | ADAM Metallopeptidase Domain 32 | Protein Coding | 39 | GC08P039084 | 4.466450214 |
| SPP1 | Secreted Phosphoprotein 1 | Protein Coding | 49 | GC04P087975 | 4.459854603 |
| ICAM1 | Intercellular Adhesion Molecule 1 | Protein Coding | 53 | GC19P010638 | 4.413910866 |
| IFNG | Interferon Gamma | Protein Coding | 52 | GC12M068154 | 4.405203819 |
| MPO | Myeloperoxidase | Protein Coding | 55 | GC17M058269 | 4.397476196 |
| CTSK | Cathepsin K | Protein Coding | 53 | GC01M152412 | 4.357009888 |
| TP53 | Tumor Protein P53 | Protein Coding | 55 | GC17M007661 | 4.320524693 |
| ADI1 | Acireductone Dioxygenase 1 | Protein Coding | 43 | GC02M003501 | 4.307343006 |
| ITGB1 | Integrin Subunit Beta 1 | Protein Coding | 53 | GC10M033391 | 4.265536308 |
| CSN1S1 | Casein Alpha S1 | Protein Coding | 38 | GC04P069932 | 4.262128353 |
| CTNNB1 | Catenin Beta 1 | Protein Coding | 56 | GC03P041236 | 4.236029148 |
| IL18 | Interleukin 18 | Protein Coding | 47 | GC11M112143 | 4.228839397 |
| CCL11 | C-C Motif Chemokine Ligand 11 | Protein Coding | 48 | GC17P034285 | 4.180007458 |
| CD40LG | CD40 Ligand | Protein Coding | 52 | GC0XP136649 | 4.145516396 |
| ADAMDEC1 | ADAM Like Decysin 1 | Protein Coding | 40 | GC08P024384 | 4.14223814 |
| HBEGF | Heparin Binding EGF Like Growth Factor | Protein Coding | 45 | GC05M140332 | 4.126496315 |
| TLR2 | Toll Like Receptor 2 | Protein Coding | 54 | GC04P153684 | 4.124048233 |
| COL1A1 | Collagen Type I Alpha 1 Chain | Protein Coding | 52 | GC17M050248 | 4.117881298 |
| ADAM18 | ADAM Metallopeptidase Domain 18 | Protein Coding | 36 | GC08P039584 | 4.067027569 |
| HIF1A | Hypoxia Inducible Factor 1 Subunit Alpha | Protein Coding | 51 | GC14P061695 | 4.055150032 |
| TNC | Tenascin C | Protein Coding | 52 | GC09M115019 | 4.030341148 |
| EDN1 | Endothelin 1 | Protein Coding | 51 | GC06P012256 | 4.026006222 |
| HPX | Hemopexin | Protein Coding | 44 | GC11M006794 | 4.019511223 |
| TGFA | Transforming Growth Factor Alpha | Protein Coding | 49 | GC02M070447 | 3.99729228 |
| ADAMTSL1 | ADAMTS Like 1 | Protein Coding | 45 | GC09P017906 | 3.974218369 |
| ACE | Angiotensin I Converting Enzyme | Protein Coding | 54 | GC17P063477 | 3.959685802 |
| THBS2 | Thrombospondin 2 | Protein Coding | 48 | GC06M169215 | 3.950322866 |
| LRP2 | LDL Receptor Related Protein 2 | Protein Coding | 51 | GC02M169127 | 3.934791088 |
| IL4 | Interleukin 4 | Protein Coding | 49 | GC05P132673 | 3.921576977 |
| HGF | Hepatocyte Growth Factor | Protein Coding | 54 | GC07M081699 | 3.914263725 |
| THBS1 | Thrombospondin 1 | Protein Coding | 48 | GC15P039581 | 3.890021801 |
| TFPI2 | Tissue Factor Pathway Inhibitor 2 | Protein Coding | 45 | GC07M093885 | 3.87980628 |
| BRCA2 | BRCA2 DNA Repair Associated | Protein Coding | 50 | GC13P032315 | 3.878227234 |
| CCN2 | Cellular Communication Network Factor 2 | Protein Coding | 48 | GC06M131948 | 3.874992609 |
| CCL5 | C-C Motif Chemokine Ligand 5 | Protein Coding | 46 | GC17M035871 | 3.874076366 |
| SP1 | Sp1 Transcription Factor | Protein Coding | 49 | GC12P053380 | 3.841941357 |
| NGF | Nerve Growth Factor | Protein Coding | 54 | GC01M115285 | 3.821743011 |
| NOS2 | Nitric Oxide Synthase 2 | Protein Coding | 51 | GC17M027756 | 3.747982979 |
| S100A4 | S100 Calcium Binding Protein A4 | Protein Coding | 48 | GC01M153543 | 3.745687008 |
| CTSG | Cathepsin G | Protein Coding | 48 | GC14M024573 | 3.743005753 |
| ADAM7 | ADAM Metallopeptidase Domain 7 | Protein Coding | 38 | GC08P024440 | 3.74107933 |
| BCL2 | BCL2 Apoptosis Regulator | Protein Coding | 53 | GC18M063123 | 3.725467682 |
| NFKBIA | NFKB Inhibitor Alpha | Protein Coding | 52 | GC14M035401 | 3.710913181 |
| DCN | Decorin | Protein Coding | 50 | GC12M091140 | 3.695376873 |
| ITGB3 | Integrin Subunit Beta 3 | Protein Coding | 55 | GC17P058846 | 3.694393396 |
| PCOLCE | Procollagen C-Endopeptidase Enhancer | Protein Coding | 41 | GC07P100602 | 3.67933321 |
| PRSS2 | Serine Protease 2 | Protein Coding | 41 | GC07P148798 | 3.671057224 |
| LRP1 | LDL Receptor Related Protein 1 | Protein Coding | 50 | GC12P057128 | 3.66504097 |
| RHOA | Ras Homolog Family Member A | Protein Coding | 52 | GC03M049359 | 3.633411407 |
| CCL3 | C-C Motif Chemokine Ligand 3 | Protein Coding | 44 | GC17M036088 | 3.62869525 |
| ADAM5 | ADAM Metallopeptidase Domain 5 (Pseudogene) | Pseudogene | 22 | GC08P039291 | 3.61353755 |
| RELA | RELA Proto-Oncogene, NF-KB Subunit | Protein Coding | 54 | GC11M065653 | 3.605583668 |
| VCAM1 | Vascular Cell Adhesion Molecule 1 | Protein Coding | 48 | GC01P100719 | 3.588171721 |
| PLAT | Plasminogen Activator, Tissue Type | Protein Coding | 54 | GC08M042174 | 3.572025299 |
| TLR4 | Toll Like Receptor 4 | Protein Coding | 54 | GC09P117704 | 3.568025589 |
| TNFSF11 | TNF Superfamily Member 11 | Protein Coding | 54 | GC13P042562 | 3.532317877 |
| IDE | Insulin Degrading Enzyme | Protein Coding | 51 | GC10M092451 | 3.530981064 |
| CAV1 | Caveolin 1 | Protein Coding | 51 | GC07P116524 | 3.499522209 |
| ESR1 | Estrogen Receptor 1 | Protein Coding | 56 | GC06P151656 | 3.496103287 |
| CCL7 | C-C Motif Chemokine Ligand 7 | Protein Coding | 45 | GC17P034270 | 3.491931915 |
| ADAMTS10 | ADAM Metallopeptidase With Thrombospondin Type 1 Motif 10 | Protein Coding | 45 | GC19M008580 | 3.487799168 |
| MIF | Macrophage Migration Inhibitory Factor | Protein Coding | 50 | GC22P023894 | 3.485972881 |
| AREG | Amphiregulin | Protein Coding | 46 | GC04P074445 | 3.478336573 |
| MIR152 | MicroRNA 152 | RNA Gene | 23 | GC17M048037 | 3.446990967 |
| MRC2 | Mannose Receptor C Type 2 | Protein Coding | 43 | GC17P062627 | 3.422608614 |
| F2R | Coagulation Factor II Thrombin Receptor | Protein Coding | 50 | GC05P076716 | 3.415864468 |
| ANPEP | Alanyl Aminopeptidase, Membrane | Protein Coding | 53 | GC15M089784 | 3.413804531 |
| CSF1 | Colony Stimulating Factor 1 | Protein Coding | 47 | GC01P109911 | 3.396835804 |
| ADAM2 | ADAM Metallopeptidase Domain 2 | Protein Coding | 40 | GC08M039743 | 3.390522957 |
| IGF1R | Insulin Like Growth Factor 1 Receptor | Protein Coding | 57 | GC15P098648 | 3.376134872 |
| CD163 | CD163 Molecule | Protein Coding | 47 | GC12M007923 | 3.373744011 |
| MAPK10 | Mitogen-Activated Protein Kinase 10 | Protein Coding | 51 | GC04M085990 | 3.372867107 |
| A2M | Alpha-2-Macroglobulin | Protein Coding | 48 | GC12M009067 | 3.358028173 |
| CMA1 | Chymase 1 | Protein Coding | 47 | GC14M024506 | 3.357465267 |
| RUNX2 | RUNX Family Transcription Factor 2 | Protein Coding | 49 | GC06P088004 | 3.332108736 |
| ERBB2 | Erb-B2 Receptor Tyrosine Kinase 2 | Protein Coding | 58 | GC17P039687 | 3.319131851 |
| FASLG | Fas Ligand | Protein Coding | 50 | GC01P172628 | 3.306241989 |
| PAPPA2 | Pappalysin 2 | Protein Coding | 42 | GC01P176463 | 3.289784193 |
| HMOX1 | Heme Oxygenase 1 | Protein Coding | 55 | GC22P035380 | 3.288172245 |
| ADAMTS20 | ADAM Metallopeptidase With Thrombospondin Type 1 Motif 20 | Protein Coding | 39 | GC12M043353 | 3.285458565 |
| MIR21 | MicroRNA 21 | RNA Gene | 26 | GC17P059872 | 3.284866333 |
| COL2A1 | Collagen Type II Alpha 1 Chain | Protein Coding | 51 | GC12M047972 | 3.284769535 |
| COMP | Cartilage Oligomeric Matrix Protein | Protein Coding | 50 | GC19M018783 | 3.281956673 |
| STAT1 | Signal Transducer And Activator Of Transcription 1 | Protein Coding | 55 | GC02M190908 | 3.26880312 |
| SPARC | Secreted Protein Acidic And Cysteine Rich | Protein Coding | 52 | GC05M151661 | 3.259855747 |
| APOE | Apolipoprotein E | Protein Coding | 53 | GC19P069967 | 3.257021904 |
| IGF1 | Insulin Like Growth Factor 1 | Protein Coding | 50 | GC12M102395 | 3.249014378 |
| ADIPOQ | Adiponectin, C1Q And Collagen Domain Containing | Protein Coding | 48 | GC03P186842 | 3.248481274 |
| SMAD2 | SMAD Family Member 2 | Protein Coding | 53 | GC18M047809 | 3.235521793 |
| MIR148A | MicroRNA 148a | RNA Gene | 23 | GC07M025993 | 3.232454538 |
| CD40 | CD40 Molecule | Protein Coding | 52 | GC20P046118 | 3.232235909 |
| MIR126 | MicroRNA 126 | RNA Gene | 24 | GC09P136670 | 3.194005966 |
| CTSB | Cathepsin B | Protein Coding | 55 | GC08M011842 | 3.189107895 |
| MAP2K1 | Mitogen-Activated Protein Kinase Kinase 1 | Protein Coding | 55 | GC15P066386 | 3.177933931 |
| MET | MET Proto-Oncogene, Receptor Tyrosine Kinase | Protein Coding | 56 | GC07P116672 | 3.125898123 |
| TNFRSF11B | TNF Receptor Superfamily Member 11b | Protein Coding | 50 | GC08M118923 | 3.120138645 |
| PCSK5 | Proprotein Convertase Subtilisin/Kexin Type 5 | Protein Coding | 43 | GC09P075890 | 3.098402739 |
| NPEPPS | Aminopeptidase Puromycin Sensitive | Protein Coding | 44 | GC17P047522 | 3.094256163 |
| VDR | Vitamin D Receptor | Protein Coding | 52 | GC12M047841 | 3.091289759 |
| IL6R | Interleukin 6 Receptor | Protein Coding | 53 | GC01P154405 | 3.0768013 |
| CXCL10 | C-X-C Motif Chemokine Ligand 10 | Protein Coding | 47 | GC04M076021 | 3.052871704 |
| KISS1 | KiSS-1 Metastasis Suppressor | Protein Coding | 44 | GC01M204190 | 3.050887108 |
| AR | Androgen Receptor | Protein Coding | 54 | GC0XP067544 | 3.039886475 |
| PRKCA | Protein Kinase C Alpha | Protein Coding | 53 | GC17P066302 | 3.039567709 |
| PTEN | Phosphatase And Tensin Homolog | Protein Coding | 54 | GC10P093576 | 3.039475441 |
| HSPG2 | Heparan Sulfate Proteoglycan 2 | Protein Coding | 51 | GC01M021822 | 3.035137415 |
| ADAM1A | ADAM Metallopeptidase Domain 1A (Pseudogene) | Pseudogene | 17 | GC12P111899 | 3.015049696 |
| SDC2 | Syndecan 2 | Protein Coding | 48 | GC08P096498 | 2.996471882 |
| LOX | Lysyl Oxidase | Protein Coding | 50 | GC05M122063 | 2.987197876 |
| PLA2G7 | Phospholipase A2 Group VII | Protein Coding | 52 | GC06M046704 | 2.979578257 |
| SIRT1 | Sirtuin 1 | Protein Coding | 52 | GC10P067884 | 2.975409031 |
| PARP1 | Poly(ADP-Ribose) Polymerase 1 | Protein Coding | 53 | GC01M226360 | 2.947145462 |
| TCF20 | Transcription Factor 20 | Protein Coding | 41 | GC22M042160 | 2.943040848 |
| FOSL1 | FOS Like 1, AP-1 Transcription Factor Subunit | Protein Coding | 47 | GC11M097091 | 2.938238144 |
| TLR9 | Toll Like Receptor 9 | Protein Coding | 48 | GC03M052222 | 2.937383413 |
| APP | Amyloid Beta Precursor Protein | Protein Coding | 54 | GC21M025880 | 2.932906151 |
| DSPP | Dentin Sialophosphoprotein | Protein Coding | 41 | GC04P087608 | 2.927646637 |
| VEGFC | Vascular Endothelial Growth Factor C | Protein Coding | 50 | GC04M176683 | 2.903582335 |
| IGFBP3 | Insulin Like Growth Factor Binding Protein 3 | Protein Coding | 50 | GC07M045912 | 2.901169777 |
| IL4R | Interleukin 4 Receptor | Protein Coding | 51 | GC16P028153 | 2.900237799 |
| FBN1 | Fibrillin 1 | Protein Coding | 50 | GC15M048408 | 2.87574029 |
| CASP3 | Caspase 3 | Protein Coding | 52 | GC04M184627 | 2.86027813 |
| SERPINA1 | Serpin Family A Member 1 | Protein Coding | 51 | GC14M094376 | 2.856907606 |
| CASP8 | Caspase 8 | Protein Coding | 55 | GC02P201233 | 2.850172758 |
| MKI67 | Marker Of Proliferation Ki-67 | Protein Coding | 47 | GC10M128096 | 2.837196112 |
| TGFB2 | Transforming Growth Factor Beta 2 | Protein Coding | 54 | GC01P218345 | 2.835713863 |
| IGF2 | Insulin Like Growth Factor 2 | Protein Coding | 51 | GC11M003998 | 2.823159218 |
| KDR | Kinase Insert Domain Receptor | Protein Coding | 56 | GC04M055078 | 2.814684868 |
| HMGB1 | High Mobility Group Box 1 | Protein Coding | 49 | GC13M030456 | 2.803646088 |
| COL3A1 | Collagen Type III Alpha 1 Chain | Protein Coding | 50 | GC02P188974 | 2.802206993 |
| CEBPB | CCAAT Enhancer Binding Protein Beta | Protein Coding | 45 | GC20P050190 | 2.800367117 |
| TNFRSF1A | TNF Receptor Superfamily Member 1A | Protein Coding | 52 | GC12M006328 | 2.796746254 |
| CCR3 | C-C Motif Chemokine Receptor 3 | Protein Coding | 48 | GC03P047176 | 2.794194937 |
| MIR148B | MicroRNA 148b | RNA Gene | 23 | GC12P054337 | 2.784889698 |
| IL5 | Interleukin 5 | Protein Coding | 48 | GC05M132541 | 2.77425909 |
| PTGS1 | Prostaglandin-Endoperoxide Synthase 1 | Protein Coding | 49 | GC09P122370 | 2.768949032 |
| NOTCH1 | Notch Receptor 1 | Protein Coding | 55 | GC09M137632 | 2.752018213 |
| S100A12 | S100 Calcium Binding Protein A12 | Protein Coding | 41 | GC01M153373 | 2.750359058 |
| CCND1 | Cyclin D1 | Protein Coding | 55 | GC11P069641 | 2.742533684 |
| NME1 | NME/NM23 Nucleoside Diphosphate Kinase 1 | Protein Coding | 48 | GC17P059079 | 2.73489666 |
| EGR1 | Early Growth Response 1 | Protein Coding | 50 | GC05P138465 | 2.728484631 |
| PGR | Progesterone Receptor | Protein Coding | 51 | GC11M101030 | 2.728067398 |
| AGER | Advanced Glycosylation End-Product Specific Receptor | Protein Coding | 49 | GC06M032180 | 2.725686073 |
| SOD1 | Superoxide Dismutase 1 | Protein Coding | 56 | GC21P031659 | 2.725384474 |
| OLR1 | Oxidized Low Density Lipoprotein Receptor 1 | Protein Coding | 45 | GC12M021842 | 2.717785597 |
| FAS | Fas Cell Surface Death Receptor | Protein Coding | 53 | GC10P093591 | 2.716909647 |
| LAMC2 | Laminin Subunit Gamma 2 | Protein Coding | 50 | GC01P183186 | 2.705520391 |
| PRSS1 | Serine Protease 1 | Protein Coding | 47 | GC07P148780 | 2.703909636 |
| IFNB1 | Interferon Beta 1 | Protein Coding | 47 | GC09M021077 | 2.702925444 |
| CARD11 | Caspase Recruitment Domain Family Member 11 | Protein Coding | 49 | GC07M002906 | 2.699351549 |
| HNMT | Histamine N-Methyltransferase | Protein Coding | 48 | GC02P137964 | 2.699351549 |
| SCGB3A2 | Secretoglobin Family 3A Member 2 | Protein Coding | 40 | GC05P147870 | 2.699351549 |
| MUC7 | Mucin 7, Secreted | Protein Coding | 37 | GC04P070430 | 2.699351549 |
| ASRT3 | Asthma-Related Traits, Susceptibility To, 3 | Genetic Locus | 2 | GC02U901663 | 2.699351549 |
| ASRT4 | Asthma-Related Traits, Susceptibility To, 4 | Genetic Locus | 2 | GC01U902370 | 2.699351549 |
| ASRT6 | Asthma-Related Traits, Susceptibility To, 6 | Genetic Locus | 2 | GC17U901148 | 2.699351549 |
| ASRT8 | Asthma-Related Traits, Susceptibility To, 8 | Genetic Locus | 2 | GC09U901396 | 2.699351549 |
| CCL4 | C-C Motif Chemokine Ligand 4 | Protein Coding | 45 | GC17P036103 | 2.697391033 |
| SELE | Selectin E | Protein Coding | 48 | GC01M169722 | 2.686920404 |
| CSF3 | Colony Stimulating Factor 3 | Protein Coding | 45 | GC17P040015 | 2.68438077 |
| HLA-DRB1 | Major Histocompatibility Complex, Class II, DR Beta 1 | Protein Coding | 50 | GC06M069255 | 2.68274951 |
| SNAI2 | Snail Family Transcriptional Repressor 2 | Protein Coding | 48 | GC08M048917 | 2.680390596 |
| ITGA2 | Integrin Subunit Alpha 2 | Protein Coding | 49 | GC05P052989 | 2.676558018 |
| VTN | Vitronectin | Protein Coding | 47 | GC17M044982 | 2.668408632 |
| CCN1 | Cellular Communication Network Factor 1 | Protein Coding | 41 | GC01P085581 | 2.667702198 |
| PTGER4 | Prostaglandin E Receptor 4 | Protein Coding | 48 | GC05P040679 | 2.66734314 |
| LTA4H | Leukotriene A4 Hydrolase | Protein Coding | 49 | GC12M096000 | 2.66692996 |
| CHUK | Component Of Inhibitor Of Nuclear Factor Kappa B Kinase Complex | Protein Coding | 54 | GC10M100191 | 2.664269447 |
| S100A8 | S100 Calcium Binding Protein A8 | Protein Coding | 45 | GC01M153391 | 2.655337334 |
| IL1R1 | Interleukin 1 Receptor Type 1 | Protein Coding | 48 | GC02P102136 | 2.645222425 |
| SDC1 | Syndecan 1 | Protein Coding | 45 | GC02M020200 | 2.64405489 |
| IL15 | Interleukin 15 | Protein Coding | 46 | GC04P141636 | 2.638155699 |
| LIF | LIF Interleukin 6 Family Cytokine | Protein Coding | 45 | GC22M030240 | 2.63657093 |
| FLT1 | Fms Related Receptor Tyrosine Kinase 1 | Protein Coding | 54 | GC13M028300 | 2.628697395 |
| ITGA4 | Integrin Subunit Alpha 4 | Protein Coding | 50 | GC02P181456 | 2.627224445 |
| NR3C1 | Nuclear Receptor Subfamily 3 Group C Member 1 | Protein Coding | 51 | GC05M143277 | 2.626901865 |
| BMP2 | Bone Morphogenetic Protein 2 | Protein Coding | 51 | GC20P006767 | 2.602860451 |
| MME | Membrane Metalloendopeptidase | Protein Coding | 53 | GC03P155024 | 2.590419531 |
| RAC1 | Rac Family Small GTPase 1 | Protein Coding | 51 | GC07P006377 | 2.586125612 |
| SELP | Selectin P | Protein Coding | 48 | GC01M169558 | 2.565793037 |
| VWF | Von Willebrand Factor | Protein Coding | 51 | GC12M005917 | 2.559482574 |
| RLN2 | Relaxin 2 | Protein Coding | 40 | GC09M005402 | 2.550992727 |
| COL18A1 | Collagen Type XVIII Alpha 1 Chain | Protein Coding | 49 | GC21P045405 | 2.529232025 |
| CCR7 | C-C Motif Chemokine Receptor 7 | Protein Coding | 47 | GC17M049365 | 2.524915457 |
| TGM2 | Transglutaminase 2 | Protein Coding | 50 | GC20M038127 | 2.522520542 |
| CSF2 | Colony Stimulating Factor 2 | Protein Coding | 46 | GC05P132073 | 2.519522667 |
| IL11 | Interleukin 11 | Protein Coding | 43 | GC19M055364 | 2.518000126 |
| LGALS3 | Galectin 3 | Protein Coding | 48 | GC14P055124 | 2.503509521 |
| FGF7 | Fibroblast Growth Factor 7 | Protein Coding | 46 | GC15P049423 | 2.493188381 |
| CXCL1 | C-X-C Motif Chemokine Ligand 1 | Protein Coding | 45 | GC04P073869 | 2.489362717 |
| ANGPT2 | Angiopoietin 2 | Protein Coding | 50 | GC08M006499 | 2.488236666 |
| GP6 | Glycoprotein VI Platelet | Protein Coding | 48 | GC19M055013 | 2.473493576 |
| SMAD3 | SMAD Family Member 3 | Protein Coding | 54 | GC15P067063 | 2.463460445 |
| IL33 | Interleukin 33 | Protein Coding | 41 | GC09P007513 | 2.460860252 |
| VIM | Vimentin | Protein Coding | 53 | GC10P017227 | 2.455626965 |
| BGLAP | Bone Gamma-Carboxyglutamate Protein | Protein Coding | 44 | GC01P156242 | 2.454268932 |
| PTGER2 | Prostaglandin E Receptor 2 | Protein Coding | 51 | GC14P052314 | 2.447265625 |
| CDH2 | Cadherin 2 | Protein Coding | 55 | GC18M027950 | 2.445085287 |
| TGFBR1 | Transforming Growth Factor Beta Receptor 1 | Protein Coding | 56 | GC09P099104 | 2.430671692 |
| AGT | Angiotensinogen | Protein Coding | 52 | GC01M230702 | 2.414785147 |
| CAT | Catalase | Protein Coding | 54 | GC11P034460 | 2.405908108 |
| ESR2 | Estrogen Receptor 2 | Protein Coding | 51 | GC14M064084 | 2.398565531 |
| CDKN2A | Cyclin Dependent Kinase Inhibitor 2A | Protein Coding | 52 | GC09M021967 | 2.396027327 |
| F2RL1 | F2R Like Trypsin Receptor 1 | Protein Coding | 47 | GC05P076818 | 2.390169621 |
| TGFB3 | Transforming Growth Factor Beta 3 | Protein Coding | 52 | GC14M075958 | 2.389668226 |
| ALB | Albumin | Protein Coding | 52 | GC04P073397 | 2.371660709 |
| EPHB2 | EPH Receptor B2 | Protein Coding | 55 | GC01P022710 | 2.364565849 |
| USP6 | Ubiquitin Specific Peptidase 6 | Protein Coding | 41 | GC17P005116 | 2.359328985 |
| F3 | Coagulation Factor III, Tissue Factor | Protein Coding | 48 | GC01M094599 | 2.353899956 |
| FCER2 | Fc Epsilon Receptor II | Protein Coding | 46 | GC19M007689 | 2.352490664 |
| PTK2B | Protein Tyrosine Kinase 2 Beta | Protein Coding | 51 | GC08P027311 | 2.349984169 |
| SMAD4 | SMAD Family Member 4 | Protein Coding | 54 | GC18P051028 | 2.347314596 |
| IGFBP7 | Insulin Like Growth Factor Binding Protein 7 | Protein Coding | 49 | GC04M057030 | 2.336513281 |
| S100A9 | S100 Calcium Binding Protein A9 | Protein Coding | 46 | GC01P153357 | 2.328769207 |
| IL17RA | Interleukin 17 Receptor A | Protein Coding | 48 | GC22P039613 | 2.328083754 |
| ITGA5 | Integrin Subunit Alpha 5 | Protein Coding | 52 | GC12M054977 | 2.324602127 |
| F2 | Coagulation Factor II, Thrombin | Protein Coding | 52 | GC11P046720 | 2.322188616 |
| MUC5AC | Mucin 5AC, Oligomeric Mucus/Gel-Forming | Protein Coding | 43 | GC11P001988 | 2.301960707 |
| FPR2 | Formyl Peptide Receptor 2 | Protein Coding | 48 | GC19P051752 | 2.301059723 |
| TWIST1 | Twist Family BHLH Transcription Factor 1 | Protein Coding | 47 | GC07M019020 | 2.299676657 |
| SYN3 | Synapsin III | Protein Coding | 43 | GC22M059317 | 2.298606634 |
| IL1RAPL2 | Interleukin 1 Receptor Accessory Protein Like 2 | Protein Coding | 42 | GC0XP104566 | 2.294146538 |
| IL2 | Interleukin 2 | Protein Coding | 51 | GC04M122451 | 2.290128231 |
| CHI3L1 | Chitinase 3 Like 1 | Protein Coding | 47 | GC01M203148 | 2.288086653 |
| CTSV | Cathepsin V | Protein Coding | 45 | GC09M097029 | 2.286957741 |
| CX3CR1 | C-X3-C Motif Chemokine Receptor 1 | Protein Coding | 45 | GC03M039279 | 2.276212692 |
| JAK1 | Janus Kinase 1 | Protein Coding | 56 | GC01M064833 | 2.270537376 |
| SERPINA3 | Serpin Family A Member 3 | Protein Coding | 45 | GC14P094612 | 2.270421267 |
| SERPINB2 | Serpin Family B Member 2 | Protein Coding | 45 | GC18P063871 | 2.265687943 |
| IGFBP1 | Insulin Like Growth Factor Binding Protein 1 | Protein Coding | 46 | GC07P047917 | 2.264184475 |
| IL17F | Interleukin 17F | Protein Coding | 48 | GC06M069573 | 2.254647255 |
| AMZ1 | Archaelysin Family Metallopeptidase 1 | Protein Coding | 36 | GC07P002679 | 2.2488873 |
| ITGB2 | Integrin Subunit Beta 2 | Protein Coding | 54 | GC21M044885 | 2.248009443 |
| DDR2 | Discoidin Domain Receptor Tyrosine Kinase 2 | Protein Coding | 55 | GC01P162631 | 2.24784565 |
| SELL | Selectin L | Protein Coding | 45 | GC01M169690 | 2.24744606 |
| PPARA | Peroxisome Proliferator Activated Receptor Alpha | Protein Coding | 49 | GC22P046150 | 2.246338606 |
| BAX | BCL2 Associated X, Apoptosis Regulator | Protein Coding | 52 | GC19P048954 | 2.245942831 |
| IFNA1 | Interferon Alpha 1 | Protein Coding | 43 | GC09P021601 | 2.242621422 |
| AGTR1 | Angiotensin II Receptor Type 1 | Protein Coding | 54 | GC03P148697 | 2.24224782 |
| COL1A2 | Collagen Type I Alpha 2 Chain | Protein Coding | 50 | GC07P094394 | 2.239206314 |
| FGF1 | Fibroblast Growth Factor 1 | Protein Coding | 50 | GC05M142555 | 2.230458021 |
| CXCR2 | C-X-C Motif Chemokine Receptor 2 | Protein Coding | 52 | GC02P218125 | 2.219028234 |
| MTHFR | Methylenetetrahydrofolate Reductase | Protein Coding | 50 | GC01M011785 | 2.215386391 |
| BIRC5 | Baculoviral IAP Repeat Containing 5 | Protein Coding | 48 | GC17P078214 | 2.213386059 |
| LGALS1 | Galectin 1 | Protein Coding | 45 | GC22P037675 | 2.212993622 |
| GRB2 | Growth Factor Receptor Bound Protein 2 | Protein Coding | 48 | GC17M075318 | 2.204838276 |
| CST3 | Cystatin C | Protein Coding | 47 | GC20M023675 | 2.203976631 |
| FOXM1 | Forkhead Box M1 | Protein Coding | 46 | GC12M002857 | 2.197218418 |
| WNT5A | Wnt Family Member 5A | Protein Coding | 52 | GC03M055465 | 2.193066597 |
| POSTN | Periostin | Protein Coding | 46 | GC13M037562 | 2.190247297 |
| BCL2L1 | BCL2 Like 1 | Protein Coding | 50 | GC20M031664 | 2.18733263 |
| IL7 | Interleukin 7 | Protein Coding | 47 | GC08M078689 | 2.18342185 |
| PIK3CA | Phosphatidylinositol-4,5-Bisphosphate 3-Kinase Catalytic Subunit Alpha | Protein Coding | 55 | GC03P179148 | 2.182323933 |
| ANXA5 | Annexin A5 | Protein Coding | 48 | GC04M121667 | 2.178713799 |
| AHR | Aryl Hydrocarbon Receptor | Protein Coding | 50 | GC07P016916 | 2.172897339 |
| ADAM21P1 | ADAM Metallopeptidase Domain 21 Pseudogene 1 | Pseudogene | 13 | GC14M070245 | 2.171657085 |
| BIRC3 | Baculoviral IAP Repeat Containing 3 | Protein Coding | 48 | GC11P102317 | 2.16622448 |
| IL1R2 | Interleukin 1 Receptor Type 2 | Protein Coding | 49 | GC02P101991 | 2.15322113 |
| DMP1 | Dentin Matrix Acidic Phosphoprotein 1 | Protein Coding | 47 | GC04P087650 | 2.151714563 |
| BIRC2 | Baculoviral IAP Repeat Containing 2 | Protein Coding | 48 | GC11P102347 | 2.151548386 |
| IBSP | Integrin Binding Sialoprotein | Protein Coding | 43 | GC04P087799 | 2.150064468 |
| JAK2 | Janus Kinase 2 | Protein Coding | 55 | GC09P004985 | 2.144734144 |
| ADAM20P1 | ADAM Metallopeptidase Domain 20 Pseudogene 1 | Pseudogene | 13 | GC14M070468 | 2.144469738 |
| ADAM24P | ADAM Metallopeptidase Domain 24, Pseudogene | Pseudogene | 9 | GC08P017469 | 2.144469738 |
| TMBIM1 | Transmembrane BAX Inhibitor Motif Containing 1 | Protein Coding | 38 | GC02M218274 | 2.144420385 |
| VCAN | Versican | Protein Coding | 50 | GC05P083471 | 2.136028767 |
| MYC | MYC Proto-Oncogene, BHLH Transcription Factor | Protein Coding | 54 | GC08P127735 | 2.132927656 |
| PON1 | Paraoxonase 1 | Protein Coding | 50 | GC07M095297 | 2.131173372 |
| TFAP2A | Transcription Factor AP-2 Alpha | Protein Coding | 49 | GC06M010393 | 2.129341841 |
| ARFIP2 | ADP Ribosylation Factor Interacting Protein 2 | Protein Coding | 43 | GC11M006796 | 2.128428936 |
| ACP1 | Acid Phosphatase 1 | Protein Coding | 45 | GC02P000364 | 2.127562523 |
| WTAPP1 | Wilms Tumor 1 Associated Protein Pseudogene 1 | Pseudogene | 12 | GC11P102746 | 2.125497341 |
| FOXO3 | Forkhead Box O3 | Protein Coding | 48 | GC06P108559 | 2.12484026 |
| CLDN4 | Claudin 4 | Protein Coding | 42 | GC07P073799 | 2.12484026 |
| CDKN1A | Cyclin Dependent Kinase Inhibitor 1A | Protein Coding | 51 | GC06P087904 | 2.111932755 |
| CTSL | Cathepsin L | Protein Coding | 50 | GC09P087725 | 2.111932755 |
| MSTN | Myostatin | Protein Coding | 50 | GC02M190055 | 2.109326601 |
| SNAI1 | Snail Family Transcriptional Repressor 1 | Protein Coding | 48 | GC20P049982 | 2.108858585 |
| TRPV1 | Transient Receptor Potential Cation Channel Subfamily V Member 1 | Protein Coding | 50 | GC17M003565 | 2.104701281 |
| GP1BA | Glycoprotein Ib Platelet Subunit Alpha | Protein Coding | 50 | GC17P004932 | 2.10360837 |
| TLR3 | Toll Like Receptor 3 | Protein Coding | 54 | GC04P186059 | 2.10225606 |
| TGIF1 | TGFB Induced Factor Homeobox 1 | Protein Coding | 48 | GC18P003411 | 2.101708412 |
| GLB1 | Galactosidase Beta 1 | Protein Coding | 51 | GC03M032963 | 2.097167492 |
| CCR5 | C-C Motif Chemokine Receptor 5 | Protein Coding | 51 | GC03P047182 | 2.088978529 |
| TAC1 | Tachykinin Precursor 1 | Protein Coding | 44 | GC07P097731 | 2.087913513 |
| BDNF | Brain Derived Neurotrophic Factor | Protein Coding | 52 | GC11M027654 | 2.08433032 |
| CXCR3 | C-X-C Motif Chemokine Receptor 3 | Protein Coding | 46 | GC0XM071615 | 2.082234859 |
| ITGAM | Integrin Subunit Alpha M | Protein Coding | 50 | GC16P041958 | 2.081221104 |
| ITGA1 | Integrin Subunit Alpha 1 | Protein Coding | 47 | GC05P052788 | 2.078667641 |
| ITGB6 | Integrin Subunit Beta 6 | Protein Coding | 51 | GC02M160099 | 2.075935841 |
| MICB | MHC Class I Polypeptide-Related Sequence B | Protein Coding | 43 | GC06P087725 | 2.075088978 |
| NOS3 | Nitric Oxide Synthase 3 | Protein Coding | 52 | GC07P150990 | 2.071884632 |
| EP300 | E1A Binding Protein P300 | Protein Coding | 54 | GC22P041114 | 2.069315195 |
| IL23A | Interleukin 23 Subunit Alpha | Protein Coding | 44 | GC12P057394 | 2.068515539 |
| IGHE | Immunoglobulin Heavy Constant Epsilon | Protein Coding | 32 | GC14M113013 | 2.065315723 |
| IL1RN | Interleukin 1 Receptor Antagonist | Protein Coding | 52 | GC02P123138 | 2.059840918 |
| CTSD | Cathepsin D | Protein Coding | 55 | GC11M001752 | 2.053142786 |
| CXCR1 | C-X-C Motif Chemokine Receptor 1 | Protein Coding | 48 | GC02M218162 | 2.048968792 |
| RAF1 | Raf-1 Proto-Oncogene, Serine/Threonine Kinase | Protein Coding | 57 | GC03M012583 | 2.046955824 |
| HPSE | Heparanase | Protein Coding | 47 | GC04M083292 | 2.046955824 |
| SELPLG | Selectin P Ligand | Protein Coding | 47 | GC12M108621 | 2.046765566 |
| NCOA3 | Nuclear Receptor Coactivator 3 | Protein Coding | 50 | GC20P047501 | 2.044082165 |
| CREB1 | CAMP Responsive Element Binding Protein 1 | Protein Coding | 51 | GC02P207529 | 2.038446903 |
| SFN | Stratifin | Protein Coding | 48 | GC01P028244 | 2.034576893 |
| ATF3 | Activating Transcription Factor 3 | Protein Coding | 47 | GC01P212565 | 2.034576893 |
| CCL8 | C-C Motif Chemokine Ligand 8 | Protein Coding | 44 | GC17P034319 | 2.034576893 |
| LTA | Lymphotoxin Alpha | Protein Coding | 47 | GC06P087729 | 2.031401634 |
| BMP4 | Bone Morphogenetic Protein 4 | Protein Coding | 51 | GC14M053949 | 2.025045156 |
| NPPB | Natriuretic Peptide B | Protein Coding | 46 | GC01M011858 | 2.009186268 |
| NID1 | Nidogen 1 | Protein Coding | 46 | GC01M235975 | 2.008022785 |
| ANGPT1 | Angiopoietin 1 | Protein Coding | 49 | GC08M107246 | 2.007107496 |
| PLA2G2A | Phospholipase A2 Group IIA | Protein Coding | 50 | GC01M019975 | 2.004225492 |
| NFE2L2 | NFE2 Like BZIP Transcription Factor 2 | Protein Coding | 53 | GC02M177227 | 1.999164939 |
| GNRH1 | Gonadotropin Releasing Hormone 1 | Protein Coding | 45 | GC08M025419 | 1.988889933 |
| TLL1 | Tolloid Like 1 | Protein Coding | 47 | GC04P165873 | 1.983826518 |
| XDH | Xanthine Dehydrogenase | Protein Coding | 50 | GC02M031334 | 1.978136778 |
| ITGA3 | Integrin Subunit Alpha 3 | Protein Coding | 51 | GC17P050055 | 1.978134632 |
| CD63 | CD63 Molecule | Protein Coding | 45 | GC12M055725 | 1.975127935 |
| JUNB | JunB Proto-Oncogene, AP-1 Transcription Factor Subunit | Protein Coding | 44 | GC19P012791 | 1.968720675 |
| PTGER3 | Prostaglandin E Receptor 3 | Protein Coding | 49 | GC01M070852 | 1.968340039 |
| CTTN | Cortactin | Protein Coding | 45 | GC11P070398 | 1.96760726 |
| CD151 | CD151 Molecule (Raph Blood Group) | Protein Coding | 47 | GC11P001966 | 1.958395481 |
| PF4 | Platelet Factor 4 | Protein Coding | 43 | GC04M073980 | 1.95290041 |
| CCL13 | C-C Motif Chemokine Ligand 13 | Protein Coding | 41 | GC17P034356 | 1.951372385 |
| EFEMP1 | EGF Containing Fibulin Extracellular Matrix Protein 1 | Protein Coding | 47 | GC02M055865 | 1.950919151 |
| CDH5 | Cadherin 5 | Protein Coding | 49 | GC16P066366 | 1.949911118 |
| MIR145 | MicroRNA 145 | RNA Gene | 25 | GC05P149430 | 1.941975832 |
| TNFRSF1B | TNF Receptor Superfamily Member 1B | Protein Coding | 52 | GC01P012272 | 1.938125849 |
| CLDN5 | Claudin 5 | Protein Coding | 42 | GC22M019523 | 1.937211037 |
| MBL2 | Mannose Binding Lectin 2 | Protein Coding | 49 | GC10M052760 | 1.933238745 |
| MTOR | Mechanistic Target Of Rapamycin Kinase | Protein Coding | 56 | GC01M011106 | 1.92814064 |
| PIK3R1 | Phosphoinositide-3-Kinase Regulatory Subunit 1 | Protein Coding | 53 | GC05P068215 | 1.928058982 |
| RETN | Resistin | Protein Coding | 45 | GC19P007669 | 1.927614927 |
| TLL2 | Tolloid Like 2 | Protein Coding | 43 | GC10M096364 | 1.927220106 |
| CCR6 | C-C Motif Chemokine Receptor 6 | Protein Coding | 46 | GC06P167111 | 1.920036197 |
| MEP1B | Meprin A Subunit Beta | Protein Coding | 44 | GC18P032185 | 1.918608427 |
| KRAS | KRAS Proto-Oncogene, GTPase | Protein Coding | 54 | GC12M025204 | 1.915542364 |
| NGFR | Nerve Growth Factor Receptor | Protein Coding | 50 | GC17P049495 | 1.915127993 |
| TCF7L2 | Transcription Factor 7 Like 2 | Protein Coding | 49 | GC10P112950 | 1.911908627 |
| SOCS3 | Suppressor Of Cytokine Signaling 3 | Protein Coding | 45 | GC17M078356 | 1.906726599 |
| PDE4A | Phosphodiesterase 4A | Protein Coding | 47 | GC19P010416 | 1.906155229 |
| MYD88 | MYD88 Innate Immune Signal Transduction Adaptor | Protein Coding | 51 | GC03P038144 | 1.89437294 |
| CLDN3 | Claudin 3 | Protein Coding | 43 | GC07M073768 | 1.893028378 |
| MIR222 | MicroRNA 222 | RNA Gene | 22 | GC0XM045747 | 1.892569304 |
| CX3CL1 | C-X3-C Motif Chemokine Ligand 1 | Protein Coding | 47 | GC16P057372 | 1.891003013 |
| CDC42 | Cell Division Cycle 42 | Protein Coding | 53 | GC01P022052 | 1.890725374 |
| CXCL5 | C-X-C Motif Chemokine Ligand 5 | Protein Coding | 43 | GC04M073995 | 1.89037776 |
| GSTM1 | Glutathione S-Transferase Mu 1 | Protein Coding | 44 | GC01P109687 | 1.888904333 |
| CHRM3 | Cholinergic Receptor Muscarinic 3 | Protein Coding | 51 | GC01P239386 | 1.888243556 |
| ROCK1 | Rho Associated Coiled-Coil Containing Protein Kinase 1 | Protein Coding | 52 | GC18M020946 | 1.886350155 |
| CDK4 | Cyclin Dependent Kinase 4 | Protein Coding | 56 | GC12M057752 | 1.882018209 |
| RTN4RL2 | Reticulon 4 Receptor Like 2 | Protein Coding | 38 | GC11P057460 | 1.880522609 |
| PLA2G4A | Phospholipase A2 Group IVA | Protein Coding | 51 | GC01P186798 | 1.87896812 |
| HTN3 | Histatin 3 | Protein Coding | 34 | GC04P070028 | 1.878205895 |
| TPSAB1 | Tryptase Alpha/Beta 1 | Protein Coding | 45 | GC16P001240 | 1.871994853 |
| IGFBP4 | Insulin Like Growth Factor Binding Protein 4 | Protein Coding | 47 | GC17P040443 | 1.861191392 |
| SH3PXD2A | SH3 And PX Domains 2A | Protein Coding | 42 | GC10M103594 | 1.855286002 |
| SOCS1 | Suppressor Of Cytokine Signaling 1 | Protein Coding | 46 | GC16M012363 | 1.854928851 |
| NQO1 | NAD(P)H Quinone Dehydrogenase 1 | Protein Coding | 50 | GC16M069706 | 1.854253769 |
| MTDH | Metadherin | Protein Coding | 43 | GC08P097644 | 1.851505756 |
| TACR1 | Tachykinin Receptor 1 | Protein Coding | 48 | GC02M075162 | 1.849794984 |
| PRKD1 | Protein Kinase D1 | Protein Coding | 52 | GC14M029576 | 1.849287152 |
| JAK3 | Janus Kinase 3 | Protein Coding | 54 | GC19M017824 | 1.848122954 |
| ETV5 | ETS Variant Transcription Factor 5 | Protein Coding | 43 | GC03M186046 | 1.847627878 |
| IL16 | Interleukin 16 | Protein Coding | 43 | GC15P081159 | 1.837313771 |
| HDAC1 | Histone Deacetylase 1 | Protein Coding | 52 | GC01P032292 | 1.836862922 |
| ELF3 | E74 Like ETS Transcription Factor 3 | Protein Coding | 41 | GC01P202007 | 1.836327791 |
| ADAMTSL2 | ADAMTS Like 2 | Protein Coding | 44 | GC09P134395 | 1.831386805 |
| KLK4 | Kallikrein Related Peptidase 4 | Protein Coding | 50 | GC19M068274 | 1.829932928 |
| ETV7 | ETS Variant Transcription Factor 7 | Protein Coding | 38 | GC06M069353 | 1.824488163 |
| KITLG | KIT Ligand | Protein Coding | 48 | GC12M088492 | 1.813977957 |
| ENG | Endoglin | Protein Coding | 51 | GC09M127815 | 1.813704729 |
| HDAC9 | Histone Deacetylase 9 | Protein Coding | 50 | GC07P018086 | 1.813254833 |
| GAPDH | Glyceraldehyde-3-Phosphate Dehydrogenase | Protein Coding | 52 | GC12P022355 | 1.813033223 |
| ODC1 | Ornithine Decarboxylase 1 | Protein Coding | 51 | GC02M010432 | 1.808834672 |
| MIR155 | MicroRNA 155 | RNA Gene | 23 | GC21P025573 | 1.80693841 |
| RELB | RELB Proto-Oncogene, NF-KB Subunit | Protein Coding | 46 | GC19P069972 | 1.804841876 |
| PPIA | Peptidylprolyl Isomerase A | Protein Coding | 49 | GC07P044807 | 1.804257989 |
| PRL | Prolactin | Protein Coding | 45 | GC06M022287 | 1.802615643 |
| PRKCD | Protein Kinase C Delta | Protein Coding | 55 | GC03P053156 | 1.800557971 |
| CD9 | CD9 Molecule | Protein Coding | 46 | GC12P022327 | 1.800557971 |
| CD14 | CD14 Molecule | Protein Coding | 50 | GC05M140631 | 1.800506353 |
| SAA1 | Serum Amyloid A1 | Protein Coding | 42 | GC11P018547 | 1.789513111 |
| SOD2 | Superoxide Dismutase 2 | Protein Coding | 51 | GC06M159669 | 1.787325382 |
| MUC1 | Mucin 1, Cell Surface Associated | Protein Coding | 51 | GC01M155185 | 1.784742832 |
| ADORA3 | Adenosine A3 Receptor | Protein Coding | 48 | GC01M111499 | 1.783512831 |
| POMC | Proopiomelanocortin | Protein Coding | 51 | GC02M025160 | 1.783130884 |
| BRAF | B-Raf Proto-Oncogene, Serine/Threonine Kinase | Protein Coding | 55 | GC07M140740 | 1.780186892 |
| IGFBP5 | Insulin Like Growth Factor Binding Protein 5 | Protein Coding | 45 | GC02M216672 | 1.779652119 |
| SLC24A3 | Solute Carrier Family 24 Member 3 | Protein Coding | 42 | GC20P019212 | 1.777654648 |
| HMGCR | 3-Hydroxy-3-Methylglutaryl-CoA Reductase | Protein Coding | 50 | GC05P075336 | 1.775600791 |
| CTLA4 | Cytotoxic T-Lymphocyte Associated Protein 4 | Protein Coding | 52 | GC02P203854 | 1.772830606 |
| C4A | Complement C4A (Rodgers Blood Group) | Protein Coding | 46 | GC06P087763 | 1.771436572 |
| HLA-DQB1 | Major Histocompatibility Complex, Class II, DQ Beta 1 | Protein Coding | 45 | GC06M069264 | 1.771436572 |
| BTC | Betacellulin | Protein Coding | 47 | GC04M074744 | 1.769200444 |
| NRG1 | Neuregulin 1 | Protein Coding | 51 | GC08P031639 | 1.768266082 |
| PTX3 | Pentraxin 3 | Protein Coding | 45 | GC03P157436 | 1.768266082 |
| GSK3B | Glycogen Synthase Kinase 3 Beta | Protein Coding | 52 | GC03M119821 | 1.768193007 |
| MIR146A | MicroRNA 146a | RNA Gene | 25 | GC05P160485 | 1.765141487 |
| TGFBR2 | Transforming Growth Factor Beta Receptor 2 | Protein Coding | 54 | GC03P030623 | 1.762398243 |
| MDM2 | MDM2 Proto-Oncogene | Protein Coding | 55 | GC12P068808 | 1.75833559 |
| ETS2 | ETS Proto-Oncogene 2, Transcription Factor | Protein Coding | 46 | GC21P038805 | 1.756959677 |
| PCNA | Proliferating Cell Nuclear Antigen | Protein Coding | 53 | GC20M005114 | 1.753149867 |
| BCAR1 | BCAR1 Scaffold Protein, Cas Family Member | Protein Coding | 46 | GC16M075228 | 1.749814749 |
| RCE1 | Ras Converting CAAX Endopeptidase 1 | Protein Coding | 41 | GC11P066842 | 1.748475909 |
| OCLN | Occludin | Protein Coding | 47 | GC05P069492 | 1.746245623 |
| PLGRKT | Plasminogen Receptor With A C-Terminal Lysine | Protein Coding | 36 | GC09M005357 | 1.741658688 |
| IKBKB | Inhibitor Of Nuclear Factor Kappa B Kinase Subunit Beta | Protein Coding | 56 | GC08P042271 | 1.740006447 |
| APOA1 | Apolipoprotein A1 | Protein Coding | 52 | GC11M116835 | 1.738389969 |
| ADRB1 | Adrenoceptor Beta 1 | Protein Coding | 50 | GC10P114044 | 1.735560656 |
| ANXA2 | Annexin A2 | Protein Coding | 51 | GC15M060347 | 1.733630657 |
| CXCL16 | C-X-C Motif Chemokine Ligand 16 | Protein Coding | 42 | GC17M004733 | 1.731862664 |
| GDF11 | Growth Differentiation Factor 11 | Protein Coding | 45 | GC12P055743 | 1.729602098 |
| IL3 | Interleukin 3 | Protein Coding | 47 | GC05P132060 | 1.726677656 |
| CDKN1B | Cyclin Dependent Kinase Inhibitor 1B | Protein Coding | 50 | GC12P022586 | 1.72581625 |
| CD4 | CD4 Molecule | Protein Coding | 52 | GC12P006786 | 1.722161293 |
| NAMPT | Nicotinamide Phosphoribosyltransferase | Protein Coding | 51 | GC07M106248 | 1.721366048 |
| MIR34A | MicroRNA 34a | RNA Gene | 25 | GC01M009151 | 1.717092752 |
| IL12B | Interleukin 12B | Protein Coding | 50 | GC05M159314 | 1.712001681 |
| MIR29B1 | MicroRNA 29b-1 | RNA Gene | 23 | GC07M130877 | 1.711599827 |
| ADAM3B | ADAM Metallopeptidase Domain 3B (Pseudogene) | Pseudogene | 12 | GC16M049517 | 1.711172819 |
| MAP2K3 | Mitogen-Activated Protein Kinase Kinase 3 | Protein Coding | 51 | GC17P057660 | 1.710745692 |
| S100A14 | S100 Calcium Binding Protein A14 | Protein Coding | 40 | GC01M153614 | 1.710068107 |
| TEK | TEK Receptor Tyrosine Kinase | Protein Coding | 54 | GC09P027109 | 1.709169269 |
| NR1H2 | Nuclear Receptor Subfamily 1 Group H Member 2 | Protein Coding | 49 | GC19P050329 | 1.706683397 |
| PCSK6 | Proprotein Convertase Subtilisin/Kexin Type 6 | Protein Coding | 44 | GC15M119445 | 1.706683397 |
| TLR1 | Toll Like Receptor 1 | Protein Coding | 50 | GC04M038793 | 1.701031327 |
| VIP | Vasoactive Intestinal Peptide | Protein Coding | 47 | GC06P152750 | 1.701031327 |
| ILK | Integrin Linked Kinase | Protein Coding | 48 | GC11P006604 | 1.6963377 |
| IRF1 | Interferon Regulatory Factor 1 | Protein Coding | 49 | GC05M132440 | 1.695212483 |
| ADRB3 | Adrenoceptor Beta 3 | Protein Coding | 49 | GC08M037962 | 1.693139911 |
| DPP4 | Dipeptidyl Peptidase 4 | Protein Coding | 53 | GC02M161992 | 1.692636132 |
| TERT | Telomerase Reverse Transcriptase | Protein Coding | 53 | GC05M001253 | 1.690848827 |
| HSP90AA1 | Heat Shock Protein 90 Alpha Family Class A Member 1 | Protein Coding | 52 | GC14M102080 | 1.689332247 |
| IL13RA2 | Interleukin 13 Receptor Subunit Alpha 2 | Protein Coding | 42 | GC0XM115003 | 1.689332247 |
| FLG | Filaggrin | Protein Coding | 43 | GC01M152304 | 1.689157248 |
| LTF | Lactotransferrin | Protein Coding | 48 | GC03M046435 | 1.687139511 |
| IGFBP2 | Insulin Like Growth Factor Binding Protein 2 | Protein Coding | 47 | GC02P216632 | 1.687139511 |
| CPQ | Carboxypeptidase Q | Protein Coding | 40 | GC08P096645 | 1.686632872 |
| LPL | Lipoprotein Lipase | Protein Coding | 52 | GC08P019901 | 1.68651402 |
| GDF15 | Growth Differentiation Factor 15 | Protein Coding | 45 | GC19P069333 | 1.68651402 |
| BMP7 | Bone Morphogenetic Protein 7 | Protein Coding | 48 | GC20M057168 | 1.685510874 |
| CTSH | Cathepsin H | Protein Coding | 49 | GC15M118976 | 1.683662176 |
| CYP1A1 | Cytochrome P450 Family 1 Subfamily A Member 1 | Protein Coding | 50 | GC15M074719 | 1.683104634 |
| TNFRSF11A | TNF Receptor Superfamily Member 11a | Protein Coding | 52 | GC18P062325 | 1.680904269 |
| IL25 | Interleukin 25 | Protein Coding | 43 | GC14P033600 | 1.680324197 |
| LRPAP1 | LDL Receptor Related Protein Associated Protein 1 | Protein Coding | 46 | GC04M003508 | 1.676245332 |
| THSD4 | Thrombospondin Type 1 Domain Containing 4 | Protein Coding | 40 | GC15P071096 | 1.675741673 |
| ADAM3A | ADAM Metallopeptidase Domain 3A (Pseudogene) | Pseudogene | 15 | GC08M039427 | 1.675741673 |
| LPAR3 | Lysophosphatidic Acid Receptor 3 | Protein Coding | 46 | GC01M084811 | 1.674528837 |
| MATN1 | Matrilin 1 | Protein Coding | 44 | GC01M030711 | 1.674528837 |
| PDGFD | Platelet Derived Growth Factor D | Protein Coding | 46 | GC11M103907 | 1.67089951 |
| KISS1R | KISS1 Receptor | Protein Coding | 51 | GC19P000917 | 1.668986082 |
| PTH | Parathyroid Hormone | Protein Coding | 48 | GC11M013492 | 1.66842854 |
| TJP1 | Tight Junction Protein 1 | Protein Coding | 46 | GC15M029699 | 1.666355371 |
| SNCG | Synuclein Gamma | Protein Coding | 44 | GC10P093503 | 1.665118933 |
| GHRL | Ghrelin And Obestatin Prepropeptide | Protein Coding | 45 | GC03M010285 | 1.663669705 |
| MEPE | Matrix Extracellular Phosphoglycoprotein | Protein Coding | 39 | GC04P087821 | 1.663657904 |
| KLKB1 | Kallikrein B1 | Protein Coding | 50 | GC04P186208 | 1.663244486 |
| SCGB1A1 | Secretoglobin Family 1A Member 1 | Protein Coding | 43 | GC11P062405 | 1.662838101 |
| RPSA | Ribosomal Protein SA | Protein Coding | 48 | GC03P039406 | 1.662597179 |
| PPBP | Pro-Platelet Basic Protein | Protein Coding | 45 | GC04M073986 | 1.660775304 |
| TXN | Thioredoxin | Protein Coding | 48 | GC09M110243 | 1.660373449 |
| FSCN1 | Fascin Actin-Bundling Protein 1 | Protein Coding | 47 | GC07P005592 | 1.653885603 |
| MGAT5 | Alpha-1,6-Mannosylglycoprotein 6-Beta-N-Acetylglucosaminyltransferase | Protein Coding | 44 | GC02P134119 | 1.652164459 |
| ADORA2A | Adenosine A2a Receptor | Protein Coding | 51 | GC22P024417 | 1.645794868 |
| HAVCR1 | Hepatitis A Virus Cellular Receptor 1 | Protein Coding | 45 | GC05M157028 | 1.645794868 |
| PAK1 | P21 (RAC1) Activated Kinase 1 | Protein Coding | 52 | GC11M097499 | 1.645517826 |
| PKD1 | Polycystin 1, Transient Receptor Potential Channel Interacting | Protein Coding | 48 | GC16M007548 | 1.645517826 |
| NRP1 | Neuropilin 1 | Protein Coding | 50 | GC10M033177 | 1.641339898 |
| EPHX1 | Epoxide Hydrolase 1 | Protein Coding | 48 | GC01P225810 | 1.635112762 |
| PTP4A3 | Protein Tyrosine Phosphatase 4A3 | Protein Coding | 41 | GC08P141391 | 1.633713007 |
| IGF2R | Insulin Like Growth Factor 2 Receptor | Protein Coding | 48 | GC06P159969 | 1.631000638 |
| HRH1 | Histamine Receptor H1 | Protein Coding | 48 | GC03P012712 | 1.627794504 |
| EPRS1 | Glutamyl-Prolyl-TRNA Synthetase 1 | Protein Coding | 45 | GC01M219969 | 1.627794504 |
| SMAD7 | SMAD Family Member 7 | Protein Coding | 48 | GC18M048919 | 1.626408815 |
| PRTN3 | Proteinase 3 | Protein Coding | 48 | GC19P000840 | 1.626083374 |
| BATF3 | Basic Leucine Zipper ATF-Like Transcription Factor 3 | Protein Coding | 39 | GC01M212686 | 1.623209238 |
| KRT19 | Keratin 19 | Protein Coding | 46 | GC17M041523 | 1.620154381 |
| LGALS7 | Galectin 7 | Protein Coding | 38 | GC19M038770 | 1.620154381 |
| STAT6 | Signal Transducer And Activator Of Transcription 6 | Protein Coding | 53 | GC12M057095 | 1.619486928 |
| NR1H3 | Nuclear Receptor Subfamily 1 Group H Member 3 | Protein Coding | 48 | GC11P047248 | 1.619358301 |
| TRAF6 | TNF Receptor Associated Factor 6 | Protein Coding | 48 | GC11M036467 | 1.619358301 |
| EZH2 | Enhancer Of Zeste 2 Polycomb Repressive Complex 2 Subunit | Protein Coding | 56 | GC07M148807 | 1.617529035 |
| HSPB1 | Heat Shock Protein Family B (Small) Member 1 | Protein Coding | 54 | GC07P076302 | 1.617529035 |
| FOSB | FosB Proto-Oncogene, AP-1 Transcription Factor Subunit | Protein Coding | 45 | GC19P045467 | 1.612712383 |
| VEGFD | Vascular Endothelial Growth Factor D | Protein Coding | 42 | GC0XM015345 | 1.612613559 |
| CYP19A1 | Cytochrome P450 Family 19 Subfamily A Member 1 | Protein Coding | 51 | GC15M051208 | 1.607140541 |
| MAP2K4 | Mitogen-Activated Protein Kinase Kinase 4 | Protein Coding | 48 | GC17P012020 | 1.607140541 |
| HAS2 | Hyaluronan Synthase 2 | Protein Coding | 43 | GC08M121594 | 1.604326487 |
| IDO1 | Indoleamine 2,3-Dioxygenase 1 | Protein Coding | 47 | GC08P039891 | 1.603461742 |
| RHOD | Ras Homolog Family Member D | Protein Coding | 40 | GC11P070573 | 1.603411317 |
| FCGR3A | Fc Gamma Receptor IIIa | Protein Coding | 49 | GC01M161541 | 1.603060007 |
| CCR8 | C-C Motif Chemokine Receptor 8 | Protein Coding | 44 | GC03P039933 | 1.602699041 |
| PSMB9 | Proteasome 20S Subunit Beta 9 | Protein Coding | 48 | GC06P087772 | 1.598177075 |
| ELK1 | ETS Transcription Factor ELK1 | Protein Coding | 45 | GC0XM047635 | 1.593899608 |
| HSP90AA2P | Heat Shock Protein 90 Alpha Family Class A Member 2, Pseudogene | Pseudogene | 20 | GC11M027888 | 1.593899608 |
| MAZ | MYC Associated Zinc Finger Protein | Protein Coding | 42 | GC16P029806 | 1.593402147 |
| IL17RC | Interleukin 17 Receptor C | Protein Coding | 45 | GC03P009917 | 1.59268558 |
| EFNB2 | Ephrin B2 | Protein Coding | 47 | GC13M106489 | 1.591218472 |
| AMZ2 | Archaelysin Family Metallopeptidase 2 | Protein Coding | 36 | GC17P069137 | 1.590203404 |
| ADAM6 | ADAM Metallopeptidase Domain 6 (Pseudogene) | Pseudogene | 13 | GC14M105969 | 1.590203404 |
| LYN | LYN Proto-Oncogene, Src Family Tyrosine Kinase | Protein Coding | 51 | GC08P055879 | 1.584443331 |
| CHRD | Chordin | Protein Coding | 44 | GC03P184380 | 1.582885146 |
| ETV1 | ETS Variant Transcription Factor 1 | Protein Coding | 48 | GC07M013891 | 1.581533432 |
| CCL20 | C-C Motif Chemokine Ligand 20 | Protein Coding | 44 | GC02P227846 | 1.581041336 |
| TSPAN32 | Tetraspanin 32 | Protein Coding | 37 | GC11P002302 | 1.578273892 |
| CCR1 | C-C Motif Chemokine Receptor 1 | Protein Coding | 48 | GC03M046218 | 1.578246951 |
| GNRHR | Gonadotropin Releasing Hormone Receptor | Protein Coding | 52 | GC04M067737 | 1.570783973 |
| TP63 | Tumor Protein P63 | Protein Coding | 50 | GC03P189598 | 1.570668697 |
| EPHA2 | EPH Receptor A2 | Protein Coding | 56 | GC01M016124 | 1.568050742 |
| CD81 | CD81 Molecule | Protein Coding | 49 | GC11P002548 | 1.566141009 |
| IRAK1 | Interleukin 1 Receptor Associated Kinase 1 | Protein Coding | 52 | GC0XM154010 | 1.564250469 |
| MPP1 | MAGUK P55 Scaffold Protein 1 | Protein Coding | 41 | GC0XM154779 | 1.561486006 |
| CETP | Cholesteryl Ester Transfer Protein | Protein Coding | 49 | GC16P056961 | 1.560286283 |
| IL2RA | Interleukin 2 Receptor Subunit Alpha | Protein Coding | 54 | GC10M006010 | 1.556083083 |
| PLA2G6 | Phospholipase A2 Group VI | Protein Coding | 50 | GC22M060879 | 1.553510427 |
| SFTPD | Surfactant Protein D | Protein Coding | 47 | GC10M079937 | 1.551855564 |
| SERPINA6 | Serpin Family A Member 6 | Protein Coding | 48 | GC14M101978 | 1.551276565 |
| DAPK1 | Death Associated Protein Kinase 1 | Protein Coding | 52 | GC09P087497 | 1.550343156 |
| MGMT | O-6-Methylguanine-DNA Methyltransferase | Protein Coding | 52 | GC10P129467 | 1.550343156 |
| ECE1 | Endothelin Converting Enzyme 1 | Protein Coding | 51 | GC01M021217 | 1.548002839 |
| ANG | Angiogenin | Protein Coding | 47 | GC14P033406 | 1.547792912 |
| PTGER1 | Prostaglandin E Receptor 1 | Protein Coding | 46 | GC19M014577 | 1.544271827 |
| MAPK9 | Mitogen-Activated Protein Kinase 9 | Protein Coding | 52 | GC05M180260 | 1.543700457 |
| CALCA | Calcitonin Related Polypeptide Alpha | Protein Coding | 48 | GC11M014945 | 1.543700457 |
| PEBP1 | Phosphatidylethanolamine Binding Protein 1 | Protein Coding | 49 | GC12P118135 | 1.543355823 |
| LEPR | Leptin Receptor | Protein Coding | 53 | GC01P065421 | 1.541353226 |
| DEFB4A | Defensin Beta 4A | Protein Coding | 37 | GC08P007895 | 1.539295197 |
| GDNF | Glial Cell Derived Neurotrophic Factor | Protein Coding | 52 | GC05M037812 | 1.538188219 |
| IL18R1 | Interleukin 18 Receptor 1 | Protein Coding | 46 | GC02P102311 | 1.533751249 |
| EPAS1 | Endothelial PAS Domain Protein 1 | Protein Coding | 52 | GC02P046293 | 1.532172441 |
| IL21 | Interleukin 21 | Protein Coding | 47 | GC04M122612 | 1.530426025 |
| HRAS | HRas Proto-Oncogene, GTPase | Protein Coding | 55 | GC11M003889 | 1.529024363 |
| CLDN1 | Claudin 1 | Protein Coding | 48 | GC03M190305 | 1.525100112 |
| TLR7 | Toll Like Receptor 7 | Protein Coding | 50 | GC0XP012867 | 1.521039486 |
| CRAT | Carnitine O-Acetyltransferase | Protein Coding | 47 | GC09M129094 | 1.518728733 |
| RUNX1 | RUNX Family Transcription Factor 1 | Protein Coding | 51 | GC21M034787 | 1.515471697 |
| PIK3CB | Phosphatidylinositol-4,5-Bisphosphate 3-Kinase Catalytic Subunit Beta | Protein Coding | 50 | GC03M138652 | 1.513398647 |
| BCL3 | BCL3 Transcription Coactivator | Protein Coding | 43 | GC19P044747 | 1.513338566 |
| THBS4 | Thrombospondin 4 | Protein Coding | 45 | GC05P079991 | 1.512275815 |
| TNFSF4 | TNF Superfamily Member 4 | Protein Coding | 46 | GC01M173183 | 1.509679675 |
| CGA | Glycoprotein Hormones, Alpha Polypeptide | Protein Coding | 47 | GC06M087085 | 1.506160378 |
| SLPI | Secretory Leukocyte Peptidase Inhibitor | Protein Coding | 42 | GC20M045252 | 1.505995274 |
| CLU | Clusterin | Protein Coding | 50 | GC08M027596 | 1.504516125 |
| MIR29A | MicroRNA 29a | RNA Gene | 23 | GC07M130876 | 1.504516125 |
| ACE2 | Angiotensin Converting Enzyme 2 | Protein Coding | 51 | GC0XM015494 | 1.504426956 |
| JUND | JunD Proto-Oncogene, AP-1 Transcription Factor Subunit | Protein Coding | 44 | GC19M018279 | 1.504052639 |
| PTTG1 | PTTG1 Regulator Of Sister Chromatid Separation, Securin | Protein Coding | 43 | GC05P160422 | 1.501831651 |
| ADORA2B | Adenosine A2b Receptor | Protein Coding | 50 | GC17P017755 | 1.500846624 |
| PROCR | Protein C Receptor | Protein Coding | 44 | GC20P035171 | 1.498074055 |
| PCNT | Pericentrin | Protein Coding | 47 | GC21P046324 | 1.495278835 |
| AMBP | Alpha-1-Microglobulin/Bikunin Precursor | Protein Coding | 47 | GC09M114060 | 1.493515015 |
| LIPC | Lipase C, Hepatic Type | Protein Coding | 48 | GC15P058410 | 1.493282795 |
| TRAF2 | TNF Receptor Associated Factor 2 | Protein Coding | 47 | GC09P136881 | 1.493282795 |
| ECM1 | Extracellular Matrix Protein 1 | Protein Coding | 47 | GC01P150508 | 1.492474079 |
| GNRH2 | Gonadotropin Releasing Hormone 2 | Protein Coding | 38 | GC20P003043 | 1.492426038 |
| PTPN6 | Protein Tyrosine Phosphatase Non-Receptor Type 6 | Protein Coding | 51 | GC12P022382 | 1.491520762 |
| CYP1A2 | Cytochrome P450 Family 1 Subfamily A Member 2 | Protein Coding | 48 | GC15P074748 | 1.48990798 |
| CAPN2 | Calpain 2 | Protein Coding | 50 | GC01P223701 | 1.488426924 |
| RB1 | RB Transcriptional Corepressor 1 | Protein Coding | 50 | GC13P048303 | 1.488426924 |
| DEFB1 | Defensin Beta 1 | Protein Coding | 42 | GC08M006870 | 1.486510158 |
| PRKAA1 | Protein Kinase AMP-Activated Catalytic Subunit Alpha 1 | Protein Coding | 50 | GC05M040759 | 1.485916734 |
| PDGFRB | Platelet Derived Growth Factor Receptor Beta | Protein Coding | 56 | GC05M150113 | 1.480655909 |
| ATF2 | Activating Transcription Factor 2 | Protein Coding | 50 | GC02M175072 | 1.480655909 |
| FGF4 | Fibroblast Growth Factor 4 | Protein Coding | 45 | GC11M097247 | 1.480655909 |
| EDNRA | Endothelin Receptor Type A | Protein Coding | 52 | GC04P147480 | 1.478384018 |
| ALCAM | Activated Leukocyte Cell Adhesion Molecule | Protein Coding | 46 | GC03P105366 | 1.477079391 |
| TYMP | Thymidine Phosphorylase | Protein Coding | 50 | GC22M050525 | 1.474748135 |
| SOAT1 | Sterol O-Acyltransferase 1 | Protein Coding | 47 | GC01P179262 | 1.474748135 |
| BCL6 | BCL6 Transcription Repressor | Protein Coding | 50 | GC03M187721 | 1.470303893 |
| CYP11B2 | Cytochrome P450 Family 11 Subfamily B Member 2 | Protein Coding | 50 | GC08M142910 | 1.470303893 |
| F12 | Coagulation Factor XII | Protein Coding | 52 | GC05M177402 | 1.466608524 |
| SH3PXD2B | SH3 And PX Domains 2B | Protein Coding | 41 | GC05M172325 | 1.466327667 |
| SPINK1 | Serine Peptidase Inhibitor Kazal Type 1 | Protein Coding | 44 | GC05M147825 | 1.465693355 |
| CDK2 | Cyclin Dependent Kinase 2 | Protein Coding | 54 | GC12P055966 | 1.464715481 |
| ROR2 | Receptor Tyrosine Kinase Like Orphan Receptor 2 | Protein Coding | 51 | GC09M095287 | 1.464715481 |
| FGF23 | Fibroblast Growth Factor 23 | Protein Coding | 48 | GC12M004368 | 1.464715481 |
| MIR125A | MicroRNA 125a | RNA Gene | 24 | GC19P070321 | 1.462244034 |
| PTPN11 | Protein Tyrosine Phosphatase Non-Receptor Type 11 | Protein Coding | 55 | GC12P112418 | 1.46217227 |
| LAMA5 | Laminin Subunit Alpha 5 | Protein Coding | 46 | GC20M062307 | 1.46217227 |
| LUM | Lumican | Protein Coding | 44 | GC12M091102 | 1.46217227 |
| AURKA | Aurora Kinase A | Protein Coding | 53 | GC20M056370 | 1.459102392 |
| FOXC1 | Forkhead Box C1 | Protein Coding | 44 | GC06P001610 | 1.457780361 |
| ZEB2 | Zinc Finger E-Box Binding Homeobox 2 | Protein Coding | 50 | GC02M144384 | 1.452727556 |
| EREG | Epiregulin | Protein Coding | 45 | GC04P074366 | 1.450824618 |
| NAT1 | N-Acetyltransferase 1 | Protein Coding | 45 | GC08P018179 | 1.446786761 |
| GATA3 | GATA Binding Protein 3 | Protein Coding | 51 | GC10P008045 | 1.443397403 |
| RNU7-159P | RNA, U7 Small Nuclear 159 Pseudogene | Pseudogene | 7 | GC11M102903 | 1.440665603 |
| HDAC4 | Histone Deacetylase 4 | Protein Coding | 54 | GC02M239048 | 1.435160518 |
| CDKN2B | Cyclin Dependent Kinase Inhibitor 2B | Protein Coding | 49 | GC09M022002 | 1.435160518 |
| PTK7 | Protein Tyrosine Kinase 7 (Inactive) | Protein Coding | 47 | GC06P043076 | 1.435160518 |
| NR4A2 | Nuclear Receptor Subfamily 4 Group A Member 2 | Protein Coding | 49 | GC02M156324 | 1.434413671 |
| C1QBP | Complement C1q Binding Protein | Protein Coding | 47 | GC17M005432 | 1.433970928 |
| CYCS | Cytochrome C, Somatic | Protein Coding | 51 | GC07M025118 | 1.433290839 |
| COL7A1 | Collagen Type VII Alpha 1 Chain | Protein Coding | 47 | GC03M048564 | 1.43306601 |
| GPX4 | Glutathione Peroxidase 4 | Protein Coding | 48 | GC19P001103 | 1.432110667 |
| INHBA | Inhibin Subunit Beta A | Protein Coding | 48 | GC07M041668 | 1.431658149 |
| GRP | Gastrin Releasing Peptide | Protein Coding | 43 | GC18P059220 | 1.43049109 |
| SDC4 | Syndecan 4 | Protein Coding | 47 | GC20M045325 | 1.42984271 |
| LPA | Lipoprotein(A) | Protein Coding | 41 | GC06M160531 | 1.42984271 |
| TNFSF12 | TNF Superfamily Member 12 | Protein Coding | 44 | GC17P011456 | 1.427128673 |
| TNFSF14 | TNF Superfamily Member 14 | Protein Coding | 44 | GC19M006663 | 1.420709252 |
| PTPN1 | Protein Tyrosine Phosphatase Non-Receptor Type 1 | Protein Coding | 54 | GC20P050510 | 1.420484543 |
| NOX4 | NADPH Oxidase 4 | Protein Coding | 47 | GC11M089324 | 1.420484543 |
| SMPD3 | Sphingomyelin Phosphodiesterase 3 | Protein Coding | 43 | GC16M068358 | 1.419368982 |
| APOB | Apolipoprotein B | Protein Coding | 50 | GC02M020956 | 1.418196201 |
| GSTP1 | Glutathione S-Transferase Pi 1 | Protein Coding | 53 | GC11P067583 | 1.417824149 |
| AKT3 | AKT Serine/Threonine Kinase 3 | Protein Coding | 57 | GC01M243488 | 1.417625785 |
| CD46 | CD46 Molecule | Protein Coding | 50 | GC01P207752 | 1.417316437 |
| TYRP1 | Tyrosinase Related Protein 1 | Protein Coding | 50 | GC09P012683 | 1.416484952 |
| NOD1 | Nucleotide Binding Oligomerization Domain Containing 1 | Protein Coding | 45 | GC07M030424 | 1.416210175 |
| YBX1 | Y-Box Binding Protein 1 | Protein Coding | 42 | GC01P042682 | 1.412586093 |
| BACE1 | Beta-Secretase 1 | Protein Coding | 50 | GC11M117285 | 1.412535787 |
| PDGFC | Platelet Derived Growth Factor C | Protein Coding | 46 | GC04M156760 | 1.411741018 |
| GLI1 | GLI Family Zinc Finger 1 | Protein Coding | 50 | GC12P057462 | 1.409402728 |
| CD34 | CD34 Molecule | Protein Coding | 48 | GC01M207880 | 1.409402728 |
| SYN2 | Synapsin II | Protein Coding | 41 | GC03P012736 | 1.406667709 |
| CCR2 | C-C Motif Chemokine Receptor 2 | Protein Coding | 48 | GC03P047181 | 1.40563798 |
| CD38 | CD38 Molecule | Protein Coding | 49 | GC04P018711 | 1.405204296 |
| TLR6 | Toll Like Receptor 6 | Protein Coding | 47 | GC04M038828 | 1.404965758 |
| FIBP | FGF1 Intracellular Binding Protein | Protein Coding | 44 | GC11M097088 | 1.404858708 |
| TREM1 | Triggering Receptor Expressed On Myeloid Cells 1 | Protein Coding | 44 | GC06M041267 | 1.404858708 |
| FMOD | Fibromodulin | Protein Coding | 45 | GC01M203340 | 1.403977394 |
| CCNB1 | Cyclin B1 | Protein Coding | 49 | GC05P069167 | 1.403407574 |
| ACP5 | Acid Phosphatase 5, Tartrate Resistant | Protein Coding | 49 | GC19M011574 | 1.400507569 |
| LAMA3 | Laminin Subunit Alpha 3 | Protein Coding | 48 | GC18P023689 | 1.399370074 |
| NTRK1 | Neurotrophic Receptor Tyrosine Kinase 1 | Protein Coding | 55 | GC01P156815 | 1.395961881 |
| NPY | Neuropeptide Y | Protein Coding | 47 | GC07P024290 | 1.395961881 |
| PRKCB | Protein Kinase C Beta | Protein Coding | 50 | GC16P024412 | 1.394891739 |
| FGF9 | Fibroblast Growth Factor 9 | Protein Coding | 49 | GC13P021671 | 1.3913064 |
| MUC16 | Mucin 16, Cell Surface Associated | Protein Coding | 40 | GC19M008848 | 1.388759494 |
| LEFTY2 | Left-Right Determination Factor 2 | Protein Coding | 45 | GC01M225937 | 1.388070822 |
| KRT14 | Keratin 14 | Protein Coding | 49 | GC17M041582 | 1.387382269 |
| HAVCR2 | Hepatitis A Virus Cellular Receptor 2 | Protein Coding | 48 | GC05M157063 | 1.384773016 |
| HSPA4 | Heat Shock Protein Family A (Hsp70) Member 4 | Protein Coding | 45 | GC05P133051 | 1.384773016 |
| RPS6KB1 | Ribosomal Protein S6 Kinase B1 | Protein Coding | 51 | GC17P059893 | 1.384015918 |
| NFKBIB | NFKB Inhibitor Beta | Protein Coding | 44 | GC19P038899 | 1.382759333 |
| HDAC7 | Histone Deacetylase 7 | Protein Coding | 48 | GC12M047782 | 1.382753015 |
| CEACAM5 | CEA Cell Adhesion Molecule 5 | Protein Coding | 45 | GC19P069849 | 1.381010771 |
| THBD | Thrombomodulin | Protein Coding | 49 | GC20M023026 | 1.380117297 |
| MEP1A | Meprin A Subunit Alpha | Protein Coding | 44 | GC06P046793 | 1.379096508 |
| ABCB1 | ATP Binding Cassette Subfamily B Member 1 | Protein Coding | 53 | GC07M087504 | 1.376770616 |
| CSPG4 | Chondroitin Sulfate Proteoglycan 4 | Protein Coding | 50 | GC15M075674 | 1.375680685 |
| MIR491 | MicroRNA 491 | RNA Gene | 21 | GC09P020716 | 1.375680685 |
| MBP | Myelin Basic Protein | Protein Coding | 47 | GC18M076978 | 1.373105764 |
| CCL15 | C-C Motif Chemokine Ligand 15 | Protein Coding | 37 | GC17M035996 | 1.373105764 |
| COL17A1 | Collagen Type XVII Alpha 1 Chain | Protein Coding | 47 | GC10M104031 | 1.369943976 |
| LDHA | Lactate Dehydrogenase A | Protein Coding | 54 | GC11P018394 | 1.369815111 |
| SNCA | Synuclein Alpha | Protein Coding | 54 | GC04M089724 | 1.369815111 |
| MAPKAPK2 | MAPK Activated Protein Kinase 2 | Protein Coding | 50 | GC01P206684 | 1.369815111 |
| GHR | Growth Hormone Receptor | Protein Coding | 49 | GC05P042429 | 1.368878365 |
| BAK1 | BCL2 Antagonist/Killer 1 | Protein Coding | 47 | GC06M033572 | 1.368878365 |
| PROC | Protein C, Inactivator Of Coagulation Factors Va And VIIIa | Protein Coding | 54 | GC02P127418 | 1.368637085 |
| COL6A3 | Collagen Type VI Alpha 3 Chain | Protein Coding | 48 | GC02M237324 | 1.368637085 |
| SERPINF2 | Serpin Family F Member 2 | Protein Coding | 48 | GC17P001742 | 1.368637085 |
| INS | Insulin | Protein Coding | 50 | GC11M002159 | 1.366602898 |
| SMARCA4 | SWI/SNF Related, Matrix Associated, Actin Dependent Regulator Of Chromatin, Subfamily A, Member 4 | Protein Coding | 53 | GC19P010932 | 1.366334796 |
| WFIKKN1 | WAP, Follistatin/Kazal, Immunoglobulin, Kunitz And Netrin Domain Containing 1 | Protein Coding | 37 | GC16P012607 | 1.365848184 |
| FCGR2A | Fc Gamma Receptor IIa | Protein Coding | 50 | GC01P161505 | 1.36200738 |
| SERPINB1 | Serpin Family B Member 1 | Protein Coding | 43 | GC06M002847 | 1.360882759 |
| ADA | Adenosine Deaminase | Protein Coding | 54 | GC20M044620 | 1.360274553 |
| PAK4 | P21 (RAC1) Activated Kinase 4 | Protein Coding | 51 | GC19P039125 | 1.358892798 |
| SPOCK1 | SPARC (Osteonectin), Cwcv And Kazal Like Domains Proteoglycan 1 | Protein Coding | 41 | GC05M136975 | 1.358892798 |
| HDAC2 | Histone Deacetylase 2 | Protein Coding | 54 | GC06M113933 | 1.356853485 |
| CAPN1 | Calpain 1 | Protein Coding | 53 | GC11P070408 | 1.356317878 |
| VEGFB | Vascular Endothelial Growth Factor B | Protein Coding | 47 | GC11P064234 | 1.354756117 |
| STX2 | Syntaxin 2 | Protein Coding | 40 | GC12M130789 | 1.354081154 |
| ARG1 | Arginase 1 | Protein Coding | 52 | GC06P131473 | 1.353543282 |
| MAP3K7 | Mitogen-Activated Protein Kinase Kinase Kinase 7 | Protein Coding | 53 | GC06M090513 | 1.350709081 |
| SNAP23 | Synaptosome Associated Protein 23 | Protein Coding | 44 | GC15P042491 | 1.34962225 |
| CIROP | Ciliated Left-Right Organizer Metallopeptidase | Protein Coding | 16 | GC14M023185 | 1.345358372 |
| EIF4E | Eukaryotic Translation Initiation Factor 4E | Protein Coding | 52 | GC04M098879 | 1.344304442 |
| AICDA | Activation Induced Cytidine Deaminase | Protein Coding | 45 | GC12M008602 | 1.342585921 |
| NTS | Neurotensin | Protein Coding | 43 | GC12P085876 | 1.340878487 |
| CTSS | Cathepsin S | Protein Coding | 48 | GC01M150730 | 1.340637088 |
| XRCC1 | X-Ray Repair Cross Complementing 1 | Protein Coding | 46 | GC19M043543 | 1.338576555 |
| STX4 | Syntaxin 4 | Protein Coding | 44 | GC16P041942 | 1.338576555 |
| DEFA1 | Defensin Alpha 1 | Protein Coding | 41 | GC08M006977 | 1.338062167 |
| FGFR2 | Fibroblast Growth Factor Receptor 2 | Protein Coding | 58 | GC10M121478 | 1.337067485 |
| MIR221 | MicroRNA 221 | RNA Gene | 22 | GC0XM045746 | 1.337067485 |
| PTAFR | Platelet Activating Factor Receptor | Protein Coding | 47 | GC01M028147 | 1.334969044 |
| SERPINB5 | Serpin Family B Member 5 | Protein Coding | 45 | GC18P063476 | 1.334969044 |
| CD80 | CD80 Molecule | Protein Coding | 48 | GC03M119524 | 1.334665775 |
| CD86 | CD86 Molecule | Protein Coding | 46 | GC03P122055 | 1.334665775 |
| HP | Haptoglobin | Protein Coding | 48 | GC16P072191 | 1.330685139 |
| SLC23A2 | Solute Carrier Family 23 Member 2 | Protein Coding | 44 | GC20M004852 | 1.330685139 |
| IL32 | Interleukin 32 | Protein Coding | 40 | GC16P012726 | 1.33039856 |
| ENAM | Enamelin | Protein Coding | 41 | GC04P070628 | 1.327121019 |
| KRT18 | Keratin 18 | Protein Coding | 51 | GC12P052948 | 1.326682806 |
| LOXL2 | Lysyl Oxidase Like 2 | Protein Coding | 50 | GC08M023296 | 1.325038552 |
| HMGA1 | High Mobility Group AT-Hook 1 | Protein Coding | 48 | GC06P087865 | 1.322354078 |
| AGRN | Agrin | Protein Coding | 47 | GC01P001020 | 1.321788669 |
| MRC1 | Mannose Receptor C-Type 1 | Protein Coding | 41 | GC10P017809 | 1.321788669 |
| ASTL | Astacin Like Metalloendopeptidase | Protein Coding | 38 | GC02M098493 | 1.321564913 |
| CXCL13 | C-X-C Motif Chemokine Ligand 13 | Protein Coding | 44 | GC04P077511 | 1.316517591 |
| BAD | BCL2 Associated Agonist Of Cell Death | Protein Coding | 47 | GC11M096993 | 1.316009045 |
| NR4A1 | Nuclear Receptor Subfamily 4 Group A Member 1 | Protein Coding | 50 | GC12P052022 | 1.315665483 |
| TSC1 | TSC Complex Subunit 1 | Protein Coding | 50 | GC09M132891 | 1.315418839 |
| FGFR1 | Fibroblast Growth Factor Receptor 1 | Protein Coding | 59 | GC08M038400 | 1.313805223 |
| FGB | Fibrinogen Beta Chain | Protein Coding | 50 | GC04P154598 | 1.312321544 |
| WNT3A | Wnt Family Member 3A | Protein Coding | 50 | GC01P229424 | 1.311323643 |
| CRH | Corticotropin Releasing Hormone | Protein Coding | 45 | GC08M066176 | 1.310430527 |
| F5 | Coagulation Factor V | Protein Coding | 50 | GC01M169511 | 1.307826638 |
| CTNND1 | Catenin Delta 1 | Protein Coding | 48 | GC11P058173 | 1.307666421 |
| NCL | Nucleolin | Protein Coding | 47 | GC02M231453 | 1.307666421 |
| HLA-E | Major Histocompatibility Complex, Class I, E | Protein Coding | 44 | GC06P087699 | 1.30354476 |
| KIT | KIT Proto-Oncogene, Receptor Tyrosine Kinase | Protein Coding | 56 | GC04P054657 | 1.301132321 |
| GPI | Glucose-6-Phosphate Isomerase | Protein Coding | 51 | GC19P034359 | 1.300383329 |
| EPX | Eosinophil Peroxidase | Protein Coding | 46 | GC17P058192 | 1.299234748 |
| F7 | Coagulation Factor VII | Protein Coding | 51 | GC13P113105 | 1.296695709 |
| RARA | Retinoic Acid Receptor Alpha | Protein Coding | 52 | GC17P040309 | 1.295472622 |
| SP3 | Sp3 Transcription Factor | Protein Coding | 45 | GC02M173882 | 1.294977427 |
| ANXA1 | Annexin A1 | Protein Coding | 51 | GC09P073151 | 1.292773247 |
| CD274 | CD274 Molecule | Protein Coding | 47 | GC09P005450 | 1.28941071 |
| PDCD4 | Programmed Cell Death 4 | Protein Coding | 45 | GC10P110871 | 1.28941071 |
| TYMS | Thymidylate Synthetase | Protein Coding | 51 | GC18P000657 | 1.287333965 |
| GH1 | Growth Hormone 1 | Protein Coding | 46 | GC17M063917 | 1.287333965 |
| SLC23A1 | Solute Carrier Family 23 Member 1 | Protein Coding | 43 | GC05M139377 | 1.287333965 |
| C5AR1 | Complement C5a Receptor 1 | Protein Coding | 47 | GC19P047290 | 1.286528111 |
| KLK2 | Kallikrein Related Peptidase 2 | Protein Coding | 47 | GC19P050861 | 1.286283851 |
| CTRB1 | Chymotrypsinogen B1 | Protein Coding | 41 | GC16P075218 | 1.286283851 |
| CTRB2 | Chymotrypsinogen B2 | Protein Coding | 34 | GC16M075204 | 1.286283851 |
| SPINT1 | Serine Peptidase Inhibitor, Kunitz Type 1 | Protein Coding | 44 | GC15P040844 | 1.283595443 |
| TSPAN7 | Tetraspanin 7 | Protein Coding | 47 | GC0XP038561 | 1.283340096 |
| CASP1 | Caspase 1 | Protein Coding | 51 | GC11M105025 | 1.282766581 |
| EPHA3 | EPH Receptor A3 | Protein Coding | 49 | GC03P089077 | 1.279907942 |
| CFLAR | CASP8 And FADD Like Apoptosis Regulator | Protein Coding | 46 | GC02P201117 | 1.27957058 |
| PZP | PZP Alpha-2-Macroglobulin Like | Protein Coding | 43 | GC12M009148 | 1.278505445 |
| CCL18 | C-C Motif Chemokine Ligand 18 | Protein Coding | 38 | GC17P036064 | 1.278302789 |
| NECTIN4 | Nectin Cell Adhesion Molecule 4 | Protein Coding | 47 | GC01M161071 | 1.272066116 |
| LIMK1 | LIM Domain Kinase 1 | Protein Coding | 51 | GC07P074082 | 1.269217849 |
| KAT2B | Lysine Acetyltransferase 2B | Protein Coding | 50 | GC03P020043 | 1.269217849 |
| DIAPH1 | Diaphanous Related Formin 1 | Protein Coding | 49 | GC05M141516 | 1.269217849 |
| TIAM1 | TIAM Rac1 Associated GEF 1 | Protein Coding | 47 | GC21M031118 | 1.267140865 |
| NES | Nestin | Protein Coding | 42 | GC01M156668 | 1.267140865 |
| CCL1 | C-C Motif Chemokine Ligand 1 | Protein Coding | 43 | GC17M045421 | 1.266335249 |
| DCUN1D5 | Defective In Cullin Neddylation 1 Domain Containing 5 | Protein Coding | 34 | GC11M103051 | 1.266031623 |
| LGI1 | Leucine Rich Glioma Inactivated 1 | Protein Coding | 46 | GC10P093757 | 1.265339732 |
| CCL23 | C-C Motif Chemokine Ligand 23 | Protein Coding | 39 | GC17M036013 | 1.265339732 |
| MIR200B | MicroRNA 200b | RNA Gene | 23 | GC01P001167 | 1.264246106 |
| ITGB4 | Integrin Subunit Beta 4 | Protein Coding | 52 | GC17P075721 | 1.263757825 |
| AGTR2 | Angiotensin II Receptor Type 2 | Protein Coding | 48 | GC0XP116170 | 1.261652112 |
| PROK1 | Prokineticin 1 | Protein Coding | 41 | GC01P110451 | 1.261652112 |
| MIR150 | MicroRNA 150 | RNA Gene | 23 | GC19M049500 | 1.261652112 |
| COL4A1 | Collagen Type IV Alpha 1 Chain | Protein Coding | 51 | GC13M110148 | 1.258574009 |
| NTRK2 | Neurotrophic Receptor Tyrosine Kinase 2 | Protein Coding | 56 | GC09P084668 | 1.258243918 |
| SYK | Spleen Associated Tyrosine Kinase | Protein Coding | 52 | GC09P092573 | 1.256035209 |
| CFH | Complement Factor H | Protein Coding | 50 | GC01P196621 | 1.253435731 |
| TAP1 | Transporter 1, ATP Binding Cassette Subfamily B Member | Protein Coding | 50 | GC06M069271 | 1.253435731 |
| FBLN5 | Fibulin 5 | Protein Coding | 50 | GC14M091869 | 1.253331065 |
| ANGPTL4 | Angiopoietin Like 4 | Protein Coding | 47 | GC19P008363 | 1.251013637 |
| OXT | Oxytocin/Neurophysin I Prepropeptide | Protein Coding | 43 | GC20P004374 | 1.251013637 |
| CD209 | CD209 Molecule | Protein Coding | 45 | GC19M007739 | 1.250352979 |
| HAS3 | Hyaluronan Synthase 3 | Protein Coding | 44 | GC16P069105 | 1.250352979 |
| C3 | Complement C3 | Protein Coding | 52 | GC19M006677 | 1.249952078 |
| NPPA | Natriuretic Peptide A | Protein Coding | 48 | GC01M011846 | 1.249952078 |
| MICA | MHC Class I Polypeptide-Related Sequence A | Protein Coding | 41 | GC06P031399 | 1.249952078 |
| HSPA5 | Heat Shock Protein Family A (Hsp70) Member 5 | Protein Coding | 51 | GC09M125234 | 1.248526454 |
| CYP3A4 | Cytochrome P450 Family 3 Subfamily A Member 4 | Protein Coding | 52 | GC07M099759 | 1.247812152 |
| LTB4R2 | Leukotriene B4 Receptor 2 | Protein Coding | 45 | GC14P033618 | 1.247812152 |
| PTGDR2 | Prostaglandin D2 Receptor 2 | Protein Coding | 45 | GC11M060850 | 1.247812152 |
| LILRB5 | Leukocyte Immunoglobulin Like Receptor B5 | Protein Coding | 41 | GC19M068416 | 1.247653008 |
| ACTB | Actin Beta | Protein Coding | 52 | GC07M005527 | 1.246297956 |
| MIR27B | MicroRNA 27b | RNA Gene | 22 | GC09P095097 | 1.246297956 |
| CTSF | Cathepsin F | Protein Coding | 51 | GC11M097132 | 1.24504137 |
| MITF | Melanocyte Inducing Transcription Factor | Protein Coding | 50 | GC03P069788 | 1.24504137 |
| CTNNA1 | Catenin Alpha 1 | Protein Coding | 48 | GC05P138613 | 1.24504137 |
| IL9 | Interleukin 9 | Protein Coding | 45 | GC05M135891 | 1.242154241 |
| ICAM3 | Intercellular Adhesion Molecule 3 | Protein Coding | 46 | GC19M010412 | 1.24145937 |
| PPARD | Peroxisome Proliferator Activated Receptor Delta | Protein Coding | 48 | GC06P087880 | 1.24123168 |
| SHC1 | SHC Adaptor Protein 1 | Protein Coding | 46 | GC01M154962 | 1.24123168 |
| SERPINH1 | Serpin Family H Member 1 | Protein Coding | 48 | GC11P075562 | 1.238718629 |
| MIR106A | MicroRNA 106a | RNA Gene | 20 | GC0XM134328 | 1.235689044 |
| COMT | Catechol-O-Methyltransferase | Protein Coding | 54 | GC22P019941 | 1.235387921 |
| P2RX7 | Purinergic Receptor P2X 7 | Protein Coding | 50 | GC12P126599 | 1.232097268 |
| MIR320A | MicroRNA 320a | RNA Gene | 23 | GC08M022271 | 1.232097268 |
| XIAP | X-Linked Inhibitor Of Apoptosis | Protein Coding | 54 | GC0XP123859 | 1.231160402 |
| PARD3 | Par-3 Family Cell Polarity Regulator | Protein Coding | 43 | GC10M034110 | 1.231160402 |
| TNFSF10 | TNF Superfamily Member 10 | Protein Coding | 47 | GC03M172505 | 1.228616834 |
| HLA-DQA1 | Major Histocompatibility Complex, Class II, DQ Alpha 1 | Protein Coding | 44 | GC06P087770 | 1.220505476 |
| SSTR2 | Somatostatin Receptor 2 | Protein Coding | 50 | GC17P073165 | 1.218600035 |
| MIR335 | MicroRNA 335 | RNA Gene | 22 | GC07P130496 | 1.218600035 |
| CNR1 | Cannabinoid Receptor 1 | Protein Coding | 50 | GC06M088139 | 1.218539357 |
| CD68 | CD68 Molecule | Protein Coding | 43 | GC17P007579 | 1.218539357 |
| BTK | Bruton Tyrosine Kinase | Protein Coding | 55 | GC0XM101349 | 1.216228127 |
| MYLK | Myosin Light Chain Kinase | Protein Coding | 54 | GC03M123610 | 1.216228127 |
| S100B | S100 Calcium Binding Protein B | Protein Coding | 47 | GC21M051071 | 1.214977026 |
| CD28 | CD28 Molecule | Protein Coding | 51 | GC02P203706 | 1.21369648 |
| FLNA | Filamin A | Protein Coding | 51 | GC0XM154348 | 1.211904407 |
| NEU3 | Neuraminidase 3 | Protein Coding | 41 | GC11P078203 | 1.211904407 |
| CEACAM1 | CEA Cell Adhesion Molecule 1 | Protein Coding | 48 | GC19M042507 | 1.211828947 |
| PAPLN | Papilin, Proteoglycan Like Sulfated Glycoprotein | Protein Coding | 41 | GC14P073237 | 1.20859468 |
| PRLR | Prolactin Receptor | Protein Coding | 52 | GC05M035048 | 1.206848145 |
| CXCL9 | C-X-C Motif Chemokine Ligand 9 | Protein Coding | 44 | GC04M076001 | 1.204885602 |
| CDKN3 | Cyclin Dependent Kinase Inhibitor 3 | Protein Coding | 44 | GC14P054398 | 1.202362061 |
| CTF1 | Cardiotrophin 1 | Protein Coding | 40 | GC16P041936 | 1.201795697 |
| CTSC | Cathepsin C | Protein Coding | 50 | GC11M097636 | 1.201533318 |
| DAG1 | Dystroglycan 1 | Protein Coding | 49 | GC03P049864 | 1.201533318 |
| ALK | ALK Receptor Tyrosine Kinase | Protein Coding | 54 | GC02M029190 | 1.200344324 |
| CANT1 | Calcium Activated Nucleotidase 1 | Protein Coding | 47 | GC17M078992 | 1.200344324 |
| ID1 | Inhibitor Of DNA Binding 1, HLH Protein | Protein Coding | 44 | GC20P031605 | 1.199317813 |
| IL1RL1 | Interleukin 1 Receptor Like 1 | Protein Coding | 44 | GC02P102294 | 1.195064545 |
| JUP | Junction Plakoglobin | Protein Coding | 50 | GC17M041754 | 1.189314485 |
| TGFBI | Transforming Growth Factor Beta Induced | Protein Coding | 49 | GC05P136027 | 1.189159513 |
| YY1 | YY1 Transcription Factor | Protein Coding | 51 | GC14P100238 | 1.188984513 |
| TMPRSS15 | Transmembrane Serine Protease 15 | Protein Coding | 46 | GC21M018269 | 1.188984513 |
| SERPINB3 | Serpin Family B Member 3 | Protein Coding | 43 | GC18M063655 | 1.188984513 |
| FUT4 | Fucosyltransferase 4 | Protein Coding | 40 | GC11P094544 | 1.188984513 |
| MIR204 | MicroRNA 204 | RNA Gene | 25 | GC09M070809 | 1.188984513 |
| CCN4 | Cellular Communication Network Factor 4 | Protein Coding | 41 | GC08P133192 | 1.187218428 |
| SIRT6 | Sirtuin 6 | Protein Coding | 47 | GC19M004174 | 1.186857224 |
| SKIL | SKI Like Proto-Oncogene | Protein Coding | 44 | GC03P170357 | 1.186857224 |
| ZNF148 | Zinc Finger Protein 148 | Protein Coding | 43 | GC03M125225 | 1.186857224 |
| TAGLN | Transgelin | Protein Coding | 46 | GC11P117199 | 1.184840679 |
| DNMT1 | DNA Methyltransferase 1 | Protein Coding | 55 | GC19M010133 | 1.184070706 |
| CXCL11 | C-X-C Motif Chemokine Ligand 11 | Protein Coding | 46 | GC04M076033 | 1.184070706 |
| KNG1 | Kininogen 1 | Protein Coding | 50 | GC03P186717 | 1.18199861 |
| CYSLTR2 | Cysteinyl Leukotriene Receptor 2 | Protein Coding | 50 | GC13P048653 | 1.180796981 |
| SERPINB4 | Serpin Family B Member 4 | Protein Coding | 38 | GC18M063637 | 1.180796981 |
| DYNC2H1 | Dynein Cytoplasmic 2 Heavy Chain 1 | Protein Coding | 41 | GC11P103109 | 1.180493355 |
| ITGA2B | Integrin Subunit Alpha 2b | Protein Coding | 54 | GC17M049884 | 1.180151463 |
| ARNT | Aryl Hydrocarbon Receptor Nuclear Translocator | Protein Coding | 47 | GC01M150809 | 1.180151463 |
| GOLM1 | Golgi Membrane Protein 1 | Protein Coding | 42 | GC09M086026 | 1.180151463 |
| CBS | Cystathionine Beta-Synthase | Protein Coding | 53 | GC21M043053 | 1.178291202 |
| ERCC2 | ERCC Excision Repair 2, TFIIH Core Complex Helicase Subunit | Protein Coding | 50 | GC19M045349 | 1.178291202 |
| PGF | Placental Growth Factor | Protein Coding | 46 | GC14M074941 | 1.177122355 |
| CALR | Calreticulin | Protein Coding | 54 | GC19P012938 | 1.171231508 |
| VHL | Von Hippel-Lindau Tumor Suppressor | Protein Coding | 49 | GC03P012694 | 1.171231508 |
| CAMP | Cathelicidin Antimicrobial Peptide | Protein Coding | 41 | GC03P049753 | 1.170356274 |
| FGFR4 | Fibroblast Growth Factor Receptor 4 | Protein Coding | 55 | GC05P177086 | 1.165814996 |
| MAPT | Microtubule Associated Protein Tau | Protein Coding | 53 | GC17P045894 | 1.165814996 |
| SRF | Serum Response Factor | Protein Coding | 45 | GC06P043171 | 1.165814996 |
| MC1R | Melanocortin 1 Receptor | Protein Coding | 50 | GC16P089912 | 1.162665367 |
| CAPNS1 | Calpain Small Subunit 1 | Protein Coding | 42 | GC19P069651 | 1.162665367 |
| VAMP3 | Vesicle Associated Membrane Protein 3 | Protein Coding | 43 | GC01P007765 | 1.161797285 |
| VSIR | V-Set Immunoregulatory Receptor | Protein Coding | 41 | GC10M071748 | 1.161797285 |
| SLC2A1 | Solute Carrier Family 2 Member 1 | Protein Coding | 56 | GC01M042925 | 1.161401033 |
| LPAR1 | Lysophosphatidic Acid Receptor 1 | Protein Coding | 49 | GC09M110873 | 1.161401033 |
| ROCK2 | Rho Associated Coiled-Coil Containing Protein Kinase 2 | Protein Coding | 49 | GC02M011320 | 1.161401033 |
| SLIT2 | Slit Guidance Ligand 2 | Protein Coding | 47 | GC04P020287 | 1.161159515 |
| CGB3 | Chorionic Gonadotropin Subunit Beta 3 | Protein Coding | 38 | GC19M068151 | 1.161159515 |
| CA9 | Carbonic Anhydrase 9 | Protein Coding | 50 | GC09P035673 | 1.16060257 |
| LIPE | Lipase E, Hormone Sensitive Type | Protein Coding | 50 | GC19M042401 | 1.157231569 |
| LAMA1 | Laminin Subunit Alpha 1 | Protein Coding | 49 | GC18M006941 | 1.157231569 |
| PRDX5 | Peroxiredoxin 5 | Protein Coding | 47 | GC11P064435 | 1.157231569 |
| CLDN2 | Claudin 2 | Protein Coding | 44 | GC0XP106900 | 1.157231569 |
| MT2A | Metallothionein 2A | Protein Coding | 44 | GC16P057080 | 1.157231569 |
| IAPP | Islet Amyloid Polypeptide | Protein Coding | 43 | GC12P021354 | 1.157231569 |
| SAA2 | Serum Amyloid A2 | Protein Coding | 37 | GC11M018238 | 1.157231569 |
| MIR133A1 | MicroRNA 133a-1 | RNA Gene | 19 | GC18M025563 | 1.157231569 |
| CCN3 | Cellular Communication Network Factor 3 | Protein Coding | 41 | GC08P119416 | 1.154443622 |
| DRD2 | Dopamine Receptor D2 | Protein Coding | 52 | GC11M113409 | 1.149616003 |
| FABP4 | Fatty Acid Binding Protein 4 | Protein Coding | 47 | GC08M081478 | 1.149616003 |
| S1PR1 | Sphingosine-1-Phosphate Receptor 1 | Protein Coding | 47 | GC01P101236 | 1.147520065 |
| WNT1 | Wnt Family Member 1 | Protein Coding | 47 | GC12P049498 | 1.147520065 |
| WNT7A | Wnt Family Member 7A | Protein Coding | 51 | GC03M021328 | 1.1458776 |
| EPHX2 | Epoxide Hydrolase 2 | Protein Coding | 50 | GC08P027490 | 1.1458776 |
| MTR | 5-Methyltetrahydrofolate-Homocysteine Methyltransferase | Protein Coding | 50 | GC01P236795 | 1.1458776 |
| VIPR1 | Vasoactive Intestinal Peptide Receptor 1 | Protein Coding | 48 | GC03P042490 | 1.1458776 |
| NLRP12 | NLR Family Pyrin Domain Containing 12 | Protein Coding | 46 | GC19M053793 | 1.1458776 |
| GGT1 | Gamma-Glutamyltransferase 1 | Protein Coding | 52 | GC22P024583 | 1.145622134 |
| MAP3K1 | Mitogen-Activated Protein Kinase Kinase Kinase 1 | Protein Coding | 52 | GC05P056815 | 1.145622134 |
| PLEK | Pleckstrin | Protein Coding | 43 | GC02P068365 | 1.145622134 |
| CXCR6 | C-X-C Motif Chemokine Receptor 6 | Protein Coding | 44 | GC03P047170 | 1.144976735 |
| ZBTB33 | Zinc Finger And BTB Domain Containing 33 | Protein Coding | 40 | GC0XP120250 | 1.144976735 |
| SFRP1 | Secreted Frizzled Related Protein 1 | Protein Coding | 47 | GC08M041262 | 1.138009906 |
| DHFR | Dihydrofolate Reductase | Protein Coding | 51 | GC05M080626 | 1.134939909 |
| AKT2 | AKT Serine/Threonine Kinase 2 | Protein Coding | 56 | GC19M040230 | 1.132843971 |
| CASR | Calcium Sensing Receptor | Protein Coding | 53 | GC03P122183 | 1.132843971 |
| HNF4A | Hepatocyte Nuclear Factor 4 Alpha | Protein Coding | 52 | GC20P044355 | 1.132843971 |
| COX5A | Cytochrome C Oxidase Subunit 5A | Protein Coding | 45 | GC15M074919 | 1.132843971 |
| MIR205 | MicroRNA 205 | RNA Gene | 23 | GC01P209432 | 1.132843971 |
| RXRA | Retinoid X Receptor Alpha | Protein Coding | 51 | GC09P134317 | 1.13184607 |
| ITGB5 | Integrin Subunit Beta 5 | Protein Coding | 50 | GC03M124761 | 1.13184607 |
| MAP3K3 | Mitogen-Activated Protein Kinase Kinase Kinase 3 | Protein Coding | 50 | GC17P063622 | 1.130044222 |
| IL21R | Interleukin 21 Receptor | Protein Coding | 48 | GC16P028156 | 1.130044222 |
| VAV1 | Vav Guanine Nucleotide Exchange Factor 1 | Protein Coding | 48 | GC19P006772 | 1.130044222 |
| SMAD1 | SMAD Family Member 1 | Protein Coding | 45 | GC04P145481 | 1.130044222 |
| POU2F3 | POU Class 2 Homeobox 3 | Protein Coding | 41 | GC11P120236 | 1.130044222 |
| POU3F1 | POU Class 3 Homeobox 1 | Protein Coding | 40 | GC01M038120 | 1.130044222 |
| PRKCE | Protein Kinase C Epsilon | Protein Coding | 51 | GC02P045651 | 1.128188848 |
| TET1 | Tet Methylcytosine Dioxygenase 1 | Protein Coding | 41 | GC10P068560 | 1.128188848 |
| MIR16-1 | MicroRNA 16-1 | RNA Gene | 22 | GC13M050048 | 1.128188848 |
| CASP7 | Caspase 7 | Protein Coding | 52 | GC10P113679 | 1.12762177 |
| FGF10 | Fibroblast Growth Factor 10 | Protein Coding | 50 | GC05M044340 | 1.12762177 |
| ROBO1 | Roundabout Guidance Receptor 1 | Protein Coding | 47 | GC03M078597 | 1.12762177 |
| VAMP7 | Vesicle Associated Membrane Protein 7 | Protein Coding | 42 | GC0XP155881 | 1.12762177 |
| CNTF | Ciliary Neurotrophic Factor | Protein Coding | 43 | GC11P058622 | 1.125910521 |
| ADAMTSL4 | ADAMTS Like 4 | Protein Coding | 41 | GC01P150549 | 1.123909473 |
| TOLLIP | Toll Interacting Protein | Protein Coding | 46 | GC11M001274 | 1.122702241 |
| VANGL2 | VANGL Planar Cell Polarity Protein 2 | Protein Coding | 44 | GC01P160400 | 1.122702241 |
| CCNT1 | Cyclin T1 | Protein Coding | 43 | GC12M048688 | 1.122702241 |
| RTN4R | Reticulon 4 Receptor | Protein Coding | 47 | GC22M020241 | 1.121806502 |
| PVT1 | Pvt1 Oncogene | RNA Gene | 27 | GC08P127877 | 1.119495392 |
| NPC1 | NPC Intracellular Cholesterol Transporter 1 | Protein Coding | 52 | GC18M023506 | 1.119314194 |
| MCL1 | MCL1 Apoptosis Regulator, BCL2 Family Member | Protein Coding | 50 | GC01M152371 | 1.119314194 |
| CYP11B1 | Cytochrome P450 Family 11 Subfamily B Member 1 | Protein Coding | 48 | GC08M142872 | 1.119314194 |
| FDFT1 | Farnesyl-Diphosphate Farnesyltransferase 1 | Protein Coding | 47 | GC08P011795 | 1.119314194 |
| SHBG | Sex Hormone Binding Globulin | Protein Coding | 44 | GC17P007613 | 1.119314194 |
| PARG | Poly(ADP-Ribose) Glycohydrolase | Protein Coding | 42 | GC10M049818 | 1.119314194 |
| SHH | Sonic Hedgehog Signaling Molecule | Protein Coding | 54 | GC07M155799 | 1.117218256 |
| SERPINE2 | Serpin Family E Member 2 | Protein Coding | 44 | GC02M223975 | 1.117218256 |
| PEG10 | Paternally Expressed 10 | Protein Coding | 42 | GC07P094656 | 1.117218256 |
| TMSB4X | Thymosin Beta 4 X-Linked | Protein Coding | 42 | GC0XP012975 | 1.117218256 |
| COL6A2 | Collagen Type VI Alpha 2 Chain | Protein Coding | 48 | GC21P046098 | 1.115995049 |
| TGFB1I1 | Transforming Growth Factor Beta 1 Induced Transcript 1 | Protein Coding | 44 | GC16P041976 | 1.107429028 |
| TNFSF13B | TNF Superfamily Member 13b | Protein Coding | 47 | GC13P108251 | 1.105998158 |
| NCAM1 | Neural Cell Adhesion Molecule 1 | Protein Coding | 51 | GC11P112961 | 1.103480697 |
| GJA1 | Gap Junction Protein Alpha 1 | Protein Coding | 53 | GC06P121436 | 1.103289008 |
| CALCR | Calcitonin Receptor | Protein Coding | 50 | GC07M093424 | 1.103289008 |
| EPO | Erythropoietin | Protein Coding | 47 | GC07P100720 | 1.103289008 |
| MAP2K7 | Mitogen-Activated Protein Kinase Kinase 7 | Protein Coding | 47 | GC19P007903 | 1.103289008 |
| SLC6A3 | Solute Carrier Family 6 Member 3 | Protein Coding | 53 | GC05M001392 | 1.102526307 |
| HMGCS2 | 3-Hydroxy-3-Methylglutaryl-CoA Synthase 2 | Protein Coding | 50 | GC01M119747 | 1.102526307 |
| S100A6 | S100 Calcium Binding Protein A6 | Protein Coding | 44 | GC01M153565 | 1.102526307 |
| STARD3NL | STARD3 N-Terminal Like | Protein Coding | 38 | GC07P038218 | 1.102526307 |
| CITED2 | Cbp/P300 Interacting Transactivator With Glu/Asp Rich Carboxy-Terminal Domain 2 | Protein Coding | 45 | GC06M139371 | 1.100430369 |
| NOX1 | NADPH Oxidase 1 | Protein Coding | 45 | GC0XM100843 | 1.100430369 |
| CTNNBIP1 | Catenin Beta Interacting Protein 1 | Protein Coding | 43 | GC01M009848 | 1.100093126 |
| PDPK1 | 3-Phosphoinositide Dependent Protein Kinase 1 | Protein Coding | 51 | GC16P002537 | 1.098141432 |
| TFPI | Tissue Factor Pathway Inhibitor | Protein Coding | 48 | GC02M187464 | 1.095515013 |
| SMYD3 | SET And MYND Domain Containing 3 | Protein Coding | 43 | GC01M245749 | 1.095515013 |
| PKM | Pyruvate Kinase M1/2 | Protein Coding | 51 | GC15M072199 | 1.093075156 |
| MTA1 | Metastasis Associated 1 | Protein Coding | 46 | GC14P105419 | 1.093075156 |
| CDH3 | Cadherin 3 | Protein Coding | 50 | GC16P068637 | 1.089740276 |
| STIP1 | Stress Induced Phosphoprotein 1 | Protein Coding | 48 | GC11P064407 | 1.089740276 |
| MAP2K2 | Mitogen-Activated Protein Kinase Kinase 2 | Protein Coding | 56 | GC19M004090 | 1.087663293 |
| CYBB | Cytochrome B-245 Beta Chain | Protein Coding | 51 | GC0XP037780 | 1.087663293 |
| NEU1 | Neuraminidase 1 | Protein Coding | 48 | GC06M031857 | 1.087663293 |
| IL12A | Interleukin 12A | Protein Coding | 46 | GC03P159988 | 1.087663293 |
| CCL21 | C-C Motif Chemokine Ligand 21 | Protein Coding | 44 | GC09M034709 | 1.087663293 |
| ERBB4 | Erb-B2 Receptor Tyrosine Kinase 4 | Protein Coding | 57 | GC02M211375 | 1.086212158 |
| MAPK11 | Mitogen-Activated Protein Kinase 11 | Protein Coding | 51 | GC22M050263 | 1.084509015 |
| TTN | Titin | Protein Coding | 50 | GC02M178525 | 1.084509015 |
| CDK9 | Cyclin Dependent Kinase 9 | Protein Coding | 48 | GC09P128062 | 1.084509015 |
| TNFRSF25 | TNF Receptor Superfamily Member 25 | Protein Coding | 44 | GC01M006460 | 1.084509015 |
| CD47 | CD47 Molecule | Protein Coding | 47 | GC03M108043 | 1.084270597 |
| PECAM1 | Platelet And Endothelial Cell Adhesion Molecule 1 | Protein Coding | 43 | GC17M064319 | 1.083563089 |
| SIK2 | Salt Inducible Kinase 2 | Protein Coding | 48 | GC11P111965 | 1.082174659 |
| FSTL1 | Follistatin Like 1 | Protein Coding | 45 | GC03M120392 | 1.082174659 |
| CCN5 | Cellular Communication Network Factor 5 | Protein Coding | 37 | GC20P044715 | 1.082174659 |
| MAP2K6 | Mitogen-Activated Protein Kinase Kinase 6 | Protein Coding | 50 | GC17P069414 | 1.071536064 |
| PSMC6 | Proteasome 26S Subunit, ATPase 6 | Protein Coding | 43 | GC14P052707 | 1.071536064 |
| CASP9 | Caspase 9 | Protein Coding | 49 | GC01M015491 | 1.070875406 |
| CCR9 | C-C Motif Chemokine Receptor 9 | Protein Coding | 46 | GC03P047167 | 1.070875406 |
| FBLN2 | Fibulin 2 | Protein Coding | 46 | GC03P013590 | 1.070875406 |
| LGMN | Legumain | Protein Coding | 44 | GC14M092703 | 1.070875406 |
| NEDD9 | Neural Precursor Cell Expressed, Developmentally Down-Regulated 9 | Protein Coding | 44 | GC06M011183 | 1.070875406 |
| AZU1 | Azurocidin 1 | Protein Coding | 42 | GC19P000825 | 1.070875406 |
| MAFF | MAF BZIP Transcription Factor F | Protein Coding | 42 | GC22P038200 | 1.070106268 |
| TYR | Tyrosinase | Protein Coding | 53 | GC11P089177 | 1.067891598 |
| CYP17A1 | Cytochrome P450 Family 17 Subfamily A Member 1 | Protein Coding | 51 | GC10M102830 | 1.067891598 |
| DRD3 | Dopamine Receptor D3 | Protein Coding | 49 | GC03M114128 | 1.067891598 |
| CYP2E1 | Cytochrome P450 Family 2 Subfamily E Member 1 | Protein Coding | 47 | GC10P133520 | 1.067891598 |
| APOA4 | Apolipoprotein A4 | Protein Coding | 44 | GC11M116820 | 1.067891598 |
| TNMD | Tenomodulin | Protein Coding | 38 | GC0XP100584 | 1.067891598 |
| GRN | Granulin Precursor | Protein Coding | 51 | GC17P044345 | 1.066820383 |
| SPOCK2 | SPARC (Osteonectin), Cwcv And Kazal Like Domains Proteoglycan 2 | Protein Coding | 40 | GC10M072059 | 1.066820383 |
| HOTAIR | HOX Transcript Antisense RNA | RNA Gene | 26 | GC12M053962 | 1.065695047 |
| STAT5A | Signal Transducer And Activator Of Transcription 5A | Protein Coding | 50 | GC17P042287 | 1.065689325 |
| IDH2 | Isocitrate Dehydrogenase (NADP(+)) 2 | Protein Coding | 55 | GC15M090083 | 1.064077735 |
| DEFB103B | Defensin Beta 103B | Protein Coding | 30 | GC08M007430 | 1.064077735 |
| TFF3 | Trefoil Factor 3 | Protein Coding | 43 | GC21M042311 | 1.061981797 |
| OPTC | Opticin | Protein Coding | 42 | GC01P203494 | 1.061981797 |
| MIR143 | MicroRNA 143 | RNA Gene | 26 | GC05P149410 | 1.061981797 |
| CR1 | Complement C3b/C4b Receptor 1 (Knops Blood Group) | Protein Coding | 47 | GC01P207496 | 1.060438037 |
| CBL | Cbl Proto-Oncogene | Protein Coding | 54 | GC11P119206 | 1.057321787 |
| NFKB2 | Nuclear Factor Kappa B Subunit 2 | Protein Coding | 54 | GC10P102394 | 1.057321787 |
| CYP27B1 | Cytochrome P450 Family 27 Subfamily B Member 1 | Protein Coding | 48 | GC12M057757 | 1.057321787 |
| ENPP2 | Ectonucleotide Pyrophosphatase/Phosphodiesterase 2 | Protein Coding | 48 | GC08M119556 | 1.057321787 |
| RASGRF1 | Ras Protein Specific Guanine Nucleotide Releasing Factor 1 | Protein Coding | 45 | GC15M078959 | 1.057321787 |
| BCAM | Basal Cell Adhesion Molecule (Lutheran Blood Group) | Protein Coding | 43 | GC19P069964 | 1.057321787 |
| LAMTOR1 | Late Endosomal/Lysosomal Adaptor, MAPK And MTOR Activator 1 | Protein Coding | 37 | GC11M072085 | 1.057321787 |
| MIR202 | MicroRNA 202 | RNA Gene | 21 | GC10M133247 | 1.057321787 |
| CSNK1A1P2 | Casein Kinase 1 Alpha 1 Pseudogene 2 | Pseudogene | 5 | GC11M102941 | 1.057321787 |
| ABL1 | ABL Proto-Oncogene 1, Non-Receptor Tyrosine Kinase | Protein Coding | 55 | GC09P130713 | 1.055910349 |
| CCN6 | Cellular Communication Network Factor 6 | Protein Coding | 43 | GC06P112053 | 1.055910349 |
| PARK7 | Parkinsonism Associated Deglycase | Protein Coding | 50 | GC01P008121 | 1.052619696 |
| MIR17 | MicroRNA 17 | RNA Gene | 23 | GC13P091350 | 1.052619696 |
| DRD5 | Dopamine Receptor D5 | Protein Coding | 52 | GC04P009783 | 1.05110383 |
| ADH5 | Alcohol Dehydrogenase 5 (Class III), Chi Polypeptide | Protein Coding | 50 | GC04M099070 | 1.05110383 |
| SHMT2 | Serine Hydroxymethyltransferase 2 | Protein Coding | 48 | GC12P057229 | 1.05110383 |
| APOA2 | Apolipoprotein A2 | Protein Coding | 47 | GC01M161222 | 1.05110383 |
| HSD3B2 | Hydroxy-Delta-5-Steroid Dehydrogenase, 3 Beta- And Steroid Delta-Isomerase 2 | Protein Coding | 47 | GC01P119414 | 1.05110383 |
| SRD5A1 | Steroid 5 Alpha-Reductase 1 | Protein Coding | 47 | GC05P006633 | 1.05110383 |
| STAR | Steroidogenic Acute Regulatory Protein | Protein Coding | 47 | GC08M038145 | 1.05110383 |
| TJP2 | Tight Junction Protein 2 | Protein Coding | 47 | GC09P069121 | 1.05110383 |
| CYP21A2 | Cytochrome P450 Family 21 Subfamily A Member 2 | Protein Coding | 46 | GC06P087764 | 1.05110383 |
| NPC2 | NPC Intracellular Cholesterol Transporter 2 | Protein Coding | 46 | GC14M074476 | 1.05110383 |
| HSD17B2 | Hydroxysteroid 17-Beta Dehydrogenase 2 | Protein Coding | 45 | GC16P082068 | 1.05110383 |
| HSD3B1 | Hydroxy-Delta-5-Steroid Dehydrogenase, 3 Beta- And Steroid Delta-Isomerase 1 | Protein Coding | 45 | GC01P119507 | 1.05110383 |
| OCA2 | OCA2 Melanosomal Transmembrane Protein | Protein Coding | 45 | GC15M027754 | 1.05110383 |
| PDSS1 | Decaprenyl Diphosphate Synthase Subunit 1 | Protein Coding | 45 | GC10P026697 | 1.05110383 |
| TNFRSF14 | TNF Receptor Superfamily Member 14 | Protein Coding | 45 | GC01P004185 | 1.05110383 |
| HMGCS1 | 3-Hydroxy-3-Methylglutaryl-CoA Synthase 1 | Protein Coding | 44 | GC05M044081 | 1.05110383 |
| HSD17B1 | Hydroxysteroid 17-Beta Dehydrogenase 1 | Protein Coding | 44 | GC17P058587 | 1.05110383 |
| BIK | BCL2 Interacting Killer | Protein Coding | 43 | GC22P043110 | 1.05110383 |
| NAT2 | N-Acetyltransferase 2 | Protein Coding | 43 | GC08P018391 | 1.05110383 |
| TIAL1 | TIA1 Cytotoxic Granule Associated RNA Binding Protein Like 1 | Protein Coding | 42 | GC10M119571 | 1.05110383 |
| ULBP2 | UL16 Binding Protein 2 | Protein Coding | 42 | GC06P149941 | 1.05110383 |
| ACBD3 | Acyl-CoA Binding Domain Containing 3 | Protein Coding | 41 | GC01M226144 | 1.05110383 |
| TNFSF8 | TNF Superfamily Member 8 | Protein Coding | 41 | GC09M114893 | 1.05110383 |
| NLRP3 | NLR Family Pyrin Domain Containing 3 | Protein Coding | 50 | GC01P247415 | 1.050895095 |
| STAT5B | Signal Transducer And Activator Of Transcription 5B | Protein Coding | 52 | GC17M042199 | 1.050063491 |
| NAGLU | N-Acetyl-Alpha-Glucosaminidase | Protein Coding | 45 | GC17P058592 | 1.041157842 |
| EBAG9 | Estrogen Receptor Binding Site Associated Antigen 9 | Protein Coding | 41 | GC08P109536 | 1.041157842 |
| MIR127 | MicroRNA 127 | RNA Gene | 23 | GC14P110110 | 1.041157842 |
| KLF4 | KLF Transcription Factor 4 | Protein Coding | 48 | GC09M107484 | 1.039633155 |
| UBE2I | Ubiquitin Conjugating Enzyme E2 I | Protein Coding | 50 | GC16P012625 | 1.039122462 |
| IRS1 | Insulin Receptor Substrate 1 | Protein Coding | 49 | GC02M226731 | 1.039122462 |
| LAMC1 | Laminin Subunit Gamma 1 | Protein Coding | 46 | GC01P182992 | 1.039122462 |
| CCL25 | C-C Motif Chemokine Ligand 25 | Protein Coding | 42 | GC19P008052 | 1.039122462 |
| CLEC4A | C-Type Lectin Domain Family 4 Member A | Protein Coding | 40 | GC12P022411 | 1.039122462 |
| IVL | Involucrin | Protein Coding | 40 | GC01P152919 | 1.039122462 |
| HSPA8 | Heat Shock Protein Family A (Hsp70) Member 8 | Protein Coding | 51 | GC11M123057 | 1.039061904 |
| MSN | Moesin | Protein Coding | 50 | GC0XP065588 | 1.039061904 |
| C5 | Complement C5 | Protein Coding | 49 | GC09M120933 | 1.037006855 |
| S100A16 | S100 Calcium Binding Protein A16 | Protein Coding | 38 | GC01M153606 | 1.034675121 |
| TMEFF2 | Transmembrane Protein With EGF Like And Two Follistatin Like Domains 2 | Protein Coding | 42 | GC02M191950 | 1.034190774 |
| PTGFR | Prostaglandin F Receptor | Protein Coding | 48 | GC01P078303 | 1.033578515 |
| IL5RA | Interleukin 5 Receptor Subunit Alpha | Protein Coding | 50 | GC03M003066 | 1.033275604 |
| FOXP3 | Forkhead Box P3 | Protein Coding | 49 | GC0XM049250 | 1.033275604 |
| IL9R | Interleukin 9 Receptor | Protein Coding | 41 | GC0XP155997 | 1.033020258 |
| LAP3 | Leucine Aminopeptidase 3 | Protein Coding | 44 | GC04P018747 | 1.03284812 |
| CYBA | Cytochrome B-245 Alpha Chain | Protein Coding | 50 | GC16M088643 | 1.032426834 |
| RHOC | Ras Homolog Family Member C | Protein Coding | 44 | GC01M112701 | 1.031954646 |
| STC1 | Stanniocalcin 1 | Protein Coding | 44 | GC08M023841 | 1.027370691 |
| CASP10 | Caspase 10 | Protein Coding | 50 | GC02P201182 | 1.021890759 |
| TNFAIP3 | TNF Alpha Induced Protein 3 | Protein Coding | 50 | GC06P137866 | 1.021890759 |
| SLC20A1 | Solute Carrier Family 20 Member 1 | Protein Coding | 47 | GC02P123131 | 1.021890759 |
| HSPD1 | Heat Shock Protein Family D (Hsp60) Member 1 | Protein Coding | 51 | GC02M197486 | 1.021381021 |
| GFAP | Glial Fibrillary Acidic Protein | Protein Coding | 50 | GC17M049916 | 1.021381021 |
| MIR181B1 | MicroRNA 181b-1 | RNA Gene | 22 | GC01M198858 | 1.021381021 |
| MAP4K4 | Mitogen-Activated Protein Kinase Kinase Kinase Kinase 4 | Protein Coding | 50 | GC02P101696 | 1.020866752 |
| ADM | Adrenomedullin | Protein Coding | 47 | GC11P010304 | 1.020866752 |
| TNFRSF21 | TNF Receptor Superfamily Member 21 | Protein Coding | 47 | GC06M047231 | 1.020866752 |
| MIR16-2 | MicroRNA 16-2 | RNA Gene | 20 | GC03P160413 | 1.020866752 |
| TMEM123 | Transmembrane Protein 123 | Protein Coding | 37 | GC11M102396 | 1.018704414 |
| LINC02552 | Long Intergenic Non-Protein Coding RNA 2552 | RNA Gene | 13 | GC11M104446 | 1.018704414 |
| ENSG00000256916 | Novel Transcript | RNA Gene | 10 | GC11P102606 | 1.018704414 |
| LOC100421658 | Sacsin Molecular Chaperone Pseudogene | Pseudogene | 7 | GC11M102766 | 1.018704414 |
| BOLA3P1 | BolA Family Member 3 Pseudogene 1 | Pseudogene | 6 | GC11P102880 | 1.018704414 |
| lnc-MMP10-1 | | RNA Gene | 5 | GC11M102768 | 1.018704414 |
| MIR223 | MicroRNA 223 | RNA Gene | 24 | GC0XP066018 | 1.017442465 |
| EFNB1 | Ephrin B1 | Protein Coding | 48 | GC0XP068828 | 1.013970613 |
| PTPRS | Protein Tyrosine Phosphatase Receptor Type S | Protein Coding | 48 | GC19M006113 | 1.013970613 |
| FOXA2 | Forkhead Box A2 | Protein Coding | 47 | GC20M022581 | 1.013970613 |
| ABI1 | Abl Interactor 1 | Protein Coding | 45 | GC10M026746 | 1.013970613 |
| ARFIP1 | ADP Ribosylation Factor Interacting Protein 1 | Protein Coding | 38 | GC04P152779 | 1.013970613 |
| HRH4 | Histamine Receptor H4 | Protein Coding | 48 | GC18P024460 | 1.013764262 |
| SLC17A5 | Solute Carrier Family 17 Member 5 | Protein Coding | 47 | GC06M073593 | 1.013489723 |
| PRG2 | Proteoglycan 2, Pro Eosinophil Major Basic Protein | Protein Coding | 43 | GC11M057386 | 1.012675166 |
| CHGA | Chromogranin A | Protein Coding | 47 | GC14P092947 | 1.012655258 |
| KLF5 | KLF Transcription Factor 5 | Protein Coding | 47 | GC13P073054 | 1.012655258 |
| SAA3P | Serum Amyloid A3, Pseudogene | Pseudogene | 14 | GC11M018112 | 1.012655258 |
| MATN3 | Matrilin 3 | Protein Coding | 45 | GC02M019992 | 1.011874676 |
| CARM1 | Coactivator Associated Arginine Methyltransferase 1 | Protein Coding | 49 | GC19P010871 | 1.009506941 |
| GADD45A | Growth Arrest And DNA Damage Inducible Alpha | Protein Coding | 48 | GC01P067685 | 1.009506941 |
| LGALS8 | Galectin 8 | Protein Coding | 43 | GC01P236518 | 1.009506941 |
| SAFB | Scaffold Attachment Factor B | Protein Coding | 42 | GC19P005623 | 1.009506941 |
| QSOX1 | Quiescin Sulfhydryl Oxidase 1 | Protein Coding | 41 | GC01P180154 | 1.009506941 |
| COL4A3 | Collagen Type IV Alpha 3 Chain | Protein Coding | 49 | GC02P227164 | 1.009225845 |
| IL6ST | Interleukin 6 Cytokine Family Signal Transducer | Protein Coding | 52 | GC05M055935 | 1.004593134 |
| CCNA2 | Cyclin A2 | Protein Coding | 48 | GC04M121816 | 1.004593134 |
| PDGFA | Platelet Derived Growth Factor Subunit A | Protein Coding | 46 | GC07M000497 | 1.004593134 |
| MIR199A2 | MicroRNA 199a-2 | RNA Gene | 23 | GC01M172235 | 1.004593134 |
| MIR199A1 | MicroRNA 199a-1 | RNA Gene | 21 | GC19M010817 | 1.004593134 |
| COL5A1 | Collagen Type V Alpha 1 Chain | Protein Coding | 50 | GC09P134641 | 1.002388477 |
| STIM1 | Stromal Interaction Molecule 1 | Protein Coding | 52 | GC11P003855 | 1.00067389 |
| AHSG | Alpha 2-HS Glycoprotein | Protein Coding | 46 | GC03P186632 | 1.00067389 |
| TUBA1B | Tubulin Alpha 1b | Protein Coding | 46 | GC12M049127 | 1.00067389 |
| H19 | H19 Imprinted Maternally Expressed Transcript | RNA Gene | 30 | GC11M001995 | 1.00067389 |
| MIR210 | MicroRNA 210 | RNA Gene | 23 | GC11M003894 | 1.00067389 |
| PXN | Paxillin | Protein Coding | 47 | GC12M120210 | 0.999838948 |
| KLK1 | Kallikrein 1 | Protein Coding | 49 | GC19M050819 | 0.998813629 |
| NOS1 | Nitric Oxide Synthase 1 | Protein Coding | 51 | GC12M117208 | 0.994827092 |
| NOD2 | Nucleotide Binding Oligomerization Domain Containing 2 | Protein Coding | 50 | GC16P050693 | 0.994827092 |
| CRHR1 | Corticotropin Releasing Hormone Receptor 1 | Protein Coding | 49 | GC17P045784 | 0.994827092 |
| TSLP | Thymic Stromal Lymphopoietin | Protein Coding | 44 | GC05P111070 | 0.994827092 |
| CCR4 | C-C Motif Chemokine Receptor 4 | Protein Coding | 47 | GC03P032951 | 0.989924431 |
| MYCN | MYCN Proto-Oncogene, BHLH Transcription Factor | Protein Coding | 50 | GC02P015949 | 0.989735305 |
| IPO7 | Importin 7 | Protein Coding | 41 | GC11P009384 | 0.989735305 |
| MIR337 | MicroRNA 337 | RNA Gene | 21 | GC14P110434 | 0.989735305 |
| FAP | Fibroblast Activation Protein Alpha | Protein Coding | 48 | GC02M162170 | 0.989513874 |
| STK11 | Serine/Threonine Kinase 11 | Protein Coding | 53 | GC19P001177 | 0.986337483 |
| EIF2AK3 | Eukaryotic Translation Initiation Factor 2 Alpha Kinase 3 | Protein Coding | 51 | GC02M088556 | 0.986337483 |
| NR1H4 | Nuclear Receptor Subfamily 1 Group H Member 4 | Protein Coding | 50 | GC12P100473 | 0.986337483 |
| TLR5 | Toll Like Receptor 5 | Protein Coding | 48 | GC01M223109 | 0.986337483 |
| CAP1 | Cyclase Associated Actin Cytoskeleton Regulatory Protein 1 | Protein Coding | 44 | GC01P040147 | 0.986337483 |
| MIR22 | MicroRNA 22 | RNA Gene | 23 | GC17M001713 | 0.986337483 |
| TP73 | Tumor Protein P73 | Protein Coding | 48 | GC01P003652 | 0.983187854 |
| PLCG1 | Phospholipase C Gamma 1 | Protein Coding | 50 | GC20P041136 | 0.982319713 |
| UBE2N | Ubiquitin Conjugating Enzyme E2 N | Protein Coding | 50 | GC12M093406 | 0.978539526 |
| LY96 | Lymphocyte Antigen 96 | Protein Coding | 45 | GC08P073991 | 0.978539526 |
| MIR34B | MicroRNA 34b | RNA Gene | 23 | GC11P111958 | 0.978539526 |
| ACVR1B | Activin A Receptor Type 1B | Protein Coding | 49 | GC12P051951 | 0.977753997 |
| BMI1 | BMI1 Proto-Oncogene, Polycomb Ring Finger | Protein Coding | 45 | GC10P022326 | 0.977753997 |
| PDLIM7 | PDZ And LIM Domain 7 | Protein Coding | 44 | GC05M177483 | 0.977753997 |
| HOXA10 | Homeobox A10 | Protein Coding | 43 | GC07M027635 | 0.977753997 |
| MIR328 | MicroRNA 328 | RNA Gene | 22 | GC16M067203 | 0.977753997 |
| RNPEP | Arginyl Aminopeptidase | Protein Coding | 43 | GC01P201982 | 0.96765995 |
| REN | Renin | Protein Coding | 51 | GC01M204154 | 0.966400027 |
| EPHA1 | EPH Receptor A1 | Protein Coding | 50 | GC07M143390 | 0.966400027 |
| PROM1 | Prominin 1 | Protein Coding | 48 | GC04M015965 | 0.966400027 |
| HAPLN1 | Hyaluronan And Proteoglycan Link Protein 1 | Protein Coding | 44 | GC05M083637 | 0.966400027 |
| PDPN | Podoplanin | Protein Coding | 44 | GC01P013583 | 0.966400027 |
| INSR | Insulin Receptor | Protein Coding | 56 | GC19M007112 | 0.966144621 |
| HDAC3 | Histone Deacetylase 3 | Protein Coding | 51 | GC05M141583 | 0.966144621 |
| FADD | Fas Associated Via Death Domain | Protein Coding | 48 | GC11P070203 | 0.966144621 |
| MAP3K14 | Mitogen-Activated Protein Kinase Kinase Kinase 14 | Protein Coding | 47 | GC17M045263 | 0.966144621 |
| MAPKAP1 | MAPK Associated Protein 1 | Protein Coding | 45 | GC09M125437 | 0.966144621 |
| FOSL2 | FOS Like 2, AP-1 Transcription Factor Subunit | Protein Coding | 44 | GC02P028392 | 0.966144621 |
| BMAL1 | Basic Helix-Loop-Helix ARNT Like 1 | Protein Coding | 42 | GC11P013277 | 0.966144621 |
| ITLN1 | Intelectin 1 | Protein Coding | 41 | GC01M160876 | 0.966144621 |
| SMTN | Smoothelin | Protein Coding | 40 | GC22P040902 | 0.966144621 |
| TRAPPC10 | Trafficking Protein Particle Complex Subunit 10 | Protein Coding | 40 | GC21P044012 | 0.965832889 |
| ADRA2C | Adrenoceptor Alpha 2C | Protein Coding | 48 | GC04P003766 | 0.962548077 |
| NEFL | Neurofilament Light Chain | Protein Coding | 48 | GC08M024950 | 0.962548077 |
| CBX5 | Chromobox 5 | Protein Coding | 47 | GC12M054230 | 0.962548077 |
| GARS1 | Glycyl-TRNA Synthetase 1 | Protein Coding | 47 | GC07P030580 | 0.962548077 |
| NDRG1 | N-Myc Downstream Regulated 1 | Protein Coding | 48 | GC08M133237 | 0.961836338 |
| PTHLH | Parathyroid Hormone Like Hormone | Protein Coding | 48 | GC12M027959 | 0.956190825 |
| PSEN1 | Presenilin 1 | Protein Coding | 55 | GC14P073136 | 0.955462456 |
| IL10RA | Interleukin 10 Receptor Subunit Alpha | Protein Coding | 49 | GC11P117987 | 0.955462456 |
| GJB2 | Gap Junction Protein Beta 2 | Protein Coding | 48 | GC13M020187 | 0.955462456 |
| HMGA2 | High Mobility Group AT-Hook 2 | Protein Coding | 45 | GC12P065824 | 0.955462456 |
| ACER3 | Alkaline Ceramidase 3 | Protein Coding | 43 | GC11P076860 | 0.952873707 |
| GSR | Glutathione-Disulfide Reductase | Protein Coding | 52 | GC08M030678 | 0.951475859 |
| GUSB | Glucuronidase Beta | Protein Coding | 50 | GC07M065960 | 0.951475859 |
| TNK2 | Tyrosine Kinase Non Receptor 2 | Protein Coding | 50 | GC03M195863 | 0.950566769 |
| NCOA2 | Nuclear Receptor Coactivator 2 | Protein Coding | 47 | GC08M070109 | 0.950566769 |
| RARS1 | Arginyl-TRNA Synthetase 1 | Protein Coding | 47 | GC05P168487 | 0.950566769 |
| SOX18 | SRY-Box Transcription Factor 18 | Protein Coding | 43 | GC20M064047 | 0.950566769 |
| GOLPH3 | Golgi Phosphoprotein 3 | Protein Coding | 41 | GC05M032124 | 0.950566769 |
| RPS9 | Ribosomal Protein S9 | Protein Coding | 44 | GC19P072366 | 0.949136913 |
| SLC2A6 | Solute Carrier Family 2 Member 6 | Protein Coding | 44 | GC09M133471 | 0.949136913 |
| SYMPK | Symplekin Scaffold Protein | Protein Coding | 41 | GC19M045815 | 0.949136913 |
| IFNGR1 | Interferon Gamma Receptor 1 | Protein Coding | 53 | GC06M137197 | 0.948144257 |
| NPR1 | Natriuretic Peptide Receptor 1 | Protein Coding | 50 | GC01P153898 | 0.948144257 |
| CTSA | Cathepsin A | Protein Coding | 48 | GC20P045890 | 0.948144257 |
| CDH15 | Cadherin 15 | Protein Coding | 47 | GC16P089171 | 0.948144257 |
| COL14A1 | Collagen Type XIV Alpha 1 Chain | Protein Coding | 45 | GC08P120109 | 0.948144257 |
| TFAP2C | Transcription Factor AP-2 Gamma | Protein Coding | 44 | GC20P056629 | 0.948144257 |
| DCUN1D1 | Defective In Cullin Neddylation 1 Domain Containing 1 | Protein Coding | 41 | GC03M182938 | 0.948144257 |
| ANGPTL2 | Angiopoietin Like 2 | Protein Coding | 40 | GC09M127087 | 0.948144257 |
| RLN1 | Relaxin 1 | Protein Coding | 40 | GC09M005334 | 0.948144257 |
| MIR130B | MicroRNA 130b | RNA Gene | 20 | GC22P043541 | 0.948144257 |
| WT1 | WT1 Transcription Factor | Protein Coding | 51 | GC11M032365 | 0.946888566 |
| VCL | Vinculin | Protein Coding | 50 | GC10P073995 | 0.946660638 |
| PKHD1 | PKHD1 Ciliary IPT Domain Containing Fibrocystin/Polyductin | Protein Coding | 41 | GC06M069563 | 0.94516176 |
| PTPRC | Protein Tyrosine Phosphatase Receptor Type C | Protein Coding | 54 | GC01P198607 | 0.943224728 |
| NFATC1 | Nuclear Factor Of Activated T Cells 1 | Protein Coding | 50 | GC18P079395 | 0.943224728 |
| MSR1 | Macrophage Scavenger Receptor 1 | Protein Coding | 48 | GC08M016107 | 0.943224728 |
| CDX2 | Caudal Type Homeobox 2 | Protein Coding | 45 | GC13M027962 | 0.943224728 |
| ITGB8 | Integrin Subunit Beta 8 | Protein Coding | 45 | GC07P020329 | 0.943224728 |
| CRABP1 | Cellular Retinoic Acid Binding Protein 1 | Protein Coding | 44 | GC15P078340 | 0.943224728 |
| SRGN | Serglycin | Protein Coding | 43 | GC10P069088 | 0.943224728 |
| RAB37 | RAB37, Member RAS Oncogene Family | Protein Coding | 40 | GC17P074671 | 0.943224728 |
| CSN2 | Casein Beta | Protein Coding | 36 | GC04M069955 | 0.943224728 |
| ENO1 | Enolase 1 | Protein Coding | 50 | GC01M008861 | 0.939836681 |
| MIR203A | MicroRNA 203a | RNA Gene | 23 | GC14P110181 | 0.939836681 |
| CCL17 | C-C Motif Chemokine Ligand 17 | Protein Coding | 45 | GC16P057415 | 0.938501894 |
| GPER1 | G Protein-Coupled Estrogen Receptor 1 | Protein Coding | 45 | GC07P002158 | 0.936646938 |
| CD82 | CD82 Molecule | Protein Coding | 45 | GC11P044586 | 0.930853963 |
| LCK | LCK Proto-Oncogene, Src Family Tyrosine Kinase | Protein Coding | 55 | GC01P032251 | 0.928555965 |
| CFTR | CF Transmembrane Conductance Regulator | Protein Coding | 54 | GC07P117287 | 0.928555965 |
| ARRB2 | Arrestin Beta 2 | Protein Coding | 48 | GC17P004711 | 0.928555965 |
| FGFR3 | Fibroblast Growth Factor Receptor 3 | Protein Coding | 58 | GC04P001795 | 0.927951455 |
| IL18RAP | Interleukin 18 Receptor Accessory Protein | Protein Coding | 44 | GC02P102418 | 0.927951455 |
| RSF1 | Remodeling And Spacing Factor 1 | Protein Coding | 37 | GC11M097504 | 0.927951455 |
| TERC | Telomerase RNA Component | RNA Gene | 31 | GC03M169765 | 0.927951455 |
| MIR590 | MicroRNA 590 | RNA Gene | 22 | GC07P074191 | 0.927951455 |
| TXNRD1 | Thioredoxin Reductase 1 | Protein Coding | 48 | GC12P104215 | 0.92711699 |
| MZF1 | Myeloid Zinc Finger 1 | Protein Coding | 40 | GC19M068619 | 0.92711699 |
| MIR29B2 | MicroRNA 29b-2 | RNA Gene | 20 | GC01M207806 | 0.92711699 |
| NDRG2 | NDRG Family Member 2 | Protein Coding | 40 | GC14M021016 | 0.923687577 |
| CFI | Complement Factor I | Protein Coding | 50 | GC04M109732 | 0.923048794 |
| MAP3K11 | Mitogen-Activated Protein Kinase Kinase Kinase 11 | Protein Coding | 50 | GC11M097060 | 0.923048794 |
| ANGPTL1 | Angiopoietin Like 1 | Protein Coding | 43 | GC01M178818 | 0.923048794 |
| STK4 | Serine/Threonine Kinase 4 | Protein Coding | 52 | GC20P044966 | 0.922720134 |
| MRPL32 | Mitochondrial Ribosomal Protein L32 | Protein Coding | 38 | GC07P042933 | 0.922720134 |
| ITGAL | Integrin Subunit Alpha L | Protein Coding | 50 | GC16P030472 | 0.920246184 |
| IL12RB1 | Interleukin 12 Receptor Subunit Beta 1 | Protein Coding | 48 | GC19M018058 | 0.920246184 |
| MUC5B | Mucin 5B, Oligomeric Mucus/Gel-Forming | Protein Coding | 44 | GC11P001990 | 0.920246184 |
| LNPEP | Leucyl And Cystinyl Aminopeptidase | Protein Coding | 48 | GC05P096935 | 0.918715894 |
| CSF1R | Colony Stimulating Factor 1 Receptor | Protein Coding | 55 | GC05M150053 | 0.91603744 |
| FBN2 | Fibrillin 2 | Protein Coding | 44 | GC05M128257 | 0.91603744 |
| TLN1 | Talin 1 | Protein Coding | 44 | GC09M035696 | 0.91603744 |
| COG2 | Component Of Oligomeric Golgi Complex 2 | Protein Coding | 43 | GC01P230642 | 0.91603744 |
| ITGA10 | Integrin Subunit Alpha 10 | Protein Coding | 41 | GC01M145891 | 0.91603744 |
| ARL6IP5 | ADP Ribosylation Factor Like GTPase 6 Interacting Protein 5 | Protein Coding | 38 | GC03P069084 | 0.91603744 |
| MIR675 | MicroRNA 675 | RNA Gene | 22 | GC11M003991 | 0.91603744 |
| MIR410 | MicroRNA 410 | RNA Gene | 19 | GC14P110447 | 0.91603744 |
| TSC2 | TSC Complex Subunit 2 | Protein Coding | 51 | GC16P012688 | 0.915135622 |
| SOCS2 | Suppressor Of Cytokine Signaling 2 | Protein Coding | 46 | GC12P093569 | 0.915135622 |
| VAV2 | Vav Guanine Nucleotide Exchange Factor 2 | Protein Coding | 45 | GC09M133761 | 0.915135622 |
| AQP5 | Aquaporin 5 | Protein Coding | 49 | GC12P049961 | 0.909288824 |
| CCND2 | Cyclin D2 | Protein Coding | 51 | GC12P022312 | 0.905031502 |
| CSTB | Cystatin B | Protein Coding | 50 | GC21M043772 | 0.905031502 |
| PLOD3 | Procollagen-Lysine,2-Oxoglutarate 5-Dioxygenase 3 | Protein Coding | 49 | GC07M101205 | 0.905031502 |
| GPX1 | Glutathione Peroxidase 1 | Protein Coding | 48 | GC03M051406 | 0.905031502 |
| GAL | Galanin And GMAP Prepropeptide | Protein Coding | 47 | GC11P070671 | 0.905031502 |
| MYOF | Myoferlin | Protein Coding | 47 | GC10M093306 | 0.905031502 |
| S100A10 | S100 Calcium Binding Protein A10 | Protein Coding | 47 | GC01M152488 | 0.905031502 |
| ACTA2 | Actin Alpha 2, Smooth Muscle | Protein Coding | 50 | GC10M088935 | 0.904793084 |
| F8 | Coagulation Factor VIII | Protein Coding | 49 | GC0XM154835 | 0.904793084 |
| NTSR1 | Neurotensin Receptor 1 | Protein Coding | 47 | GC20P062708 | 0.904793084 |
| ADCY10 | Adenylate Cyclase 10 | Protein Coding | 46 | GC01M167809 | 0.904793084 |
| BACH1 | BTB Domain And CNC Homolog 1 | Protein Coding | 44 | GC21P029194 | 0.904793084 |
| CA2 | Carbonic Anhydrase 2 | Protein Coding | 55 | GC08P085463 | 0.90403986 |
| CLCA1 | Chloride Channel Accessory 1 | Protein Coding | 43 | GC01P086468 | 0.901368737 |
| IFNGR2 | Interferon Gamma Receptor 2 | Protein Coding | 49 | GC21P033402 | 0.900053322 |
| IL12RB2 | Interleukin 12 Receptor Subunit Beta 2 | Protein Coding | 47 | GC01P067307 | 0.900053322 |
| IL19 | Interleukin 19 | Protein Coding | 43 | GC01P206770 | 0.900053322 |
| IL31 | Interleukin 31 | Protein Coding | 41 | GC12M122391 | 0.900053322 |
| APLP2 | Amyloid Beta Precursor Like Protein 2 | Protein Coding | 45 | GC11P130069 | 0.895532906 |
| NUAK1 | NUAK Family Kinase 1 | Protein Coding | 45 | GC12M106063 | 0.89461863 |
| ITGA6 | Integrin Subunit Alpha 6 | Protein Coding | 52 | GC02P172427 | 0.88905704 |
| EDN2 | Endothelin 2 | Protein Coding | 45 | GC01M041478 | 0.888414145 |
| PRKCI | Protein Kinase C Iota | Protein Coding | 50 | GC03P170222 | 0.884600222 |
| CD74 | CD74 Molecule | Protein Coding | 47 | GC05M150378 | 0.884600222 |
| HNRNPK | Heterogeneous Nuclear Ribonucleoprotein K | Protein Coding | 47 | GC09M095069 | 0.884600222 |
| NTF3 | Neurotrophin 3 | Protein Coding | 47 | GC12P022322 | 0.884600222 |
| GPT | Glutamic--Pyruvic Transaminase | Protein Coding | 44 | GC08P144502 | 0.884600222 |
| CSRP2 | Cysteine And Glycine Rich Protein 2 | Protein Coding | 43 | GC12M076859 | 0.884600222 |
| COL8A1 | Collagen Type VIII Alpha 1 Chain | Protein Coding | 42 | GC03P099638 | 0.884600222 |
| CREB3 | CAMP Responsive Element Binding Protein 3 | Protein Coding | 42 | GC09P048090 | 0.884600222 |
| LORICRIN | Loricrin Cornified Envelope Precursor Protein | Protein Coding | 35 | GC01P153262 | 0.884600222 |
| MIR451A | MicroRNA 451a | RNA Gene | 20 | GC17M028861 | 0.884600222 |
| S100A7 | S100 Calcium Binding Protein A7 | Protein Coding | 43 | GC01M153457 | 0.883220553 |
| NECTIN2 | Nectin Cell Adhesion Molecule 2 | Protein Coding | 46 | GC19P069965 | 0.882121742 |
| GPR37L1 | G Protein-Coupled Receptor 37 Like 1 | Protein Coding | 44 | GC01P202122 | 0.882121742 |
| DDX31 | DEAD-Box Helicase 31 | Protein Coding | 41 | GC09M132594 | 0.882121742 |
| PPP1R12B | Protein Phosphatase 1 Regulatory Subunit 12B | Protein Coding | 41 | GC01P202348 | 0.882121742 |
| SURF4 | Surfeit 4 | Protein Coding | 40 | GC09M133361 | 0.882121742 |
| TTF1 | Transcription Termination Factor 1 | Protein Coding | 40 | GC09M132375 | 0.882121742 |
| FBXO46 | F-Box Protein 46 | Protein Coding | 31 | GC19M045710 | 0.882121742 |
| PODXL | Podocalyxin Like | Protein Coding | 45 | GC07M131500 | 0.880606353 |
| TES | Testin LIM Domain Protein | Protein Coding | 42 | GC07P116210 | 0.880606353 |
| FLT4 | Fms Related Receptor Tyrosine Kinase 4 | Protein Coding | 55 | GC05M180614 | 0.879651427 |
| MYB | MYB Proto-Oncogene, Transcription Factor | Protein Coding | 51 | GC06P135180 | 0.877844274 |
| BTRC | Beta-Transducin Repeat Containing E3 Ubiquitin Protein Ligase | Protein Coding | 48 | GC10P101354 | 0.877844274 |
| KPNA2 | Karyopherin Subunit Alpha 2 | Protein Coding | 47 | GC17P068035 | 0.877844274 |
| PBK | PDZ Binding Kinase | Protein Coding | 47 | GC08M027809 | 0.877844274 |
| CDH17 | Cadherin 17 | Protein Coding | 45 | GC08M094127 | 0.877844274 |
| VPS4B | Vacuolar Protein Sorting 4 Homolog B | Protein Coding | 43 | GC18M063389 | 0.877844274 |
| BOK | BCL2 Family Apoptosis Regulator BOK | Protein Coding | 42 | GC02P241558 | 0.877844274 |
| UCN | Urocortin | Protein Coding | 38 | GC02M027308 | 0.877844274 |
| MIR193B | MicroRNA 193b | RNA Gene | 23 | GC16P014307 | 0.877844274 |
| MIR346 | MicroRNA 346 | RNA Gene | 22 | GC10M086264 | 0.877844274 |
| MIR582 | MicroRNA 582 | RNA Gene | 22 | GC05M059703 | 0.877844274 |
| C3AR1 | Complement C3a Receptor 1 | Protein Coding | 45 | GC12M008058 | 0.877133429 |
| RNASE3 | Ribonuclease A Family Member 3 | Protein Coding | 42 | GC14P020891 | 0.877133429 |
| RUNX3 | RUNX Family Transcription Factor 3 | Protein Coding | 46 | GC01M024899 | 0.875854433 |
| L1CAM | L1 Cell Adhesion Molecule | Protein Coding | 50 | GC0XM153864 | 0.871626258 |
| EDN3 | Endothelin 3 | Protein Coding | 48 | GC20P059300 | 0.871626258 |
| FBLN1 | Fibulin 1 | Protein Coding | 47 | GC22P045502 | 0.871626258 |
| SCARB2 | Scavenger Receptor Class B Member 2 | Protein Coding | 48 | GC04M076158 | 0.869389355 |
| LGR6 | Leucine Rich Repeat Containing G Protein-Coupled Receptor 6 | Protein Coding | 44 | GC01P202193 | 0.868489385 |
| SOX9 | SRY-Box Transcription Factor 9 | Protein Coding | 48 | GC17P072121 | 0.867881358 |
| A1BG | Alpha-1-B Glycoprotein | Protein Coding | 41 | GC19M058345 | 0.865581691 |
| PEPD | Peptidase D | Protein Coding | 48 | GC19M033386 | 0.864304721 |
| ATM | ATM Serine/Threonine Kinase | Protein Coding | 55 | GC11P108222 | 0.861680329 |
| PKD2 | Polycystin 2, Transient Receptor Potential Cation Channel | Protein Coding | 50 | GC04P088007 | 0.861680329 |
| CEBPA | CCAAT Enhancer Binding Protein Alpha | Protein Coding | 49 | GC19M033299 | 0.861680329 |
| CD59 | CD59 Molecule (CD59 Blood Group) | Protein Coding | 48 | GC11M033704 | 0.861680329 |
| CPOX | Coproporphyrinogen Oxidase | Protein Coding | 47 | GC03M098576 | 0.861680329 |
| IQGAP1 | IQ Motif Containing GTPase Activating Protein 1 | Protein Coding | 46 | GC15P090388 | 0.861680329 |
| GNA13 | G Protein Subunit Alpha 13 | Protein Coding | 45 | GC17M065009 | 0.861680329 |
| AMFR | Autocrine Motility Factor Receptor | Protein Coding | 44 | GC16M056361 | 0.861680329 |
| FGF18 | Fibroblast Growth Factor 18 | Protein Coding | 44 | GC05P171419 | 0.861680329 |
| HERPUD1 | Homocysteine Inducible ER Protein With Ubiquitin Like Domain 1 | Protein Coding | 41 | GC16P056932 | 0.861680329 |
| DUSP19 | Dual Specificity Phosphatase 19 | Protein Coding | 40 | GC02P183078 | 0.861680329 |
| ULBP1 | UL16 Binding Protein 1 | Protein Coding | 42 | GC06P149963 | 0.860101819 |
| ULBP3 | UL16 Binding Protein 3 | Protein Coding | 40 | GC06M150062 | 0.860101819 |
| SLC9A1 | Solute Carrier Family 9 Member A1 | Protein Coding | 53 | GC01M027176 | 0.858342171 |
| MALAT1 | Metastasis Associated Lung Adenocarcinoma Transcript 1 | RNA Gene | 26 | GC11P070433 | 0.856680751 |
| DLL4 | Delta Like Canonical Notch Ligand 4 | Protein Coding | 50 | GC15P040929 | 0.853370547 |
| WWOX | WW Domain Containing Oxidoreductase | Protein Coding | 48 | GC16P078099 | 0.853370547 |
| CSTA | Cystatin A | Protein Coding | 47 | GC03P122325 | 0.853370547 |
| TRAF1 | TNF Receptor Associated Factor 1 | Protein Coding | 44 | GC09M120902 | 0.853370547 |
| FYN | FYN Proto-Oncogene, Src Family Tyrosine Kinase | Protein Coding | 49 | GC06M111660 | 0.849946201 |
| ALOX15 | Arachidonate 15-Lipoxygenase | Protein Coding | 48 | GC17M004630 | 0.849946201 |
| CYSLTR1 | Cysteinyl Leukotriene Receptor 1 | Protein Coding | 48 | GC0XM078271 | 0.849946201 |
| ALOX12 | Arachidonate 12-Lipoxygenase, 12S Type | Protein Coding | 47 | GC17P006995 | 0.849946201 |
| CCL26 | C-C Motif Chemokine Ligand 26 | Protein Coding | 43 | GC07M075769 | 0.849946201 |
| PDGFB | Platelet Derived Growth Factor Subunit B | Protein Coding | 53 | GC22M060099 | 0.848274827 |
| ASIC1 | Acid Sensing Ion Channel Subunit 1 | Protein Coding | 47 | GC12P050057 | 0.842413187 |
| THY1 | Thy-1 Cell Surface Antigen | Protein Coding | 46 | GC11M119417 | 0.842413187 |
| TNFSF15 | TNF Superfamily Member 15 | Protein Coding | 46 | GC09M114784 | 0.842413187 |
| LASP1 | LIM And SH3 Protein 1 | Protein Coding | 44 | GC17P038869 | 0.842413187 |
| MATR3 | Matrin 3 | Protein Coding | 44 | GC05P139274 | 0.842413187 |
| MFAP2 | Microfibril Associated Protein 2 | Protein Coding | 44 | GC01M016974 | 0.842413187 |
| PADI2 | Peptidyl Arginine Deiminase 2 | Protein Coding | 44 | GC01M017066 | 0.842413187 |
| VTI1B | Vesicle Transport Through Interaction With T-SNAREs 1B | Protein Coding | 43 | GC14M067647 | 0.842413187 |
| ASH1L | ASH1 Like Histone Lysine Methyltransferase | Protein Coding | 41 | GC01M155335 | 0.842413187 |
| MT3 | Metallothionein 3 | Protein Coding | 40 | GC16P056589 | 0.842413187 |
| STX12 | Syntaxin 12 | Protein Coding | 40 | GC01P028303 | 0.842413187 |
| TMBIM6 | Transmembrane BAX Inhibitor Motif Containing 6 | Protein Coding | 40 | GC12P049707 | 0.842413187 |
| GSTT1 | Glutathione S-Transferase Theta 1 | Protein Coding | 34 | GC22Mi00270 | 0.842413187 |
| MIR182 | MicroRNA 182 | RNA Gene | 23 | GC07M129770 | 0.842413187 |
| IL17C | Interleukin 17C | Protein Coding | 40 | GC16P088638 | 0.841578722 |
| KNTC1 | Kinetochore Associated 1 | Protein Coding | 37 | GC12P122527 | 0.841578722 |
| CBY1 | Chibby Family Member 1, Beta Catenin Antagonist | Protein Coding | 41 | GC22P038656 | 0.841302156 |
| SPHK1 | Sphingosine Kinase 1 | Protein Coding | 49 | GC17P076376 | 0.839953005 |
| GPNMB | Glycoprotein Nmb | Protein Coding | 47 | GC07P023238 | 0.839953005 |
| FOXCUT | FOXC1 Upstream Transcript | RNA Gene | 15 | GC06P001604 | 0.836130857 |
| AIFM1 | Apoptosis Inducing Factor Mitochondria Associated 1 | Protein Coding | 51 | GC0XM130129 | 0.834493041 |
| GATA2 | GATA Binding Protein 2 | Protein Coding | 51 | GC03M128479 | 0.834493041 |
| RIPK1 | Receptor Interacting Serine/Threonine Kinase 1 | Protein Coding | 51 | GC06P003313 | 0.834493041 |
| PTPRA | Protein Tyrosine Phosphatase Receptor Type A | Protein Coding | 48 | GC20P002864 | 0.834493041 |
| S1PR3 | Sphingosine-1-Phosphate Receptor 3 | Protein Coding | 48 | GC09P089414 | 0.834493041 |
| CDKN2C | Cyclin Dependent Kinase Inhibitor 2C | Protein Coding | 47 | GC01P050960 | 0.834493041 |
| NAA10 | N-Alpha-Acetyltransferase 10, NatA Catalytic Subunit | Protein Coding | 47 | GC0XM153929 | 0.834493041 |
| RAC3 | Rac Family Small GTPase 3 | Protein Coding | 47 | GC17P082031 | 0.834493041 |
| EXOC4 | Exocyst Complex Component 4 | Protein Coding | 43 | GC07P133253 | 0.834493041 |
| RAP1GAP | RAP1 GTPase Activating Protein | Protein Coding | 42 | GC01M021596 | 0.834493041 |
| KLF2 | KLF Transcription Factor 2 | Protein Coding | 41 | GC19P069290 | 0.834493041 |
| LRP10 | LDL Receptor Related Protein 10 | Protein Coding | 41 | GC14P022871 | 0.834493041 |
| SEPTIN9 | Septin 9 | Protein Coding | 40 | GC17P077282 | 0.834493041 |
| ODAM | Odontogenic, Ameloblast Associated | Protein Coding | 37 | GC04P070195 | 0.834493041 |
| FABP12 | Fatty Acid Binding Protein 12 | Protein Coding | 36 | GC08M081524 | 0.834493041 |
| MIR101-1 | MicroRNA 101-1 | RNA Gene | 22 | GC01M065058 | 0.834493041 |
| FOLH1 | Folate Hydrolase 1 | Protein Coding | 50 | GC11M096736 | 0.833177686 |
| HSP90B1 | Heat Shock Protein 90 Beta Family Member 1 | Protein Coding | 50 | GC12P103930 | 0.833177686 |
| SETD2 | SET Domain Containing 2, Histone Lysine Methyltransferase | Protein Coding | 50 | GC03M047033 | 0.833177686 |
| CFP | Complement Factor Properdin | Protein Coding | 45 | GC0XM047624 | 0.833177686 |
| NUDT6 | Nudix Hydrolase 6 | Protein Coding | 40 | GC04M122888 | 0.833177686 |
| MIR15A | MicroRNA 15a | RNA Gene | 19 | GC13M050049 | 0.833177686 |
| HLA-B | Major Histocompatibility Complex, Class I, B | Protein Coding | 48 | GC06M069166 | 0.833038151 |
| MIR93 | MicroRNA 93 | RNA Gene | 23 | GC07M102066 | 0.832906365 |
| APC | APC Regulator Of WNT Signaling Pathway | Protein Coding | 51 | GC05P112707 | 0.825276971 |
| PDE4D | Phosphodiesterase 4D | Protein Coding | 51 | GC05M058969 | 0.823750615 |
| PMEL | Premelanosome Protein | Protein Coding | 44 | GC12M055954 | 0.822299719 |
| PTPRZ1 | Protein Tyrosine Phosphatase Receptor Type Z1 | Protein Coding | 48 | GC07P121873 | 0.821398973 |
| XBP1 | X-Box Binding Protein 1 | Protein Coding | 47 | GC22M028794 | 0.821398973 |
| PTN | Pleiotrophin | Protein Coding | 44 | GC07M137227 | 0.821398973 |
| CIB1 | Calcium And Integrin Binding 1 | Protein Coding | 43 | GC15M090229 | 0.821398973 |
| GP5 | Glycoprotein V Platelet | Protein Coding | 43 | GC03M194395 | 0.821398973 |
| H3-4 | H3.4 Histone, Cluster Member | Protein Coding | 38 | GC01M228427 | 0.821398973 |
| LTC4S | Leukotriene C4 Synthase | Protein Coding | 45 | GC05P179793 | 0.814515114 |
| CD69 | CD69 Molecule | Protein Coding | 44 | GC12M021836 | 0.814515114 |
| ORMDL3 | ORMDL Sphingolipid Biosynthesis Regulator 3 | Protein Coding | 41 | GC17M039921 | 0.814515114 |
| PRNP | Prion Protein | Protein Coding | 51 | GC20P004686 | 0.810257792 |
| IFNAR1 | Interferon Alpha And Beta Receptor Subunit 1 | Protein Coding | 50 | GC21P033324 | 0.810257792 |
| DVL2 | Dishevelled Segment Polarity Protein 2 | Protein Coding | 47 | GC17M007225 | 0.810257792 |
| LTB | Lymphotoxin Beta | Protein Coding | 45 | GC06M069210 | 0.810257792 |
| TICAM1 | TIR Domain Containing Adaptor Molecule 1 | Protein Coding | 45 | GC19M004815 | 0.810257792 |
| TSPAN12 | Tetraspanin 12 | Protein Coding | 45 | GC07M120787 | 0.810257792 |
| DSG3 | Desmoglein 3 | Protein Coding | 44 | GC18P031447 | 0.810257792 |
| AP1G1 | Adaptor Related Protein Complex 1 Subunit Gamma 1 | Protein Coding | 43 | GC16M071729 | 0.810257792 |
| CD200 | CD200 Molecule | Protein Coding | 43 | GC03P112332 | 0.810257792 |
| E2F3 | E2F Transcription Factor 3 | Protein Coding | 43 | GC06P020402 | 0.810257792 |
| PAWR | Pro-Apoptotic WT1 Regulator | Protein Coding | 43 | GC12M079574 | 0.810257792 |
| GGA2 | Golgi Associated, Gamma Adaptin Ear Containing, ARF Binding Protein 2 | Protein Coding | 40 | GC16M023464 | 0.810257792 |
| KLF9 | KLF Transcription Factor 9 | Protein Coding | 40 | GC09M070384 | 0.810257792 |
| MIA | MIA SH3 Domain Containing | Protein Coding | 40 | GC19P040771 | 0.810257792 |
| ZNF384 | Zinc Finger Protein 384 | Protein Coding | 40 | GC12M006934 | 0.810257792 |
| TPT1 | Tumor Protein, Translationally-Controlled 1 | Protein Coding | 48 | GC13M045333 | 0.810118258 |
| MIR195 | MicroRNA 195 | RNA Gene | 23 | GC17M007018 | 0.807934642 |
| RPL7A | Ribosomal Protein L7a | Protein Coding | 44 | GC09P133348 | 0.805871069 |
| GPSM3 | G Protein Signaling Modulator 3 | Protein Coding | 36 | GC06M069237 | 0.805871069 |
| MIR140 | MicroRNA 140 | RNA Gene | 26 | GC16P069934 | 0.800905824 |
| SERPINF1 | Serpin Family F Member 1 | Protein Coding | 48 | GC17P001761 | 0.799761176 |
| CCNE1 | Cyclin E1 | Protein Coding | 51 | GC19P029811 | 0.799061954 |
| PTPRJ | Protein Tyrosine Phosphatase Receptor Type J | Protein Coding | 51 | GC11P048002 | 0.799061954 |
| KMT2A | Lysine Methyltransferase 2A | Protein Coding | 50 | GC11P118436 | 0.799061954 |
| P2RY1 | Purinergic Receptor P2Y1 | Protein Coding | 50 | GC03P152835 | 0.799061954 |
| ASCL1 | Achaete-Scute Family BHLH Transcription Factor 1 | Protein Coding | 48 | GC12P102957 | 0.799061954 |
| LMAN1 | Lectin, Mannose Binding 1 | Protein Coding | 48 | GC18M059327 | 0.799061954 |
| PDE5A | Phosphodiesterase 5A | Protein Coding | 48 | GC04M119494 | 0.799061954 |
| APOC2 | Apolipoprotein C2 | Protein Coding | 46 | GC19P069971 | 0.799061954 |
| CIITA | Class II Major Histocompatibility Complex Transactivator | Protein Coding | 46 | GC16P012924 | 0.799061954 |
| RPS3 | Ribosomal Protein S3 | Protein Coding | 46 | GC11P078206 | 0.799061954 |
| KLK7 | Kallikrein Related Peptidase 7 | Protein Coding | 44 | GC19M068278 | 0.799061954 |
| SP7 | Sp7 Transcription Factor | Protein Coding | 44 | GC12M053326 | 0.799061954 |
| KIF13A | Kinesin Family Member 13A | Protein Coding | 43 | GC06M017759 | 0.799061954 |
| ACR | Acrosin | Protein Coding | 41 | GC22P050738 | 0.799061954 |
| NUPR1 | Nuclear Protein 1, Transcriptional Regulator | Protein Coding | 37 | GC16M028532 | 0.799061954 |
| CASP12 | Caspase 12 (Gene/Pseudogene) | Protein Coding | 36 | GC11M104885 | 0.799061954 |
| PLA2R1 | Phospholipase A2 Receptor 1 | Protein Coding | 42 | GC02M159924 | 0.797005177 |
| ERVMER34-1 | Endogenous Retrovirus Group MER34 Member 1, Envelope | Protein Coding | 31 | GC04M052722 | 0.797005177 |
| RARB | Retinoic Acid Receptor Beta | Protein Coding | 51 | GC03P024689 | 0.796628058 |
| CADM1 | Cell Adhesion Molecule 1 | Protein Coding | 46 | GC11M115169 | 0.795068145 |
| ABCA1 | ATP Binding Cassette Subfamily A Member 1 | Protein Coding | 50 | GC09M104781 | 0.793207884 |
| ZEB1 | Zinc Finger E-Box Binding Homeobox 1 | Protein Coding | 51 | GC10P031318 | 0.792863369 |
| HTRA1 | HtrA Serine Peptidase 1 | Protein Coding | 48 | GC10P122461 | 0.786766231 |
| HLA-A | Major Histocompatibility Complex, Class I, A | Protein Coding | 48 | GC06P087685 | 0.786355376 |
| PTPRU | Protein Tyrosine Phosphatase Receptor Type U | Protein Coding | 45 | GC01P029236 | 0.786355376 |
| GPRC6A | G Protein-Coupled Receptor Class C Group 6 Member A | Protein Coding | 40 | GC06M116793 | 0.786355376 |
| RAB27A | RAB27A, Member RAS Oncogene Family | Protein Coding | 51 | GC15M055202 | 0.783070505 |
| AQP4 | Aquaporin 4 | Protein Coding | 50 | GC18M026852 | 0.783070505 |
| MAG | Myelin Associated Glycoprotein | Protein Coding | 50 | GC19P035292 | 0.783070505 |
| MAP3K5 | Mitogen-Activated Protein Kinase Kinase Kinase 5 | Protein Coding | 50 | GC06M136557 | 0.783070505 |
| AQP3 | Aquaporin 3 (Gill Blood Group) | Protein Coding | 49 | GC09M033431 | 0.783070505 |
| TARDBP | TAR DNA Binding Protein | Protein Coding | 49 | GC01P011013 | 0.783070505 |
| GALNT3 | Polypeptide N-Acetylgalactosaminyltransferase 3 | Protein Coding | 48 | GC02M165747 | 0.783070505 |
| NR1I2 | Nuclear Receptor Subfamily 1 Group I Member 2 | Protein Coding | 47 | GC03P119780 | 0.783070505 |
| PGRMC1 | Progesterone Receptor Membrane Component 1 | Protein Coding | 47 | GC0XP119236 | 0.783070505 |
| UBE2T | Ubiquitin Conjugating Enzyme E2 T | Protein Coding | 47 | GC01M202332 | 0.783070505 |
| CNGA3 | Cyclic Nucleotide Gated Channel Subunit Alpha 3 | Protein Coding | 45 | GC02P098329 | 0.783070505 |
| CNGB1 | Cyclic Nucleotide Gated Channel Subunit Beta 1 | Protein Coding | 45 | GC16M057884 | 0.783070505 |
| F11R | F11 Receptor | Protein Coding | 45 | GC01M160995 | 0.783070505 |
| CNGB3 | Cyclic Nucleotide Gated Channel Subunit Beta 3 | Protein Coding | 44 | GC08M086553 | 0.783070505 |
| POU1F1 | POU Class 1 Homeobox 1 | Protein Coding | 44 | GC03M087259 | 0.783070505 |
| SH2B1 | SH2B Adaptor Protein 1 | Protein Coding | 44 | GC16P041757 | 0.783070505 |
| UBE2V1 | Ubiquitin Conjugating Enzyme E2 V1 | Protein Coding | 44 | GC20M050082 | 0.783070505 |
| CAPN7 | Calpain 7 | Protein Coding | 42 | GC03P017222 | 0.783070505 |
| HBP1 | HMG-Box Transcription Factor 1 | Protein Coding | 41 | GC07P107168 | 0.783070505 |
| MED28 | Mediator Complex Subunit 28 | Protein Coding | 40 | GC04P018744 | 0.783070505 |
| H4C16 | H4 Histone 16 | Protein Coding | 39 | GC12M022008 | 0.783070505 |
| ADM2 | Adrenomedullin 2 | Protein Coding | 37 | GC22P050481 | 0.783070505 |
| ETV2 | ETS Variant Transcription Factor 2 | Protein Coding | 37 | GC19P069630 | 0.783070505 |
| MIRLET7A1 | MicroRNA Let-7a-1 | RNA Gene | 23 | GC09P094175 | 0.783070505 |
| MIR483 | MicroRNA 483 | RNA Gene | 22 | GC11M004005 | 0.783070505 |
| SNHG16 | Small Nucleolar RNA Host Gene 16 | RNA Gene | 21 | GC17P077162 | 0.783070505 |
| MIR98 | MicroRNA 98 | RNA Gene | 20 | GC0XM053641 | 0.783070505 |
| KLK11 | Kallikrein Related Peptidase 11 | Protein Coding | 43 | GC19M068282 | 0.78293103 |
| SFTPC | Surfactant Protein C | Protein Coding | 44 | GC08P022156 | 0.780399382 |
| MSLN | Mesothelin | Protein Coding | 45 | GC16P012620 | 0.778531849 |
| PTPRM | Protein Tyrosine Phosphatase Receptor Type M | Protein Coding | 47 | GC18P007557 | 0.774563551 |
| PTH1R | Parathyroid Hormone 1 Receptor | Protein Coding | 52 | GC03P046877 | 0.76823622 |
| THPO | Thrombopoietin | Protein Coding | 48 | GC03M184371 | 0.76823622 |
| CASP6 | Caspase 6 | Protein Coding | 51 | GC04M109688 | 0.766162515 |
| CDC25C | Cell Division Cycle 25C | Protein Coding | 50 | GC05M138285 | 0.766162515 |
| PAX5 | Paired Box 5 | Protein Coding | 48 | GC09M036859 | 0.766162515 |
| EWSR1 | EWS RNA Binding Protein 1 | Protein Coding | 47 | GC22P040495 | 0.766162515 |
| LOXL1 | Lysyl Oxidase Like 1 | Protein Coding | 45 | GC15P073925 | 0.766162515 |
| TNFRSF8 | TNF Receptor Superfamily Member 8 | Protein Coding | 45 | GC01P012063 | 0.766162515 |
| ID3 | Inhibitor Of DNA Binding 3, HLH Protein | Protein Coding | 42 | GC01M023557 | 0.766162515 |
| CCL14 | C-C Motif Chemokine Ligand 14 | Protein Coding | 38 | GC17M046176 | 0.766162515 |
| ELAVL1 | ELAV Like RNA Binding Protein 1 | Protein Coding | 43 | GC19M007958 | 0.762906075 |
| CXCL2 | C-X-C Motif Chemokine Ligand 2 | Protein Coding | 42 | GC04M074097 | 0.759341478 |
| PTGES | Prostaglandin E Synthase | Protein Coding | 44 | GC09M129738 | 0.75392139 |
| CXCR5 | C-X-C Motif Chemokine Receptor 5 | Protein Coding | 45 | GC11P118928 | 0.753560185 |
| E2F1 | E2F Transcription Factor 1 | Protein Coding | 45 | GC20M033675 | 0.753560185 |
| IL22 | Interleukin 22 | Protein Coding | 44 | GC12M068248 | 0.753560185 |
| G6PD | Glucose-6-Phosphate Dehydrogenase | Protein Coding | 53 | GC0XM154558 | 0.747639418 |
| PKN1 | Protein Kinase N1 | Protein Coding | 51 | GC19P014433 | 0.747639418 |
| SLC25A4 | Solute Carrier Family 25 Member 4 | Protein Coding | 51 | GC04P185143 | 0.747639418 |
| TAB2 | TGF-Beta Activated Kinase 1 (MAP3K7) Binding Protein 2 | Protein Coding | 51 | GC06P149218 | 0.747639418 |
| ITGA7 | Integrin Subunit Alpha 7 | Protein Coding | 50 | GC12M055684 | 0.747639418 |
| PIK3C2A | Phosphatidylinositol-4-Phosphate 3-Kinase Catalytic Subunit Type 2 Alpha | Protein Coding | 50 | GC11M017614 | 0.747639418 |
| TGFBR3 | Transforming Growth Factor Beta Receptor 3 | Protein Coding | 50 | GC01M091680 | 0.747639418 |
| NME2 | NME/NM23 Nucleoside Diphosphate Kinase 2 | Protein Coding | 49 | GC17P051165 | 0.747639418 |
| PRKD2 | Protein Kinase D2 | Protein Coding | 49 | GC19M046674 | 0.747639418 |
| ABHD5 | Abhydrolase Domain Containing 5, Lysophosphatidic Acid Acyltransferase | Protein Coding | 48 | GC03P043707 | 0.747639418 |
| HELLS | Helicase, Lymphoid Specific | Protein Coding | 48 | GC10P094546 | 0.747639418 |
| NEDD4L | NEDD4 Like E3 Ubiquitin Protein Ligase | Protein Coding | 48 | GC18P058044 | 0.747639418 |
| CD2 | CD2 Molecule | Protein Coding | 47 | GC01P116754 | 0.747639418 |
| TFAP2B | Transcription Factor AP-2 Beta | Protein Coding | 47 | GC06P088068 | 0.747639418 |
| HDAC10 | Histone Deacetylase 10 | Protein Coding | 46 | GC22M050245 | 0.747639418 |
| IRF9 | Interferon Regulatory Factor 9 | Protein Coding | 46 | GC14P024161 | 0.747639418 |
| JAG2 | Jagged Canonical Notch Ligand 2 | Protein Coding | 46 | GC14M105140 | 0.747639418 |
| PEX2 | Peroxisomal Biogenesis Factor 2 | Protein Coding | 46 | GC08M076980 | 0.747639418 |
| ERLIN1 | ER Lipid Raft Associated 1 | Protein Coding | 45 | GC10M100150 | 0.747639418 |
| YARS1 | Tyrosyl-TRNA Synthetase 1 | Protein Coding | 45 | GC01M032776 | 0.747639418 |
| POT1 | Protection Of Telomeres 1 | Protein Coding | 44 | GC07M124822 | 0.747639418 |
| POLR2L | RNA Polymerase II, I And III Subunit L | Protein Coding | 43 | GC11M003917 | 0.747639418 |
| WWP1 | WW Domain Containing E3 Ubiquitin Protein Ligase 1 | Protein Coding | 43 | GC08P086342 | 0.747639418 |
| CAMLG | Calcium Modulating Ligand | Protein Coding | 42 | GC05P134738 | 0.747639418 |
| USP22 | Ubiquitin Specific Peptidase 22 | Protein Coding | 42 | GC17M020999 | 0.747639418 |
| B3GNT3 | UDP-GlcNAc:BetaGal Beta-1,3-N-Acetylglucosaminyltransferase 3 | Protein Coding | 41 | GC19P017794 | 0.747639418 |
| FERMT2 | FERM Domain Containing Kindlin 2 | Protein Coding | 41 | GC14M052857 | 0.747639418 |
| ZBTB7A | Zinc Finger And BTB Domain Containing 7A | Protein Coding | 40 | GC19M006067 | 0.747639418 |
| TRIM59 | Tripartite Motif Containing 59 | Protein Coding | 38 | GC03M160432 | 0.747639418 |
| DDI1 | DNA Damage Inducible 1 Homolog 1 | Protein Coding | 36 | GC11P104036 | 0.747639418 |
| MIR133A2 | MicroRNA 133a-2 | RNA Gene | 22 | GC20P063406 | 0.747639418 |
| MIR9-1 | MicroRNA 9-1 | RNA Gene | 22 | GC01M156420 | 0.747639418 |
| MIR147B | MicroRNA 147b | RNA Gene | 19 | GC15P045433 | 0.747639418 |
| IGKV2D-29 | Immunoglobulin Kappa Variable 2D-29 | Protein Coding | 14 | GC02P091407 | 0.747639418 |
| LOC107963948 | BCR-ABL Micro-Breakpoint Cluster Region | Biological Region | 2 | GC22P043467 | 0.747639418 |
| LOC107980440 | ABL Breakpoint Recombination Region | Biological Region | 2 | GC09P130710 | 0.747639418 |
| FKBP5 | FKBP Prolyl Isomerase 5 | Protein Coding | 51 | GC06M069345 | 0.747499943 |
| ADORA1 | Adenosine A1 Receptor | Protein Coding | 50 | GC01P203090 | 0.747499943 |
| RORA | RAR Related Orphan Receptor A | Protein Coding | 50 | GC15M060488 | 0.747499943 |
| CYP24A1 | Cytochrome P450 Family 24 Subfamily A Member 1 | Protein Coding | 48 | GC20M054153 | 0.747499943 |
| RORC | RAR Related Orphan Receptor C | Protein Coding | 48 | GC01M151806 | 0.747499943 |
| TNFRSF4 | TNF Receptor Superfamily Member 4 | Protein Coding | 48 | GC01M001211 | 0.747499943 |
| CCL19 | C-C Motif Chemokine Ligand 19 | Protein Coding | 41 | GC09M034703 | 0.747499943 |
| EZR | Ezrin | Protein Coding | 48 | GC06M158765 | 0.746118188 |
| SQSTM1 | Sequestosome 1 | Protein Coding | 51 | GC05P179806 | 0.743242621 |
| IL1RAP | Interleukin 1 Receptor Accessory Protein | Protein Coding | 48 | GC03P190514 | 0.743242621 |
| LBP | Lipopolysaccharide Binding Protein | Protein Coding | 47 | GC20P038346 | 0.743242621 |
| NNMT | Nicotinamide N-Methyltransferase | Protein Coding | 47 | GC11P114257 | 0.743242621 |
| SEMA3C | Semaphorin 3C | Protein Coding | 47 | GC07M080742 | 0.743242621 |
| IGFBP6 | Insulin Like Growth Factor Binding Protein 6 | Protein Coding | 46 | GC12P053097 | 0.743242621 |
| SFTPA1 | Surfactant Protein A1 | Protein Coding | 46 | GC10P094450 | 0.743242621 |
| GGT7 | Gamma-Glutamyltransferase 7 | Protein Coding | 43 | GC20M034844 | 0.743242621 |
| LMLN | Leishmanolysin Like Peptidase | Protein Coding | 37 | GC03P197961 | 0.743242621 |
| MYDGF | Myeloid Derived Growth Factor | Protein Coding | 37 | GC19M004641 | 0.743242621 |
| TSPO2 | Translocator Protein 2 | Protein Coding | 35 | GC06P041041 | 0.743242621 |
| ERBB3 | Erb-B2 Receptor Tyrosine Kinase 3 | Protein Coding | 55 | GC12P057357 | 0.737934411 |
| LAMB1 | Laminin Subunit Beta 1 | Protein Coding | 52 | GC07M107923 | 0.737934411 |
| TNFRSF12A | TNF Receptor Superfamily Member 12A | Protein Coding | 44 | GC16P003018 | 0.737934411 |
| LTBP1 | Latent Transforming Growth Factor Beta Binding Protein 1 | Protein Coding | 48 | GC02P032946 | 0.736465812 |
| LMNA | Lamin A/C | Protein Coding | 51 | GC01P156082 | 0.733804941 |
| ALOX5AP | Arachidonate 5-Lipoxygenase Activating Protein | Protein Coding | 47 | GC13P030713 | 0.728976846 |
| CYP3A5 | Cytochrome P450 Family 3 Subfamily A Member 5 | Protein Coding | 47 | GC07M099648 | 0.728976846 |
| NPSR1 | Neuropeptide S Receptor 1 | Protein Coding | 45 | GC07P034664 | 0.728976846 |
| PHF11 | PHD Finger Protein 11 | Protein Coding | 37 | GC13P049495 | 0.728976846 |
| LEF1 | Lymphoid Enhancer Binding Factor 1 | Protein Coding | 51 | GC04M108047 | 0.727862477 |
| SKP2 | S-Phase Kinase Associated Protein 2 | Protein Coding | 46 | GC05P036151 | 0.727862477 |
| CUL1 | Cullin 1 | Protein Coding | 45 | GC07P148697 | 0.727862477 |
| LDLR | Low Density Lipoprotein Receptor | Protein Coding | 54 | GC19P011091 | 0.727305472 |
| CXCL6 | C-X-C Motif Chemokine Ligand 6 | Protein Coding | 41 | GC04P073837 | 0.727305472 |
| HPGD | 15-Hydroxyprostaglandin Dehydrogenase | Protein Coding | 49 | GC04M174490 | 0.724366426 |
| SOD3 | Superoxide Dismutase 3 | Protein Coding | 45 | GC04P024798 | 0.724366426 |
| CHMP1A | Charged Multivesicular Body Protein 1A | Protein Coding | 42 | GC16M089644 | 0.724069595 |
| GRIA4 | Glutamate Ionotropic Receptor AMPA Type Subunit 4 | Protein Coding | 52 | GC11P105609 | 0.720332801 |
| PSMB8 | Proteasome 20S Subunit Beta 8 | Protein Coding | 52 | GC06M032840 | 0.720332801 |
| FUT2 | Fucosyltransferase 2 | Protein Coding | 48 | GC19P048695 | 0.720332801 |
| HLA-DRA | Major Histocompatibility Complex, Class II, DR Alpha | Protein Coding | 48 | GC06P032439 | 0.720332801 |
| TAP2 | Transporter 2, ATP Binding Cassette Subfamily B Member | Protein Coding | 47 | GC06M032821 | 0.720332801 |
| WDR5 | WD Repeat Domain 5 | Protein Coding | 47 | GC09P134135 | 0.720332801 |
| CFHR1 | Complement Factor H Related 1 | Protein Coding | 46 | GC01P196822 | 0.720332801 |
| FBXO7 | F-Box Protein 7 | Protein Coding | 45 | GC22P032474 | 0.720332801 |
| PRIM1 | DNA Primase Subunit 1 | Protein Coding | 45 | GC12M056731 | 0.720332801 |
| BRD3 | Bromodomain Containing 3 | Protein Coding | 44 | GC09M134030 | 0.720332801 |
| SURF1 | SURF1 Cytochrome C Oxidase Assembly Factor | Protein Coding | 44 | GC09M133351 | 0.720332801 |
| ABO | ABO, Alpha 1-3-N-Acetylgalactosaminyltransferase And Alpha 1-3-Galactosyltransferase | Protein Coding | 43 | GC09M133250 | 0.720332801 |
| CFHR5 | Complement Factor H Related 5 | Protein Coding | 43 | GC01P196977 | 0.720332801 |
| LMOD1 | Leiomodin 1 | Protein Coding | 43 | GC01M201896 | 0.720332801 |
| TOMM40 | Translocase Of Outer Mitochondrial Membrane 40 | Protein Coding | 43 | GC19P044890 | 0.720332801 |
| XK | X-Linked Kx Blood Group Antigen, Kell And VPS13A Binding Protein | Protein Coding | 43 | GC0XP037685 | 0.720332801 |
| ZFPM2 | Zinc Finger Protein, FOG Family Member 2 | Protein Coding | 43 | GC08P104590 | 0.720332801 |
| HLA-DOB | Major Histocompatibility Complex, Class II, DO Beta | Protein Coding | 42 | GC06M069269 | 0.720332801 |
| HLA-DRB5 | Major Histocompatibility Complex, Class II, DR Beta 5 | Protein Coding | 42 | GC06M069251 | 0.720332801 |
| STK19 | Serine/Threonine Kinase 19 | Protein Coding | 42 | GC06P031971 | 0.720332801 |
| BTNL2 | Butyrophilin Like 2 | Protein Coding | 41 | GC06M032393 | 0.720332801 |
| DACH1 | Dachshund Family Transcription Factor 1 | Protein Coding | 41 | GC13M071437 | 0.720332801 |
| GTF3C4 | General Transcription Factor IIIC Subunit 4 | Protein Coding | 41 | GC09P132671 | 0.720332801 |
| ASPM | Assembly Factor For Spindle Microtubules | Protein Coding | 40 | GC01M197084 | 0.720332801 |
| CKAP2L | Cytoskeleton Associated Protein 2 Like | Protein Coding | 40 | GC02M112736 | 0.720332801 |
| HLA-DQA2 | Major Histocompatibility Complex, Class II, DQ Alpha 2 | Protein Coding | 40 | GC06P032741 | 0.720332801 |
| HLA-DQB2 | Major Histocompatibility Complex, Class II, DQ Beta 2 | Protein Coding | 40 | GC06M032756 | 0.720332801 |
| MED22 | Mediator Complex Subunit 22 | Protein Coding | 40 | GC09M133338 | 0.720332801 |
| REXO4 | REX4 Homolog, 3'-5' Exonuclease | Protein Coding | 40 | GC09M133406 | 0.720332801 |
| BAIAP2L2 | BAR/IMD Domain Containing Adaptor Protein 2 Like 2 | Protein Coding | 38 | GC22M059589 | 0.720332801 |
| CFHR4 | Complement Factor H Related 4 | Protein Coding | 38 | GC01P196888 | 0.720332801 |
| GTF3C5 | General Transcription Factor IIIC Subunit 5 | Protein Coding | 38 | GC09P133030 | 0.720332801 |
| TMEM184B | Transmembrane Protein 184B | Protein Coding | 38 | GC22M060966 | 0.720332801 |
| ZNF224 | Zinc Finger Protein 224 | Protein Coding | 38 | GC19P044094 | 0.720332801 |
| ANKRD54 | Ankyrin Repeat Domain 54 | Protein Coding | 37 | GC22M059588 | 0.720332801 |
| CLASRP | CLK4 Associating Serine/Arginine Rich Protein | Protein Coding | 37 | GC19P045039 | 0.720332801 |
| GEMIN7 | Gem Nuclear Organelle Associated Protein 7 | Protein Coding | 37 | GC19P045075 | 0.720332801 |
| QPCTL | Glutaminyl-Peptide Cyclotransferase Like | Protein Coding | 37 | GC19P045692 | 0.720332801 |
| SURF2 | Surfeit 2 | Protein Coding | 37 | GC09P134347 | 0.720332801 |
| SURF6 | Surfeit 6 | Protein Coding | 37 | GC09M133331 | 0.720332801 |
| ZNF180 | Zinc Finger Protein 180 | Protein Coding | 37 | GC19M067980 | 0.720332801 |
| ZNF45 | Zinc Finger Protein 45 | Protein Coding | 37 | GC19M067969 | 0.720332801 |
| CACFD1 | Calcium Channel Flower Domain Containing 1 | Protein Coding | 36 | GC09P133459 | 0.720332801 |
| PRDM15 | PR/SET Domain 15 | Protein Coding | 36 | GC21M041798 | 0.720332801 |
| LY6G5B | Lymphocyte Antigen 6 Family Member G5B | Protein Coding | 35 | GC06P087744 | 0.720332801 |
| MZT1 | Mitotic Spindle Organizing Protein 1 | Protein Coding | 35 | GC13M072708 | 0.720332801 |
| ZNF230 | Zinc Finger Protein 230 | Protein Coding | 35 | GC19P044002 | 0.720332801 |
| MSANTD4 | Myb/SANT DNA Binding Domain Containing 4 With Coiled-Coils | Protein Coding | 34 | GC11M105995 | 0.720332801 |
| STKLD1 | Serine/Threonine Kinase Like Domain Containing 1 | Protein Coding | 34 | GC09P133376 | 0.720332801 |
| ZNF227 | Zinc Finger Protein 227 | Protein Coding | 34 | GC19P044207 | 0.720332801 |
| ZNF234 | Zinc Finger Protein 234 | Protein Coding | 34 | GC19P044141 | 0.720332801 |
| OBP2B | Odorant Binding Protein 2B | Protein Coding | 33 | GC09M133205 | 0.720332801 |
| ZNF155 | Zinc Finger Protein 155 | Protein Coding | 33 | GC19P043967 | 0.720332801 |
| ZNF225 | Zinc Finger Protein 225 | Protein Coding | 33 | GC19P069927 | 0.720332801 |
| ZNF284 | Zinc Finger Protein 284 | Protein Coding | 31 | GC19P044072 | 0.720332801 |
| ZNF285 | Zinc Finger Protein 285 | Protein Coding | 31 | GC19M067978 | 0.720332801 |
| NT5DC4 | 5'-Nucleotidase Domain Containing 4 | Protein Coding | 30 | GC02P123133 | 0.720332801 |
| FAM227A | Family With Sequence Similarity 227 Member A | Protein Coding | 29 | GC22M038578 | 0.720332801 |
| MIR9-1HG | MIR9-1 Host Gene | RNA Gene | 29 | GC01M156408 | 0.720332801 |
| SNORD24 | Small Nucleolar RNA, C/D Box 24 | RNA Gene | 19 | GC09P133349 | 0.720332801 |
| CYP21A1P | Cytochrome P450 Family 21 Subfamily A Member 1, Pseudogene | Pseudogene | 17 | GC06P032005 | 0.720332801 |
| EEF1A1P5 | Eukaryotic Translation Elongation Factor 1 Alpha 1 Pseudogene 5 | Pseudogene | 17 | GC09P133019 | 0.720332801 |
| HLA-DRB6 | Major Histocompatibility Complex, Class II, DR Beta 6 (Pseudogene) | Pseudogene | 17 | GC06M069253 | 0.720332801 |
| SNORD36A | Small Nucleolar RNA, C/D Box 36A | RNA Gene | 17 | GC09P134345 | 0.720332801 |
| ZFPM2-AS1 | ZFPM2 Antisense RNA 1 | RNA Gene | 17 | GC08M105546 | 0.720332801 |
| OR10J6P | Olfactory Receptor Family 10 Subfamily J Member 6 Pseudogene | Pseudogene | 16 | GC01P159598 | 0.720332801 |
| SNORD36B | Small Nucleolar RNA, C/D Box 36B | RNA Gene | 16 | GC09P134343 | 0.720332801 |
| SNORD36C | Small Nucleolar RNA, C/D Box 36C | RNA Gene | 16 | GC09P134346 | 0.720332801 |
| ELF3-AS1 | ELF3 Antisense RNA 1 | RNA Gene | 15 | GC01M202001 | 0.720332801 |
| HLA-DQB1-AS1 | HLA-DQB1 Antisense RNA 1 | RNA Gene | 15 | GC06P032659 | 0.720332801 |
| APOC1P1 | Apolipoprotein C1 Pseudogene 1 | Pseudogene | 14 | GC19P044926 | 0.720332801 |
| MIR4693 | MicroRNA 4693 | RNA Gene | 14 | GC11P103849 | 0.720332801 |
| DM1-AS | DM1 Locus Antisense RNA | RNA Gene | 13 | GC19P070002 | 0.720332801 |
| LOC100130548 | Uncharacterized LOC100130548 | RNA Gene | 12 | GC09P134054 | 0.720332801 |
| CASP4LP | Caspase 4 Like, Pseudogene | Pseudogene | 11 | GC11M104904 | 0.720332801 |
| LOC101928009 | Uncharacterized LOC101928009 | RNA Gene | 11 | GC01P153174 | 0.720332801 |
| CRPP1 | C-Reactive Protein Pseudogene 1 | Pseudogene | 9 | GC01M159675 | 0.720332801 |
| ENSG00000223563 | Novel Transcript | RNA Gene | 9 | GC21P026855 | 0.720332801 |
| ENSG00000228274 | Novel Transcript, Antisense To CBY1 | RNA Gene | 9 | GC22M038667 | 0.720332801 |
| ENSG00000249007 | Novel Transcript | RNA Gene | 9 | GC01P202054 | 0.720332801 |
| RNU6-160P | RNA, U6 Small Nuclear 160, Pseudogene | Pseudogene | 9 | GC01P153331 | 0.720332801 |
| RNU6-952P | RNA, U6 Small Nuclear 952, Pseudogene | Pseudogene | 9 | GC11P102313 | 0.720332801 |
| RPS10P21 | Ribosomal Protein S10 Pseudogene 21 | Pseudogene | 9 | GC13M071913 | 0.720332801 |
| S100A15A | S100 Calcium Binding Protein A15A (Pseudogene) | Pseudogene | 9 | GC01M153397 | 0.720332801 |
| ENSG00000259605 | Novel Transcript, Antisense To SIX5 | RNA Gene | 8 | GC19P045764 | 0.720332801 |
| ENSG00000267282 | Novel Transcript, Antisense To PVRL2 | RNA Gene | 8 | GC19M067990 | 0.720332801 |
| ENSG00000273473 | Novel Transcript | RNA Gene | 8 | GC09P134408 | 0.720332801 |
| ENSG00000288528 | Novel Transcript | RNA Gene | 8 | GC11M102303 | 0.720332801 |
| GPX1P2 | Glutathione Peroxidase Pseudogene 2 | Pseudogene | 8 | GC21M027143 | 0.720332801 |
| LOC101928477 | Uncharacterized LOC101928477 | RNA Gene | 8 | GC11P102627 | 0.720332801 |
| LOC105447645 | Uncharacterized LOC105447645 | RNA Gene | 8 | GC19M068135 | 0.720332801 |
| ENSG00000269148 | Novel Transcript, Antisense To SYMPK | RNA Gene | 7 | GC19P045830 | 0.720332801 |
| LCN1P2 | Lipocalin 1 Pseudogene 2 | Pseudogene | 7 | GC09P133317 | 0.720332801 |
| lnc-BORA-13 | | RNA Gene | 7 | GC13P071867 | 0.720332801 |
| ENSG00000229673 | Novel Transcript | RNA Gene | 6 | GC22P041080 | 0.720332801 |
| HSALNG0132438 | | RNA Gene | 6 | GC21P026967 | 0.720332801 |
| lnc-ABO-33 | | RNA Gene | 6 | GC09M133232 | 0.720332801 |
| lnc-BIRC2-1 | | RNA Gene | 6 | GC11P102318 | 0.720332801 |
| lnc-BPIFC-4 | | RNA Gene | 6 | GC22M032629 | 0.720332801 |
| lnc-DYNC2H1-4 | | RNA Gene | 6 | GC11P102811 | 0.720332801 |
| lnc-GABPA-22 | | RNA Gene | 6 | GC21P026846 | 0.720332801 |
| ENSG00000270679 | Mitochondrial Ribosomal Protein S15 (MRPS15) Pseudogene | Pseudogene | 5 | GC19P069940 | 0.720332801 |
| HSALNG0049404 | | RNA Gene | 5 | GC06M069948 | 0.720332801 |
| HSALNG0075007 | | RNA Gene | 5 | GC09M133411 | 0.720332801 |
| HSALNG0075008 | | RNA Gene | 5 | GC09P134423 | 0.720332801 |
| HSALNG0086792 | | RNA Gene | 5 | GC11P102553 | 0.720332801 |
| HSALNG0135390 | | RNA Gene | 5 | GC22M059725 | 0.720332801 |
| L13304-024 | | RNA Gene | 5 | GC22M059862 | 0.720332801 |
| L13715-018 | | RNA Gene | 5 | GC22M059865 | 0.720332801 |
| LOC124902739 | Uncharacterized LOC124902739 | RNA Gene | 5 | GC11M102316 | 0.720332801 |
| MK280383 | | RNA Gene | 5 | GC22P032743 | 0.720332801 |
| MN308987 | | RNA Gene | 5 | GC01M153357 | 0.720332801 |
| NONHSAG026007.2 | | RNA Gene | 5 | GC19P044892 | 0.720332801 |
| NONHSAG045793.2 | | RNA Gene | 5 | GC06P090311 | 0.720332801 |
| RF00017-3533 | | RNA Gene | 5 | GC22P041574 | 0.720332801 |
| RF00017-7610 | | RNA Gene | 5 | GC09P134438 | 0.720332801 |
| RF00026-198 | | RNA Gene | 5 | GC11P102316 | 0.720332801 |
| lnc-BTNL2-2 | | RNA Gene | 5 | GC06M032435 | 0.720332801 |
| lnc-DACH1-1 | | RNA Gene | 5 | GC13M071914 | 0.720332801 |
| lnc-LARGE1-8 | | RNA Gene | 5 | GC22M032860 | 0.720332801 |
| lnc-MMP12-2 | | RNA Gene | 5 | GC11M102945 | 0.720332801 |
| lnc-MMP7-1 | | RNA Gene | 5 | GC11M102552 | 0.720332801 |
| lnc-SURF1-3 | | RNA Gene | 5 | GC09M133363 | 0.720332801 |
| lnc-TIMP3-2 | | RNA Gene | 5 | GC22P032652 | 0.720332801 |
| lnc-ZNF296-6 | | RNA Gene | 5 | GC19M044907 | 0.720332801 |
| piR-37125 | | RNA Gene | 5 | GC09P134348 | 0.720332801 |
| piR-56133-230 | | RNA Gene | 5 | GC09P134355 | 0.720332801 |
| ENSG00000256035 | Casein Kinase 1, Alpha 1 (CSNK1A1) Pseudogene | Pseudogene | 4 | GC11M102944 | 0.720332801 |
| HSALNG0009701 | | RNA Gene | 4 | GC01P202045 | 0.720332801 |
| HSALNG0075009 | | RNA Gene | 4 | GC09M133421 | 0.720332801 |
| HSALNG0086791 | | RNA Gene | 4 | GC11P102527 | 0.720332801 |
| HSALNG0086813 | | RNA Gene | 4 | GC11P102931 | 0.720332801 |
| HSALNG0097858 | | RNA Gene | 4 | GC13P071875 | 0.720332801 |
| JA662190 |  | RNA Gene | 4 | GC11M102938 | 0.720332801 |
| NONHSAG053687.2 | | RNA Gene | 4 | GC09P134332 | 0.720332801 |
| lnc-MMP1-1 | | RNA Gene | 4 | GC11M102943 | 0.720332801 |
| lnc-MMP10-3 | | RNA Gene | 4 | GC11M102940 | 0.720332801 |
| piR-36588-076 | | RNA Gene | 4 | GC11M102568 | 0.720332801 |
| piR-43105-432 | | RNA Gene | 4 | GC22M032826 | 0.720332801 |
| piR-52079-130 | | RNA Gene | 4 | GC22P032764 | 0.720332801 |
| piR-57134-009 | | RNA Gene | 4 | GC01M202064 | 0.720332801 |
| piR-59907-030 | | RNA Gene | 4 | GC09M133272 | 0.720332801 |
| HSALNG0007587 | | RNA Gene | 3 | GC01P156446 | 0.720332801 |
| LOC100996886 | Complement Factor H-Related Protein 3-Like | Pseudogene | 3 | GC01P196850 | 0.720332801 |
| LOC124902741 | Uncharacterized LOC124902741 | RNA Gene | 3 | GC11P102852 | 0.720332801 |
| piR-33614-045 | | RNA Gene | 3 | GC11P102677 | 0.720332801 |
| piR-37656-044 | | RNA Gene | 3 | GC11P102906 | 0.720332801 |
| NONHSAG026010.2 | | RNA Gene | 2 | GC19P044907 | 0.720332801 |
| PRKG1 | Protein Kinase CGMP-Dependent 1 | Protein Coding | 54 | GC10P050991 | 0.716055334 |
| SMPD1 | Sphingomyelin Phosphodiesterase 1 | Protein Coding | 52 | GC11P006390 | 0.716055334 |
| EIF2AK2 | Eukaryotic Translation Initiation Factor 2 Alpha Kinase 2 | Protein Coding | 51 | GC02M037099 | 0.716055334 |
| MAPK12 | Mitogen-Activated Protein Kinase 12 | Protein Coding | 51 | GC22M059568 | 0.716055334 |
| GALNS | Galactosamine (N-Acetyl)-6-Sulfatase | Protein Coding | 50 | GC16M088813 | 0.716055334 |
| GNA11 | G Protein Subunit Alpha 11 | Protein Coding | 50 | GC19P003094 | 0.716055334 |
| NCF1 | Neutrophil Cytosolic Factor 1 | Protein Coding | 50 | GC07P075854 | 0.716055334 |
| CALM1 | Calmodulin 1 | Protein Coding | 48 | GC14P090396 | 0.716055334 |
| CUBN | Cubilin | Protein Coding | 48 | GC10M016824 | 0.716055334 |
| HTRA2 | HtrA Serine Peptidase 2 | Protein Coding | 48 | GC02P074529 | 0.716055334 |
| IRF8 | Interferon Regulatory Factor 8 | Protein Coding | 48 | GC16P085898 | 0.716055334 |
| NPHS1 | NPHS1 Adhesion Molecule, Nephrin | Protein Coding | 48 | GC19M035825 | 0.716055334 |
| RIPK2 | Receptor Interacting Serine/Threonine Kinase 2 | Protein Coding | 48 | GC08P089813 | 0.716055334 |
| SLC9A3R1 | SLC9A3 Regulator 1 | Protein Coding | 48 | GC17P074749 | 0.716055334 |
| XPC | XPC Complex Subunit, DNA Damage Recognition And Repair Factor | Protein Coding | 48 | GC03M021331 | 0.716055334 |
| PTPN3 | Protein Tyrosine Phosphatase Non-Receptor Type 3 | Protein Coding | 46 | GC09M109375 | 0.716055334 |
| RNASEL | Ribonuclease L | Protein Coding | 46 | GC01M182573 | 0.716055334 |
| GRIP1 | Glutamate Receptor Interacting Protein 1 | Protein Coding | 45 | GC12M066347 | 0.716055334 |
| STX3 | Syntaxin 3 | Protein Coding | 44 | GC11P059713 | 0.716055334 |
| SIRT7 | Sirtuin 7 | Protein Coding | 43 | GC17M081911 | 0.716055334 |
| SPAG5 | Sperm Associated Antigen 5 | Protein Coding | 40 | GC17M028577 | 0.716055334 |
| UBP1 | Upstream Binding Protein 1 | Protein Coding | 38 | GC03M033404 | 0.716055334 |
| WDFY2 | WD Repeat And FYVE Domain Containing 2 | Protein Coding | 37 | GC13P051584 | 0.716055334 |
| MIRLET7F1 | MicroRNA Let-7f-1 | RNA Gene | 20 | GC09P094499 | 0.716055334 |
| MIR6796 | MicroRNA 6796 | RNA Gene | 10 | GC19P040369 | 0.716055334 |
| CCND3 | Cyclin D3 | Protein Coding | 49 | GC06M041934 | 0.713523746 |
| KIF3A | Kinesin Family Member 3A | Protein Coding | 43 | GC05M132689 | 0.713523746 |
| PIBF1 | Progesterone Immunomodulatory Binding Factor 1 | Protein Coding | 43 | GC13P072782 | 0.713523746 |
| E2F5 | E2F Transcription Factor 5 | Protein Coding | 42 | GC08P085177 | 0.713523746 |
| PIGU | Phosphatidylinositol Glycan Anchor Biosynthesis Class U | Protein Coding | 40 | GC20M034560 | 0.713523746 |
| HFE | Homeostatic Iron Regulator | Protein Coding | 48 | GC06P026087 | 0.711679697 |
| GJA4 | Gap Junction Protein Alpha 4 | Protein Coding | 45 | GC01P034792 | 0.707669616 |
| AMBN | Ameloblastin | Protein Coding | 43 | GC04P070593 | 0.704307616 |
| EPHB4 | EPH Receptor B4 | Protein Coding | 56 | GC07M102134 | 0.699546933 |
| KLF6 | KLF Transcription Factor 6 | Protein Coding | 45 | GC10M003779 | 0.699546933 |
| YAP1 | Yes1 Associated Transcriptional Regulator | Protein Coding | 50 | GC11P102110 | 0.698548973 |
| MUC15 | Mucin 15, Cell Surface Associated | Protein Coding | 38 | GC11M026537 | 0.69489181 |
| MIR214 | MicroRNA 214 | RNA Gene | 24 | GC01M172234 | 0.69489181 |
| RASSF1 | Ras Association Domain Family Member 1 | Protein Coding | 45 | GC03M050329 | 0.684749722 |
| CERS2 | Ceramide Synthase 2 | Protein Coding | 44 | GC01M150960 | 0.684749722 |
| WASF3 | WASP Family Member 3 | Protein Coding | 42 | GC13P026557 | 0.684749722 |
| FABP5 | Fatty Acid Binding Protein 5 | Protein Coding | 45 | GC08P081282 | 0.683921158 |
| MIR146B | MicroRNA 146b | RNA Gene | 23 | GC10P102436 | 0.683921158 |
| AURKB | Aurora Kinase B | Protein Coding | 52 | GC17M010614 | 0.680624247 |
| TUBA1A | Tubulin Alpha 1a | Protein Coding | 52 | GC12M049184 | 0.680624247 |
| HDAC5 | Histone Deacetylase 5 | Protein Coding | 51 | GC17M044076 | 0.680624247 |
| IKZF1 | IKAROS Family Zinc Finger 1 | Protein Coding | 51 | GC07P050303 | 0.680624247 |
| MUSK | Muscle Associated Receptor Tyrosine Kinase | Protein Coding | 51 | GC09P110668 | 0.680624247 |
| TBP | TATA-Box Binding Protein | Protein Coding | 51 | GC06P170554 | 0.680624247 |
| ASNS | Asparagine Synthetase (Glutamine-Hydrolyzing) | Protein Coding | 50 | GC07M097854 | 0.680624247 |
| FGF8 | Fibroblast Growth Factor 8 | Protein Coding | 50 | GC10M101770 | 0.680624247 |
| STAT2 | Signal Transducer And Activator Of Transcription 2 | Protein Coding | 50 | GC12M056341 | 0.680624247 |
| C1R | Complement C1r | Protein Coding | 49 | GC12M007889 | 0.680624247 |
| CDK7 | Cyclin Dependent Kinase 7 | Protein Coding | 49 | GC05P069403 | 0.680624247 |
| IRF5 | Interferon Regulatory Factor 5 | Protein Coding | 49 | GC07P128937 | 0.680624247 |
| ARHGEF1 | Rho Guanine Nucleotide Exchange Factor 1 | Protein Coding | 48 | GC19P041883 | 0.680624247 |
| CLEC7A | C-Type Lectin Domain Containing 7A | Protein Coding | 48 | GC12M021841 | 0.680624247 |
| RTN4 | Reticulon 4 | Protein Coding | 48 | GC02M054934 | 0.680624247 |
| TMPO | Thymopoietin | Protein Coding | 48 | GC12P098515 | 0.680624247 |
| AVP | Arginine Vasopressin | Protein Coding | 47 | GC20M003082 | 0.680624247 |
| GHRHR | Growth Hormone Releasing Hormone Receptor | Protein Coding | 47 | GC07P030938 | 0.680624247 |
| IL10RB | Interleukin 10 Receptor Subunit Beta | Protein Coding | 47 | GC21P033266 | 0.680624247 |
| LOXL4 | Lysyl Oxidase Like 4 | Protein Coding | 47 | GC10M098247 | 0.680624247 |
| PTS | 6-Pyruvoyltetrahydropterin Synthase | Protein Coding | 47 | GC11P112226 | 0.680624247 |
| TCF3 | Transcription Factor 3 | Protein Coding | 47 | GC19M001609 | 0.680624247 |
| CLDN18 | Claudin 18 | Protein Coding | 46 | GC03P137998 | 0.680624247 |
| CBX3 | Chromobox 3 | Protein Coding | 45 | GC07P026201 | 0.680624247 |
| CHKA | Choline Kinase Alpha | Protein Coding | 45 | GC11M068052 | 0.680624247 |
| E2F2 | E2F Transcription Factor 2 | Protein Coding | 45 | GC01M023668 | 0.680624247 |
| HOXA2 | Homeobox A2 | Protein Coding | 45 | GC07M027100 | 0.680624247 |
| KMT2D | Lysine Methyltransferase 2D | Protein Coding | 45 | GC12M049018 | 0.680624247 |
| MB | Myoglobin | Protein Coding | 45 | GC22M035606 | 0.680624247 |
| PYY | Peptide YY | Protein Coding | 45 | GC17M043952 | 0.680624247 |
| FAF1 | Fas Associated Factor 1 | Protein Coding | 44 | GC01M050439 | 0.680624247 |
| GPR65 | G Protein-Coupled Receptor 65 | Protein Coding | 44 | GC14P088005 | 0.680624247 |
| HPSE2 | Heparanase 2 (Inactive) | Protein Coding | 44 | GC10M098457 | 0.680624247 |
| PRRX1 | Paired Related Homeobox 1 | Protein Coding | 44 | GC01P170662 | 0.680624247 |
| AAAS | Aladin WD Repeat Nucleoporin | Protein Coding | 43 | GC12M053307 | 0.680624247 |
| ERO1A | Endoplasmic Reticulum Oxidoreductase 1 Alpha | Protein Coding | 43 | GC14M052640 | 0.680624247 |
| GNA12 | G Protein Subunit Alpha 12 | Protein Coding | 43 | GC07M002728 | 0.680624247 |
| IRAK2 | Interleukin 1 Receptor Associated Kinase 2 | Protein Coding | 43 | GC03P012693 | 0.680624247 |
| MAP1LC3A | Microtubule Associated Protein 1 Light Chain 3 Alpha | Protein Coding | 43 | GC20P034546 | 0.680624247 |
| PARD6A | Par-6 Family Cell Polarity Regulator Alpha | Protein Coding | 43 | GC16P067661 | 0.680624247 |
| ST8SIA1 | ST8 Alpha-N-Acetyl-Neuraminide Alpha-2,8-Sialyltransferase 1 | Protein Coding | 43 | GC12M022063 | 0.680624247 |
| TFCP2 | Transcription Factor CP2 | Protein Coding | 43 | GC12M051093 | 0.680624247 |
| CD200R1 | CD200 Receptor 1 | Protein Coding | 42 | GC03M112921 | 0.680624247 |
| AOPEP | Aminopeptidase O (Putative) | Protein Coding | 41 | GC09P094728 | 0.680624247 |
| APBA3 | Amyloid Beta Precursor Protein Binding Family A Member 3 | Protein Coding | 41 | GC19M003750 | 0.680624247 |
| KLK14 | Kallikrein Related Peptidase 14 | Protein Coding | 41 | GC19M051077 | 0.680624247 |
| DNAH8 | Dynein Axonemal Heavy Chain 8 | Protein Coding | 40 | GC06P087939 | 0.680624247 |
| GCM1 | Glial Cells Missing Transcription Factor 1 | Protein Coding | 40 | GC06M069614 | 0.680624247 |
| CCL16 | C-C Motif Chemokine Ligand 16 | Protein Coding | 39 | GC17M035976 | 0.680624247 |
| MPP3 | MAGUK P55 Scaffold Protein 3 | Protein Coding | 39 | GC17M043800 | 0.680624247 |
| UBXN11 | UBX Domain Protein 11 | Protein Coding | 39 | GC01M026281 | 0.680624247 |
| GTF3A | General Transcription Factor IIIA | Protein Coding | 38 | GC13P027427 | 0.680624247 |
| HORMAD1 | HORMA Domain Containing 1 | Protein Coding | 38 | GC01M152402 | 0.680624247 |
| ABT1 | Activator Of Basal Transcription 1 | Protein Coding | 37 | GC06P087515 | 0.680624247 |
| SSX2 | SSX Family Member 2 | Protein Coding | 36 | GC0XM052696 | 0.680624247 |
| H3C14 | H3 Clustered Histone 14 | Protein Coding | 34 | GC01M152334 | 0.680624247 |
| KRBOX4 | KRAB Box Domain Containing 4 | Protein Coding | 34 | GC0XP046614 | 0.680624247 |
| CTAG1B | Cancer/Testis Antigen 1B | Protein Coding | 31 | GC0XM154617 | 0.680624247 |
| MIR9-2 | MicroRNA 9-2 | RNA Gene | 22 | GC05M088666 | 0.680624247 |
| MIR9-3 | MicroRNA 9-3 | RNA Gene | 21 | GC15P089368 | 0.680624247 |
| MIR503HG | MIR503 Host Gene | RNA Gene | 20 | GC0XM134695 | 0.680624247 |
| MIR376C | MicroRNA 376c | RNA Gene | 19 | GC14P110439 | 0.680624247 |
| MIR4423 | MicroRNA 4423 | RNA Gene | 16 | GC01P085133 | 0.680624247 |
| MIR4429 | MicroRNA 4429 | RNA Gene | 16 | GC02M011540 | 0.680624247 |
| MIR939 | MicroRNA 939 | RNA Gene | 16 | GC08M144394 | 0.680624247 |
| MIR522 | MicroRNA 522 | RNA Gene | 15 | GC19P053751 | 0.680624247 |
| MIR7109 | MicroRNA 7109 | RNA Gene | 12 | GC22M031621 | 0.680624247 |
| F13A1 | Coagulation Factor XIII A Chain | Protein Coding | 50 | GC06M006144 | 0.679998636 |
| COL5A2 | Collagen Type V Alpha 2 Chain | Protein Coding | 46 | GC02M189031 | 0.679998636 |
| ING4 | Inhibitor Of Growth Family Member 4 | Protein Coding | 40 | GC12M006650 | 0.6766361 |
| SPINK5 | Serine Peptidase Inhibitor Kazal Type 5 | Protein Coding | 43 | GC05P148025 | 0.67124927 |
| CCL24 | C-C Motif Chemokine Ligand 24 | Protein Coding | 42 | GC07M076931 | 0.67124927 |
| AXL | AXL Receptor Tyrosine Kinase | Protein Coding | 55 | GC19P041219 | 0.670183599 |
| EPCAM | Epithelial Cell Adhesion Molecule | Protein Coding | 51 | GC02P047345 | 0.66999197 |
| COL4A2 | Collagen Type IV Alpha 2 Chain | Protein Coding | 49 | GC13P110305 | 0.66999197 |
| ALPP | Alkaline Phosphatase, Placental | Protein Coding | 47 | GC02P232378 | 0.66999197 |
| ACKR3 | Atypical Chemokine Receptor 3 | Protein Coding | 44 | GC02P236537 | 0.66999197 |
| COL10A1 | Collagen Type X Alpha 1 Chain | Protein Coding | 44 | GC06M116118 | 0.66999197 |
| PRKCZ | Protein Kinase C Zeta | Protein Coding | 52 | GC01P004159 | 0.667133272 |
| DUSP1 | Dual Specificity Phosphatase 1 | Protein Coding | 49 | GC05M172768 | 0.667133272 |
| MIR124-1 | MicroRNA 124-1 | RNA Gene | 21 | GC08M009903 | 0.667133272 |
| NF1 | Neurofibromin 1 | Protein Coding | 51 | GC17P031094 | 0.66210115 |
| RHEB | Ras Homolog, MTORC1 Binding | Protein Coding | 51 | GC07M151466 | 0.66210115 |
| GABRA3 | Gamma-Aminobutyric Acid Type A Receptor Subunit Alpha3 | Protein Coding | 49 | GC0XM152166 | 0.66210115 |
| DMD | Dystrophin | Protein Coding | 48 | GC0XM031097 | 0.66210115 |
| ORM1 | Orosomucoid 1 | Protein Coding | 43 | GC09P114323 | 0.66210115 |
| SARM1 | Sterile Alpha And TIR Motif Containing 1 | Protein Coding | 43 | GC17P028364 | 0.66210115 |
| BBC3 | BCL2 Binding Component 3 | Protein Coding | 42 | GC19M047220 | 0.66210115 |
| CSDE1 | Cold Shock Domain Containing E1 | Protein Coding | 42 | GC01M114716 | 0.66210115 |
| ELK3 | ETS Transcription Factor ELK3 | Protein Coding | 40 | GC12P096194 | 0.66210115 |
| TMED7 | Transmembrane P24 Trafficking Protein 7 | Protein Coding | 37 | GC05M115613 | 0.66210115 |
| SELENOK | Selenoprotein K | Protein Coding | 34 | GC03M053884 | 0.66210115 |
| CCDC183 | Coiled-Coil Domain Containing 183 | Protein Coding | 33 | GC09P136796 | 0.66210115 |
| SELENOF | Selenoprotein F | Protein Coding | 33 | GC01M086863 | 0.66210115 |
| TBXA2R | Thromboxane A2 Receptor | Protein Coding | 52 | GC19M003594 | 0.661961675 |
| CHRM2 | Cholinergic Receptor Muscarinic 2 | Protein Coding | 51 | GC07P136868 | 0.661961675 |
| ADCY9 | Adenylate Cyclase 9 | Protein Coding | 48 | GC16M003953 | 0.661961675 |
| BCL2A1 | BCL2 Related Protein A1 | Protein Coding | 45 | GC15M118988 | 0.661961675 |
| IL13RA1 | Interleukin 13 Receptor Subunit Alpha 1 | Protein Coding | 45 | GC0XP118727 | 0.661961675 |
| GSDMB | Gasdermin B | Protein Coding | 37 | GC17M039904 | 0.661961675 |
| KRT8 | Keratin 8 | Protein Coding | 50 | GC12M052897 | 0.659778059 |
| REG3A | Regenerating Family Member 3 Alpha | Protein Coding | 40 | GC02M079157 | 0.659778059 |
| MIR373 | MicroRNA 373 | RNA Gene | 22 | GC19P070437 | 0.659778059 |
| RXFP1 | Relaxin Family Peptide Receptor 1 | Protein Coding | 47 | GC04P158315 | 0.656443238 |
| AMELX | Amelogenin X-Linked | Protein Coding | 42 | GC0XP011293 | 0.656443238 |
| CD24 | CD24 Molecule | Protein Coding | 40 | GC06M106969 | 0.656443238 |
| MIR206 | MicroRNA 206 | RNA Gene | 22 | GC06P052144 | 0.656443238 |
| GSN | Gelsolin | Protein Coding | 51 | GC09P121201 | 0.654366195 |
| STC2 | Stanniocalcin 2 | Protein Coding | 45 | GC05M173314 | 0.654366195 |
| MCAM | Melanoma Cell Adhesion Molecule | Protein Coding | 45 | GC11M119308 | 0.652915061 |
| IFI27 | Interferon Alpha Inducible Protein 27 | Protein Coding | 40 | GC14P094104 | 0.652915061 |
| CREBBP | CREB Binding Protein | Protein Coding | 55 | GC16M007712 | 0.648877561 |
| FOXO1 | Forkhead Box O1 | Protein Coding | 51 | GC13M040555 | 0.648877561 |
| AFP | Alpha Fetoprotein | Protein Coding | 48 | GC04P073431 | 0.648877561 |
| LTB4R | Leukotriene B4 Receptor | Protein Coding | 47 | GC14P024311 | 0.648329318 |
| CD55 | CD55 Molecule (Cromer Blood Group) | Protein Coding | 51 | GC01P207321 | 0.645469308 |
| COL9A2 | Collagen Type IX Alpha 2 Chain | Protein Coding | 45 | GC01M040300 | 0.638239026 |
| IL24 | Interleukin 24 | Protein Coding | 44 | GC01P206897 | 0.633523345 |
| SEPTIN7 | Septin 7 | Protein Coding | 38 | GC07P036057 | 0.63259083 |
| AKR1B1 | Aldo-Keto Reductase Family 1 Member B | Protein Coding | 50 | GC07M134442 | 0.6286847 |
| FOXC2 | Forkhead Box C2 | Protein Coding | 45 | GC16P086587 | 0.6286847 |
| PAEP | Progestagen Associated Endometrial Protein | Protein Coding | 43 | GC09P135561 | 0.6286847 |
| LINC00312 | Long Intergenic Non-Protein Coding RNA 312 | RNA Gene | 20 | GC03P008571 | 0.6286847 |
| EHF | ETS Homologous Factor | Protein Coding | 41 | GC11P034621 | 0.626605809 |
| BCL2L11 | BCL2 Like 11 | Protein Coding | 47 | GC02P111119 | 0.624566436 |
| ADPRH | ADP-Ribosylarginine Hydrolase | Protein Coding | 38 | GC03P119579 | 0.624566436 |
| B3GNT8 | UDP-GlcNAc:BetaGal Beta-1,3-N-Acetylglucosaminyltransferase 8 | Protein Coding | 37 | GC19M041425 | 0.622613251 |
| CPA3 | Carboxypeptidase A3 | Protein Coding | 44 | GC03P148865 | 0.621142089 |
| ICOSLG | Inducible T Cell Costimulator Ligand | Protein Coding | 43 | GC21M044222 | 0.621142089 |
| CCL22 | C-C Motif Chemokine Ligand 22 | Protein Coding | 42 | GC16P057359 | 0.621142089 |
| CRLF2 | Cytokine Receptor Like Factor 2 | Protein Coding | 41 | GC0XM001190 | 0.621142089 |
| FLI1 | Fli-1 Proto-Oncogene, ETS Transcription Factor | Protein Coding | 50 | GC11P128686 | 0.619322598 |
| SIM2 | SIM BHLH Transcription Factor 2 | Protein Coding | 40 | GC21P036699 | 0.619322598 |
| BRCA1 | BRCA1 DNA Repair Associated | Protein Coding | 52 | GC17M043044 | 0.618385792 |
| TNFAIP6 | TNF Alpha Induced Protein 6 | Protein Coding | 43 | GC02P151357 | 0.618385792 |
| MGP | Matrix Gla Protein | Protein Coding | 45 | GC12M021919 | 0.607276142 |
| CA12 | Carbonic Anhydrase 12 | Protein Coding | 50 | GC15M063321 | 0.605825424 |
| CFB | Complement Factor B | Protein Coding | 50 | GC06P031945 | 0.605825424 |
| PRDX6 | Peroxiredoxin 6 | Protein Coding | 50 | GC01P173477 | 0.605825424 |
| LAMB3 | Laminin Subunit Beta 3 | Protein Coding | 48 | GC01M209614 | 0.605825424 |
| COL4A4 | Collagen Type IV Alpha 4 Chain | Protein Coding | 47 | GC02M226973 | 0.605825424 |
| POU5F1 | POU Class 5 Homeobox 1 | Protein Coding | 47 | GC06M069160 | 0.605825424 |
| COL11A2 | Collagen Type XI Alpha 2 Chain | Protein Coding | 46 | GC06M033162 | 0.605825424 |
| COL11A1 | Collagen Type XI Alpha 1 Chain | Protein Coding | 45 | GC01M102876 | 0.605825424 |
| SPTBN1 | Spectrin Beta, Non-Erythrocytic 1 | Protein Coding | 48 | GC02P054456 | 0.605764806 |
| HES1 | Hes Family BHLH Transcription Factor 1 | Protein Coding | 46 | GC03P194136 | 0.605764806 |
| CMKLR1 | Chemerin Chemokine-Like Receptor 1 | Protein Coding | 44 | GC12M108288 | 0.605764806 |
| BST2 | Bone Marrow Stromal Cell Antigen 2 | Protein Coding | 42 | GC19M017403 | 0.605764806 |
| CXCL14 | C-X-C Motif Chemokine Ligand 14 | Protein Coding | 39 | GC05M135617 | 0.605764806 |
| NEAT1 | Nuclear Paraspeckle Assembly Transcript 1 | RNA Gene | 27 | GC11P070383 | 0.605764806 |
| CDK11A | Cyclin Dependent Kinase 11A | Protein Coding | 41 | GC01M001702 | 0.604373574 |
| COL6A1 | Collagen Type VI Alpha 1 Chain | Protein Coding | 47 | GC21P045981 | 0.603709757 |
| GNAQ | G Protein Subunit Alpha Q | Protein Coding | 51 | GC09M077716 | 0.599129796 |
| LIFR | LIF Receptor Subunit Alpha | Protein Coding | 49 | GC05M038475 | 0.599129796 |
| SOX2 | SRY-Box Transcription Factor 2 | Protein Coding | 47 | GC03P181711 | 0.599129796 |
| KRT16 | Keratin 16 | Protein Coding | 45 | GC17M041609 | 0.599129796 |
| SPAG9 | Sperm Associated Antigen 9 | Protein Coding | 43 | GC17M050962 | 0.599129796 |
| SCNN1A | Sodium Channel Epithelial 1 Subunit Alpha | Protein Coding | 51 | GC12M006346 | 0.595085979 |
| SOS1 | SOS Ras/Rac Guanine Nucleotide Exchange Factor 1 | Protein Coding | 51 | GC02M039043 | 0.595085979 |
| TBK1 | TANK Binding Kinase 1 | Protein Coding | 51 | GC12P064451 | 0.595085979 |
| GABBR1 | Gamma-Aminobutyric Acid Type B Receptor Subunit 1 | Protein Coding | 50 | GC06M029555 | 0.595085979 |
| MS4A1 | Membrane Spanning 4-Domains A1 | Protein Coding | 50 | GC11P060520 | 0.595085979 |
| NCF2 | Neutrophil Cytosolic Factor 2 | Protein Coding | 50 | GC01M183855 | 0.595085979 |
| CD27 | CD27 Molecule | Protein Coding | 49 | GC12P022338 | 0.595085979 |
| PRF1 | Perforin 1 | Protein Coding | 49 | GC10M070597 | 0.595085979 |
| BLNK | B Cell Linker | Protein Coding | 48 | GC10M096253 | 0.595085979 |
| CD33 | CD33 Molecule | Protein Coding | 48 | GC19P070285 | 0.595085979 |
| CDC6 | Cell Division Cycle 6 | Protein Coding | 48 | GC17P040287 | 0.595085979 |
| FGF17 | Fibroblast Growth Factor 17 | Protein Coding | 48 | GC08P022042 | 0.595085979 |
| CDKN1C | Cyclin Dependent Kinase Inhibitor 1C | Protein Coding | 47 | GC11M004031 | 0.595085979 |
| CPT1B | Carnitine Palmitoyltransferase 1B | Protein Coding | 47 | GC22M059577 | 0.595085979 |
| DPAGT1 | Dolichyl-Phosphate N-Acetylglucosaminephosphotransferase 1 | Protein Coding | 47 | GC11M119256 | 0.595085979 |
| E2F4 | E2F Transcription Factor 4 | Protein Coding | 47 | GC16P067192 | 0.595085979 |
| LPAR6 | Lysophosphatidic Acid Receptor 6 | Protein Coding | 47 | GC13M048389 | 0.595085979 |
| NTHL1 | Nth Like DNA Glycosylase 1 | Protein Coding | 47 | GC16M007547 | 0.595085979 |
| TBX2 | T-Box Transcription Factor 2 | Protein Coding | 47 | GC17P061399 | 0.595085979 |
| TNKS2 | Tankyrase 2 | Protein Coding | 47 | GC10P091798 | 0.595085979 |
| BCL10 | BCL10 Immune Signaling Adaptor | Protein Coding | 46 | GC01M085265 | 0.595085979 |
| DLL3 | Delta Like Canonical Notch Ligand 3 | Protein Coding | 46 | GC19P039498 | 0.595085979 |
| AIRE | Autoimmune Regulator | Protein Coding | 45 | GC21P044285 | 0.595085979 |
| ALAS1 | 5'-Aminolevulinate Synthase 1 | Protein Coding | 45 | GC03P052198 | 0.595085979 |
| CD5 | CD5 Molecule | Protein Coding | 45 | GC11P061114 | 0.595085979 |
| HS2ST1 | Heparan Sulfate 2-O-Sulfotransferase 1 | Protein Coding | 45 | GC01P086914 | 0.595085979 |
| KCNH6 | Potassium Voltage-Gated Channel Subfamily H Member 6 | Protein Coding | 45 | GC17P063523 | 0.595085979 |
| TIRAP | TIR Domain Containing Adaptor Protein | Protein Coding | 45 | GC11P126284 | 0.595085979 |
| ACAA1 | Acetyl-CoA Acyltransferase 1 | Protein Coding | 44 | GC03M038103 | 0.595085979 |
| G6PC3 | Glucose-6-Phosphatase Catalytic Subunit 3 | Protein Coding | 44 | GC17P044070 | 0.595085979 |
| GFI1B | Growth Factor Independent 1B Transcriptional Repressor | Protein Coding | 44 | GC09P132945 | 0.595085979 |
| LPCAT2 | Lysophosphatidylcholine Acyltransferase 2 | Protein Coding | 44 | GC16P055510 | 0.595085979 |
| PMAIP1 | Phorbol-12-Myristate-13-Acetate-Induced Protein 1 | Protein Coding | 44 | GC18P059899 | 0.595085979 |
| SLC44A2 | Solute Carrier Family 44 Member 2 | Protein Coding | 44 | GC19P010602 | 0.595085979 |
| SOX17 | SRY-Box Transcription Factor 17 | Protein Coding | 44 | GC08P054457 | 0.595085979 |
| SPRY1 | Sprouty RTK Signaling Antagonist 1 | Protein Coding | 44 | GC04P123396 | 0.595085979 |
| TERF1 | Telomeric Repeat Binding Factor 1 | Protein Coding | 44 | GC08P073003 | 0.595085979 |
| TERF2 | Telomeric Repeat Binding Factor 2 | Protein Coding | 44 | GC16M069355 | 0.595085979 |
| ABCF1 | ATP Binding Cassette Subfamily F Member 1 | Protein Coding | 43 | GC06P030571 | 0.595085979 |
| E2F6 | E2F Transcription Factor 6 | Protein Coding | 43 | GC02M011434 | 0.595085979 |
| FRS2 | Fibroblast Growth Factor Receptor Substrate 2 | Protein Coding | 43 | GC12P069471 | 0.595085979 |
| SIGIRR | Single Ig And TIR Domain Containing | Protein Coding | 43 | GC11M003883 | 0.595085979 |
| SOCS5 | Suppressor Of Cytokine Signaling 5 | Protein Coding | 43 | GC02P046698 | 0.595085979 |
| SOCS6 | Suppressor Of Cytokine Signaling 6 | Protein Coding | 43 | GC18P070288 | 0.595085979 |
| TRAF5 | TNF Receptor Associated Factor 5 | Protein Coding | 43 | GC01P211326 | 0.595085979 |
| CD180 | CD180 Molecule | Protein Coding | 42 | GC05M067181 | 0.595085979 |
| MSRB1 | Methionine Sulfoxide Reductase B1 | Protein Coding | 42 | GC16M007534 | 0.595085979 |
| MKRN2 | Makorin Ring Finger Protein 2 | Protein Coding | 41 | GC03P012745 | 0.595085979 |
| RPL3L | Ribosomal Protein L3 Like | Protein Coding | 41 | GC16M001943 | 0.595085979 |
| TEP1 | Telomerase Associated Protein 1 | Protein Coding | 41 | GC14M020365 | 0.595085979 |
| AP1M2 | Adaptor Related Protein Complex 1 Subunit Mu 2 | Protein Coding | 40 | GC19M010572 | 0.595085979 |
| BYSL | Bystin Like | Protein Coding | 40 | GC06P087963 | 0.595085979 |
| GMPR2 | Guanosine Monophosphate Reductase 2 | Protein Coding | 40 | GC14P024232 | 0.595085979 |
| NDUFA3 | NADH:Ubiquinone Oxidoreductase Subunit A3 | Protein Coding | 40 | GC19P054102 | 0.595085979 |
| PRG3 | Proteoglycan 3, Pro Eosinophil Major Basic Protein 2 | Protein Coding | 40 | GC11M057376 | 0.595085979 |
| SELENON | Selenoprotein N | Protein Coding | 40 | GC01P025800 | 0.595085979 |
| TIMMDC1 | Translocase Of Inner Mitochondrial Membrane Domain Containing 1 | Protein Coding | 40 | GC03P119498 | 0.595085979 |
| AFMID | Arylformamidase | Protein Coding | 38 | GC17P078185 | 0.595085979 |
| TMEM230 | Transmembrane Protein 230 | Protein Coding | 38 | GC20M005064 | 0.595085979 |
| TRIM55 | Tripartite Motif Containing 55 | Protein Coding | 38 | GC08P066575 | 0.595085979 |
| NECAB3 | N-Terminal EF-Hand Calcium Binding Protein 3 | Protein Coding | 37 | GC20M033659 | 0.595085979 |
| ACTR8 | Actin Related Protein 8 | Protein Coding | 36 | GC03M053864 | 0.595085979 |
| PRSS50 | Serine Protease 50 | Protein Coding | 36 | GC03M046712 | 0.595085979 |
| RMDN1 | Regulator Of Microtubule Dynamics 1 | Protein Coding | 36 | GC08M086520 | 0.595085979 |
| FLACC1 | Flagellum Associated Containing Coiled-Coil Domains 1 | Protein Coding | 35 | GC02M201441 | 0.595085979 |
| RNF175 | Ring Finger Protein 175 | Protein Coding | 34 | GC04M153710 | 0.595085979 |
| PHAF1 | Phagosome Assembly Factor 1 | Protein Coding | 33 | GC16P067110 | 0.595085979 |
| SPACA9 | Sperm Acrosome Associated 9 | Protein Coding | 33 | GC09P132878 | 0.595085979 |
| PEDS1 | Plasmanylethanolamine Desaturase 1 | Protein Coding | 32 | GC20M050119 | 0.595085979 |
| CARD18 | Caspase Recruitment Domain Family Member 18 | Protein Coding | 31 | GC11M105137 | 0.595085979 |
| GPATCH11 | G-Patch Domain Containing 11 | Protein Coding | 31 | GC02P037084 | 0.595085979 |
| SELENOH | Selenoprotein H | Protein Coding | 31 | GC11P058164 | 0.595085979 |
| SPATA32 | Spermatogenesis Associated 32 | Protein Coding | 30 | GC17M045254 | 0.595085979 |
| TBC1D3 | TBC1 Domain Family Member 3 | Protein Coding | 28 | GC17M038181 | 0.595085979 |
| CDKN2A-DT | CDKN2A Divergent Transcript | RNA Gene | 23 | GC09P021967 | 0.595085979 |
| PTENP1 | Phosphatase And Tensin Homolog Pseudogene 1 | Pseudogene | 22 | GC09M033673 | 0.595085979 |
| BAK1P1 | BCL2 Antagonist/Killer 1 Pseudogene 1 | Pseudogene | 11 | GC20M032688 | 0.595085979 |
| PRKRAP1 | Protein Activator Of Interferon Induced Protein Kinase EIF2AK2 Pseudogene 1 | Pseudogene | 5 | GC06Po03930 | 0.595085979 |
| ST14 | ST14 Transmembrane Serine Protease Matriptase | Protein Coding | 51 | GC11P130159 | 0.588083982 |
| SDCBP | Syndecan Binding Protein | Protein Coding | 44 | GC08P058539 | 0.588083982 |
| TPD52 | Tumor Protein D52 | Protein Coding | 43 | GC08M080034 | 0.588083982 |
| DKK1 | Dickkopf WNT Signaling Pathway Inhibitor 1 | Protein Coding | 50 | GC10P052314 | 0.587569654 |
| SLC16A1 | Solute Carrier Family 16 Member 1 | Protein Coding | 50 | GC01M112998 | 0.587569654 |
| APAF1 | Apoptotic Peptidase Activating Factor 1 | Protein Coding | 48 | GC12P098645 | 0.587569654 |
| CLCN3 | Chloride Voltage-Gated Channel 3 | Protein Coding | 47 | GC04P169612 | 0.587569654 |
| COL4A6 | Collagen Type IV Alpha 6 Chain | Protein Coding | 47 | GC0XM108155 | 0.587569654 |
| CCNA1 | Cyclin A1 | Protein Coding | 44 | GC13P036431 | 0.587569654 |
| MIR29C | MicroRNA 29c | RNA Gene | 20 | GC01M207802 | 0.587569654 |
| GZMB | Granzyme B | Protein Coding | 50 | GC14M024630 | 0.585711002 |
| TACR2 | Tachykinin Receptor 2 | Protein Coding | 48 | GC10M069403 | 0.585711002 |
| DPP10 | Dipeptidyl Peptidase Like 10 | Protein Coding | 45 | GC02P114442 | 0.585711002 |
| CLC | Charcot-Leyden Crystal Galectin | Protein Coding | 41 | GC19M067840 | 0.585711002 |
| RNASE2 | Ribonuclease A Family Member 2 | Protein Coding | 41 | GC14P033417 | 0.585711002 |
| BCAN | Brevican | Protein Coding | 44 | GC01P156641 | 0.581453681 |
| NFAT5 | Nuclear Factor Of Activated T Cells 5 | Protein Coding | 44 | GC16P069565 | 0.581453681 |
| OXTR | Oxytocin Receptor | Protein Coding | 50 | GC03M008767 | 0.580192566 |
| PRSS8 | Serine Protease 8 | Protein Coding | 47 | GC16M037886 | 0.578577518 |
| NCOR2 | Nuclear Receptor Corepressor 2 | Protein Coding | 46 | GC12M124324 | 0.578577518 |
| MX1 | MX Dynamin Like GTPase 1 | Protein Coding | 43 | GC21P041420 | 0.578577518 |
| SNHG15 | Small Nucleolar RNA Host Gene 15 | RNA Gene | 23 | GC07M044983 | 0.578577518 |
| GBP1 | Guanylate Binding Protein 1 | Protein Coding | 43 | GC01M089052 | 0.576209843 |
| SASH1 | SAM And SH3 Domain Containing 1 | Protein Coding | 42 | GC06P148193 | 0.576209843 |
| GAS5 | Growth Arrest Specific 5 | RNA Gene | 26 | GC01M173947 | 0.576209843 |
| MIR200A | MicroRNA 200a | RNA Gene | 22 | GC01P004110 | 0.576209843 |
| COL9A3 | Collagen Type IX Alpha 3 Chain | Protein Coding | 45 | GC20P062816 | 0.571296096 |
| WFDC2 | WAP Four-Disulfide Core Domain 2 | Protein Coding | 40 | GC20P045469 | 0.571296096 |
| DICER1 | Dicer 1, Ribonuclease III | Protein Coding | 51 | GC14M095086 | 0.567376852 |
| CRYAB | Crystallin Alpha B | Protein Coding | 48 | GC11M111908 | 0.567376852 |
| MFGE8 | Milk Fat Globule EGF And Factor V/VIII Domain Containing | Protein Coding | 47 | GC15M088898 | 0.567376852 |
| MIR10B | MicroRNA 10b | RNA Gene | 23 | GC02P176150 | 0.567376852 |
| MIR218-1 | MicroRNA 218-1 | RNA Gene | 20 | GC04P020866 | 0.567376852 |
| TF | Transferrin | Protein Coding | 53 | GC03P134678 | 0.565516591 |
| BDKRB2 | Bradykinin Receptor B2 | Protein Coding | 48 | GC14P096205 | 0.565516591 |
| GYPE | Glycophorin E (MNS Blood Group) | Protein Coding | 32 | GC04M143870 | 0.554266453 |
| MIR181D | MicroRNA 181d | RNA Gene | 19 | GC19P013874 | 0.554266453 |
| ABCG2 | ATP Binding Cassette Subfamily G Member 2 (Junior Blood Group) | Protein Coding | 52 | GC04M088090 | 0.553040385 |
| SCARB1 | Scavenger Receptor Class B Member 1 | Protein Coding | 50 | GC12M124776 | 0.553040385 |
| APOC3 | Apolipoprotein C3 | Protein Coding | 48 | GC11P116829 | 0.553040385 |
| LPAR2 | Lysophosphatidic Acid Receptor 2 | Protein Coding | 48 | GC19M019624 | 0.553040385 |
| APOL1 | Apolipoprotein L1 | Protein Coding | 47 | GC22P036253 | 0.553040385 |
| RAB5A | RAB5A, Member RAS Oncogene Family | Protein Coding | 47 | GC03P019948 | 0.553040385 |
| ADGRE5 | Adhesion G Protein-Coupled Receptor E5 | Protein Coding | 45 | GC19P014671 | 0.553040385 |
| CRK | CRK Proto-Oncogene, Adaptor Protein | Protein Coding | 45 | GC17M001420 | 0.553040385 |
| PROX1 | Prospero Homeobox 1 | Protein Coding | 45 | GC01P213983 | 0.553040385 |
| TNFRSF18 | TNF Receptor Superfamily Member 18 | Protein Coding | 44 | GC01M001203 | 0.553040385 |
| IL37 | Interleukin 37 | Protein Coding | 40 | GC02P123135 | 0.553040385 |
| PI3 | Peptidase Inhibitor 3 | Protein Coding | 40 | GC20P045174 | 0.553040385 |
| MIR139 | MicroRNA 139 | RNA Gene | 23 | GC11M072615 | 0.553040385 |
| MIR142 | MicroRNA 142 | RNA Gene | 23 | GC17M058331 | 0.553040385 |
| ZFAS1 | ZNFX1 Antisense RNA 1 | RNA Gene | 22 | GC20P049276 | 0.553040385 |
| PRG4 | Proteoglycan 4 | Protein Coding | 43 | GC01P186296 | 0.549890816 |
| MLH1 | MutL Homolog 1 | Protein Coding | 51 | GC03P036993 | 0.549022615 |
| HOXA11 | Homeobox A11 | Protein Coding | 46 | GC07M027632 | 0.549022615 |
| ENPP1 | Ectonucleotide Pyrophosphatase/Phosphodiesterase 1 | Protein Coding | 51 | GC06P131808 | 0.544456899 |
| GDF5 | Growth Differentiation Factor 5 | Protein Coding | 48 | GC20M035433 | 0.544456899 |
| TRPM7 | Transient Receptor Potential Cation Channel Subfamily M Member 7 | Protein Coding | 46 | GC15M050552 | 0.544456899 |
| BAG3 | BAG Cochaperone 3 | Protein Coding | 45 | GC10P119651 | 0.544456899 |
| TFF1 | Trefoil Factor 1 | Protein Coding | 45 | GC21M042362 | 0.544456899 |
| DKK3 | Dickkopf WNT Signaling Pathway Inhibitor 3 | Protein Coding | 44 | GC11M011962 | 0.544456899 |
| UTS2 | Urotensin 2 | Protein Coding | 42 | GC01M007843 | 0.544456899 |
| CDCP1 | CUB Domain Containing Protein 1 | Protein Coding | 41 | GC03M045082 | 0.544456899 |
| IL20 | Interleukin 20 | Protein Coding | 41 | GC01P206866 | 0.544456899 |
| MIR490 | MicroRNA 490 | RNA Gene | 19 | GC07P136903 | 0.544456899 |
| UCA1 | Urothelial Cancer Associated 1 | RNA Gene | 25 | GC19P015828 | 0.543146431 |
| BGN | Biglycan | Protein Coding | 47 | GC0XP153494 | 0.536841393 |
| PPM1A | Protein Phosphatase, Mg2+/Mn2+ Dependent 1A | Protein Coding | 49 | GC14P060245 | 0.53310293 |
| COL4A5 | Collagen Type IV Alpha 5 Chain | Protein Coding | 47 | GC0XP108439 | 0.53310293 |
| FABP3 | Fatty Acid Binding Protein 3 | Protein Coding | 46 | GC01M031365 | 0.53310293 |
| KLRK1 | Killer Cell Lectin Like Receptor K1 | Protein Coding | 44 | GC12M021843 | 0.53310293 |
| DEFA5 | Defensin Alpha 5 | Protein Coding | 38 | GC08M007057 | 0.53310293 |
| DNM2 | Dynamin 2 | Protein Coding | 52 | GC19P010718 | 0.532847524 |
| HSP90AB1 | Heat Shock Protein 90 Alpha Family Class B Member 1 | Protein Coding | 50 | GC06P044246 | 0.532847524 |
| F2RL3 | F2R Like Thrombin Or Trypsin Receptor 3 | Protein Coding | 49 | GC19P016888 | 0.532847524 |
| GNB3 | G Protein Subunit Beta 3 | Protein Coding | 49 | GC12P006839 | 0.532847524 |
| ATF1 | Activating Transcription Factor 1 | Protein Coding | 48 | GC12P050763 | 0.532847524 |
| OGT | O-Linked N-Acetylglucosamine (GlcNAc) Transferase | Protein Coding | 47 | GC0XP071534 | 0.532847524 |
| NODAL | Nodal Growth Differentiation Factor | Protein Coding | 46 | GC10M070431 | 0.532847524 |
| COPS5 | COP9 Signalosome Subunit 5 | Protein Coding | 44 | GC08M067043 | 0.532847524 |
| FABP2 | Fatty Acid Binding Protein 2 | Protein Coding | 44 | GC04M119317 | 0.532847524 |
| SLC37A4 | Solute Carrier Family 37 Member 4 | Protein Coding | 44 | GC11M119236 | 0.532847524 |
| TNFRSF6B | TNF Receptor Superfamily Member 6b | Protein Coding | 44 | GC20P063696 | 0.532847524 |
| MRTFA | Myocardin Related Transcription Factor A | Protein Coding | 43 | GC22M060093 | 0.532847524 |
| RARRES2 | Retinoic Acid Receptor Responder 2 | Protein Coding | 42 | GC07M150333 | 0.532847524 |
| APLN | Apelin | Protein Coding | 41 | GC0XM129645 | 0.532847524 |
| ARMS2 | Age-Related Maculopathy Susceptibility 2 | Protein Coding | 35 | GC10P122454 | 0.532847524 |
| PLCG2 | Phospholipase C Gamma 2 | Protein Coding | 54 | GC16P081773 | 0.518835306 |
| HADHB | Hydroxyacyl-CoA Dehydrogenase Trifunctional Multienzyme Complex Subunit Beta | Protein Coding | 50 | GC02P026243 | 0.518835306 |
| MALT1 | MALT1 Paracaspase | Protein Coding | 50 | GC18P058671 | 0.518835306 |
| RUVBL2 | RuvB Like AAA ATPase 2 | Protein Coding | 48 | GC19P048993 | 0.518835306 |
| LOXL3 | Lysyl Oxidase Like 3 | Protein Coding | 44 | GC02M074532 | 0.518835306 |
| CD1C | CD1c Molecule | Protein Coding | 41 | GC01P158289 | 0.518835306 |
| GYPB | Glycophorin B (MNS Blood Group) | Protein Coding | 41 | GC04M143996 | 0.518835306 |
| RAET1E | Retinoic Acid Early Transcript 1E | Protein Coding | 38 | GC06M149883 | 0.518835306 |
| MT1H | Metallothionein 1H | Protein Coding | 37 | GC16P057086 | 0.518835306 |
| MIR28 | MicroRNA 28 | RNA Gene | 20 | GC03P188688 | 0.518835306 |
| PLD2 | Phospholipase D2 | Protein Coding | 50 | GC17P004808 | 0.517269671 |
| CRKL | CRK Like Proto-Oncogene, Adaptor Protein | Protein Coding | 49 | GC22P020917 | 0.517269671 |
| ETV6 | ETS Variant Transcription Factor 6 | Protein Coding | 48 | GC12P011649 | 0.517269671 |
| RHOB | Ras Homolog Family Member B | Protein Coding | 47 | GC02P020447 | 0.517269671 |
| FADS1 | Fatty Acid Desaturase 1 | Protein Coding | 46 | GC11M061799 | 0.517269671 |
| GIT1 | GIT ArfGAP 1 | Protein Coding | 45 | GC17M029573 | 0.517269671 |
| CNMD | Chondromodulin | Protein Coding | 40 | GC13M052704 | 0.517269671 |
| MIR125B1 | MicroRNA 125b-1 | RNA Gene | 23 | GC11M122100 | 0.517269671 |
| DNMT3B | DNA Methyltransferase 3 Beta | Protein Coding | 53 | GC20P032762 | 0.514847219 |
| CYP1B1 | Cytochrome P450 Family 1 Subfamily B Member 1 | Protein Coding | 50 | GC02M038066 | 0.514847219 |
| AQP2 | Aquaporin 2 | Protein Coding | 49 | GC12P049950 | 0.514847219 |
| SOST | Sclerostin | Protein Coding | 49 | GC17M043753 | 0.514847219 |
| ARRB1 | Arrestin Beta 1 | Protein Coding | 47 | GC11M097430 | 0.514847219 |
| STMN1 | Stathmin 1 | Protein Coding | 47 | GC01M025884 | 0.514847219 |
| RACK1 | Receptor For Activated C Kinase 1 | Protein Coding | 45 | GC05M181873 | 0.514847219 |
| CTHRC1 | Collagen Triple Helix Repeat Containing 1 | Protein Coding | 44 | GC08P103371 | 0.514847219 |
| MUC4 | Mucin 4, Cell Surface Associated | Protein Coding | 42 | GC03M195746 | 0.514847219 |
| SFRP5 | Secreted Frizzled Related Protein 5 | Protein Coding | 41 | GC10M097766 | 0.514847219 |
| IFNL1 | Interferon Lambda 1 | Protein Coding | 40 | GC19P039296 | 0.514847219 |
| MIR330 | MicroRNA 330 | RNA Gene | 22 | GC19M068020 | 0.514847219 |
| KDM1A | Lysine Demethylase 1A | Protein Coding | 51 | GC01P023019 | 0.50992763 |
| WNT5B | Wnt Family Member 5B | Protein Coding | 47 | GC12P001529 | 0.50992763 |
| GAB1 | GRB2 Associated Binding Protein 1 | Protein Coding | 46 | GC04P143336 | 0.50992763 |
| PON2 | Paraoxonase 2 | Protein Coding | 46 | GC07M095404 | 0.50992763 |
| SEL1L | SEL1L Adaptor Subunit Of ERAD E3 Ubiquitin Ligase | Protein Coding | 45 | GC14M081471 | 0.50992763 |
| CEP55 | Centrosomal Protein 55 | Protein Coding | 43 | GC10P093496 | 0.50992763 |
| MIR378A | MicroRNA 378a | RNA Gene | 23 | GC05P149732 | 0.50992763 |
| MIR24-1 | MicroRNA 24-1 | RNA Gene | 21 | GC09P095086 | 0.50992763 |
| MIR181A1 | MicroRNA 181a-1 | RNA Gene | 20 | GC01M198860 | 0.50992763 |
| SEMA4D | Semaphorin 4D | Protein Coding | 50 | GC09M089360 | 0.506539583 |
| SFRP2 | Secreted Frizzled Related Protein 2 | Protein Coding | 43 | GC04M153780 | 0.506539583 |
| MACC1 | MET Transcriptional Regulator MACC1 | Protein Coding | 38 | GC07M020140 | 0.506539583 |
| MIR19A | MicroRNA 19a | RNA Gene | 23 | GC13P091566 | 0.506539583 |
| ITK | IL2 Inducible T Cell Kinase | Protein Coding | 55 | GC05P157158 | 0.500172734 |
| FCGR2B | Fc Gamma Receptor IIb | Protein Coding | 51 | GC01P161779 | 0.500172734 |
| PTGDR | Prostaglandin D2 Receptor | Protein Coding | 50 | GC14P052267 | 0.500172734 |
| STAT4 | Signal Transducer And Activator Of Transcription 4 | Protein Coding | 50 | GC02M191029 | 0.500172734 |
| HRH2 | Histamine Receptor H2 | Protein Coding | 49 | GC05P175659 | 0.500172734 |
| IRAK3 | Interleukin 1 Receptor Associated Kinase 3 | Protein Coding | 49 | GC12P066289 | 0.500172734 |
| ARG2 | Arginase 2 | Protein Coding | 48 | GC14P067619 | 0.500172734 |
| TBX21 | T-Box Transcription Factor 21 | Protein Coding | 48 | GC17P047733 | 0.500172734 |
| CHIT1 | Chitinase 1 | Protein Coding | 47 | GC01M203213 | 0.500172734 |
| HDC | Histidine Decarboxylase | Protein Coding | 47 | GC15M050241 | 0.500172734 |
| PDE4B | Phosphodiesterase 4B | Protein Coding | 47 | GC01P065792 | 0.500172734 |
| SFTPB | Surfactant Protein B | Protein Coding | 47 | GC02M085657 | 0.500172734 |
| PDE3B | Phosphodiesterase 3B | Protein Coding | 46 | GC11P014643 | 0.500172734 |
| FCER1A | Fc Epsilon Receptor Ia | Protein Coding | 45 | GC01P159259 | 0.500172734 |
| MS4A2 | Membrane Spanning 4-Domains A2 | Protein Coding | 45 | GC11P060088 | 0.500172734 |
| SLC7A2 | Solute Carrier Family 7 Member 2 | Protein Coding | 45 | GC08P017497 | 0.500172734 |
| AOC1 | Amine Oxidase Copper Containing 1 | Protein Coding | 44 | GC07P150824 | 0.500172734 |
| CHIA | Chitinase Acidic | Protein Coding | 44 | GC01P111291 | 0.500172734 |
| TLR10 | Toll Like Receptor 10 | Protein Coding | 41 | GC04M038773 | 0.500172734 |
| RASGRP4 | RAS Guanyl Releasing Protein 4 | Protein Coding | 40 | GC19M038409 | 0.500172734 |
| CDHR3 | Cadherin Related Family Member 3 | Protein Coding | 39 | GC07P105876 | 0.500172734 |
| COL26A1 | Collagen Type XXVI Alpha 1 Chain | Protein Coding | 38 | GC07P101362 | 0.500172734 |
| DENND1B | DENN Domain Containing 1B | Protein Coding | 38 | GC01M197473 | 0.500172734 |
| ACP4 | Acid Phosphatase 4 | Protein Coding | 37 | GC19P070264 | 0.500172734 |
| FGFBP2 | Fibroblast Growth Factor Binding Protein 2 | Protein Coding | 37 | GC04M015961 | 0.500172734 |
| SPATS2L | Spermatogenesis Associated Serine Rich 2 Like | Protein Coding | 37 | GC02P200305 | 0.500172734 |
| AMTN | Amelotin | Protein Coding | 36 | GC04P070518 | 0.500172734 |
| ODAPH | Odontogenesis Associated Phosphoprotein | Protein Coding | 34 | GC04P075562 | 0.500172734 |
| NPSR1-AS1 | NPSR1 Antisense RNA 1 | RNA Gene | 21 | GC07M034387 | 0.500172734 |
| IGES | Immunoglobulin E Concentration, Serum | Genetic Locus | 4 | GC05U990033 | 0.500172734 |
| HDAC6 | Histone Deacetylase 6 | Protein Coding | 56 | GC0XP048801 | 0.494654357 |
| DES | Desmin | Protein Coding | 51 | GC02P219418 | 0.494654357 |
| PLD1 | Phospholipase D1 | Protein Coding | 51 | GC03M171600 | 0.494654357 |
| TUBB3 | Tubulin Beta 3 Class III | Protein Coding | 51 | GC16P091470 | 0.494654357 |
| CSK | C-Terminal Src Kinase | Protein Coding | 50 | GC15P074782 | 0.494654357 |
| YWHAB | Tyrosine 3-Monooxygenase/Tryptophan 5-Monooxygenase Activation Protein Beta | Protein Coding | 50 | GC20P044885 | 0.494654357 |
| BECN1 | Beclin 1 | Protein Coding | 48 | GC17M042810 | 0.494654357 |
| EPHB6 | EPH Receptor B6 | Protein Coding | 48 | GC07P148810 | 0.494654357 |
| PTGES3 | Prostaglandin E Synthase 3 | Protein Coding | 47 | GC12M056974 | 0.494654357 |
| GAB2 | GRB2 Associated Binding Protein 2 | Protein Coding | 45 | GC11M078215 | 0.494654357 |
| PLXNB1 | Plexin B1 | Protein Coding | 45 | GC03M048403 | 0.494654357 |
| RXRG | Retinoid X Receptor Gamma | Protein Coding | 45 | GC01M165401 | 0.494654357 |
| SLC3A2 | Solute Carrier Family 3 Member 2 | Protein Coding | 45 | GC11P062856 | 0.494654357 |
| COP1 | COP1 E3 Ubiquitin Ligase | Protein Coding | 43 | GC01M175944 | 0.494654357 |
| ADGRB1 | Adhesion G Protein-Coupled Receptor B1 | Protein Coding | 40 | GC08P142449 | 0.494654357 |
| EFS | Embryonal Fyn-Associated Substrate | Protein Coding | 37 | GC14M023356 | 0.494654357 |
| RLN3 | Relaxin 3 | Protein Coding | 37 | GC19P014654 | 0.494654357 |
| HOXA-AS2 | HOXA Cluster Antisense RNA 2 | RNA Gene | 20 | GC07P027107 | 0.494654357 |
| MIR874 | MicroRNA 874 | RNA Gene | 20 | GC05M137647 | 0.494654357 |
| MIR519D | MicroRNA 519d | RNA Gene | 19 | GC19P053713 | 0.494654357 |
| GRHL2-DT | GRHL2 Divergent Transcript | RNA Gene | 8 | GC08M101492 | 0.494654357 |
| F10 | Coagulation Factor X | Protein Coding | 53 | GC13P113122 | 0.489751726 |
| FST | Follistatin | Protein Coding | 51 | GC05P053480 | 0.489751726 |
| DSG2 | Desmoglein 2 | Protein Coding | 48 | GC18P031498 | 0.489751726 |
| DLG1 | Discs Large MAGUK Scaffold Protein 1 | Protein Coding | 46 | GC03M197042 | 0.485418856 |
| NTN1 | Netrin 1 | Protein Coding | 48 | GC17P011538 | 0.482740402 |
| NFATC2 | Nuclear Factor Of Activated T Cells 2 | Protein Coding | 47 | GC20M051386 | 0.482740402 |
| RAP1A | RAP1A, Member Of RAS Oncogene Family | Protein Coding | 47 | GC01P111542 | 0.482740402 |
| BCL2L2 | BCL2 Like 2 | Protein Coding | 45 | GC14P033738 | 0.482740402 |
| RXFP2 | Relaxin Family Peptide Receptor 2 | Protein Coding | 45 | GC13P031739 | 0.482740402 |
| KRT7 | Keratin 7 | Protein Coding | 44 | GC12P052232 | 0.482740402 |
| PPP1R9B | Protein Phosphatase 1 Regulatory Subunit 9B | Protein Coding | 43 | GC17M050133 | 0.482740402 |
| RAB8A | RAB8A, Member RAS Oncogene Family | Protein Coding | 42 | GC19P016111 | 0.482740402 |
| STOML2 | Stomatin Like 2 | Protein Coding | 42 | GC09M035099 | 0.482740402 |
| TRO | Trophinin | Protein Coding | 38 | GC0XP054920 | 0.482740402 |
| MIR200C | MicroRNA 200c | RNA Gene | 26 | GC12P022384 | 0.482740402 |
| MIR429 | MicroRNA 429 | RNA Gene | 22 | GC01P004111 | 0.482740402 |
| RRM2 | Ribonucleotide Reductase Regulatory Subunit M2 | Protein Coding | 51 | GC02P010123 | 0.481838584 |
| RBP4 | Retinol Binding Protein 4 | Protein Coding | 48 | GC10M093591 | 0.481838584 |
| BDKRB1 | Bradykinin Receptor B1 | Protein Coding | 47 | GC14P096299 | 0.481838584 |
| RNF213 | Ring Finger Protein 213 | Protein Coding | 41 | GC17P080260 | 0.481838584 |
| RAB40B | RAB40B, Member RAS Oncogene Family | Protein Coding | 38 | GC17M082654 | 0.481838584 |
| PRKACA | Protein Kinase CAMP-Activated Catalytic Subunit Alpha | Protein Coding | 54 | GC19M014559 | 0.471734464 |
| NOTCH3 | Notch Receptor 3 | Protein Coding | 53 | GC19M015159 | 0.471734464 |
| VLDLR | Very Low Density Lipoprotein Receptor | Protein Coding | 52 | GC09P002611 | 0.471734464 |
| ALDOA | Aldolase, Fructose-Bisphosphate A | Protein Coding | 51 | GC16P030064 | 0.471734464 |
| ADRA1A | Adrenoceptor Alpha 1A | Protein Coding | 50 | GC08M026747 | 0.471734464 |
| ARSB | Arylsulfatase B | Protein Coding | 49 | GC05M078777 | 0.471734464 |
| SIRPA | Signal Regulatory Protein Alpha | Protein Coding | 47 | GC20P001894 | 0.471734464 |
| SLC7A5 | Solute Carrier Family 7 Member 5 | Protein Coding | 47 | GC16M087830 | 0.471734464 |
| TDGF1 | Teratocarcinoma-Derived Growth Factor 1 | Protein Coding | 47 | GC03P047198 | 0.471734464 |
| KRT10 | Keratin 10 | Protein Coding | 45 | GC17M040818 | 0.471734464 |
| CLIC4 | Chloride Intracellular Channel 4 | Protein Coding | 44 | GC01P024745 | 0.471734464 |
| NPPC | Natriuretic Peptide C | Protein Coding | 44 | GC02M231921 | 0.471734464 |
| TWIST2 | Twist Family BHLH Transcription Factor 2 | Protein Coding | 44 | GC02P238848 | 0.471734464 |
| CLDN6 | Claudin 6 | Protein Coding | 43 | GC16M003014 | 0.471734464 |
| PIAS3 | Protein Inhibitor Of Activated STAT 3 | Protein Coding | 43 | GC01M145848 | 0.471734464 |
| RND3 | Rho Family GTPase 3 | Protein Coding | 43 | GC02M150468 | 0.471734464 |
| TAGLN2 | Transgelin 2 | Protein Coding | 43 | GC01M159918 | 0.471734464 |
| RTCB | RNA 2',3'-Cyclic Phosphate And 5'-OH Ligase | Protein Coding | 41 | GC22M032387 | 0.471734464 |
| IL27 | Interleukin 27 | Protein Coding | 40 | GC16M037582 | 0.471734464 |
| PEG3 | Paternally Expressed 3 | Protein Coding | 40 | GC19M056810 | 0.471734464 |
| CNKSR1 | Connector Enhancer Of Kinase Suppressor Of Ras 1 | Protein Coding | 39 | GC01P026178 | 0.471734464 |
| MIR149 | MicroRNA 149 | RNA Gene | 25 | GC02P240456 | 0.471734464 |
| MIR26B | MicroRNA 26b | RNA Gene | 24 | GC02P218402 | 0.471734464 |
| MIR191 | MicroRNA 191 | RNA Gene | 23 | GC03M051371 | 0.471734464 |
| MIR197 | MicroRNA 197 | RNA Gene | 22 | GC01P109549 | 0.471734464 |
| MIR15B | MicroRNA 15b | RNA Gene | 21 | GC03P160404 | 0.471734464 |
| MIR338 | MicroRNA 338 | RNA Gene | 20 | GC17M081126 | 0.471734464 |
| MIR495 | MicroRNA 495 | RNA Gene | 20 | GC14P110456 | 0.471734464 |
| MIR382 | MicroRNA 382 | RNA Gene | 18 | GC14P110445 | 0.471734464 |
| EDNRB | Endothelin Receptor Type B | Protein Coding | 52 | GC13M077895 | 0.471495986 |
| CDC25A | Cell Division Cycle 25A | Protein Coding | 50 | GC03M048183 | 0.471495986 |
| DDIT3 | DNA Damage Inducible Transcript 3 | Protein Coding | 48 | GC12M057516 | 0.471495986 |
| F2RL2 | Coagulation Factor II Thrombin Receptor Like 2 | Protein Coding | 46 | GC05M076615 | 0.471495986 |
| ZBTB16 | Zinc Finger And BTB Domain Containing 16 | Protein Coding | 45 | GC11P114059 | 0.471495986 |
| CEACAM6 | CEA Cell Adhesion Molecule 6 | Protein Coding | 44 | GC19P041750 | 0.471495986 |
| IL18BP | Interleukin 18 Binding Protein | Protein Coding | 44 | GC11P071998 | 0.471495986 |
| NINJ1 | Ninjurin 1 | Protein Coding | 43 | GC09M093121 | 0.471495986 |
| TUG1 | Taurine Up-Regulated 1 | Protein Coding | 25 | GC22P030969 | 0.471495986 |
| ID2 | Inhibitor Of DNA Binding 2 | Protein Coding | 46 | GC02P008678 | 0.470742822 |
| GAST | Gastrin | Protein Coding | 41 | GC17P041712 | 0.470742822 |
| CPB2 | Carboxypeptidase B2 | Protein Coding | 48 | GC13M046053 | 0.457608193 |
| LLGL1 | LLGL Scribble Cell Polarity Complex Component 1 | Protein Coding | 42 | GC17P018225 | 0.457608193 |
| SERPINC1 | Serpin Family C Member 1 | Protein Coding | 52 | GC01M174593 | 0.455117047 |
| TREM2 | Triggering Receptor Expressed On Myeloid Cells 2 | Protein Coding | 47 | GC06M069402 | 0.455117047 |
| HPN | Hepsin | Protein Coding | 46 | GC19P035040 | 0.455117047 |
| NTRK3 | Neurotrophic Receptor Tyrosine Kinase 3 | Protein Coding | 55 | GC15M087859 | 0.451303154 |
| JAG1 | Jagged Canonical Notch Ligand 1 | Protein Coding | 54 | GC20M010637 | 0.451303154 |
| TNFRSF10B | TNF Receptor Superfamily Member 10b | Protein Coding | 52 | GC08M023020 | 0.451303154 |
| PROS1 | Protein S | Protein Coding | 51 | GC03M093873 | 0.451303154 |
| KLK3 | Kallikrein Related Peptidase 3 | Protein Coding | 50 | GC19P050854 | 0.451303154 |
| FKBP1A | FKBP Prolyl Isomerase 1A | Protein Coding | 49 | GC20M001369 | 0.451303154 |
| GPC3 | Glypican 3 | Protein Coding | 49 | GC0XM133535 | 0.451303154 |
| SEMA3A | Semaphorin 3A | Protein Coding | 49 | GC07M083955 | 0.451303154 |
| PAFAH1B1 | Platelet Activating Factor Acetylhydrolase 1b Regulatory Subunit 1 | Protein Coding | 48 | GC17P002593 | 0.451303154 |
| PRODH | Proline Dehydrogenase 1 | Protein Coding | 48 | GC22M018912 | 0.451303154 |
| PTGIS | Prostaglandin I2 Synthase | Protein Coding | 48 | GC20M049503 | 0.451303154 |
| TAT | Tyrosine Aminotransferase | Protein Coding | 48 | GC16M071565 | 0.451303154 |
| TET2 | Tet Methylcytosine Dioxygenase 2 | Protein Coding | 48 | GC04P105145 | 0.451303154 |
| AP2M1 | Adaptor Related Protein Complex 2 Subunit Mu 1 | Protein Coding | 47 | GC03P184174 | 0.451303154 |
| RELN | Reelin | Protein Coding | 47 | GC07M103471 | 0.451303154 |
| TNFSF13 | TNF Superfamily Member 13 | Protein Coding | 47 | GC17P007558 | 0.451303154 |
| APOD | Apolipoprotein D | Protein Coding | 45 | GC03M195568 | 0.451303154 |
| G6PC1 | Glucose-6-Phosphatase Catalytic Subunit 1 | Protein Coding | 45 | GC17P059240 | 0.451303154 |
| LAMP1 | Lysosomal Associated Membrane Protein 1 | Protein Coding | 45 | GC13P113297 | 0.451303154 |
| PTRH2 | Peptidyl-TRNA Hydrolase 2 | Protein Coding | 45 | GC17M059674 | 0.451303154 |
| S100A11 | S100 Calcium Binding Protein A11 | Protein Coding | 45 | GC01M152032 | 0.451303154 |
| SOX4 | SRY-Box Transcription Factor 4 | Protein Coding | 45 | GC06P021593 | 0.451303154 |
| KRT3 | Keratin 3 | Protein Coding | 43 | GC12M052789 | 0.451303154 |
| RARRES1 | Retinoic Acid Receptor Responder 1 | Protein Coding | 43 | GC03M158696 | 0.451303154 |
| MUC2 | Mucin 2, Oligomeric Mucus/Gel-Forming | Protein Coding | 41 | GC11P001074 | 0.451303154 |
| STRAP | Serine/Threonine Kinase Receptor Associated Protein | Protein Coding | 41 | GC12P015882 | 0.451303154 |
| SPARCL1 | SPARC Like 1 | Protein Coding | 40 | GC04M087473 | 0.451303154 |
| VSX1 | Visual System Homeobox 1 | Protein Coding | 40 | GC20M025070 | 0.451303154 |
| CEMIP | Cell Migration Inducing Hyaluronidase 1 | Protein Coding | 39 | GC15P080779 | 0.451303154 |
| HULC | Hepatocellular Carcinoma Up-Regulated Long Non-Coding RNA | RNA Gene | 23 | GC06P008438 | 0.451303154 |
| MIR141 | MicroRNA 141 | RNA Gene | 23 | GC12P022385 | 0.451303154 |
| MIR26A1 | MicroRNA 26a-1 | RNA Gene | 23 | GC03P037969 | 0.451303154 |
| FPR1 | Formyl Peptide Receptor 1 | Protein Coding | 50 | GC19M051745 | 0.447309315 |
| HK2 | Hexokinase 2 | Protein Coding | 48 | GC02P074833 | 0.447309315 |
| KRT5 | Keratin 5 | Protein Coding | 48 | GC12M052514 | 0.447309315 |
| NKX2-1 | NK2 Homeobox 1 | Protein Coding | 48 | GC14M036516 | 0.447309315 |
| BID | BH3 Interacting Domain Death Agonist | Protein Coding | 47 | GC22M017734 | 0.447309315 |
| VAV3 | Vav Guanine Nucleotide Exchange Factor 3 | Protein Coding | 45 | GC01M107571 | 0.447309315 |
| PINX1 | PIN2 (TERF1) Interacting Telomerase Inhibitor 1 | Protein Coding | 41 | GC08M010764 | 0.447309315 |
| DERL1 | Derlin 1 | Protein Coding | 38 | GC08M123013 | 0.447309315 |
| HOXA11-AS | HOXA11 Antisense RNA | RNA Gene | 23 | GC07P027184 | 0.447309315 |
| PCSK9 | Proprotein Convertase Subtilisin/Kexin Type 9 | Protein Coding | 54 | GC01P055039 | 0.444547206 |
| BMPR1A | Bone Morphogenetic Protein Receptor Type 1A | Protein Coding | 53 | GC10P093501 | 0.444547206 |
| SMO | Smoothened, Frizzled Class Receptor | Protein Coding | 52 | GC07P132235 | 0.444547206 |
| YWHAG | Tyrosine 3-Monooxygenase/Tryptophan 5-Monooxygenase Activation Protein Gamma | Protein Coding | 51 | GC07M076957 | 0.444547206 |
| C1QA | Complement C1q A Chain | Protein Coding | 49 | GC01P022636 | 0.444547206 |
| CASP4 | Caspase 4 | Protein Coding | 49 | GC11M104942 | 0.444547206 |
| ADRA2A | Adrenoceptor Alpha 2A | Protein Coding | 48 | GC10P111077 | 0.444547206 |
| ERG | ETS Transcription Factor ERG | Protein Coding | 48 | GC21M038367 | 0.444547206 |
| ADIPOR1 | Adiponectin Receptor 1 | Protein Coding | 47 | GC01M202940 | 0.444547206 |
| DCX | Doublecortin | Protein Coding | 47 | GC0XM111293 | 0.444547206 |
| DLC1 | DLC1 Rho GTPase Activating Protein | Protein Coding | 47 | GC08M013083 | 0.444547206 |
| MAP3K20 | Mitogen-Activated Protein Kinase Kinase Kinase 20 | Protein Coding | 47 | GC02P173076 | 0.444547206 |
| PLCE1 | Phospholipase C Epsilon 1 | Protein Coding | 47 | GC10P093993 | 0.444547206 |
| SENP1 | SUMO Specific Peptidase 1 | Protein Coding | 47 | GC12M048042 | 0.444547206 |
| CRABP2 | Cellular Retinoic Acid Binding Protein 2 | Protein Coding | 46 | GC01M156701 | 0.444547206 |
| CLIC1 | Chloride Intracellular Channel 1 | Protein Coding | 45 | GC06M069220 | 0.444547206 |
| GREM1 | Gremlin 1, DAN Family BMP Antagonist | Protein Coding | 45 | GC15P044385 | 0.444547206 |
| ING1 | Inhibitor Of Growth Family Member 1 | Protein Coding | 45 | GC13P110712 | 0.444547206 |
| ADCYAP1 | Adenylate Cyclase Activating Polypeptide 1 | Protein Coding | 44 | GC18P000895 | 0.444547206 |
| ARHGDIB | Rho GDP Dissociation Inhibitor Beta | Protein Coding | 44 | GC12M014942 | 0.444547206 |
| BMPER | BMP Binding Endothelial Regulator | Protein Coding | 44 | GC07P033944 | 0.444547206 |
| POLI | DNA Polymerase Iota | Protein Coding | 44 | GC18P054274 | 0.444547206 |
| SETDB1 | SET Domain Bifurcated Histone Lysine Methyltransferase 1 | Protein Coding | 44 | GC01P150926 | 0.444547206 |
| BAG1 | BAG Cochaperone 1 | Protein Coding | 43 | GC09M033245 | 0.444547206 |
| CCDC88A | Coiled-Coil Domain Containing 88A | Protein Coding | 43 | GC02M055287 | 0.444547206 |
| ESM1 | Endothelial Cell Specific Molecule 1 | Protein Coding | 43 | GC05M054977 | 0.444547206 |
| HOXB7 | Homeobox B7 | Protein Coding | 43 | GC17M048607 | 0.444547206 |
| REG4 | Regenerating Family Member 4 | Protein Coding | 43 | GC01M119794 | 0.444547206 |
| SST | Somatostatin | Protein Coding | 43 | GC03M187668 | 0.444547206 |
| KRT20 | Keratin 20 | Protein Coding | 42 | GC17M040875 | 0.444547206 |
| MCPH1 | Microcephalin 1 | Protein Coding | 42 | GC08P006406 | 0.444547206 |
| ELF5 | E74 Like ETS Transcription Factor 5 | Protein Coding | 41 | GC11M034500 | 0.444547206 |
| TNFAIP8 | TNF Alpha Induced Protein 8 | Protein Coding | 41 | GC05P119268 | 0.444547206 |
| MEG3 | Maternally Expressed 3 | RNA Gene | 28 | GC14P110419 | 0.444547206 |
| MIR27A | MicroRNA 27a | RNA Gene | 25 | GC19M014544 | 0.444547206 |
| MIR100 | MicroRNA 100 | RNA Gene | 23 | GC11M122152 | 0.444547206 |
| MIR133B | MicroRNA 133b | RNA Gene | 23 | GC06P052148 | 0.444547206 |
| MIR20A | MicroRNA 20a | RNA Gene | 23 | GC13P091567 | 0.444547206 |
| MIR181A2 | MicroRNA 181a-2 | RNA Gene | 22 | GC09P124692 | 0.444547206 |
| MIR194-2 | MicroRNA 194-2 | RNA Gene | 22 | GC11M097014 | 0.444547206 |
| MIR30A | MicroRNA 30a | RNA Gene | 22 | GC06M071403 | 0.444547206 |
| MIR183 | MicroRNA 183 | RNA Gene | 21 | GC07M129839 | 0.444547206 |
| MIR455 | MicroRNA 455 | RNA Gene | 21 | GC09P114209 | 0.444547206 |
| MIR340 | MicroRNA 340 | RNA Gene | 20 | GC05M180015 | 0.444547206 |
| MIR194-1 | MicroRNA 194-1 | RNA Gene | 18 | GC01M220118 | 0.444547206 |
| NT5E | 5'-Nucleotidase Ecto | Protein Coding | 55 | GC06P085449 | 0.43832916 |
| PCSK7 | Proprotein Convertase Subtilisin/Kexin Type 7 | Protein Coding | 47 | GC11M117199 | 0.43832916 |
| RPN2 | Ribophorin II | Protein Coding | 45 | GC20P037178 | 0.43832916 |
| XCL1 | X-C Motif Chemokine Ligand 1 | Protein Coding | 42 | GC01P168576 | 0.43832916 |
| FZD4 | Frizzled Class Receptor 4 | Protein Coding | 53 | GC11M086945 | 0.433297068 |
| APLP1 | Amyloid Beta Precursor Like Protein 1 | Protein Coding | 44 | GC19P069642 | 0.433297068 |
| NARF | Nuclear Prelamin A Recognition Factor | Protein Coding | 40 | GC17P082458 | 0.433297068 |
| FLT3 | Fms Related Receptor Tyrosine Kinase 3 | Protein Coding | 55 | GC13M028003 | 0.428383231 |
| ALPL | Alkaline Phosphatase, Biomineralization Associated | Protein Coding | 54 | GC01P021508 | 0.428383231 |
| IL2RB | Interleukin 2 Receptor Subunit Beta | Protein Coding | 52 | GC22M059411 | 0.428383231 |
| NR2F2 | Nuclear Receptor Subfamily 2 Group F Member 2 | Protein Coding | 52 | GC15P096325 | 0.428383231 |
| FASN | Fatty Acid Synthase | Protein Coding | 51 | GC17M082078 | 0.428383231 |
| C2 | Complement C2 | Protein Coding | 50 | GC06P031897 | 0.428383231 |
| CDK1 | Cyclin Dependent Kinase 1 | Protein Coding | 50 | GC10P060772 | 0.428383231 |
| CSF3R | Colony Stimulating Factor 3 Receptor | Protein Coding | 50 | GC01M036466 | 0.428383231 |
| HSPA9 | Heat Shock Protein Family A (Hsp70) Member 9 | Protein Coding | 50 | GC05M138554 | 0.428383231 |
| PIN1 | Peptidylprolyl Cis/Trans Isomerase, NIMA-Interacting 1 | Protein Coding | 50 | GC19P009835 | 0.428383231 |
| KRT1 | Keratin 1 | Protein Coding | 49 | GC12M052674 | 0.428383231 |
| LAMB2 | Laminin Subunit Beta 2 | Protein Coding | 49 | GC03M049121 | 0.428383231 |
| SLC4A4 | Solute Carrier Family 4 Member 4 | Protein Coding | 49 | GC04P071063 | 0.428383231 |
| AIMP1 | Aminoacyl TRNA Synthetase Complex Interacting Multifunctional Protein 1 | Protein Coding | 48 | GC04P106315 | 0.428383231 |
| HYOU1 | Hypoxia Up-Regulated 1 | Protein Coding | 48 | GC11M119242 | 0.428383231 |
| MEF2A | Myocyte Enhancer Factor 2A | Protein Coding | 48 | GC15P099565 | 0.428383231 |
| PPARGC1A | PPARG Coactivator 1 Alpha | Protein Coding | 48 | GC04M023755 | 0.428383231 |
| SLC40A1 | Solute Carrier Family 40 Member 1 | Protein Coding | 48 | GC02M189560 | 0.428383231 |
| KLK6 | Kallikrein Related Peptidase 6 | Protein Coding | 47 | GC19M050958 | 0.428383231 |
| PLOD1 | Procollagen-Lysine,2-Oxoglutarate 5-Dioxygenase 1 | Protein Coding | 47 | GC01P011934 | 0.428383231 |
| S1PR2 | Sphingosine-1-Phosphate Receptor 2 | Protein Coding | 47 | GC19M010223 | 0.428383231 |
| MAP2 | Microtubule Associated Protein 2 | Protein Coding | 46 | GC02P209424 | 0.428383231 |
| NDUFA13 | NADH:Ubiquinone Oxidoreductase Subunit A13 | Protein Coding | 46 | GC19P019515 | 0.428383231 |
| AKR1B10 | Aldo-Keto Reductase Family 1 Member B10 | Protein Coding | 45 | GC07P134527 | 0.428383231 |
| ARF6 | ADP Ribosylation Factor 6 | Protein Coding | 45 | GC14P049895 | 0.428383231 |
| COL9A1 | Collagen Type IX Alpha 1 Chain | Protein Coding | 45 | GC06M070215 | 0.428383231 |
| FFAR1 | Free Fatty Acid Receptor 1 | Protein Coding | 45 | GC19P070725 | 0.428383231 |
| PREP | Prolyl Endopeptidase | Protein Coding | 45 | GC06M105277 | 0.428383231 |
| TAB1 | TGF-Beta Activated Kinase 1 (MAP3K7) Binding Protein 1 | Protein Coding | 45 | GC22P041781 | 0.428383231 |
| CLDN10 | Claudin 10 | Protein Coding | 44 | GC13P095433 | 0.428383231 |
| PTPA | Protein Phosphatase 2 Phosphatase Activator | Protein Coding | 44 | GC09P129111 | 0.428383231 |
| ADGRL2 | Adhesion G Protein-Coupled Receptor L2 | Protein Coding | 43 | GC01P081306 | 0.428383231 |
| PLA2G12A | Phospholipase A2 Group XIIA | Protein Coding | 43 | GC04M109712 | 0.428383231 |
| ACKR2 | Atypical Chemokine Receptor 2 | Protein Coding | 42 | GC03P042804 | 0.428383231 |
| IL36G | Interleukin 36 Gamma | Protein Coding | 41 | GC02P112973 | 0.428383231 |
| KLF8 | KLF Transcription Factor 8 | Protein Coding | 40 | GC0XP055909 | 0.428383231 |
| JPH4 | Junctophilin 4 | Protein Coding | 37 | GC14M023568 | 0.428383231 |
| WASHC1 | WASH Complex Subunit 1 | Protein Coding | 30 | GC09M000016 | 0.428383231 |
| FENDRR | FOXF1 Adjacent Non-Coding Developmental Regulatory RNA | RNA Gene | 22 | GC16M086511 | 0.428383231 |
| MIR708 | MicroRNA 708 | RNA Gene | 20 | GC11M079402 | 0.428383231 |
| LINC00673 | Long Intergenic Non-Protein Coding RNA 673 | RNA Gene | 18 | GC17M072290 | 0.428383231 |
| MIR494 | MicroRNA 494 | RNA Gene | 18 | GC14P110455 | 0.428383231 |
| MIR4443 | MicroRNA 4443 | RNA Gene | 14 | GC03P049739 | 0.428383231 |
| LRP2BP-AS1 | LRP2BP Antisense RNA 1 | RNA Gene | 13 | GC04P185380 | 0.428383231 |
| LINC02633 | Long Intergenic Non-Protein Coding RNA 2633 | RNA Gene | 11 | GC10P043313 | 0.428383231 |
| CD99 | CD99 Molecule (Xg Blood Group) | Protein Coding | 41 | GC0XP002691 | 0.42805326 |
| DNMT3A | DNA Methyltransferase 3 Alpha | Protein Coding | 54 | GC02M025228 | 0.42007345 |
| HTR2A | 5-Hydroxytryptamine Receptor 2A | Protein Coding | 51 | GC13M046831 | 0.42007345 |
| CD79A | CD79a Molecule | Protein Coding | 50 | GC19P041877 | 0.42007345 |
| LRP6 | LDL Receptor Related Protein 6 | Protein Coding | 50 | GC12M021873 | 0.42007345 |
| PSAP | Prosaposin | Protein Coding | 50 | GC10M071816 | 0.42007345 |
| MYH2 | Myosin Heavy Chain 2 | Protein Coding | 48 | GC17M010521 | 0.42007345 |
| CD276 | CD276 Molecule | Protein Coding | 45 | GC15P073683 | 0.42007345 |
| ASPN | Asporin | Protein Coding | 44 | GC09M095327 | 0.42007345 |
| LGALS9 | Galectin 9 | Protein Coding | 43 | GC17P027629 | 0.42007345 |
| AQP1 | Aquaporin 1 (Colton Blood Group) | Protein Coding | 50 | GC07P030911 | 0.409116089 |
| NCOA1 | Nuclear Receptor Coactivator 1 | Protein Coding | 48 | GC02P024492 | 0.409116089 |
| ATF4 | Activating Transcription Factor 4 | Protein Coding | 47 | GC22P039519 | 0.409116089 |
| NR5A2 | Nuclear Receptor Subfamily 5 Group A Member 2 | Protein Coding | 47 | GC01P199996 | 0.409116089 |
| PRMT7 | Protein Arginine Methyltransferase 7 | Protein Coding | 47 | GC16P068517 | 0.409116089 |
| ST6GAL1 | ST6 Beta-Galactoside Alpha-2,6-Sialyltransferase 1 | Protein Coding | 47 | GC03P186930 | 0.409116089 |
| ACTC1 | Actin Alpha Cardiac Muscle 1 | Protein Coding | 46 | GC15M034790 | 0.409116089 |
| SPINT2 | Serine Peptidase Inhibitor, Kunitz Type 2 | Protein Coding | 45 | GC19P038244 | 0.409116089 |
| ADIPOR2 | Adiponectin Receptor 2 | Protein Coding | 44 | GC12P001670 | 0.409116089 |
| FBXO11 | F-Box Protein 11 | Protein Coding | 44 | GC02M047789 | 0.409116089 |
| ITIH2 | Inter-Alpha-Trypsin Inhibitor Heavy Chain 2 | Protein Coding | 44 | GC10P007703 | 0.409116089 |
| PNPLA3 | Patatin Like Phospholipase Domain Containing 3 | Protein Coding | 44 | GC22P043923 | 0.409116089 |
| TRPC1 | Transient Receptor Potential Cation Channel Subfamily C Member 1 | Protein Coding | 44 | GC03P142724 | 0.409116089 |
| WASL | WASP Like Actin Nucleation Promoting Factor | Protein Coding | 44 | GC07M123681 | 0.409116089 |
| BTG2 | BTG Anti-Proliferation Factor 2 | Protein Coding | 43 | GC01P203305 | 0.409116089 |
| SERPINB7 | Serpin Family B Member 7 | Protein Coding | 43 | GC18P063752 | 0.409116089 |
| SIPA1 | Signal-Induced Proliferation-Associated 1 | Protein Coding | 43 | GC11P065638 | 0.409116089 |
| COL16A1 | Collagen Type XVI Alpha 1 Chain | Protein Coding | 41 | GC01M031653 | 0.409116089 |
| RASA2 | RAS P21 Protein Activator 2 | Protein Coding | 41 | GC03P141487 | 0.409116089 |
| PEBP4 | Phosphatidylethanolamine Binding Protein 4 | Protein Coding | 40 | GC08M022713 | 0.409116089 |
| IFNL2 | Interferon Lambda 2 | Protein Coding | 37 | GC19P039268 | 0.409116089 |
| ZNF746 | Zinc Finger Protein 746 | Protein Coding | 37 | GC07M149472 | 0.409116089 |
| CLEC3A | C-Type Lectin Domain Family 3 Member A | Protein Coding | 34 | GC16P078022 | 0.409116089 |
| NBPF14 | NBPF Member 14 | Protein Coding | 26 | GC01M148531 | 0.409116089 |
| MIR106B | MicroRNA 106b | RNA Gene | 23 | GC07M102068 | 0.409116089 |
| MIR488 | MicroRNA 488 | RNA Gene | 19 | GC01M177029 | 0.409116089 |
| VTRNA2-1 | Vault RNA 2-1 | RNA Gene | 17 | GC05M136081 | 0.409116089 |
| PLIN2 | Perilipin 2 | Protein Coding | 44 | GC09M019173 | 0.403727651 |
| CDK6 | Cyclin Dependent Kinase 6 | Protein Coding | 55 | GC07M092604 | 0.401196003 |
| GRIN1 | Glutamate Ionotropic Receptor NMDA Type Subunit 1 | Protein Coding | 54 | GC09P137146 | 0.401196003 |
| TH | Tyrosine Hydroxylase | Protein Coding | 54 | GC11M002163 | 0.401196003 |
| BRD4 | Bromodomain Containing 4 | Protein Coding | 51 | GC19M015236 | 0.401196003 |
| FGA | Fibrinogen Alpha Chain | Protein Coding | 51 | GC04M154583 | 0.401196003 |
| SGK1 | Serum/Glucocorticoid Regulated Kinase 1 | Protein Coding | 51 | GC06M134169 | 0.401196003 |
| TLR8 | Toll Like Receptor 8 | Protein Coding | 51 | GC0XP012924 | 0.401196003 |
| KL | Klotho | Protein Coding | 50 | GC13P033016 | 0.401196003 |
| RAB7A | RAB7A, Member RAS Oncogene Family | Protein Coding | 50 | GC03P134584 | 0.401196003 |
| SCN9A | Sodium Voltage-Gated Channel Alpha Subunit 9 | Protein Coding | 50 | GC02M166195 | 0.401196003 |
| RAB11A | RAB11A, Member RAS Oncogene Family | Protein Coding | 49 | GC15P120465 | 0.401196003 |
| UCP2 | Uncoupling Protein 2 | Protein Coding | 49 | GC11M073974 | 0.401196003 |
| OSMR | Oncostatin M Receptor | Protein Coding | 48 | GC05P038845 | 0.401196003 |
| PML | PML Nuclear Body Scaffold | Protein Coding | 48 | GC15P073994 | 0.401196003 |
| TACSTD2 | Tumor Associated Calcium Signal Transducer 2 | Protein Coding | 48 | GC01M058575 | 0.401196003 |
| CDH13 | Cadherin 13 | Protein Coding | 47 | GC16P082626 | 0.401196003 |
| FGF14 | Fibroblast Growth Factor 14 | Protein Coding | 47 | GC13M101710 | 0.401196003 |
| IKBKE | Inhibitor Of Nuclear Factor Kappa B Kinase Subunit Epsilon | Protein Coding | 47 | GC01P206470 | 0.401196003 |
| KRT17 | Keratin 17 | Protein Coding | 47 | GC17M041619 | 0.401196003 |
| LAMA2 | Laminin Subunit Alpha 2 | Protein Coding | 47 | GC06P128863 | 0.401196003 |
| LATS1 | Large Tumor Suppressor Kinase 1 | Protein Coding | 46 | GC06M149658 | 0.401196003 |
| RHOH | Ras Homolog Family Member H | Protein Coding | 46 | GC04P040192 | 0.401196003 |
| ACVR1C | Activin A Receptor Type 1C | Protein Coding | 45 | GC02M157526 | 0.401196003 |
| HMMR | Hyaluronan Mediated Motility Receptor | Protein Coding | 45 | GC05P163480 | 0.401196003 |
| ITGA9 | Integrin Subunit Alpha 9 | Protein Coding | 45 | GC03P037468 | 0.401196003 |
| NR4A3 | Nuclear Receptor Subfamily 4 Group A Member 3 | Protein Coding | 45 | GC09P099821 | 0.401196003 |
| SGCE | Sarcoglycan Epsilon | Protein Coding | 45 | GC07M094524 | 0.401196003 |
| TSG101 | Tumor Susceptibility 101 | Protein Coding | 45 | GC11M018468 | 0.401196003 |
| KDM3A | Lysine Demethylase 3A | Protein Coding | 44 | GC02P086440 | 0.401196003 |
| METTL3 | Methyltransferase 3, N6-Adenosine-Methyltransferase Complex Catalytic Subunit | Protein Coding | 44 | GC14M021498 | 0.401196003 |
| PIGF | Phosphatidylinositol Glycan Anchor Biosynthesis Class F | Protein Coding | 44 | GC02M046580 | 0.401196003 |
| SULF1 | Sulfatase 1 | Protein Coding | 44 | GC08P069466 | 0.401196003 |
| BRD1 | Bromodomain Containing 1 | Protein Coding | 43 | GC22M049773 | 0.401196003 |
| DAB2IP | DAB2 Interacting Protein | Protein Coding | 43 | GC09P121566 | 0.401196003 |
| DMBT1 | Deleted In Malignant Brain Tumors 1 | Protein Coding | 43 | GC10P122560 | 0.401196003 |
| KRT12 | Keratin 12 | Protein Coding | 43 | GC17M040861 | 0.401196003 |
| MTA2 | Metastasis Associated 1 Family Member 2 | Protein Coding | 43 | GC11M096924 | 0.401196003 |
| PTP4A1 | Protein Tyrosine Phosphatase 4A1 | Protein Coding | 43 | GC06P088234 | 0.401196003 |
| RAP2A | RAP2A, Member Of RAS Oncogene Family | Protein Coding | 43 | GC13P097436 | 0.401196003 |
| SEMA5A | Semaphorin 5A | Protein Coding | 43 | GC05M009036 | 0.401196003 |
| WWTR1 | WW Domain Containing Transcription Regulator 1 | Protein Coding | 43 | GC03M149517 | 0.401196003 |
| ZFP36 | ZFP36 Ring Finger Protein | Protein Coding | 43 | GC19P039406 | 0.401196003 |
| GADD45B | Growth Arrest And DNA Damage Inducible Beta | Protein Coding | 42 | GC19P002476 | 0.401196003 |
| LPAR5 | Lysophosphatidic Acid Receptor 5 | Protein Coding | 42 | GC12M006618 | 0.401196003 |
| SCARA5 | Scavenger Receptor Class A Member 5 | Protein Coding | 42 | GC08M027869 | 0.401196003 |
| MAFG | MAF BZIP Transcription Factor G | Protein Coding | 41 | GC17M081918 | 0.401196003 |
| NID2 | Nidogen 2 | Protein Coding | 41 | GC14M052004 | 0.401196003 |
| SP2 | Sp2 Transcription Factor | Protein Coding | 41 | GC17P047896 | 0.401196003 |
| CPEB4 | Cytoplasmic Polyadenylation Element Binding Protein 4 | Protein Coding | 40 | GC05P173888 | 0.401196003 |
| FBLIM1 | Filamin Binding LIM Protein 1 | Protein Coding | 40 | GC01P015756 | 0.401196003 |
| SHOX | Short Stature Homeobox | Protein Coding | 40 | GC0XP000624 | 0.401196003 |
| DIXDC1 | DIX Domain Containing 1 | Protein Coding | 38 | GC11P111927 | 0.401196003 |
| NASP | Nuclear Autoantigenic Sperm Protein | Protein Coding | 38 | GC01P045583 | 0.401196003 |
| INSL4 | Insulin Like 4 | Protein Coding | 37 | GC09P005231 | 0.401196003 |
| GSDMC | Gasdermin C | Protein Coding | 36 | GC08M129705 | 0.401196003 |
| MIEN1 | Migration And Invasion Enhancer 1 | Protein Coding | 36 | GC17M039728 | 0.401196003 |
| CYRIB | CYFIP Related Rac1 Interactor B | Protein Coding | 35 | GC08M132202 | 0.401196003 |
| STATH | Statherin | Protein Coding | 33 | GC04P069995 | 0.401196003 |
| MIR132 | MicroRNA 132 | RNA Gene | 23 | GC17M002049 | 0.401196003 |
| MIR375 | MicroRNA 375 | RNA Gene | 23 | GC02M219001 | 0.401196003 |
| MIR218-2 | MicroRNA 218-2 | RNA Gene | 22 | GC05M168768 | 0.401196003 |
| MIR92A1 | MicroRNA 92a-1 | RNA Gene | 22 | GC13P091570 | 0.401196003 |
| MIR424 | MicroRNA 424 | RNA Gene | 21 | GC0XM134697 | 0.401196003 |
| MIR520A | MicroRNA 520a | RNA Gene | 20 | GC19P053690 | 0.401196003 |
| LOC106728418 | LEP 5' Regulatory Region | Biological Region | 3 | GC07P128238 | 0.401196003 |
| PKD3 | Polycystic Kidney Disease 3 (Autosomal Dominant) | Genetic Locus | 3 | GC02U990077 | 0.401196003 |
| NRAS | NRAS Proto-Oncogene, GTPase | Protein Coding | 53 | GC01M114704 | 0.399880588 |
| TBXAS1 | Thromboxane A Synthase 1 | Protein Coding | 52 | GC07P139777 | 0.399880588 |
| DUSP6 | Dual Specificity Phosphatase 6 | Protein Coding | 51 | GC12M089347 | 0.399880588 |
| ENO2 | Enolase 2 | Protein Coding | 50 | GC12P006913 | 0.399880588 |
| DLAT | Dihydrolipoamide S-Acetyltransferase | Protein Coding | 49 | GC11P112026 | 0.399880588 |
| FHL2 | Four And A Half LIM Domains 2 | Protein Coding | 49 | GC02M105357 | 0.399880588 |
| GALNT2 | Polypeptide N-Acetylgalactosaminyltransferase 2 | Protein Coding | 49 | GC01P230057 | 0.399880588 |
| LYZ | Lysozyme | Protein Coding | 49 | GC12P069348 | 0.399880588 |
| CRHR2 | Corticotropin Releasing Hormone Receptor 2 | Protein Coding | 48 | GC07M030651 | 0.399880588 |
| NR0B2 | Nuclear Receptor Subfamily 0 Group B Member 2 | Protein Coding | 48 | GC01M027137 | 0.399880588 |
| IL1RAPL1 | Interleukin 1 Receptor Accessory Protein Like 1 | Protein Coding | 47 | GC0XP028605 | 0.399880588 |
| IL3RA | Interleukin 3 Receptor Subunit Alpha | Protein Coding | 46 | GC0XP001336 | 0.399880588 |
| LYVE1 | Lymphatic Vessel Endothelial Hyaluronan Receptor 1 | Protein Coding | 46 | GC11M011096 | 0.399880588 |
| SLC39A14 | Solute Carrier Family 39 Member 14 | Protein Coding | 46 | GC08P022367 | 0.399880588 |
| LRP8 | LDL Receptor Related Protein 8 | Protein Coding | 45 | GC01M053243 | 0.399880588 |
| TNR | Tenascin R | Protein Coding | 45 | GC01M175291 | 0.399880588 |
| F13B | Coagulation Factor XIII B Chain | Protein Coding | 44 | GC01M197008 | 0.399880588 |
| BTG1 | BTG Anti-Proliferation Factor 1 | Protein Coding | 43 | GC12M092140 | 0.399880588 |
| MMRN1 | Multimerin 1 | Protein Coding | 43 | GC04P089879 | 0.399880588 |
| RPS3A | Ribosomal Protein S3A | Protein Coding | 43 | GC04P151099 | 0.399880588 |
| HSPB2 | Heat Shock Protein Family B (Small) Member 2 | Protein Coding | 41 | GC11P111975 | 0.399880588 |
| LGR4 | Leucine Rich Repeat Containing G Protein-Coupled Receptor 4 | Protein Coding | 41 | GC11M027365 | 0.399880588 |
| HMGN5 | High Mobility Group Nucleosome Binding Domain 5 | Protein Coding | 33 | GC0XM081113 | 0.399880588 |
| MIR326 | MicroRNA 326 | RNA Gene | 23 | GC11M075335 | 0.399880588 |
| MIR181B2 | MicroRNA 181b-2 | RNA Gene | 22 | GC09P124693 | 0.399880588 |
| PLCD1 | Phospholipase C Delta 1 | Protein Coding | 51 | GC03M038008 | 0.396300316 |
| SATB1 | SATB Homeobox 1 | Protein Coding | 47 | GC03M021445 | 0.396300316 |
| SPOP | Speckle Type BTB/POZ Protein | Protein Coding | 45 | GC17M049598 | 0.396300316 |
| ERLNC1 | Estrogen Receptor Responsive LncRNA 1 | RNA Gene | 14 | GC01P204141 | 0.396300316 |
| IRF3 | Interferon Regulatory Factor 3 | Protein Coding | 47 | GC19M049659 | 0.388101876 |
| MIR122 | MicroRNA 122 | RNA Gene | 24 | GC18P058451 | 0.388101876 |
| RARG | Retinoic Acid Receptor Gamma | Protein Coding | 50 | GC12M053210 | 0.376960695 |
| SPTAN1 | Spectrin Alpha, Non-Erythrocytic 1 | Protein Coding | 50 | GC09P128552 | 0.376960695 |
| YWHAH | Tyrosine 3-Monooxygenase/Tryptophan 5-Monooxygenase Activation Protein Eta | Protein Coding | 50 | GC22P031944 | 0.376960695 |
| RBPJ | Recombination Signal Binding Protein For Immunoglobulin Kappa J Region | Protein Coding | 49 | GC04P026105 | 0.376960695 |
| SYP | Synaptophysin | Protein Coding | 48 | GC0XM049187 | 0.376960695 |
| ATXN3 | Ataxin 3 | Protein Coding | 47 | GC14M101946 | 0.376960695 |
| CNR2 | Cannabinoid Receptor 2 | Protein Coding | 47 | GC01M023870 | 0.376960695 |
| GAS6 | Growth Arrest Specific 6 | Protein Coding | 47 | GC13M113820 | 0.376960695 |
| GC | GC Vitamin D Binding Protein | Protein Coding | 47 | GC04M071741 | 0.376960695 |
| MAF | MAF BZIP Transcription Factor | Protein Coding | 47 | GC16M079204 | 0.376960695 |
| SET | SET Nuclear Proto-Oncogene | Protein Coding | 47 | GC09P129028 | 0.376960695 |
| LAMP2 | Lysosomal Associated Membrane Protein 2 | Protein Coding | 46 | GC0XM120426 | 0.376960695 |
| NCK1 | NCK Adaptor Protein 1 | Protein Coding | 46 | GC03P136862 | 0.376960695 |
| AQP9 | Aquaporin 9 | Protein Coding | 45 | GC15P058138 | 0.376960695 |
| DSC3 | Desmocollin 3 | Protein Coding | 45 | GC18M030990 | 0.376960695 |
| SPRY4 | Sprouty RTK Signaling Antagonist 4 | Protein Coding | 45 | GC05M142310 | 0.376960695 |
| AKAP12 | A-Kinase Anchoring Protein 12 | Protein Coding | 44 | GC06P151239 | 0.376960695 |
| CBFA2T3 | CBFA2/RUNX1 Partner Transcriptional Co-Repressor 3 | Protein Coding | 44 | GC16M088874 | 0.376960695 |
| CD164 | CD164 Molecule | Protein Coding | 44 | GC06M109366 | 0.376960695 |
| DUSP5 | Dual Specificity Phosphatase 5 | Protein Coding | 44 | GC10P110497 | 0.376960695 |
| LGALS3BP | Galectin 3 Binding Protein | Protein Coding | 44 | GC17M078971 | 0.376960695 |
| XCR1 | X-C Motif Chemokine Receptor 1 | Protein Coding | 43 | GC03M046016 | 0.376960695 |
| CELA1 | Chymotrypsin Like Elastase 1 | Protein Coding | 42 | GC12M051328 | 0.376960695 |
| KLK10 | Kallikrein Related Peptidase 10 | Protein Coding | 42 | GC19M051012 | 0.376960695 |
| CD300LF | CD300 Molecule Like Family Member F | Protein Coding | 41 | GC17M074694 | 0.376960695 |
| IL34 | Interleukin 34 | Protein Coding | 41 | GC16P072129 | 0.376960695 |
| MAML3 | Mastermind Like Transcriptional Coactivator 3 | Protein Coding | 41 | GC04M139716 | 0.376960695 |
| TRHDE | Thyrotropin Releasing Hormone Degrading Enzyme | Protein Coding | 41 | GC12P072087 | 0.376960695 |
| CTNNAL1 | Catenin Alpha Like 1 | Protein Coding | 40 | GC09M108942 | 0.376960695 |
| CKAP4 | Cytoskeleton Associated Protein 4 | Protein Coding | 39 | GC12M106237 | 0.376960695 |
| ING2 | Inhibitor Of Growth Family Member 2 | Protein Coding | 37 | GC04P183504 | 0.376960695 |
| INTS2 | Integrator Complex Subunit 2 | Protein Coding | 37 | GC17M061865 | 0.376960695 |
| RGCC | Regulator Of Cell Cycle | Protein Coding | 37 | GC13P041457 | 0.376960695 |
| ZKSCAN3 | Zinc Finger With KRAB And SCAN Domains 3 | Protein Coding | 36 | GC06P028349 | 0.376960695 |
| BCYRN1 | Brain Cytoplasmic RNA 1 | RNA Gene | 20 | GC02P047331 | 0.376960695 |
| SPRY4-IT1 | SPRY4 Intronic Transcript 1 | RNA Gene | 16 | GC05M142318 | 0.376960695 |
| PSEN2 | Presenilin 2 | Protein Coding | 52 | GC01P226870 | 0.371313989 |
| EMD | Emerin | Protein Coding | 47 | GC0XP154379 | 0.371313989 |
| GPBAR1 | G Protein-Coupled Bile Acid Receptor 1 | Protein Coding | 45 | GC02P218259 | 0.371313989 |
| BCHE | Butyrylcholinesterase | Protein Coding | 53 | GC03M165772 | 0.365764916 |
| PLCB3 | Phospholipase C Beta 3 | Protein Coding | 52 | GC11P064251 | 0.365764916 |
| PRDX1 | Peroxiredoxin 1 | Protein Coding | 52 | GC01M045511 | 0.365764916 |
| MAPK7 | Mitogen-Activated Protein Kinase 7 | Protein Coding | 50 | GC17P057561 | 0.365764916 |
| PPP1CA | Protein Phosphatase 1 Catalytic Subunit Alpha | Protein Coding | 50 | GC11M097161 | 0.365764916 |
| SLC12A6 | Solute Carrier Family 12 Member 6 | Protein Coding | 50 | GC15M034229 | 0.365764916 |
| CUL3 | Cullin 3 | Protein Coding | 49 | GC02M224470 | 0.365764916 |
| YWHAQ | Tyrosine 3-Monooxygenase/Tryptophan 5-Monooxygenase Activation Protein Theta | Protein Coding | 49 | GC02M009583 | 0.365764916 |
| HNF1A | HNF1 Homeobox A | Protein Coding | 48 | GC12P120978 | 0.365764916 |
| FUS | FUS RNA Binding Protein | Protein Coding | 47 | GC16P031180 | 0.365764916 |
| GRK2 | G Protein-Coupled Receptor Kinase 2 | Protein Coding | 47 | GC11P067266 | 0.365764916 |
| ORAI1 | ORAI Calcium Release-Activated Calcium Modulator 1 | Protein Coding | 47 | GC12P126604 | 0.365764916 |
| CTBP2 | C-Terminal Binding Protein 2 | Protein Coding | 46 | GC10M124984 | 0.365764916 |
| EIF2S1 | Eukaryotic Translation Initiation Factor 2 Subunit Alpha | Protein Coding | 46 | GC14P067359 | 0.365764916 |
| P2RY11 | Purinergic Receptor P2Y11 | Protein Coding | 46 | GC19P010618 | 0.365764916 |
| CUL4A | Cullin 4A | Protein Coding | 45 | GC13P113208 | 0.365764916 |
| PLAA | Phospholipase A2 Activating Protein | Protein Coding | 45 | GC09M026903 | 0.365764916 |
| RCAN1 | Regulator Of Calcineurin 1 | Protein Coding | 45 | GC21M034513 | 0.365764916 |
| ANXA4 | Annexin A4 | Protein Coding | 44 | GC02P069644 | 0.365764916 |
| DNAJB6 | DnaJ Heat Shock Protein Family (Hsp40) Member B6 | Protein Coding | 44 | GC07P157335 | 0.365764916 |
| HIPK1 | Homeodomain Interacting Protein Kinase 1 | Protein Coding | 44 | GC01P113929 | 0.365764916 |
| HOXD10 | Homeobox D10 | Protein Coding | 44 | GC02P176108 | 0.365764916 |
| PGLYRP1 | Peptidoglycan Recognition Protein 1 | Protein Coding | 44 | GC19M068032 | 0.365764916 |
| RHCG | Rh Family C Glycoprotein | Protein Coding | 44 | GC15M089471 | 0.365764916 |
| TERF2IP | TERF2 Interacting Protein | Protein Coding | 44 | GC16P075647 | 0.365764916 |
| TXNDC5 | Thioredoxin Domain Containing 5 | Protein Coding | 44 | GC06M007893 | 0.365764916 |
| HIC1 | HIC ZBTB Transcriptional Repressor 1 | Protein Coding | 43 | GC17P002054 | 0.365764916 |
| SMARCA5 | SWI/SNF Related, Matrix Associated, Actin Dependent Regulator Of Chromatin, Subfamily A, Member 5 | Protein Coding | 43 | GC04P143513 | 0.365764916 |
| TSPAN1 | Tetraspanin 1 | Protein Coding | 43 | GC01P046175 | 0.365764916 |
| CORO1C | Coronin 1C | Protein Coding | 42 | GC12M108645 | 0.365764916 |
| PIGS | Phosphatidylinositol Glycan Anchor Biosynthesis Class S | Protein Coding | 42 | GC17M028553 | 0.365764916 |
| BCO1 | Beta-Carotene Oxygenase 1 | Protein Coding | 41 | GC16P081238 | 0.365764916 |
| CRIM1 | Cysteine Rich Transmembrane BMP Regulator 1 | Protein Coding | 41 | GC02P036355 | 0.365764916 |
| LZTFL1 | Leucine Zipper Transcription Factor Like 1 | Protein Coding | 41 | GC03M045823 | 0.365764916 |
| MYBBP1A | MYB Binding Protein 1a | Protein Coding | 41 | GC17M004538 | 0.365764916 |
| CORT | Cortistatin | Protein Coding | 40 | GC01P010519 | 0.365764916 |
| IL36A | Interleukin 36 Alpha | Protein Coding | 40 | GC02P113005 | 0.365764916 |
| SCGN | Secretagogin, EF-Hand Calcium Binding Protein | Protein Coding | 40 | GC06P025652 | 0.365764916 |
| PIWIL2 | Piwi Like RNA-Mediated Gene Silencing 2 | Protein Coding | 39 | GC08P022275 | 0.365764916 |
| RETNLB | Resistin Like Beta | Protein Coding | 38 | GC03M108743 | 0.365764916 |
| SEPTIN2 | Septin 2 | Protein Coding | 38 | GC02P241316 | 0.365764916 |
| SKA3 | Spindle And Kinetochore Associated Complex Subunit 3 | Protein Coding | 38 | GC13M021153 | 0.365764916 |
| DERL3 | Derlin 3 | Protein Coding | 37 | GC22M023834 | 0.365764916 |
| FILIP1L | Filamin A Interacting Protein 1 Like | Protein Coding | 37 | GC03M099830 | 0.365764916 |
| TOR1AIP2 | Torsin 1A Interacting Protein 2 | Protein Coding | 37 | GC01M183807 | 0.365764916 |
| SLFN5 | Schlafen Family Member 5 | Protein Coding | 33 | GC17P035243 | 0.365764916 |
| MIR130A | MicroRNA 130a | RNA Gene | 23 | GC11P057641 | 0.365764916 |
| MIR196A1 | MicroRNA 196a-1 | RNA Gene | 23 | GC17M048632 | 0.365764916 |
| MIR1-1 | MicroRNA 1-1 | RNA Gene | 22 | GC20P063403 | 0.365764916 |
| MIR31 | MicroRNA 31 | RNA Gene | 22 | GC09M021793 | 0.365764916 |
| MIR324 | MicroRNA 324 | RNA Gene | 22 | GC17M007223 | 0.365764916 |
| MIR381 | MicroRNA 381 | RNA Gene | 20 | GC14P110443 | 0.365764916 |
| MIR409 | MicroRNA 409 | RNA Gene | 20 | GC14P110446 | 0.365764916 |
| LINC00511 | Long Intergenic Non-Protein Coding RNA 511 | RNA Gene | 19 | GC17M072323 | 0.365764916 |
| MIR124-3 | MicroRNA 124-3 | RNA Gene | 19 | GC20P063429 | 0.365764916 |
| PTCSC3 | Papillary Thyroid Carcinoma Susceptibility Candidate 3 | RNA Gene | 18 | GC14M036184 | 0.365764916 |
| LINC01128 | Long Intergenic Non-Protein Coding RNA 1128 | RNA Gene | 17 | GC01P004103 | 0.365764916 |
| BANCR | BRAF-Activated Non-Protein Coding RNA | RNA Gene | 16 | GC09M069296 | 0.365764916 |
| MIR665 | MicroRNA 665 | RNA Gene | 16 | GC14P110464 | 0.365764916 |
| MIR558 | MicroRNA 558 | RNA Gene | 15 | GC02P032572 | 0.365764916 |
| MIR888 | MicroRNA 888 | RNA Gene | 13 | GC0XM145996 | 0.365764916 |
| HYAL1 | Hyaluronidase 1 | Protein Coding | 50 | GC03M050299 | 0.361771047 |
| PAX6 | Paired Box 6 | Protein Coding | 50 | GC11M031784 | 0.361771047 |
| PPP1R12A | Protein Phosphatase 1 Regulatory Subunit 12A | Protein Coding | 46 | GC12M079773 | 0.361771047 |
| ARHGAP5 | Rho GTPase Activating Protein 5 | Protein Coding | 44 | GC14P033720 | 0.361771047 |
| DDX17 | DEAD-Box Helicase 17 | Protein Coding | 43 | GC22M038483 | 0.361771047 |
| PELP1 | Proline, Glutamate And Leucine Rich Protein 1 | Protein Coding | 42 | GC17M004669 | 0.361771047 |
| SPDEF | SAM Pointed Domain Containing ETS Transcription Factor | Protein Coding | 41 | GC06M069336 | 0.361771047 |
| TUFT1 | Tuftelin 1 | Protein Coding | 41 | GC01P151513 | 0.361771047 |
| DANCR | Differentiation Antagonizing Non-Protein Coding RNA | RNA Gene | 25 | GC04P052712 | 0.361771047 |
| ZEB1-AS1 | ZEB1 Antisense RNA 1 | RNA Gene | 19 | GC10M031166 | 0.361771047 |
| CFL1 | Cofilin 1 | Protein Coding | 48 | GC11M065823 | 0.353058279 |
| NR3C2 | Nuclear Receptor Subfamily 3 Group C Member 2 | Protein Coding | 48 | GC04M148078 | 0.353058279 |
| SLC39A8 | Solute Carrier Family 39 Member 8 | Protein Coding | 47 | GC04M102252 | 0.353058279 |
| DSG1 | Desmoglein 1 | Protein Coding | 45 | GC18P031318 | 0.353058279 |
| RHBDF2 | Rhomboid 5 Homolog 2 | Protein Coding | 45 | GC17M076470 | 0.353058279 |
| HGFAC | HGF Activator | Protein Coding | 44 | GC04P003443 | 0.353058279 |
| PKP1 | Plakophilin 1 | Protein Coding | 44 | GC01P201283 | 0.353058279 |
| SVIL | Supervillin | Protein Coding | 44 | GC10M033306 | 0.353058279 |
| PLXNC1 | Plexin C1 | Protein Coding | 43 | GC12P094150 | 0.353058279 |
| SEMA3B | Semaphorin 3B | Protein Coding | 43 | GC03P050267 | 0.353058279 |
| TXNIP | Thioredoxin Interacting Protein | Protein Coding | 41 | GC01M145992 | 0.353058279 |
| COL5A3 | Collagen Type V Alpha 3 Chain | Protein Coding | 40 | GC19M009931 | 0.353058279 |
| NOTCH2 | Notch Receptor 2 | Protein Coding | 54 | GC01M119911 | 0.349773467 |
| NPM1 | Nucleophosmin 1 | Protein Coding | 53 | GC05P171387 | 0.349773467 |
| LRP5 | LDL Receptor Related Protein 5 | Protein Coding | 52 | GC11P068298 | 0.349773467 |
| ACTA1 | Actin Alpha 1, Skeletal Muscle | Protein Coding | 51 | GC01M229584 | 0.349773467 |
| GLI2 | GLI Family Zinc Finger 2 | Protein Coding | 51 | GC02P120735 | 0.349773467 |
| SLC5A1 | Solute Carrier Family 5 Member 1 | Protein Coding | 51 | GC22P032043 | 0.349773467 |
| ABCC6 | ATP Binding Cassette Subfamily C Member 6 | Protein Coding | 50 | GC16M016148 | 0.349773467 |
| FAAH | Fatty Acid Amide Hydrolase | Protein Coding | 50 | GC01P046394 | 0.349773467 |
| HDAC8 | Histone Deacetylase 8 | Protein Coding | 50 | GC0XM072329 | 0.349773467 |
| NR1D1 | Nuclear Receptor Subfamily 1 Group D Member 1 | Protein Coding | 50 | GC17M040092 | 0.349773467 |
| SLC2A3 | Solute Carrier Family 2 Member 3 | Protein Coding | 50 | GC12M007919 | 0.349773467 |
| ACTN4 | Actinin Alpha 4 | Protein Coding | 49 | GC19P038647 | 0.349773467 |
| MAP3K8 | Mitogen-Activated Protein Kinase Kinase Kinase 8 | Protein Coding | 49 | GC10P030520 | 0.349773467 |
| DDX5 | DEAD-Box Helicase 5 | Protein Coding | 48 | GC17M064498 | 0.349773467 |
| GNAI1 | G Protein Subunit Alpha I1 | Protein Coding | 48 | GC07P079769 | 0.349773467 |
| MST1 | Macrophage Stimulating 1 | Protein Coding | 48 | GC03M049683 | 0.349773467 |
| TGM1 | Transglutaminase 1 | Protein Coding | 48 | GC14M024249 | 0.349773467 |
| ALAS2 | 5'-Aminolevulinate Synthase 2 | Protein Coding | 47 | GC0XM055009 | 0.349773467 |
| ANTXR1 | ANTXR Cell Adhesion Molecule 1 | Protein Coding | 47 | GC02P068977 | 0.349773467 |
| APOH | Apolipoprotein H | Protein Coding | 47 | GC17M066212 | 0.349773467 |
| DOCK1 | Dedicator Of Cytokinesis 1 | Protein Coding | 47 | GC10P126905 | 0.349773467 |
| HLA-C | Major Histocompatibility Complex, Class I, C | Protein Coding | 47 | GC06M069165 | 0.349773467 |
| IL11RA | Interleukin 11 Receptor Subunit Alpha | Protein Coding | 47 | GC09P048030 | 0.349773467 |
| INVS | Inversin | Protein Coding | 47 | GC09P100099 | 0.349773467 |
| ITM2B | Integral Membrane Protein 2B | Protein Coding | 47 | GC13P048233 | 0.349773467 |
| KIF1B | Kinesin Family Member 1B | Protein Coding | 47 | GC01P010210 | 0.349773467 |
| NR1I3 | Nuclear Receptor Subfamily 1 Group I Member 3 | Protein Coding | 47 | GC01M161229 | 0.349773467 |
| PON3 | Paraoxonase 3 | Protein Coding | 47 | GC07M095359 | 0.349773467 |
| PRPS2 | Phosphoribosyl Pyrophosphate Synthetase 2 | Protein Coding | 47 | GC0XP012791 | 0.349773467 |
| RACGAP1 | Rac GTPase Activating Protein 1 | Protein Coding | 47 | GC12M049978 | 0.349773467 |
| TRAF3IP2 | TRAF3 Interacting Protein 2 | Protein Coding | 47 | GC06M111555 | 0.349773467 |
| C7 | Complement C7 | Protein Coding | 46 | GC05P040909 | 0.349773467 |
| FHIT | Fragile Histidine Triad Diadenosine Triphosphatase | Protein Coding | 46 | GC03M059747 | 0.349773467 |
| HGS | Hepatocyte Growth Factor-Regulated Tyrosine Kinase Substrate | Protein Coding | 46 | GC17P081683 | 0.349773467 |
| MYL9 | Myosin Light Chain 9 | Protein Coding | 46 | GC20P036541 | 0.349773467 |
| SPI1 | Spi-1 Proto-Oncogene | Protein Coding | 46 | GC11M096710 | 0.349773467 |
| TFAM | Transcription Factor A, Mitochondrial | Protein Coding | 46 | GC10P058385 | 0.349773467 |
| TNFRSF9 | TNF Receptor Superfamily Member 9 | Protein Coding | 46 | GC01M007915 | 0.349773467 |
| ARHGEF7 | Rho Guanine Nucleotide Exchange Factor 7 | Protein Coding | 45 | GC13P111114 | 0.349773467 |
| CYP2J2 | Cytochrome P450 Family 2 Subfamily J Member 2 | Protein Coding | 45 | GC01M059893 | 0.349773467 |
| FOXP2 | Forkhead Box P2 | Protein Coding | 45 | GC07P114086 | 0.349773467 |
| IL1RL2 | Interleukin 1 Receptor Like 2 | Protein Coding | 45 | GC02P102186 | 0.349773467 |
| NDUFAF4 | NADH:Ubiquinone Oxidoreductase Complex Assembly Factor 4 | Protein Coding | 45 | GC06M096889 | 0.349773467 |
| NLRP1 | NLR Family Pyrin Domain Containing 1 | Protein Coding | 45 | GC17M005499 | 0.349773467 |
| NMBR | Neuromedin B Receptor | Protein Coding | 45 | GC06M142059 | 0.349773467 |
| P4HA1 | Prolyl 4-Hydroxylase Subunit Alpha 1 | Protein Coding | 45 | GC10M073007 | 0.349773467 |
| RBP3 | Retinol Binding Protein 3 | Protein Coding | 45 | GC10P047348 | 0.349773467 |
| SATB2 | SATB Homeobox 2 | Protein Coding | 45 | GC02M199269 | 0.349773467 |
| ANO6 | Anoctamin 6 | Protein Coding | 44 | GC12P045215 | 0.349773467 |
| CYB561 | Cytochrome B561 | Protein Coding | 44 | GC17M063432 | 0.349773467 |
| FRZB | Frizzled Related Protein | Protein Coding | 44 | GC02M182833 | 0.349773467 |
| KRT4 | Keratin 4 | Protein Coding | 44 | GC12M052806 | 0.349773467 |
| NPTN | Neuroplastin | Protein Coding | 44 | GC15M073560 | 0.349773467 |
| PTMA | Prothymosin Alpha | Protein Coding | 44 | GC02P231728 | 0.349773467 |
| RREB1 | Ras Responsive Element Binding Protein 1 | Protein Coding | 44 | GC06P007107 | 0.349773467 |
| RUNX1T1 | RUNX1 Partner Transcriptional Co-Repressor 1 | Protein Coding | 44 | GC08M091954 | 0.349773467 |
| SLC52A3 | Solute Carrier Family 52 Member 3 | Protein Coding | 44 | GC20M000741 | 0.349773467 |
| SPTB | Spectrin Beta, Erythrocytic | Protein Coding | 44 | GC14M064746 | 0.349773467 |
| TOPBP1 | DNA Topoisomerase II Binding Protein 1 | Protein Coding | 44 | GC03M133600 | 0.349773467 |
| CAVIN1 | Caveolae Associated Protein 1 | Protein Coding | 43 | GC17M049471 | 0.349773467 |
| COL8A2 | Collagen Type VIII Alpha 2 Chain | Protein Coding | 43 | GC01M036095 | 0.349773467 |
| CRYBA4 | Crystallin Beta A4 | Protein Coding | 43 | GC22P040369 | 0.349773467 |
| DUSP16 | Dual Specificity Phosphatase 16 | Protein Coding | 43 | GC12M012473 | 0.349773467 |
| ELMO1 | Engulfment And Cell Motility 1 | Protein Coding | 43 | GC07M036860 | 0.349773467 |
| HDGF | Heparin Binding Growth Factor | Protein Coding | 43 | GC01M156925 | 0.349773467 |
| PIEZO1 | Piezo Type Mechanosensitive Ion Channel Component 1 | Protein Coding | 43 | GC16M088715 | 0.349773467 |
| RAB14 | RAB14, Member RAS Oncogene Family | Protein Coding | 43 | GC09M121178 | 0.349773467 |
| TARBP2 | TARBP2 Subunit Of RISC Loading Complex | Protein Coding | 43 | GC12P053499 | 0.349773467 |
| ELOC | Elongin C | Protein Coding | 42 | GC08M073939 | 0.349773467 |
| INSL3 | Insulin Like 3 | Protein Coding | 42 | GC19M017816 | 0.349773467 |
| SLC39A6 | Solute Carrier Family 39 Member 6 | Protein Coding | 42 | GC18M036108 | 0.349773467 |
| ACKR1 | Atypical Chemokine Receptor 1 (Duffy Blood Group) | Protein Coding | 41 | GC01P159203 | 0.349773467 |
| COL15A1 | Collagen Type XV Alpha 1 Chain | Protein Coding | 41 | GC09P098912 | 0.349773467 |
| DPP8 | Dipeptidyl Peptidase 8 | Protein Coding | 41 | GC15M065442 | 0.349773467 |
| GLCE | Glucuronic Acid Epimerase | Protein Coding | 41 | GC15P120475 | 0.349773467 |
| HAS1 | Hyaluronan Synthase 1 | Protein Coding | 41 | GC19M068314 | 0.349773467 |
| NANOS1 | Nanos C2HC-Type Zinc Finger 1 | Protein Coding | 41 | GC10P119029 | 0.349773467 |
| RSU1 | Ras Suppressor Protein 1 | Protein Coding | 41 | GC10M016672 | 0.349773467 |
| SPON1 | Spondin 1 | Protein Coding | 41 | GC11P013940 | 0.349773467 |
| SULF2 | Sulfatase 2 | Protein Coding | 41 | GC20M047656 | 0.349773467 |
| COL21A1 | Collagen Type XXI Alpha 1 Chain | Protein Coding | 40 | GC06M069648 | 0.349773467 |
| CYGB | Cytoglobin | Protein Coding | 40 | GC17M076527 | 0.349773467 |
| ELOB | Elongin B | Protein Coding | 40 | GC16M007619 | 0.349773467 |
| MPP2 | MAGUK P55 Scaffold Protein 2 | Protein Coding | 40 | GC17M043875 | 0.349773467 |
| SPSB1 | SplA/Ryanodine Receptor Domain And SOCS Box Containing 1 | Protein Coding | 40 | GC01P009292 | 0.349773467 |
| CBFA2T2 | CBFA2/RUNX1 Partner Transcriptional Co-Repressor 2 | Protein Coding | 39 | GC20P033490 | 0.349773467 |
| FETUB | Fetuin B | Protein Coding | 39 | GC03P186635 | 0.349773467 |
| IL36B | Interleukin 36 Beta | Protein Coding | 39 | GC02M113022 | 0.349773467 |
| MPIG6B | Megakaryocyte And Platelet Inhibitory Receptor G6b | Protein Coding | 39 | GC06P090239 | 0.349773467 |
| RAP2B | RAP2B, Member Of RAS Oncogene Family | Protein Coding | 39 | GC03P153162 | 0.349773467 |
| TFAP4 | Transcription Factor AP-4 | Protein Coding | 39 | GC16M007723 | 0.349773467 |
| USP39 | Ubiquitin Specific Peptidase 39 | Protein Coding | 39 | GC02P085866 | 0.349773467 |
| EXOC1 | Exocyst Complex Component 1 | Protein Coding | 38 | GC04P055853 | 0.349773467 |
| KIAA0586 | KIAA0586 | Protein Coding | 38 | GC14P058427 | 0.349773467 |
| NRDC | Nardilysin Convertase | Protein Coding | 38 | GC01M051833 | 0.349773467 |
| ZBTB38 | Zinc Finger And BTB Domain Containing 38 | Protein Coding | 38 | GC03P141324 | 0.349773467 |
| NCR3LG1 | Natural Killer Cell Cytotoxicity Receptor 3 Ligand 1 | Protein Coding | 37 | GC11P017351 | 0.349773467 |
| ZBTB4 | Zinc Finger And BTB Domain Containing 4 | Protein Coding | 37 | GC17M007459 | 0.349773467 |
| PRR11 | Proline Rich 11 | Protein Coding | 36 | GC17P059155 | 0.349773467 |
| SPSB4 | SplA/Ryanodine Receptor Domain And SOCS Box Containing 4 | Protein Coding | 35 | GC03P141051 | 0.349773467 |
| TMEM88 | Transmembrane Protein 88 | Protein Coding | 33 | GC17P011469 | 0.349773467 |
| ZNF580 | Zinc Finger Protein 580 | Protein Coding | 33 | GC19P070499 | 0.349773467 |
| ANXA2R | Annexin A2 Receptor | Protein Coding | 31 | GC05M044078 | 0.349773467 |
| DLEU1 | Deleted In Lymphocytic Leukemia 1 | RNA Gene | 31 | GC13P050127 | 0.349773467 |
| XIST | X Inactive Specific Transcript | RNA Gene | 25 | GC0XM073820 | 0.349773467 |
| MIR10A | MicroRNA 10a | RNA Gene | 23 | GC17M048579 | 0.349773467 |
| MIR24-2 | MicroRNA 24-2 | RNA Gene | 23 | GC19M014543 | 0.349773467 |
| MIR449A | MicroRNA 449a | RNA Gene | 23 | GC05M055171 | 0.349773467 |
| TRB | T Cell Receptor Beta Locus | Protein Coding | 23 | GC07P149825 | 0.349773467 |
| MIR193A | MicroRNA 193a | RNA Gene | 22 | GC17P031559 | 0.349773467 |
| MIR134 | MicroRNA 134 | RNA Gene | 21 | GC14P110425 | 0.349773467 |
| MIR144 | MicroRNA 144 | RNA Gene | 21 | GC17M044993 | 0.349773467 |
| MIR296 | MicroRNA 296 | RNA Gene | 20 | GC20M058817 | 0.349773467 |
| MIR584 | MicroRNA 584 | RNA Gene | 20 | GC05M149062 | 0.349773467 |
| MIR361 | MicroRNA 361 | RNA Gene | 19 | GC0XM085903 | 0.349773467 |
| FOXC2-AS1 | FOXC2 Antisense RNA 1 | RNA Gene | 16 | GC16M086566 | 0.349773467 |
| LINC02663 | Long Intergenic Non-Protein Coding RNA 2663 | RNA Gene | 9 | GC10M009444 | 0.349773467 |
| RPL28P4 | Ribosomal Protein L28 Pseudogene 4 | Pseudogene | 5 | GC15P058200 | 0.349773467 |
| LOC111099027 | Transmembrane Protease Serine 2 Breakpoint Cluster Recombination Region | Biological Region | 3 | GC21P041485 | 0.349773467 |
| LOC111099028 | ERG, ETS Transcription Factor Breakpoint Cluster Recombination Region | Biological Region | 3 | GC21P038454 | 0.349773467 |
| PDGFRA | Platelet Derived Growth Factor Receptor Alpha | Protein Coding | 56 | GC04P054229 | 0.332865417 |
| HSD11B1 | Hydroxysteroid 11-Beta Dehydrogenase 1 | Protein Coding | 54 | GC01P209686 | 0.332865417 |
| CSNK2A1 | Casein Kinase 2 Alpha 1 | Protein Coding | 53 | GC20M000472 | 0.332865417 |
| MYH9 | Myosin Heavy Chain 9 | Protein Coding | 51 | GC22M036281 | 0.332865417 |
| CCKBR | Cholecystokinin B Receptor | Protein Coding | 50 | GC11P006259 | 0.332865417 |
| GHSR | Growth Hormone Secretagogue Receptor | Protein Coding | 50 | GC03M172443 | 0.332865417 |
| P2RY2 | Purinergic Receptor P2Y2 | Protein Coding | 50 | GC11P073202 | 0.332865417 |
| NOTCH4 | Notch Receptor 4 | Protein Coding | 49 | GC06M069238 | 0.332865417 |
| AOC3 | Amine Oxidase Copper Containing 3 | Protein Coding | 48 | GC17P042851 | 0.332865417 |
| SERPINI1 | Serpin Family I Member 1 | Protein Coding | 48 | GC03P167735 | 0.332865417 |
| STS | Steroid Sulfatase | Protein Coding | 48 | GC0XP007146 | 0.332865417 |
| DLK1 | Delta Like Non-Canonical Notch Ligand 1 | Protein Coding | 47 | GC14P110229 | 0.332865417 |
| IRS2 | Insulin Receptor Substrate 2 | Protein Coding | 47 | GC13M109752 | 0.332865417 |
| SFRP4 | Secreted Frizzled Related Protein 4 | Protein Coding | 47 | GC07M037912 | 0.332865417 |
| DPEP1 | Dipeptidase 1 | Protein Coding | 46 | GC16P089613 | 0.332865417 |
| PRELP | Proline And Arginine Rich End Leucine Rich Repeat Protein | Protein Coding | 45 | GC01P203475 | 0.332865417 |
| CILP | Cartilage Intermediate Layer Protein | Protein Coding | 44 | GC15M065194 | 0.332865417 |
| AP2A1 | Adaptor Related Protein Complex 2 Subunit Alpha 1 | Protein Coding | 43 | GC19P070214 | 0.332865417 |
| CABIN1 | Calcineurin Binding Protein 1 | Protein Coding | 43 | GC22P024011 | 0.332865417 |
| S100A2 | S100 Calcium Binding Protein A2 | Protein Coding | 43 | GC01M153561 | 0.332865417 |
| OPN3 | Opsin 3 | Protein Coding | 41 | GC01M241590 | 0.332865417 |
| MIR137 | MicroRNA 137 | RNA Gene | 22 | GC01M098046 | 0.332865417 |
| B4GALT1 | Beta-1,4-Galactosyltransferase 1 | Protein Coding | 50 | GC09M033100 | 0.323577851 |
| C1S | Complement C1s | Protein Coding | 50 | GC12P022388 | 0.323577851 |
| CYP27A1 | Cytochrome P450 Family 27 Subfamily A Member 1 | Protein Coding | 49 | GC02P218781 | 0.323577851 |
| ABCA4 | ATP Binding Cassette Subfamily A Member 4 | Protein Coding | 48 | GC01M093992 | 0.323577851 |
| SREBF1 | Sterol Regulatory Element Binding Transcription Factor 1 | Protein Coding | 48 | GC17M017810 | 0.323577851 |
| ATP6V1B1 | ATPase H+ Transporting V1 Subunit B1 | Protein Coding | 47 | GC02P070935 | 0.323577851 |
| MAP2K5 | Mitogen-Activated Protein Kinase Kinase 5 | Protein Coding | 47 | GC15P120470 | 0.323577851 |
| HYAL2 | Hyaluronidase 2 | Protein Coding | 46 | GC03M050317 | 0.323577851 |
| ATG7 | Autophagy Related 7 | Protein Coding | 45 | GC03P012714 | 0.323577851 |
| NR2C2 | Nuclear Receptor Subfamily 2 Group C Member 2 | Protein Coding | 45 | GC03P014947 | 0.323577851 |
| RAB2A | RAB2A, Member RAS Oncogene Family | Protein Coding | 44 | GC08P060516 | 0.323577851 |
| SMPD2 | Sphingomyelin Phosphodiesterase 2 | Protein Coding | 44 | GC06P109440 | 0.323577851 |
| DLX1 | Distal-Less Homeobox 1 | Protein Coding | 41 | GC02P172084 | 0.323577851 |
| DLX2 | Distal-Less Homeobox 2 | Protein Coding | 41 | GC02M172099 | 0.323577851 |
| CCDC134 | Coiled-Coil Domain Containing 134 | Protein Coding | 40 | GC22P041800 | 0.323577851 |
| TM4SF1 | Transmembrane 4 L Six Family Member 1 | Protein Coding | 39 | GC03M149370 | 0.323577851 |
| BRINP1 | BMP/Retinoic Acid Inducible Neural Specific 1 | Protein Coding | 37 | GC09M119153 | 0.323577851 |
| ZNF267 | Zinc Finger Protein 267 | Protein Coding | 37 | GC16P042008 | 0.323577851 |
| SOHLH2 | Spermatogenesis And Oogenesis Specific Basic Helix-Loop-Helix 2 | Protein Coding | 35 | GC13M037017 | 0.323577851 |
| TNFAIP8L1 | TNF Alpha Induced Protein 8 Like 1 | Protein Coding | 35 | GC19P004639 | 0.323577851 |
| AJAP1 | Adherens Junctions Associated Protein 1 | Protein Coding | 34 | GC01P004654 | 0.323577851 |
| HEPN1 | Hepatocellular Carcinoma, Down-Regulated 1 | Protein Coding | 29 | GC11P124919 | 0.323577851 |
| TP73-AS1 | TP73 Antisense RNA 1 | RNA Gene | 24 | GC01M007075 | 0.323577851 |
| AFAP1-AS1 | AFAP1 Antisense RNA 1 | RNA Gene | 20 | GC04P007756 | 0.323577851 |
| NNT-AS1 | NNT Antisense RNA 1 | RNA Gene | 17 | GC05M044138 | 0.323577851 |
| CDK5 | Cyclin Dependent Kinase 5 | Protein Coding | 55 | GC07M151053 | 0.31434235 |
| ACVRL1 | Activin A Receptor Like Type 1 | Protein Coding | 53 | GC12P051906 | 0.31434235 |
| ACTN1 | Actinin Alpha 1 | Protein Coding | 52 | GC14M068874 | 0.31434235 |
| BMPR1B | Bone Morphogenetic Protein Receptor Type 1B | Protein Coding | 52 | GC04P094757 | 0.31434235 |
| BUB1B | BUB1 Mitotic Checkpoint Serine/Threonine Kinase B | Protein Coding | 52 | GC15P040161 | 0.31434235 |
| GLA | Galactosidase Alpha | Protein Coding | 52 | GC0XM101393 | 0.31434235 |
| MSH2 | MutS Homolog 2 | Protein Coding | 52 | GC02P047403 | 0.31434235 |
| TRPC6 | Transient Receptor Potential Cation Channel Subfamily C Member 6 | Protein Coding | 52 | GC11M101451 | 0.31434235 |
| YWHAE | Tyrosine 3-Monooxygenase/Tryptophan 5-Monooxygenase Activation Protein Epsilon | Protein Coding | 52 | GC17M004893 | 0.31434235 |
| ADCY1 | Adenylate Cyclase 1 | Protein Coding | 51 | GC07P045580 | 0.31434235 |
| GABRG2 | Gamma-Aminobutyric Acid Type A Receptor Subunit Gamma2 | Protein Coding | 51 | GC05P162000 | 0.31434235 |
| KAT5 | Lysine Acetyltransferase 5 | Protein Coding | 51 | GC11P065711 | 0.31434235 |
| P2RY12 | Purinergic Receptor P2Y12 | Protein Coding | 51 | GC03M151336 | 0.31434235 |
| USP9X | Ubiquitin Specific Peptidase 9 X-Linked | Protein Coding | 51 | GC0XP041085 | 0.31434235 |
| CHRNA3 | Cholinergic Receptor Nicotinic Alpha 3 Subunit | Protein Coding | 50 | GC15M118958 | 0.31434235 |
| CSNK1E | Casein Kinase 1 Epsilon | Protein Coding | 50 | GC22M059593 | 0.31434235 |
| DNM1L | Dynamin 1 Like | Protein Coding | 50 | GC12P032679 | 0.31434235 |
| FLNB | Filamin B | Protein Coding | 50 | GC03P058008 | 0.31434235 |
| NF2 | NF2, Moesin-Ezrin-Radixin Like (MERLIN) Tumor Suppressor | Protein Coding | 50 | GC22P029603 | 0.31434235 |
| OAT | Ornithine Aminotransferase | Protein Coding | 50 | GC10M124397 | 0.31434235 |
| SIRT3 | Sirtuin 3 | Protein Coding | 50 | GC11M000215 | 0.31434235 |
| TPM3 | Tropomyosin 3 | Protein Coding | 50 | GC01M154127 | 0.31434235 |
| TRPV6 | Transient Receptor Potential Cation Channel Subfamily V Member 6 | Protein Coding | 50 | GC07M142871 | 0.31434235 |
| IHH | Indian Hedgehog Signaling Molecule | Protein Coding | 49 | GC02M219054 | 0.31434235 |
| NOG | Noggin | Protein Coding | 49 | GC17P056593 | 0.31434235 |
| ST3GAL3 | ST3 Beta-Galactoside Alpha-2,3-Sialyltransferase 3 | Protein Coding | 49 | GC01P043705 | 0.31434235 |
| WNT4 | Wnt Family Member 4 | Protein Coding | 49 | GC01M022190 | 0.31434235 |
| C1QC | Complement C1q C Chain | Protein Coding | 48 | GC01P022643 | 0.31434235 |
| EPS8 | Epidermal Growth Factor Receptor Pathway Substrate 8 | Protein Coding | 48 | GC12M021929 | 0.31434235 |
| GFER | Growth Factor, Augmenter Of Liver Regeneration | Protein Coding | 48 | GC16P001984 | 0.31434235 |
| HIPK2 | Homeodomain Interacting Protein Kinase 2 | Protein Coding | 48 | GC07M139561 | 0.31434235 |
| HSPA1A | Heat Shock Protein Family A (Hsp70) Member 1A | Protein Coding | 48 | GC06P087759 | 0.31434235 |
| IL7R | Interleukin 7 Receptor | Protein Coding | 48 | GC05P035852 | 0.31434235 |
| LAMA4 | Laminin Subunit Alpha 4 | Protein Coding | 48 | GC06M112107 | 0.31434235 |
| MPZ | Myelin Protein Zero | Protein Coding | 48 | GC01M161304 | 0.31434235 |
| PHB1 | Prohibitin 1 | Protein Coding | 48 | GC17M049452 | 0.31434235 |
| PVR | PVR Cell Adhesion Molecule | Protein Coding | 48 | GC19P069955 | 0.31434235 |
| ROS1 | ROS Proto-Oncogene 1, Receptor Tyrosine Kinase | Protein Coding | 48 | GC06M117287 | 0.31434235 |
| STAG2 | Stromal Antigen 2 | Protein Coding | 48 | GC0XP123960 | 0.31434235 |
| STT3A | STT3 Oligosaccharyltransferase Complex Catalytic Subunit A | Protein Coding | 48 | GC11P125592 | 0.31434235 |
| STUB1 | STIP1 Homology And U-Box Containing Protein 1 | Protein Coding | 48 | GC16P012618 | 0.31434235 |
| TKT | Transketolase | Protein Coding | 48 | GC03M053224 | 0.31434235 |
| BLVRA | Biliverdin Reductase A | Protein Coding | 47 | GC07P043758 | 0.31434235 |
| ELOVL5 | ELOVL Fatty Acid Elongase 5 | Protein Coding | 47 | GC06M053267 | 0.31434235 |
| FADS2 | Fatty Acid Desaturase 2 | Protein Coding | 47 | GC11P061792 | 0.31434235 |
| GLRX | Glutaredoxin | Protein Coding | 47 | GC05M095752 | 0.31434235 |
| GPC4 | Glypican 4 | Protein Coding | 47 | GC0XM133300 | 0.31434235 |
| HSPA6 | Heat Shock Protein Family A (Hsp70) Member 6 | Protein Coding | 47 | GC01P161524 | 0.31434235 |
| LATS2 | Large Tumor Suppressor Kinase 2 | Protein Coding | 47 | GC13M020973 | 0.31434235 |
| LPP | LIM Domain Containing Preferred Translocation Partner In Lipoma | Protein Coding | 47 | GC03P188153 | 0.31434235 |
| MSX1 | Msh Homeobox 1 | Protein Coding | 47 | GC04P004861 | 0.31434235 |
| PEX1 | Peroxisomal Biogenesis Factor 1 | Protein Coding | 47 | GC07M092487 | 0.31434235 |
| PRKN | Parkin RBR E3 Ubiquitin Protein Ligase | Protein Coding | 47 | GC06M161348 | 0.31434235 |
| PXDN | Peroxidasin | Protein Coding | 47 | GC02M001635 | 0.31434235 |
| RPN1 | Ribophorin I | Protein Coding | 47 | GC03M128619 | 0.31434235 |
| TMPRSS6 | Transmembrane Serine Protease 6 | Protein Coding | 47 | GC22M059410 | 0.31434235 |
| TNNI2 | Troponin I2, Fast Skeletal Type | Protein Coding | 47 | GC11P002031 | 0.31434235 |
| UGT8 | UDP Glycosyltransferase 8 | Protein Coding | 47 | GC04P114598 | 0.31434235 |
| XRCC5 | X-Ray Repair Cross Complementing 5 | Protein Coding | 47 | GC02P216107 | 0.31434235 |
| ATP2B1 | ATPase Plasma Membrane Ca2+ Transporting 1 | Protein Coding | 46 | GC12M089588 | 0.31434235 |
| ATP6AP1 | ATPase H+ Transporting Accessory Protein 1 | Protein Coding | 46 | GC0XP154428 | 0.31434235 |
| CUX1 | Cut Like Homeobox 1 | Protein Coding | 46 | GC07P101815 | 0.31434235 |
| ESRRG | Estrogen Related Receptor Gamma | Protein Coding | 46 | GC01M216503 | 0.31434235 |
| FGF5 | Fibroblast Growth Factor 5 | Protein Coding | 46 | GC04P080266 | 0.31434235 |
| MAPK6 | Mitogen-Activated Protein Kinase 6 | Protein Coding | 46 | GC15P051952 | 0.31434235 |
| STT3B | STT3 Oligosaccharyltransferase Complex Catalytic Subunit B | Protein Coding | 46 | GC03P031550 | 0.31434235 |
| TP53BP1 | Tumor Protein P53 Binding Protein 1 | Protein Coding | 46 | GC15M043403 | 0.31434235 |
| UGCG | UDP-Glucose Ceramide Glucosyltransferase | Protein Coding | 46 | GC09P111896 | 0.31434235 |
| VASP | Vasodilator Stimulated Phosphoprotein | Protein Coding | 46 | GC19P069987 | 0.31434235 |
| AMD1 | Adenosylmethionine Decarboxylase 1 | Protein Coding | 45 | GC06P110814 | 0.31434235 |
| ANO1 | Anoctamin 1 | Protein Coding | 45 | GC11P070715 | 0.31434235 |
| DDX20 | DEAD-Box Helicase 20 | Protein Coding | 45 | GC01P111755 | 0.31434235 |
| GABARAP | GABA Type A Receptor-Associated Protein | Protein Coding | 45 | GC17M007240 | 0.31434235 |
| GPR39 | G Protein-Coupled Receptor 39 | Protein Coding | 45 | GC02P137392 | 0.31434235 |
| H2AX | H2A.X Variant Histone | Protein Coding | 45 | GC11M119250 | 0.31434235 |
| KERA | Keratocan | Protein Coding | 45 | GC12M091050 | 0.31434235 |
| LAMC3 | Laminin Subunit Gamma 3 | Protein Coding | 45 | GC09P131009 | 0.31434235 |
| PAK5 | P21 (RAC1) Activated Kinase 5 | Protein Coding | 45 | GC20M009538 | 0.31434235 |
| PHEX | Phosphate Regulating Endopeptidase X-Linked | Protein Coding | 45 | GC0XP022032 | 0.31434235 |
| PLD3 | Phospholipase D Family Member 3 | Protein Coding | 45 | GC19P040348 | 0.31434235 |
| PPP1R1B | Protein Phosphatase 1 Regulatory Inhibitor Subunit 1B | Protein Coding | 45 | GC17P039626 | 0.31434235 |
| RPS6 | Ribosomal Protein S6 | Protein Coding | 45 | GC09M019375 | 0.31434235 |
| RSPO2 | R-Spondin 2 | Protein Coding | 45 | GC08M107899 | 0.31434235 |
| SLAMF7 | SLAM Family Member 7 | Protein Coding | 45 | GC01P160740 | 0.31434235 |
| SLC39A4 | Solute Carrier Family 39 Member 4 | Protein Coding | 45 | GC08M144409 | 0.31434235 |
| TSFM | Ts Translation Elongation Factor, Mitochondrial | Protein Coding | 45 | GC12P057778 | 0.31434235 |
| ACTR3 | Actin Related Protein 3 | Protein Coding | 44 | GC02P113889 | 0.31434235 |
| ARTN | Artemin | Protein Coding | 44 | GC01P043933 | 0.31434235 |
| BIRC7 | Baculoviral IAP Repeat Containing 7 | Protein Coding | 44 | GC20P063235 | 0.31434235 |
| EEA1 | Early Endosome Antigen 1 | Protein Coding | 44 | GC12M092770 | 0.31434235 |
| ELP3 | Elongator Acetyltransferase Complex Subunit 3 | Protein Coding | 44 | GC08P028089 | 0.31434235 |
| EPB41L3 | Erythrocyte Membrane Protein Band 4.1 Like 3 | Protein Coding | 44 | GC18M005392 | 0.31434235 |
| ERF | ETS2 Repressor Factor | Protein Coding | 44 | GC19M042247 | 0.31434235 |
| KIF2A | Kinesin Family Member 2A | Protein Coding | 44 | GC05P062306 | 0.31434235 |
| KIR3DL1 | Killer Cell Immunoglobulin Like Receptor, Three Ig Domains And Long Cytoplasmic Tail 1 | Protein Coding | 44 | GC19P070471 | 0.31434235 |
| LNX1 | Ligand Of Numb-Protein X 1 | Protein Coding | 44 | GC04M053459 | 0.31434235 |
| LRRC8A | Leucine Rich Repeat Containing 8 VRAC Subunit A | Protein Coding | 44 | GC09P128882 | 0.31434235 |
| MBD3 | Methyl-CpG Binding Domain Protein 3 | Protein Coding | 44 | GC19M005932 | 0.31434235 |
| MYL1 | Myosin Light Chain 1 | Protein Coding | 44 | GC02M210290 | 0.31434235 |
| NDN | Necdin, MAGE Family Member | Protein Coding | 44 | GC15M024251 | 0.31434235 |
| NPY4R | Neuropeptide Y Receptor Y4 | Protein Coding | 44 | GC10M046461 | 0.31434235 |
| NUCB1 | Nucleobindin 1 | Protein Coding | 44 | GC19P048900 | 0.31434235 |
| PI4K2A | Phosphatidylinositol 4-Kinase Type 2 Alpha | Protein Coding | 44 | GC10P097640 | 0.31434235 |
| PIGT | Phosphatidylinositol Glycan Anchor Biosynthesis Class T | Protein Coding | 44 | GC20P045416 | 0.31434235 |
| PLS1 | Plastin 1 | Protein Coding | 44 | GC03P142596 | 0.31434235 |
| RAB10 | RAB10, Member RAS Oncogene Family | Protein Coding | 44 | GC02P026033 | 0.31434235 |
| RAPGEF1 | Rap Guanine Nucleotide Exchange Factor 1 | Protein Coding | 44 | GC09M131576 | 0.31434235 |
| RPL12 | Ribosomal Protein L12 | Protein Coding | 44 | GC09M127447 | 0.31434235 |
| SUZ12 | SUZ12 Polycomb Repressive Complex 2 Subunit | Protein Coding | 44 | GC17P031937 | 0.31434235 |
| TDG | Thymine DNA Glycosylase | Protein Coding | 44 | GC12P103965 | 0.31434235 |
| TMED10 | Transmembrane P24 Trafficking Protein 10 | Protein Coding | 44 | GC14M075145 | 0.31434235 |
| TPBG | Trophoblast Glycoprotein | Protein Coding | 44 | GC06P088436 | 0.31434235 |
| TPX2 | TPX2 Microtubule Nucleation Factor | Protein Coding | 44 | GC20P031739 | 0.31434235 |
| AQP8 | Aquaporin 8 | Protein Coding | 43 | GC16P028131 | 0.31434235 |
| CCL28 | C-C Motif Chemokine Ligand 28 | Protein Coding | 43 | GC05M043356 | 0.31434235 |
| ERAP2 | Endoplasmic Reticulum Aminopeptidase 2 | Protein Coding | 43 | GC05P096875 | 0.31434235 |
| GORASP1 | Golgi Reassembly Stacking Protein 1 | Protein Coding | 43 | GC03M039096 | 0.31434235 |
| KLRB1 | Killer Cell Lectin Like Receptor B1 | Protein Coding | 43 | GC12M021835 | 0.31434235 |
| LIMS1 | LIM Zinc Finger Domain Containing 1 | Protein Coding | 43 | GC02P108534 | 0.31434235 |
| PAX4 | Paired Box 4 | Protein Coding | 43 | GC07M127610 | 0.31434235 |
| PIWIL1 | Piwi Like RNA-Mediated Gene Silencing 1 | Protein Coding | 43 | GC12P130337 | 0.31434235 |
| PRDM2 | PR/SET Domain 2 | Protein Coding | 43 | GC01P013700 | 0.31434235 |
| RBP1 | Retinol Binding Protein 1 | Protein Coding | 43 | GC03M139517 | 0.31434235 |
| SALL2 | Spalt Like Transcription Factor 2 | Protein Coding | 43 | GC14M021521 | 0.31434235 |
| SENP2 | SUMO Specific Peptidase 2 | Protein Coding | 43 | GC03P185582 | 0.31434235 |
| TCTN3 | Tectonic Family Member 3 | Protein Coding | 43 | GC10M095663 | 0.31434235 |
| TRAIP | TRAF Interacting Protein | Protein Coding | 43 | GC03M051436 | 0.31434235 |
| CRMP1 | Collapsin Response Mediator Protein 1 | Protein Coding | 42 | GC04M005750 | 0.31434235 |
| GORASP2 | Golgi Reassembly Stacking Protein 2 | Protein Coding | 42 | GC02P170928 | 0.31434235 |
| IER3 | Immediate Early Response 3 | Protein Coding | 42 | GC06M030743 | 0.31434235 |
| IRX2 | Iroquois Homeobox 2 | Protein Coding | 42 | GC05M002708 | 0.31434235 |
| KIF13B | Kinesin Family Member 13B | Protein Coding | 42 | GC08M029067 | 0.31434235 |
| MATN2 | Matrilin 2 | Protein Coding | 42 | GC08P097868 | 0.31434235 |
| PCDH10 | Protocadherin 10 | Protein Coding | 42 | GC04P133149 | 0.31434235 |
| SLC52A2 | Solute Carrier Family 52 Member 2 | Protein Coding | 42 | GC08P144333 | 0.31434235 |
| STXBP3 | Syntaxin Binding Protein 3 | Protein Coding | 42 | GC01P108746 | 0.31434235 |
| TOR1B | Torsin Family 1 Member B | Protein Coding | 42 | GC09P129803 | 0.31434235 |
| CDX1 | Caudal Type Homeobox 1 | Protein Coding | 41 | GC05P150166 | 0.31434235 |
| CHPF | Chondroitin Polymerizing Factor | Protein Coding | 41 | GC02M219538 | 0.31434235 |
| CPA5 | Carboxypeptidase A5 | Protein Coding | 41 | GC07P130344 | 0.31434235 |
| ELP4 | Elongator Acetyltransferase Complex Subunit 4 | Protein Coding | 41 | GC11P031509 | 0.31434235 |
| FLRT2 | Fibronectin Leucine Rich Transmembrane Protein 2 | Protein Coding | 41 | GC14P085527 | 0.31434235 |
| LAMP3 | Lysosomal Associated Membrane Protein 3 | Protein Coding | 41 | GC03M183122 | 0.31434235 |
| NANOG | Nanog Homeobox | Protein Coding | 41 | GC12P007787 | 0.31434235 |
| NLRC5 | NLR Family CARD Domain Containing 5 | Protein Coding | 41 | GC16P057121 | 0.31434235 |
| RAB27B | RAB27B, Member RAS Oncogene Family | Protein Coding | 41 | GC18P054717 | 0.31434235 |
| STMN2 | Stathmin 2 | Protein Coding | 41 | GC08P079610 | 0.31434235 |
| BET1 | Bet1 Golgi Vesicular Membrane Trafficking Protein | Protein Coding | 40 | GC07M093962 | 0.31434235 |
| CADM4 | Cell Adhesion Molecule 4 | Protein Coding | 40 | GC19M043622 | 0.31434235 |
| CLDN15 | Claudin 15 | Protein Coding | 40 | GC07M101232 | 0.31434235 |
| CNNM3 | Cyclin And CBS Domain Divalent Metal Cation Transport Mediator 3 | Protein Coding | 40 | GC02P096815 | 0.31434235 |
| EMC1 | ER Membrane Protein Complex Subunit 1 | Protein Coding | 40 | GC01M019215 | 0.31434235 |
| ERGIC2 | ERGIC And Golgi 2 | Protein Coding | 40 | GC12M029337 | 0.31434235 |
| OTUD7A | OTU Deubiquitinase 7A | Protein Coding | 40 | GC15M031475 | 0.31434235 |
| RAB21 | RAB21, Member RAS Oncogene Family | Protein Coding | 40 | GC12P071754 | 0.31434235 |
| RPL32 | Ribosomal Protein L32 | Protein Coding | 40 | GC03M012834 | 0.31434235 |
| TCF21 | Transcription Factor 21 | Protein Coding | 40 | GC06P133889 | 0.31434235 |
| TRIM39 | Tripartite Motif Containing 39 | Protein Coding | 40 | GC06P087694 | 0.31434235 |
| BAG6 | BAG Cochaperone 6 | Protein Coding | 39 | GC06M031639 | 0.31434235 |
| SLC39A9 | Solute Carrier Family 39 Member 9 | Protein Coding | 39 | GC14P069398 | 0.31434235 |
| ASTN1 | Astrotactin 1 | Protein Coding | 38 | GC01M176857 | 0.31434235 |
| BRMS1 | BRMS1 Transcriptional Repressor And Anoikis Regulator | Protein Coding | 38 | GC11M097113 | 0.31434235 |
| PEAK1 | Pseudopodium Enriched Atypical Kinase 1 | Protein Coding | 38 | GC15M077100 | 0.31434235 |
| CARD16 | Caspase Recruitment Domain Family Member 16 | Protein Coding | 37 | GC11M105041 | 0.31434235 |
| MMRN2 | Multimerin 2 | Protein Coding | 37 | GC10M086935 | 0.31434235 |
| CSRNP1 | Cysteine And Serine Rich Nuclear Protein 1 | Protein Coding | 36 | GC03M039159 | 0.31434235 |
| FAM234A | Family With Sequence Similarity 234 Member A | Protein Coding | 36 | GC16P012596 | 0.31434235 |
| RHBDD1 | Rhomboid Domain Containing 1 | Protein Coding | 36 | GC02P226805 | 0.31434235 |
| SLC48A1 | Solute Carrier Family 48 Member 1 | Protein Coding | 36 | GC12P047753 | 0.31434235 |
| SMAGP | Small Cell Adhesion Glycoprotein | Protein Coding | 36 | GC12M051244 | 0.31434235 |
| ECRG4 | ECRG4 Augurin Precursor | Protein Coding | 34 | GC02P106063 | 0.31434235 |
| NME9 | NME/NM23 Family Member 9 | Protein Coding | 33 | GC03M138261 | 0.31434235 |
| C19orf48 | Chromosome 19 Putative Open Reading Frame 48 | Pseudogene | 31 | GC19M050797 | 0.31434235 |
| CNTNAP3B | Contactin Associated Protein Family Member 3B | Protein Coding | 31 | GC09M041890 | 0.31434235 |
| CT45A1 | Cancer/Testis Antigen Family 45 Member A1 | Protein Coding | 30 | GC0XP135713 | 0.31434235 |
| MIR30E | MicroRNA 30e | RNA Gene | 25 | GC01P040754 | 0.31434235 |
| LINC00473 | Long Intergenic Non-Protein Coding RNA 473 | RNA Gene | 23 | GC06M165328 | 0.31434235 |
| MIR135B | MicroRNA 135b | RNA Gene | 23 | GC01M205448 | 0.31434235 |
| MIR192 | MicroRNA 192 | RNA Gene | 23 | GC11M064891 | 0.31434235 |
| MIR196B | MicroRNA 196b | RNA Gene | 23 | GC07M027633 | 0.31434235 |
| MIR23A | MicroRNA 23a | RNA Gene | 23 | GC19M014545 | 0.31434235 |
| MIR107 | MicroRNA 107 | RNA Gene | 22 | GC10M089600 | 0.31434235 |
| MIR135A1 | MicroRNA 135a-1 | RNA Gene | 22 | GC03M052296 | 0.31434235 |
| MIR181C | MicroRNA 181c | RNA Gene | 22 | GC19P014583 | 0.31434235 |
| MIR1-2 | MicroRNA 1-2 | RNA Gene | 20 | GC18M025565 | 0.31434235 |
| MIR493 | MicroRNA 493 | RNA Gene | 20 | GC14P110453 | 0.31434235 |
| ACTA2-AS1 | ACTA2 Antisense RNA 1 | RNA Gene | 18 | GC10P088932 | 0.31434235 |
| LUCAT1 | Lung Cancer Associated Transcript 1 | RNA Gene | 18 | GC05M091054 | 0.31434235 |
| MIR7-1 | MicroRNA 7-1 | RNA Gene | 18 | GC09M095071 | 0.31434235 |
| MIR92A2 | MicroRNA 92a-2 | RNA Gene | 18 | GC0XM134323 | 0.31434235 |
| GHET1 | Gastric Carcinoma Proliferation Enhancing Transcript 1 | RNA Gene | 15 | GC07P149647 | 0.31434235 |
| MIR5196 | MicroRNA 5196 | RNA Gene | 15 | GC19P069624 | 0.31434235 |
| THBS2-AS1 | THBS2 Antisense RNA 1 | RNA Gene | 13 | GC06P169215 | 0.31434235 |
| MIR4262 | MicroRNA 4262 | RNA Gene | 12 | GC02M011836 | 0.31434235 |
| ST2 | Suppression Of Tumorigenicity 2 | Genetic Locus | 8 | GC11U990127 | 0.31434235 |
| RET | Ret Proto-Oncogene | Protein Coding | 57 | GC10P043208 | 0.309945524 |
| PTPRF | Protein Tyrosine Phosphatase Receptor Type F | Protein Coding | 52 | GC01P043527 | 0.309945524 |
| FZD2 | Frizzled Class Receptor 2 | Protein Coding | 51 | GC17P044557 | 0.309945524 |
| HCK | HCK Proto-Oncogene, Src Family Tyrosine Kinase | Protein Coding | 51 | GC20P032052 | 0.309945524 |
| KYNU | Kynureninase | Protein Coding | 51 | GC02P142877 | 0.309945524 |
| MYL2 | Myosin Light Chain 2 | Protein Coding | 51 | GC12M110910 | 0.309945524 |
| NCSTN | Nicastrin | Protein Coding | 50 | GC01P160343 | 0.309945524 |
| OGG1 | 8-Oxoguanine DNA Glycosylase | Protein Coding | 50 | GC03P012644 | 0.309945524 |
| EPHB3 | EPH Receptor B3 | Protein Coding | 49 | GC03P184561 | 0.309945524 |
| ITGAX | Integrin Subunit Alpha X | Protein Coding | 49 | GC16P041969 | 0.309945524 |
| ABL2 | ABL Proto-Oncogene 2, Non-Receptor Tyrosine Kinase | Protein Coding | 48 | GC01M179137 | 0.309945524 |
| HABP2 | Hyaluronan Binding Protein 2 | Protein Coding | 48 | GC10P113550 | 0.309945524 |
| MYOD1 | Myogenic Differentiation 1 | Protein Coding | 48 | GC11P017741 | 0.309945524 |
| NRP2 | Neuropilin 2 | Protein Coding | 48 | GC02P205681 | 0.309945524 |
| CFD | Complement Factor D | Protein Coding | 47 | GC19P000859 | 0.309945524 |
| EFEMP2 | EGF Containing Fibulin Extracellular Matrix Protein 2 | Protein Coding | 47 | GC11M097089 | 0.309945524 |
| GDF2 | Growth Differentiation Factor 2 | Protein Coding | 47 | GC10P047322 | 0.309945524 |
| PLXNA1 | Plexin A1 | Protein Coding | 47 | GC03P126988 | 0.309945524 |
| PRDX3 | Peroxiredoxin 3 | Protein Coding | 47 | GC10M119167 | 0.309945524 |
| PTPRK | Protein Tyrosine Phosphatase Receptor Type K | Protein Coding | 47 | GC06M127949 | 0.309945524 |
| HCLS1 | Hematopoietic Cell-Specific Lyn Substrate 1 | Protein Coding | 46 | GC03M121631 | 0.309945524 |
| ITIH4 | Inter-Alpha-Trypsin Inhibitor Heavy Chain 4 | Protein Coding | 45 | GC03M052812 | 0.309945524 |
| TRADD | TNFRSF1A Associated Via Death Domain | Protein Coding | 45 | GC16M067154 | 0.309945524 |
| EXOC7 | Exocyst Complex Component 7 | Protein Coding | 44 | GC17M076080 | 0.309945524 |
| NRTN | Neurturin | Protein Coding | 44 | GC19P005805 | 0.309945524 |
| PRDX4 | Peroxiredoxin 4 | Protein Coding | 44 | GC0XP023665 | 0.309945524 |
| AEBP1 | AE Binding Protein 1 | Protein Coding | 43 | GC07P044106 | 0.309945524 |
| ANKH | ANKH Inorganic Pyrophosphate Transport Regulator | Protein Coding | 43 | GC05M014732 | 0.309945524 |
| G3BP1 | G3BP Stress Granule Assembly Factor 1 | Protein Coding | 43 | GC05P151771 | 0.309945524 |
| IFITM1 | Interferon Induced Transmembrane Protein 1 | Protein Coding | 43 | GC11P000313 | 0.309945524 |
| OGN | Osteoglycin | Protein Coding | 43 | GC09M092383 | 0.309945524 |
| CDKL1 | Cyclin Dependent Kinase Like 1 | Protein Coding | 42 | GC14M050330 | 0.309945524 |
| HTATIP2 | HIV-1 Tat Interactive Protein 2 | Protein Coding | 42 | GC11P020363 | 0.309945524 |
| AIF1 | Allograft Inflammatory Factor 1 | Protein Coding | 41 | GC06P087730 | 0.309945524 |
| EYA2 | EYA Transcriptional Coactivator And Phosphatase 2 | Protein Coding | 41 | GC20P046894 | 0.309945524 |
| SLC16A4 | Solute Carrier Family 16 Member 4 | Protein Coding | 41 | GC01M110362 | 0.309945524 |
| DPT | Dermatopontin | Protein Coding | 40 | GC01M168696 | 0.309945524 |
| ST7 | Suppression Of Tumorigenicity 7 | Protein Coding | 38 | GC07P117090 | 0.309945524 |
| SEL1L3 | SEL1L Family Member 3 | Protein Coding | 37 | GC04M025715 | 0.309945524 |
| SCX | Scleraxis BHLH Transcription Factor | Protein Coding | 32 | GC08P144515 | 0.309945524 |
| MIR370 | MicroRNA 370 | RNA Gene | 23 | GC14P110111 | 0.309945524 |
| TNXA | Tenascin XA (Pseudogene) | Pseudogene | 23 | GC06M069230 | 0.309945524 |
| MIR302A | MicroRNA 302a | RNA Gene | 22 | GC04M112780 | 0.309945524 |
| MIR365A | MicroRNA 365a | RNA Gene | 21 | GC16P014309 | 0.309945524 |
| CLEC12A | C-Type Lectin Domain Family 12 Member A | Protein Coding | 42 | GC12P009951 | 0.283698291 |
| IDH1 | Isocitrate Dehydrogenase (NADP(+)) 1 | Protein Coding | 56 | GC02M208236 | 0.282758296 |
| PIK3CD | Phosphatidylinositol-4,5-Bisphosphate 3-Kinase Catalytic Subunit Delta | Protein Coding | 56 | GC01P009629 | 0.282758296 |
| TYK2 | Tyrosine Kinase 2 | Protein Coding | 55 | GC19M010350 | 0.282758296 |
| UCHL1 | Ubiquitin C-Terminal Hydrolase L1 | Protein Coding | 55 | GC04P041256 | 0.282758296 |
| TFRC | Transferrin Receptor | Protein Coding | 54 | GC03M196031 | 0.282758296 |
| B2M | Beta-2-Microglobulin | Protein Coding | 52 | GC15P044711 | 0.282758296 |
| CHEK1 | Checkpoint Kinase 1 | Protein Coding | 52 | GC11P125625 | 0.282758296 |
| MEF2C | Myocyte Enhancer Factor 2C | Protein Coding | 51 | GC05M088718 | 0.282758296 |
| ALDH1A1 | Aldehyde Dehydrogenase 1 Family Member A1 | Protein Coding | 50 | GC09M072900 | 0.282758296 |
| CDH11 | Cadherin 11 | Protein Coding | 50 | GC16M064943 | 0.282758296 |
| ESRRB | Estrogen Related Receptor Beta | Protein Coding | 50 | GC14P076310 | 0.282758296 |
| KDM6A | Lysine Demethylase 6A | Protein Coding | 50 | GC0XP044873 | 0.282758296 |
| PRMT5 | Protein Arginine Methyltransferase 5 | Protein Coding | 50 | GC14M022920 | 0.282758296 |
| TMPRSS2 | Transmembrane Serine Protease 2 | Protein Coding | 50 | GC21M041464 | 0.282758296 |
| TSHR | Thyroid Stimulating Hormone Receptor | Protein Coding | 50 | GC14P080954 | 0.282758296 |
| CA4 | Carbonic Anhydrase 4 | Protein Coding | 49 | GC17P060149 | 0.282758296 |
| PFN1 | Profilin 1 | Protein Coding | 49 | GC17M004945 | 0.282758296 |
| ADRA1D | Adrenoceptor Alpha 1D | Protein Coding | 48 | GC20M004220 | 0.282758296 |
| CALCRL | Calcitonin Receptor Like Receptor | Protein Coding | 48 | GC02M187341 | 0.282758296 |
| MYH10 | Myosin Heavy Chain 10 | Protein Coding | 48 | GC17M008474 | 0.282758296 |
| PAX3 | Paired Box 3 | Protein Coding | 48 | GC02M222199 | 0.282758296 |
| PITX2 | Paired Like Homeodomain 2 | Protein Coding | 48 | GC04M110617 | 0.282758296 |
| SMARCB1 | SWI/SNF Related, Matrix Associated, Actin Dependent Regulator Of Chromatin, Subfamily B, Member 1 | Protein Coding | 48 | GC22P023786 | 0.282758296 |
| SPRY2 | Sprouty RTK Signaling Antagonist 2 | Protein Coding | 48 | GC13M080335 | 0.282758296 |
| YWHAZ | Tyrosine 3-Monooxygenase/Tryptophan 5-Monooxygenase Activation Protein Zeta | Protein Coding | 48 | GC08M100917 | 0.282758296 |
| APEX1 | Apurinic/Apyrimidinic Endodeoxyribonuclease 1 | Protein Coding | 47 | GC14P020455 | 0.282758296 |
| ARF1 | ADP Ribosylation Factor 1 | Protein Coding | 47 | GC01P228082 | 0.282758296 |
| CLOCK | Clock Circadian Regulator | Protein Coding | 47 | GC04M055427 | 0.282758296 |
| MFAP5 | Microfibril Associated Protein 5 | Protein Coding | 47 | GC12M008637 | 0.282758296 |
| NCOR1 | Nuclear Receptor Corepressor 1 | Protein Coding | 47 | GC17M016029 | 0.282758296 |
| NRCAM | Neuronal Cell Adhesion Molecule | Protein Coding | 47 | GC07M108147 | 0.282758296 |
| PLA2G1B | Phospholipase A2 Group IB | Protein Coding | 47 | GC12M120322 | 0.282758296 |
| PRDM1 | PR/SET Domain 1 | Protein Coding | 47 | GC06P105993 | 0.282758296 |
| RAN | RAN, Member RAS Oncogene Family | Protein Coding | 47 | GC12P130871 | 0.282758296 |
| ROBO4 | Roundabout Guidance Receptor 4 | Protein Coding | 47 | GC11M124883 | 0.282758296 |
| SETD7 | SET Domain Containing 7, Histone Lysine Methyltransferase | Protein Coding | 47 | GC04M139495 | 0.282758296 |
| SKI | SKI Proto-Oncogene | Protein Coding | 47 | GC01P002228 | 0.282758296 |
| ABCG1 | ATP Binding Cassette Subfamily G Member 1 | Protein Coding | 46 | GC21P042199 | 0.282758296 |
| CD244 | CD244 Molecule | Protein Coding | 46 | GC01M160830 | 0.282758296 |
| SAT1 | Spermidine/Spermine N1-Acetyltransferase 1 | Protein Coding | 46 | GC0XP023784 | 0.282758296 |
| TNXB | Tenascin XB | Protein Coding | 46 | GC06M069227 | 0.282758296 |
| ADGRG6 | Adhesion G Protein-Coupled Receptor G6 | Protein Coding | 45 | GC06P142301 | 0.282758296 |
| APH1A | Aph-1 Homolog A, Gamma-Secretase Subunit | Protein Coding | 45 | GC01M150265 | 0.282758296 |
| CDC20 | Cell Division Cycle 20 | Protein Coding | 45 | GC01P043358 | 0.282758296 |
| FFAR4 | Free Fatty Acid Receptor 4 | Protein Coding | 45 | GC10P093566 | 0.282758296 |
| GAP43 | Growth Associated Protein 43 | Protein Coding | 45 | GC03P115623 | 0.282758296 |
| SLC5A8 | Solute Carrier Family 5 Member 8 | Protein Coding | 45 | GC12M101155 | 0.282758296 |
| STK39 | Serine/Threonine Kinase 39 | Protein Coding | 45 | GC02M167954 | 0.282758296 |
| COL12A1 | Collagen Type XII Alpha 1 Chain | Protein Coding | 44 | GC06M075084 | 0.282758296 |
| ENTPD2 | Ectonucleoside Triphosphate Diphosphohydrolase 2 | Protein Coding | 44 | GC09M137048 | 0.282758296 |
| FLOT2 | Flotillin 2 | Protein Coding | 44 | GC17M044992 | 0.282758296 |
| HMGB2 | High Mobility Group Box 2 | Protein Coding | 44 | GC04M173331 | 0.282758296 |
| LRP1B | LDL Receptor Related Protein 1B | Protein Coding | 44 | GC02M140231 | 0.282758296 |
| LTBR | Lymphotoxin Beta Receptor | Protein Coding | 44 | GC12P006375 | 0.282758296 |
| MGST1 | Microsomal Glutathione S-Transferase 1 | Protein Coding | 44 | GC12P016347 | 0.282758296 |
| MYOCD | Myocardin | Protein Coding | 44 | GC17P012665 | 0.282758296 |
| NFYA | Nuclear Transcription Factor Y Subunit Alpha | Protein Coding | 44 | GC06P087955 | 0.282758296 |
| OPHN1 | Oligophrenin 1 | Protein Coding | 44 | GC0XM067949 | 0.282758296 |
| RAD18 | RAD18 E3 Ubiquitin Protein Ligase | Protein Coding | 44 | GC03M008775 | 0.282758296 |
| RRAS | RAS Related | Protein Coding | 44 | GC19M049635 | 0.282758296 |
| SLIT3 | Slit Guidance Ligand 3 | Protein Coding | 44 | GC05M168661 | 0.282758296 |
| SREBF2 | Sterol Regulatory Element Binding Transcription Factor 2 | Protein Coding | 44 | GC22P041833 | 0.282758296 |
| THBS3 | Thrombospondin 3 | Protein Coding | 44 | GC01M155195 | 0.282758296 |
| BRAP | BRCA1 Associated Protein | Protein Coding | 43 | GC12M111642 | 0.282758296 |
| FGL2 | Fibrinogen Like 2 | Protein Coding | 43 | GC07M077193 | 0.282758296 |
| ID4 | Inhibitor Of DNA Binding 4, HLH Protein | Protein Coding | 43 | GC06P019837 | 0.282758296 |
| KLF11 | KLF Transcription Factor 11 | Protein Coding | 43 | GC02P010044 | 0.282758296 |
| MSMB | Microseminoprotein Beta | Protein Coding | 43 | GC10M046033 | 0.282758296 |
| NPTX1 | Neuronal Pentraxin 1 | Protein Coding | 43 | GC17M080466 | 0.282758296 |
| EMB | Embigin | Protein Coding | 42 | GC05M050396 | 0.282758296 |
| GALNT1 | Polypeptide N-Acetylgalactosaminyltransferase 1 | Protein Coding | 42 | GC18P035581 | 0.282758296 |
| SCUBE2 | Signal Peptide, CUB Domain And EGF Like Domain Containing 2 | Protein Coding | 42 | GC11M009019 | 0.282758296 |
| SUN2 | Sad1 And UNC84 Domain Containing 2 | Protein Coding | 42 | GC22M059429 | 0.282758296 |
| API5 | Apoptosis Inhibitor 5 | Protein Coding | 41 | GC11P043311 | 0.282758296 |
| BCAR3 | BCAR3 Adaptor Protein, NSP Family Member | Protein Coding | 41 | GC01M093561 | 0.282758296 |
| MOXD1 | Monooxygenase DBH Like 1 | Protein Coding | 41 | GC06M132296 | 0.282758296 |
| NFKBIZ | NFKB Inhibitor Zeta | Protein Coding | 41 | GC03P101827 | 0.282758296 |
| NFYB | Nuclear Transcription Factor Y Subunit Beta | Protein Coding | 41 | GC12M104117 | 0.282758296 |
| ALKBH3 | AlkB Homolog 3, Alpha-Ketoglutarate Dependent Dioxygenase | Protein Coding | 40 | GC11P043902 | 0.282758296 |
| COL20A1 | Collagen Type XX Alpha 1 Chain | Protein Coding | 40 | GC20P063293 | 0.282758296 |
| HOXC6 | Homeobox C6 | Protein Coding | 40 | GC12P053990 | 0.282758296 |
| IL17D | Interleukin 17D | Protein Coding | 40 | GC13P020702 | 0.282758296 |
| P4HTM | Prolyl 4-Hydroxylase, Transmembrane | Protein Coding | 40 | GC03P049846 | 0.282758296 |
| TNFSF9 | TNF Superfamily Member 9 | Protein Coding | 40 | GC19P006531 | 0.282758296 |
| TRIM44 | Tripartite Motif Containing 44 | Protein Coding | 40 | GC11P035684 | 0.282758296 |
| TSPAN13 | Tetraspanin 13 | Protein Coding | 40 | GC07P016753 | 0.282758296 |
| TWSG1 | Twisted Gastrulation BMP Signaling Modulator 1 | Protein Coding | 40 | GC18P009334 | 0.282758296 |
| ZNF24 | Zinc Finger Protein 24 | Protein Coding | 40 | GC18M035332 | 0.282758296 |
| CRIP2 | Cysteine Rich Protein 2 | Protein Coding | 39 | GC14P105472 | 0.282758296 |
| DNAJB4 | DnaJ Heat Shock Protein Family (Hsp40) Member B4 | Protein Coding | 39 | GC01P077979 | 0.282758296 |
| SKA1 | Spindle And Kinetochore Associated Complex Subunit 1 | Protein Coding | 39 | GC18P050374 | 0.282758296 |
| CLEC2B | C-Type Lectin Domain Family 2 Member B | Protein Coding | 38 | GC12M021838 | 0.282758296 |
| SPAG16 | Sperm Associated Antigen 16 | Protein Coding | 38 | GC02P213284 | 0.282758296 |
| STARD8 | StAR Related Lipid Transfer Domain Containing 8 | Protein Coding | 38 | GC0XP068647 | 0.282758296 |
| ARMC8 | Armadillo Repeat Containing 8 | Protein Coding | 37 | GC03P138187 | 0.282758296 |
| CACUL1 | CDK2 Associated Cullin Domain 1 | Protein Coding | 36 | GC10M118674 | 0.282758296 |
| CIP2A | Cellular Inhibitor Of PP2A | Protein Coding | 36 | GC03M108578 | 0.282758296 |
| MIAT | Myocardial Infarction Associated Transcript | RNA Gene | 26 | GC22P026646 | 0.282758296 |
| MIR212 | MicroRNA 212 | RNA Gene | 23 | GC17M002050 | 0.282758296 |
| MIR186 | MicroRNA 186 | RNA Gene | 22 | GC01M071067 | 0.282758296 |
| MIR216A | MicroRNA 216a | RNA Gene | 22 | GC02M055988 | 0.282758296 |
| MIRLET7B | MicroRNA Let-7b | RNA Gene | 22 | GC22P046119 | 0.282758296 |
| MIR199B | MicroRNA 199b | RNA Gene | 21 | GC09M128244 | 0.282758296 |
| MIR30D | MicroRNA 30d | RNA Gene | 21 | GC08M134804 | 0.282758296 |
| MIR19B1 | MicroRNA 19b-1 | RNA Gene | 20 | GC13P091564 | 0.282758296 |
| MIR377 | MicroRNA 377 | RNA Gene | 20 | GC14P110440 | 0.282758296 |
| MIR299 | MicroRNA 299 | RNA Gene | 19 | GC14P110428 | 0.282758296 |
| MIR543 | MicroRNA 543 | RNA Gene | 18 | GC14P110155 | 0.282758296 |
| MIR663A | MicroRNA 663a | RNA Gene | 18 | GC20M026189 | 0.282758296 |
| MIR19B2 | MicroRNA 19b-2 | RNA Gene | 16 | GC0XM134324 | 0.282758296 |
| MIR634 | MicroRNA 634 | RNA Gene | 16 | GC17P066787 | 0.282758296 |
| ACVR1 | Activin A Receptor Type 1 | Protein Coding | 54 | GC02M157736 | 0.280226648 |
| CAMK2A | Calcium/Calmodulin Dependent Protein Kinase II Alpha | Protein Coding | 54 | GC05M150219 | 0.280226648 |
| TOP2A | DNA Topoisomerase II Alpha | Protein Coding | 54 | GC17M040388 | 0.280226648 |
| ABCC1 | ATP Binding Cassette Subfamily C Member 1 | Protein Coding | 52 | GC16P015949 | 0.280226648 |
| BLM | BLM RecQ Like Helicase | Protein Coding | 51 | GC15P090717 | 0.280226648 |
| TNNI3 | Troponin I3, Cardiac Type | Protein Coding | 51 | GC19M055151 | 0.280226648 |
| ALDH1A2 | Aldehyde Dehydrogenase 1 Family Member A2 | Protein Coding | 50 | GC15M118500 | 0.280226648 |
| GATA4 | GATA Binding Protein 4 | Protein Coding | 50 | GC08P011676 | 0.280226648 |
| PCK1 | Phosphoenolpyruvate Carboxykinase 1 | Protein Coding | 50 | GC20P057561 | 0.280226648 |
| EHMT2 | Euchromatic Histone Lysine Methyltransferase 2 | Protein Coding | 48 | GC06M031879 | 0.280226648 |
| EIF2S3 | Eukaryotic Translation Initiation Factor 2 Subunit Gamma | Protein Coding | 48 | GC0XP024054 | 0.280226648 |
| GCLC | Glutamate-Cysteine Ligase Catalytic Subunit | Protein Coding | 47 | GC06M053497 | 0.280226648 |
| PIAS1 | Protein Inhibitor Of Activated STAT 1 | Protein Coding | 47 | GC15P068054 | 0.280226648 |
| DCK | Deoxycytidine Kinase | Protein Coding | 46 | GC04P070992 | 0.280226648 |
| TRIM28 | Tripartite Motif Containing 28 | Protein Coding | 46 | GC19P058544 | 0.280226648 |
| ANXA3 | Annexin A3 | Protein Coding | 45 | GC04P078551 | 0.280226648 |
| BEST1 | Bestrophin 1 | Protein Coding | 45 | GC11P061949 | 0.280226648 |
| CHD3 | Chromodomain Helicase DNA Binding Protein 3 | Protein Coding | 45 | GC17P011474 | 0.280226648 |
| DAXX | Death Domain Associated Protein | Protein Coding | 45 | GC06M033318 | 0.280226648 |
| WNT2 | Wnt Family Member 2 | Protein Coding | 45 | GC07M117309 | 0.280226648 |
| ZFYVE9 | Zinc Finger FYVE-Type Containing 9 | Protein Coding | 45 | GC01P052142 | 0.280226648 |
| CCDC6 | Coiled-Coil Domain Containing 6 | Protein Coding | 44 | GC10M059788 | 0.280226648 |
| CLASP1 | Cytoplasmic Linker Associated Protein 1 | Protein Coding | 44 | GC02M121337 | 0.280226648 |
| GALNT14 | Polypeptide N-Acetylgalactosaminyltransferase 14 | Protein Coding | 44 | GC02M030888 | 0.280226648 |
| GCNT1 | Glucosaminyl (N-Acetyl) Transferase 1 | Protein Coding | 44 | GC09P076420 | 0.280226648 |
| GPC5 | Glypican 5 | Protein Coding | 44 | GC13P091398 | 0.280226648 |
| HSPE1 | Heat Shock Protein Family E (Hsp10) Member 1 | Protein Coding | 44 | GC02P197501 | 0.280226648 |
| HIBADH | 3-Hydroxyisobutyrate Dehydrogenase | Protein Coding | 43 | GC07M027525 | 0.280226648 |
| ITGB3BP | Integrin Subunit Beta 3 Binding Protein | Protein Coding | 43 | GC01M063440 | 0.280226648 |
| ITIH1 | Inter-Alpha-Trypsin Inhibitor Heavy Chain 1 | Protein Coding | 43 | GC03P052777 | 0.280226648 |
| MAP4 | Microtubule Associated Protein 4 | Protein Coding | 43 | GC03M047850 | 0.280226648 |
| PRMT6 | Protein Arginine Methyltransferase 6 | Protein Coding | 43 | GC01P107056 | 0.280226648 |
| BTF3 | Basic Transcription Factor 3 | Protein Coding | 42 | GC05P073498 | 0.280226648 |
| ARHGAP35 | Rho GTPase Activating Protein 35 | Protein Coding | 41 | GC19P046860 | 0.280226648 |
| L3MBTL1 | L3MBTL Histone Methyl-Lysine Binding Protein 1 | Protein Coding | 41 | GC20P043582 | 0.280226648 |
| NECTIN3 | Nectin Cell Adhesion Molecule 3 | Protein Coding | 41 | GC03P111071 | 0.280226648 |
| CBLL1 | Cbl Proto-Oncogene Like 1 | Protein Coding | 40 | GC07P107743 | 0.280226648 |
| CLASP2 | Cytoplasmic Linker Associated Protein 2 | Protein Coding | 40 | GC03M033537 | 0.280226648 |
| COPS6 | COP9 Signalosome Subunit 6 | Protein Coding | 40 | GC07P100088 | 0.280226648 |
| SNX27 | Sorting Nexin 27 | Protein Coding | 40 | GC01P151611 | 0.280226648 |
| CDK5RAP3 | CDK5 Regulatory Subunit Associated Protein 3 | Protein Coding | 39 | GC17P047967 | 0.280226648 |
| NUDT21 | Nudix Hydrolase 21 | Protein Coding | 39 | GC16M056429 | 0.280226648 |
| MPP7 | MAGUK P55 Scaffold Protein 7 | Protein Coding | 38 | GC10M028057 | 0.280226648 |
| TUBA3D | Tubulin Alpha 3d | Protein Coding | 36 | GC02P137324 | 0.280226648 |
| TNFAIP8L2 | TNF Alpha Induced Protein 8 Like 2 | Protein Coding | 33 | GC01P151156 | 0.280226648 |
| MIR185 | MicroRNA 185 | RNA Gene | 24 | GC22P039755 | 0.280226648 |
| MIR301A | MicroRNA 301a | RNA Gene | 22 | GC17M059151 | 0.280226648 |
| MIR539 | MicroRNA 539 | RNA Gene | 20 | GC14P110458 | 0.280226648 |
| HAGLR | HOXD Antisense Growth-Associated Long Non-Coding RNA | RNA Gene | 19 | GC02M176173 | 0.280226648 |
| MIR188 | MicroRNA 188 | RNA Gene | 19 | GC0XP050003 | 0.280226648 |
| MIR320B1 | MicroRNA 320b-1 | RNA Gene | 19 | GC01P116671 | 0.280226648 |
| GATA6-AS1 | GATA6 Antisense RNA 1 (Head To Head) | RNA Gene | 18 | GC18M025568 | 0.280226648 |
| LINC00460 | Long Intergenic Non-Protein Coding RNA 460 | RNA Gene | 18 | GC13P106376 | 0.280226648 |
| MIR224 | MicroRNA 224 | RNA Gene | 18 | GC0XM151958 | 0.280226648 |
| LOC408186 | Alpha-2-Macroglobulin Like 1 Pseudogene | Pseudogene | 12 | GC12P023076 | 0.280226648 |
| BMPR2 | Bone Morphogenetic Protein Receptor Type 2 | Protein Coding | 54 | GC02P202376 | 0.247327179 |
| NEK2 | NIMA Related Kinase 2 | Protein Coding | 54 | GC01M211658 | 0.247327179 |
| PRKAA2 | Protein Kinase AMP-Activated Catalytic Subunit Alpha 2 | Protein Coding | 54 | GC01P056645 | 0.247327179 |
| PRKDC | Protein Kinase, DNA-Activated, Catalytic Subunit | Protein Coding | 53 | GC08M047773 | 0.247327179 |
| SCN5A | Sodium Voltage-Gated Channel Alpha Subunit 5 | Protein Coding | 53 | GC03M038549 | 0.247327179 |
| ACVR2B | Activin A Receptor Type 2B | Protein Coding | 52 | GC03P038453 | 0.247327179 |
| ENTPD1 | Ectonucleoside Triphosphate Diphosphohydrolase 1 | Protein Coding | 52 | GC10P095711 | 0.247327179 |
| KCNMA1 | Potassium Calcium-Activated Channel Subfamily M Alpha 1 | Protein Coding | 52 | GC10M076869 | 0.247327179 |
| PIM1 | Pim-1 Proto-Oncogene, Serine/Threonine Kinase | Protein Coding | 52 | GC06P087916 | 0.247327179 |
| PTCH1 | Patched 1 | Protein Coding | 52 | GC09M095442 | 0.247327179 |
| SCN8A | Sodium Voltage-Gated Channel Alpha Subunit 8 | Protein Coding | 52 | GC12P051590 | 0.247327179 |
| SLC1A3 | Solute Carrier Family 1 Member 3 | Protein Coding | 52 | GC05P036650 | 0.247327179 |
| SMARCA2 | SWI/SNF Related, Matrix Associated, Actin Dependent Regulator Of Chromatin, Subfamily A, Member 2 | Protein Coding | 52 | GC09P001980 | 0.247327179 |
| TPO | Thyroid Peroxidase | Protein Coding | 52 | GC02P001374 | 0.247327179 |
| ASAH1 | N-Acylsphingosine Amidohydrolase 1 | Protein Coding | 51 | GC08M018055 | 0.247327179 |
| GLUL | Glutamate-Ammonia Ligase | Protein Coding | 51 | GC01M182378 | 0.247327179 |
| IKBKG | Inhibitor Of Nuclear Factor Kappa B Kinase Regulatory Subunit Gamma | Protein Coding | 51 | GC0XP154541 | 0.247327179 |
| KIF11 | Kinesin Family Member 11 | Protein Coding | 51 | GC10P092574 | 0.247327179 |
| MASP1 | MBL Associated Serine Protease 1 | Protein Coding | 51 | GC03M187216 | 0.247327179 |
| MC4R | Melanocortin 4 Receptor | Protein Coding | 51 | GC18M060371 | 0.247327179 |
| MST1R | Macrophage Stimulating 1 Receptor | Protein Coding | 51 | GC03M051443 | 0.247327179 |
| OPRM1 | Opioid Receptor Mu 1 | Protein Coding | 51 | GC06P154107 | 0.247327179 |
| RRM2B | Ribonucleotide Reductase Regulatory TP53 Inducible Subunit M2B | Protein Coding | 51 | GC08M102204 | 0.247327179 |
| SLC12A5 | Solute Carrier Family 12 Member 5 | Protein Coding | 51 | GC20P046021 | 0.247327179 |
| TPM1 | Tropomyosin 1 | Protein Coding | 51 | GC15P123360 | 0.247327179 |
| TRPV4 | Transient Receptor Potential Cation Channel Subfamily V Member 4 | Protein Coding | 51 | GC12M109783 | 0.247327179 |
| YES1 | YES Proto-Oncogene 1, Src Family Tyrosine Kinase | Protein Coding | 51 | GC18M000721 | 0.247327179 |
| ARSA | Arylsulfatase A | Protein Coding | 50 | GC22M050622 | 0.247327179 |
| ATF6 | Activating Transcription Factor 6 | Protein Coding | 50 | GC01P161766 | 0.247327179 |
| AVPR2 | Arginine Vasopressin Receptor 2 | Protein Coding | 50 | GC0XP153902 | 0.247327179 |
| DGKE | Diacylglycerol Kinase Epsilon | Protein Coding | 50 | GC17P056834 | 0.247327179 |
| ESRRA | Estrogen Related Receptor Alpha | Protein Coding | 50 | GC11P064305 | 0.247327179 |
| EXT1 | Exostosin Glycosyltransferase 1 | Protein Coding | 50 | GC08M117798 | 0.247327179 |
| KAT2A | Lysine Acetyltransferase 2A | Protein Coding | 50 | GC17M042113 | 0.247327179 |
| MC2R | Melanocortin 2 Receptor | Protein Coding | 50 | GC18M025519 | 0.247327179 |
| MTNR1B | Melatonin Receptor 1B | Protein Coding | 50 | GC11P092969 | 0.247327179 |
| NBN | Nibrin | Protein Coding | 50 | GC08M089933 | 0.247327179 |
| NCF4 | Neutrophil Cytosolic Factor 4 | Protein Coding | 50 | GC22P036860 | 0.247327179 |
| PRCP | Prolylcarboxypeptidase | Protein Coding | 50 | GC11M097554 | 0.247327179 |
| ABCD1 | ATP Binding Cassette Subfamily D Member 1 | Protein Coding | 49 | GC0XP153724 | 0.247327179 |
| EIF4EBP1 | Eukaryotic Translation Initiation Factor 4E Binding Protein 1 | Protein Coding | 49 | GC08P038535 | 0.247327179 |
| GATA1 | GATA Binding Protein 1 | Protein Coding | 49 | GC0XP048786 | 0.247327179 |
| GSS | Glutathione Synthetase | Protein Coding | 49 | GC20M034928 | 0.247327179 |
| MDH1 | Malate Dehydrogenase 1 | Protein Coding | 49 | GC02P063557 | 0.247327179 |
| RDX | Radixin | Protein Coding | 49 | GC11M109864 | 0.247327179 |
| RPS6KA2 | Ribosomal Protein S6 Kinase A2 | Protein Coding | 49 | GC06M166409 | 0.247327179 |
| TPM2 | Tropomyosin 2 | Protein Coding | 49 | GC09M035672 | 0.247327179 |
| ADAR | Adenosine Deaminase RNA Specific | Protein Coding | 48 | GC01M154582 | 0.247327179 |
| AKR1C3 | Aldo-Keto Reductase Family 1 Member C3 | Protein Coding | 48 | GC10P005035 | 0.247327179 |
| ARAF | A-Raf Proto-Oncogene, Serine/Threonine Kinase | Protein Coding | 48 | GC0XP047646 | 0.247327179 |
| ATP7A | ATPase Copper Transporting Alpha | Protein Coding | 48 | GC0XP078001 | 0.247327179 |
| CAMK4 | Calcium/Calmodulin Dependent Protein Kinase IV | Protein Coding | 48 | GC05P111223 | 0.247327179 |
| CBLB | Cbl Proto-Oncogene B | Protein Coding | 48 | GC03M105655 | 0.247327179 |
| CHRM1 | Cholinergic Receptor Muscarinic 1 | Protein Coding | 48 | GC11M096948 | 0.247327179 |
| CSF2RA | Colony Stimulating Factor 2 Receptor Subunit Alpha | Protein Coding | 48 | GC0XP001795 | 0.247327179 |
| CTSZ | Cathepsin Z | Protein Coding | 48 | GC20M058995 | 0.247327179 |
| EGLN3 | Egl-9 Family Hypoxia Inducible Factor 3 | Protein Coding | 48 | GC14M033924 | 0.247327179 |
| ERAP1 | Endoplasmic Reticulum Aminopeptidase 1 | Protein Coding | 48 | GC05M096760 | 0.247327179 |
| FLNC | Filamin C | Protein Coding | 48 | GC07P128830 | 0.247327179 |
| FTO | FTO Alpha-Ketoglutarate Dependent Dioxygenase | Protein Coding | 48 | GC16P053853 | 0.247327179 |
| HSF1 | Heat Shock Transcription Factor 1 | Protein Coding | 48 | GC08P144291 | 0.247327179 |
| IDUA | Alpha-L-Iduronidase | Protein Coding | 48 | GC04P000986 | 0.247327179 |
| LIMK2 | LIM Domain Kinase 2 | Protein Coding | 48 | GC22P031212 | 0.247327179 |
| LONP1 | Lon Peptidase 1, Mitochondrial | Protein Coding | 48 | GC19M005691 | 0.247327179 |
| MYL3 | Myosin Light Chain 3 | Protein Coding | 48 | GC03M046836 | 0.247327179 |
| NTF4 | Neurotrophin 4 | Protein Coding | 48 | GC19M068158 | 0.247327179 |
| NUMB | NUMB Endocytic Adaptor Protein | Protein Coding | 48 | GC14M073275 | 0.247327179 |
| PLOD2 | Procollagen-Lysine,2-Oxoglutarate 5-Dioxygenase 2 | Protein Coding | 48 | GC03M146069 | 0.247327179 |
| PRKD3 | Protein Kinase D3 | Protein Coding | 48 | GC02M037251 | 0.247327179 |
| RIGI | RNA Sensor RIG-I | Protein Coding | 48 | GC09M032458 | 0.247327179 |
| SGPL1 | Sphingosine-1-Phosphate Lyase 1 | Protein Coding | 48 | GC10P070815 | 0.247327179 |
| SGSH | N-Sulfoglucosamine Sulfohydrolase | Protein Coding | 48 | GC17M080206 | 0.247327179 |
| SIN3A | SIN3 Transcription Regulator Family Member A | Protein Coding | 48 | GC15M075369 | 0.247327179 |
| SLC12A4 | Solute Carrier Family 12 Member 4 | Protein Coding | 48 | GC16M068117 | 0.247327179 |
| SORL1 | Sortilin Related Receptor 1 | Protein Coding | 48 | GC11P121452 | 0.247327179 |
| TRAF3 | TNF Receptor Associated Factor 3 | Protein Coding | 48 | GC14P110165 | 0.247327179 |
| ABCD3 | ATP Binding Cassette Subfamily D Member 3 | Protein Coding | 47 | GC01P094385 | 0.247327179 |
| AMACR | Alpha-Methylacyl-CoA Racemase | Protein Coding | 47 | GC05M033986 | 0.247327179 |
| APPL1 | Adaptor Protein, Phosphotyrosine Interacting With PH Domain And Leucine Zipper 1 | Protein Coding | 47 | GC03P057227 | 0.247327179 |
| CBR1 | Carbonyl Reductase 1 | Protein Coding | 47 | GC21P036069 | 0.247327179 |
| CDC7 | Cell Division Cycle 7 | Protein Coding | 47 | GC01P091500 | 0.247327179 |
| DGKA | Diacylglycerol Kinase Alpha | Protein Coding | 47 | GC12P055927 | 0.247327179 |
| DUOX2 | Dual Oxidase 2 | Protein Coding | 47 | GC15M045092 | 0.247327179 |
| EPS15 | Epidermal Growth Factor Receptor Pathway Substrate 15 | Protein Coding | 47 | GC01M051354 | 0.247327179 |
| H6PD | Hexose-6-Phosphate Dehydrogenase/Glucose 1-Dehydrogenase | Protein Coding | 47 | GC01P009234 | 0.247327179 |
| HIF1AN | Hypoxia Inducible Factor 1 Subunit Alpha Inhibitor | Protein Coding | 47 | GC10P100529 | 0.247327179 |
| HINT1 | Histidine Triad Nucleotide Binding Protein 1 | Protein Coding | 47 | GC05M131159 | 0.247327179 |
| MAN2B1 | Mannosidase Alpha Class 2B Member 1 | Protein Coding | 47 | GC19M012663 | 0.247327179 |
| MAPRE1 | Microtubule Associated Protein RP/EB Family Member 1 | Protein Coding | 47 | GC20P032819 | 0.247327179 |
| NLK | Nemo Like Kinase | Protein Coding | 47 | GC17P057740 | 0.247327179 |
| PLTP | Phospholipid Transfer Protein | Protein Coding | 47 | GC20M045898 | 0.247327179 |
| PRKCSH | Protein Kinase C Substrate 80K-H | Protein Coding | 47 | GC19P011435 | 0.247327179 |
| SEPSECS | Sep (O-Phosphoserine) TRNA:Sec (Selenocysteine) TRNA Synthase | Protein Coding | 47 | GC04M025121 | 0.247327179 |
| SLC1A5 | Solute Carrier Family 1 Member 5 | Protein Coding | 47 | GC19M068072 | 0.247327179 |
| SMS | Spermine Synthase | Protein Coding | 47 | GC0XP021958 | 0.247327179 |
| SYN1 | Synapsin I | Protein Coding | 47 | GC0XM047571 | 0.247327179 |
| TAL1 | TAL BHLH Transcription Factor 1, Erythroid Differentiation Factor | Protein Coding | 47 | GC01M047216 | 0.247327179 |
| TECR | Trans-2,3-Enoyl-CoA Reductase | Protein Coding | 47 | GC19P014517 | 0.247327179 |
| TIE1 | Tyrosine Kinase With Immunoglobulin Like And EGF Like Domains 1 | Protein Coding | 47 | GC01P043300 | 0.247327179 |
| TNFRSF10A | TNF Receptor Superfamily Member 10a | Protein Coding | 47 | GC08M023190 | 0.247327179 |
| VNN1 | Vanin 1 | Protein Coding | 47 | GC06M132680 | 0.247327179 |
| WIF1 | WNT Inhibitory Factor 1 | Protein Coding | 47 | GC12M065050 | 0.247327179 |
| BAMBI | BMP And Activin Membrane Bound Inhibitor | Protein Coding | 46 | GC10P028685 | 0.247327179 |
| CLCN5 | Chloride Voltage-Gated Channel 5 | Protein Coding | 46 | GC0XP049922 | 0.247327179 |
| GIPC1 | GIPC PDZ Domain Containing Family Member 1 | Protein Coding | 46 | GC19M014578 | 0.247327179 |
| GPR55 | G Protein-Coupled Receptor 55 | Protein Coding | 46 | GC02M230907 | 0.247327179 |
| OPTN | Optineurin | Protein Coding | 46 | GC10P013099 | 0.247327179 |
| PDE6G | Phosphodiesterase 6G | Protein Coding | 46 | GC17M081650 | 0.247327179 |
| POFUT1 | Protein O-Fucosyltransferase 1 | Protein Coding | 46 | GC20P032207 | 0.247327179 |
| POMT2 | Protein O-Mannosyltransferase 2 | Protein Coding | 46 | GC14M077274 | 0.247327179 |
| PREX1 | Phosphatidylinositol-3,4,5-Trisphosphate Dependent Rac Exchange Factor 1 | Protein Coding | 46 | GC20M048624 | 0.247327179 |
| PSMD4 | Proteasome 26S Subunit Ubiquitin Receptor, Non-ATPase 4 | Protein Coding | 46 | GC01P151274 | 0.247327179 |
| SMURF2 | SMAD Specific E3 Ubiquitin Protein Ligase 2 | Protein Coding | 46 | GC17M064542 | 0.247327179 |
| SOX3 | SRY-Box Transcription Factor 3 | Protein Coding | 46 | GC0XM140502 | 0.247327179 |
| TCF7 | Transcription Factor 7 | Protein Coding | 46 | GC05P134114 | 0.247327179 |
| USP2 | Ubiquitin Specific Peptidase 2 | Protein Coding | 46 | GC11M119355 | 0.247327179 |
| AHCYL1 | Adenosylhomocysteinase Like 1 | Protein Coding | 45 | GC01P109984 | 0.247327179 |
| ANGPT4 | Angiopoietin 4 | Protein Coding | 45 | GC20M000869 | 0.247327179 |
| APLNR | Apelin Receptor | Protein Coding | 45 | GC11M057233 | 0.247327179 |
| ATXN1 | Ataxin 1 | Protein Coding | 45 | GC06M016299 | 0.247327179 |
| CALB1 | Calbindin 1 | Protein Coding | 45 | GC08M090058 | 0.247327179 |
| CDC42BPA | CDC42 Binding Protein Kinase Alpha | Protein Coding | 45 | GC01M226989 | 0.247327179 |
| CNDP2 | Carnosine Dipeptidase 2 | Protein Coding | 45 | GC18P074495 | 0.247327179 |
| CRTAP | Cartilage Associated Protein | Protein Coding | 45 | GC03P033113 | 0.247327179 |
| CUL5 | Cullin 5 | Protein Coding | 45 | GC11P108008 | 0.247327179 |
| CYP4B1 | Cytochrome P450 Family 4 Subfamily B Member 1 | Protein Coding | 45 | GC01P046757 | 0.247327179 |
| DNASE1 | Deoxyribonuclease 1 | Protein Coding | 45 | GC16P003611 | 0.247327179 |
| EDEM1 | ER Degradation Enhancing Alpha-Mannosidase Like Protein 1 | Protein Coding | 45 | GC03P005187 | 0.247327179 |
| FABP7 | Fatty Acid Binding Protein 7 | Protein Coding | 45 | GC06P122815 | 0.247327179 |
| FKBP10 | FKBP Prolyl Isomerase 10 | Protein Coding | 45 | GC17P041812 | 0.247327179 |
| GCNT2 | Glucosaminyl (N-Acetyl) Transferase 2 (I Blood Group) | Protein Coding | 45 | GC06P010492 | 0.247327179 |
| HEY1 | Hes Related Family BHLH Transcription Factor With YRPW Motif 1 | Protein Coding | 45 | GC08M079764 | 0.247327179 |
| IRF2 | Interferon Regulatory Factor 2 | Protein Coding | 45 | GC04M184387 | 0.247327179 |
| KRT13 | Keratin 13 | Protein Coding | 45 | GC17M041500 | 0.247327179 |
| LAIR1 | Leukocyte Associated Immunoglobulin Like Receptor 1 | Protein Coding | 45 | GC19M054351 | 0.247327179 |
| MANBA | Mannosidase Beta | Protein Coding | 45 | GC04M102631 | 0.247327179 |
| MIP | Major Intrinsic Protein Of Lens Fiber | Protein Coding | 45 | GC12M056449 | 0.247327179 |
| NR2E1 | Nuclear Receptor Subfamily 2 Group E Member 1 | Protein Coding | 45 | GC06P108166 | 0.247327179 |
| PABPC1 | Poly(A) Binding Protein Cytoplasmic 1 | Protein Coding | 45 | GC08M100685 | 0.247327179 |
| PARVA | Parvin Alpha | Protein Coding | 45 | GC11P012398 | 0.247327179 |
| PHOX2B | Paired Like Homeobox 2B | Protein Coding | 45 | GC04M041746 | 0.247327179 |
| PLXNA2 | Plexin A2 | Protein Coding | 45 | GC01M208023 | 0.247327179 |
| REST | RE1 Silencing Transcription Factor | Protein Coding | 45 | GC04P056907 | 0.247327179 |
| RGS4 | Regulator Of G Protein Signaling 4 | Protein Coding | 45 | GC01P163038 | 0.247327179 |
| ROM1 | Retinal Outer Segment Membrane Protein 1 | Protein Coding | 45 | GC11P062611 | 0.247327179 |
| RYK | Receptor Like Tyrosine Kinase | Protein Coding | 45 | GC03M134065 | 0.247327179 |
| SIX1 | SIX Homeobox 1 | Protein Coding | 45 | GC14M060643 | 0.247327179 |
| SRI | Sorcin | Protein Coding | 45 | GC07M088205 | 0.247327179 |
| SSB | Small RNA Binding Exonuclease Protection Factor La | Protein Coding | 45 | GC02P169791 | 0.247327179 |
| USP4 | Ubiquitin Specific Peptidase 4 | Protein Coding | 45 | GC03M049277 | 0.247327179 |
| VANGL1 | VANGL Planar Cell Polarity Protein 1 | Protein Coding | 45 | GC01P115641 | 0.247327179 |
| VPS35 | VPS35 Retromer Complex Component | Protein Coding | 45 | GC16M047022 | 0.247327179 |
| XYLT1 | Xylosyltransferase 1 | Protein Coding | 45 | GC16M017101 | 0.247327179 |
| ABCA2 | ATP Binding Cassette Subfamily A Member 2 | Protein Coding | 44 | GC09M137007 | 0.247327179 |
| ARHGEF11 | Rho Guanine Nucleotide Exchange Factor 11 | Protein Coding | 44 | GC01M156934 | 0.247327179 |
| BCAP31 | B Cell Receptor Associated Protein 31 | Protein Coding | 44 | GC0XM153701 | 0.247327179 |
| BMP3 | Bone Morphogenetic Protein 3 | Protein Coding | 44 | GC04P081030 | 0.247327179 |
| CCRL2 | C-C Motif Chemokine Receptor Like 2 | Protein Coding | 44 | GC03P046407 | 0.247327179 |
| CYP4V2 | Cytochrome P450 Family 4 Subfamily V Member 2 | Protein Coding | 44 | GC04P186191 | 0.247327179 |
| EDA | Ectodysplasin A | Protein Coding | 44 | GC0XP069618 | 0.247327179 |
| EFNA2 | Ephrin A2 | Protein Coding | 44 | GC19P003274 | 0.247327179 |
| ERLIN2 | ER Lipid Raft Associated 2 | Protein Coding | 44 | GC08P037736 | 0.247327179 |
| FABP6 | Fatty Acid Binding Protein 6 | Protein Coding | 44 | GC05P160187 | 0.247327179 |
| FCGR1A | Fc Gamma Receptor Ia | Protein Coding | 44 | GC01P150320 | 0.247327179 |
| FGF13 | Fibroblast Growth Factor 13 | Protein Coding | 44 | GC0XM138615 | 0.247327179 |
| FGF21 | Fibroblast Growth Factor 21 | Protein Coding | 44 | GC19P070140 | 0.247327179 |
| FRG1 | FSHD Region Gene 1 | Protein Coding | 44 | GC04P189940 | 0.247327179 |
| FUCA2 | Alpha-L-Fucosidase 2 | Protein Coding | 44 | GC06M143494 | 0.247327179 |
| IKZF3 | IKAROS Family Zinc Finger 3 | Protein Coding | 44 | GC17M049237 | 0.247327179 |
| IL36RN | Interleukin 36 Receptor Antagonist | Protein Coding | 44 | GC02P123137 | 0.247327179 |
| KDM2A | Lysine Demethylase 2A | Protein Coding | 44 | GC11P067119 | 0.247327179 |
| LDHC | Lactate Dehydrogenase C | Protein Coding | 44 | GC11P018554 | 0.247327179 |
| MC5R | Melanocortin 5 Receptor | Protein Coding | 44 | GC18P013824 | 0.247327179 |
| MICAL1 | Microtubule Associated Monooxygenase, Calponin And LIM Domain Containing 1 | Protein Coding | 44 | GC06M109444 | 0.247327179 |
| MPV17 | Mitochondrial Inner Membrane Protein MPV17 | Protein Coding | 44 | GC02M027309 | 0.247327179 |
| MYL4 | Myosin Light Chain 4 | Protein Coding | 44 | GC17P047189 | 0.247327179 |
| NUP210 | Nucleoporin 210 | Protein Coding | 44 | GC03M021322 | 0.247327179 |
| OS9 | OS9 Endoplasmic Reticulum Lectin | Protein Coding | 44 | GC12P057693 | 0.247327179 |
| P3H1 | Prolyl 3-Hydroxylase 1 | Protein Coding | 44 | GC01M042746 | 0.247327179 |
| PALLD | Palladin, Cytoskeletal Associated Protein | Protein Coding | 44 | GC04P168497 | 0.247327179 |
| PLIN3 | Perilipin 3 | Protein Coding | 44 | GC19M006106 | 0.247327179 |
| PPA1 | Inorganic Pyrophosphatase 1 | Protein Coding | 44 | GC10M070202 | 0.247327179 |
| PSME3 | Proteasome Activator Subunit 3 | Protein Coding | 44 | GC17P042824 | 0.247327179 |
| SETD1A | SET Domain Containing 1A, Histone Lysine Methyltransferase | Protein Coding | 44 | GC16P041938 | 0.247327179 |
| SLC7A6 | Solute Carrier Family 7 Member 6 | Protein Coding | 44 | GC16P068263 | 0.247327179 |
| SPTBN2 | Spectrin Beta, Non-Erythrocytic 2 | Protein Coding | 44 | GC11M097141 | 0.247327179 |
| TIA1 | TIA1 Cytotoxic Granule Associated RNA Binding Protein | Protein Coding | 44 | GC02M070209 | 0.247327179 |
| TRIM32 | Tripartite Motif Containing 32 | Protein Coding | 44 | GC09P116687 | 0.247327179 |
| ARHGAP6 | Rho GTPase Activating Protein 6 | Protein Coding | 43 | GC0XM012585 | 0.247327179 |
| ARHGEF4 | Rho Guanine Nucleotide Exchange Factor 4 | Protein Coding | 43 | GC02P130836 | 0.247327179 |
| B3GALNT1 | Beta-1,3-N-Acetylgalactosaminyltransferase 1 (Globoside Blood Group) | Protein Coding | 43 | GC03M161083 | 0.247327179 |
| B3GALNT2 | Beta-1,3-N-Acetylgalactosaminyltransferase 2 | Protein Coding | 43 | GC01M235440 | 0.247327179 |
| BAG2 | BAG Cochaperone 2 | Protein Coding | 43 | GC06P057172 | 0.247327179 |
| CAB39 | Calcium Binding Protein 39 | Protein Coding | 43 | GC02P230712 | 0.247327179 |
| CCNE2 | Cyclin E2 | Protein Coding | 43 | GC08M094879 | 0.247327179 |
| CD109 | CD109 Molecule | Protein Coding | 43 | GC06P088334 | 0.247327179 |
| CD160 | CD160 Molecule | Protein Coding | 43 | GC01P145719 | 0.247327179 |
| DKK4 | Dickkopf WNT Signaling Pathway Inhibitor 4 | Protein Coding | 43 | GC08M042373 | 0.247327179 |
| DPYSL5 | Dihydropyrimidinase Like 5 | Protein Coding | 43 | GC02P026847 | 0.247327179 |
| DUSP2 | Dual Specificity Phosphatase 2 | Protein Coding | 43 | GC02M098496 | 0.247327179 |
| ECT2 | Epithelial Cell Transforming 2 | Protein Coding | 43 | GC03P172750 | 0.247327179 |
| FAT1 | FAT Atypical Cadherin 1 | Protein Coding | 43 | GC04M186587 | 0.247327179 |
| HIF3A | Hypoxia Inducible Factor 3 Subunit Alpha | Protein Coding | 43 | GC19P046297 | 0.247327179 |
| IL17B | Interleukin 17B | Protein Coding | 43 | GC05M149371 | 0.247327179 |
| IL20RB | Interleukin 20 Receptor Subunit Beta | Protein Coding | 43 | GC03P136946 | 0.247327179 |
| IL22RA2 | Interleukin 22 Receptor Subunit Alpha 2 | Protein Coding | 43 | GC06M137143 | 0.247327179 |
| IPO5 | Importin 5 | Protein Coding | 43 | GC13P097953 | 0.247327179 |
| KLF15 | KLF Transcription Factor 15 | Protein Coding | 43 | GC03M126293 | 0.247327179 |
| LRIG2 | Leucine Rich Repeats And Immunoglobulin Like Domains 2 | Protein Coding | 43 | GC01P113073 | 0.247327179 |
| MBD2 | Methyl-CpG Binding Domain Protein 2 | Protein Coding | 43 | GC18M054151 | 0.247327179 |
| MED1 | Mediator Complex Subunit 1 | Protein Coding | 43 | GC17M039404 | 0.247327179 |
| OSBPL2 | Oxysterol Binding Protein Like 2 | Protein Coding | 43 | GC20P062231 | 0.247327179 |
| PRSS23 | Serine Protease 23 | Protein Coding | 43 | GC11P086791 | 0.247327179 |
| PSMD10 | Proteasome 26S Subunit, Non-ATPase 10 | Protein Coding | 43 | GC0XM108084 | 0.247327179 |
| RAB4A | RAB4A, Member RAS Oncogene Family | Protein Coding | 43 | GC01P229271 | 0.247327179 |
| SGMS2 | Sphingomyelin Synthase 2 | Protein Coding | 43 | GC04P107824 | 0.247327179 |
| SNX9 | Sorting Nexin 9 | Protein Coding | 43 | GC06P157685 | 0.247327179 |
| TSPAN5 | Tetraspanin 5 | Protein Coding | 43 | GC04M098470 | 0.247327179 |
| WWC1 | WW And C2 Domain Containing 1 | Protein Coding | 43 | GC05P168291 | 0.247327179 |
| ZMYND8 | Zinc Finger MYND-Type Containing 8 | Protein Coding | 43 | GC20M047209 | 0.247327179 |
| ALPG | Alkaline Phosphatase, Germ Cell | Protein Coding | 42 | GC02P232407 | 0.247327179 |
| CCNL1 | Cyclin L1 | Protein Coding | 42 | GC03M157146 | 0.247327179 |
| CLTA | Clathrin Light Chain A | Protein Coding | 42 | GC09P036190 | 0.247327179 |
| CLTB | Clathrin Light Chain B | Protein Coding | 42 | GC05M176392 | 0.247327179 |
| COLGALT1 | Collagen Beta(1-O)Galactosyltransferase 1 | Protein Coding | 42 | GC19P017555 | 0.247327179 |
| DSC1 | Desmocollin 1 | Protein Coding | 42 | GC18M031129 | 0.247327179 |
| ELF4 | E74 Like ETS Transcription Factor 4 | Protein Coding | 42 | GC0XM130064 | 0.247327179 |
| FSTL3 | Follistatin Like 3 | Protein Coding | 42 | GC19P000676 | 0.247327179 |
| GABPA | GA Binding Protein Transcription Factor Subunit Alpha | Protein Coding | 42 | GC21P025734 | 0.247327179 |
| LCN1 | Lipocalin 1 | Protein Coding | 42 | GC09P135521 | 0.247327179 |
| MNDA | Myeloid Cell Nuclear Differentiation Antigen | Protein Coding | 42 | GC01P158801 | 0.247327179 |
| NCOA4 | Nuclear Receptor Coactivator 4 | Protein Coding | 42 | GC10M046005 | 0.247327179 |
| RAB31 | RAB31, Member RAS Oncogene Family | Protein Coding | 42 | GC18P009701 | 0.247327179 |
| RAI1 | Retinoic Acid Induced 1 | Protein Coding | 42 | GC17P017919 | 0.247327179 |
| RNF7 | Ring Finger Protein 7 | Protein Coding | 42 | GC03P141738 | 0.247327179 |
| SKIC2 | SKI2 Subunit Of Superkiller Complex | Protein Coding | 42 | GC06P091884 | 0.247327179 |
| SPG11 | SPG11 Vesicle Trafficking Associated, Spatacsin | Protein Coding | 42 | GC15M044562 | 0.247327179 |
| SRSF5 | Serine And Arginine Rich Splicing Factor 5 | Protein Coding | 42 | GC14P069727 | 0.247327179 |
| TMEM165 | Transmembrane Protein 165 | Protein Coding | 42 | GC04P055395 | 0.247327179 |
| TMPRSS4 | Transmembrane Serine Protease 4 | Protein Coding | 42 | GC11P118077 | 0.247327179 |
| TNFSF18 | TNF Superfamily Member 18 | Protein Coding | 42 | GC01M173009 | 0.247327179 |
| VAMP8 | Vesicle Associated Membrane Protein 8 | Protein Coding | 42 | GC02P085561 | 0.247327179 |
| WNK2 | WNK Lysine Deficient Protein Kinase 2 | Protein Coding | 42 | GC09P093184 | 0.247327179 |
| ACTN3 | Actinin Alpha 3 | Protein Coding | 41 | GC11P066546 | 0.247327179 |
| ATAD2 | ATPase Family AAA Domain Containing 2 | Protein Coding | 41 | GC08M123319 | 0.247327179 |
| CIAPIN1 | Cytokine Induced Apoptosis Inhibitor 1 | Protein Coding | 41 | GC16M057428 | 0.247327179 |
| CLEC11A | C-Type Lectin Domain Containing 11A | Protein Coding | 41 | GC19P050723 | 0.247327179 |
| CLIC2 | Chloride Intracellular Channel 2 | Protein Coding | 41 | GC0XM155276 | 0.247327179 |
| CLPTM1L | CLPTM1 Like | Protein Coding | 41 | GC05M001317 | 0.247327179 |
| CSPP1 | Centrosome And Spindle Pole Associated Protein 1 | Protein Coding | 41 | GC08P067062 | 0.247327179 |
| EDEM3 | ER Degradation Enhancing Alpha-Mannosidase Like Protein 3 | Protein Coding | 41 | GC01M184690 | 0.247327179 |
| EIF3I | Eukaryotic Translation Initiation Factor 3 Subunit I | Protein Coding | 41 | GC01P032221 | 0.247327179 |
| EXTL2 | Exostosin Like Glycosyltransferase 2 | Protein Coding | 41 | GC01M100872 | 0.247327179 |
| FAM13A | Family With Sequence Similarity 13 Member A | Protein Coding | 41 | GC04M088725 | 0.247327179 |
| FRAS1 | Fraser Extracellular Matrix Complex Subunit 1 | Protein Coding | 41 | GC04P078057 | 0.247327179 |
| FUBP1 | Far Upstream Element Binding Protein 1 | Protein Coding | 41 | GC01M077944 | 0.247327179 |
| GALNT6 | Polypeptide N-Acetylgalactosaminyltransferase 6 | Protein Coding | 41 | GC12M051351 | 0.247327179 |
| GCNT3 | Glucosaminyl (N-Acetyl) Transferase 3, Mucin Type | Protein Coding | 41 | GC15P059594 | 0.247327179 |
| GINS2 | GINS Complex Subunit 2 | Protein Coding | 41 | GC16M085676 | 0.247327179 |
| LMCD1 | LIM And Cysteine Rich Domains 1 | Protein Coding | 41 | GC03P008619 | 0.247327179 |
| LYST | Lysosomal Trafficking Regulator | Protein Coding | 41 | GC01M235661 | 0.247327179 |
| MAFK | MAF BZIP Transcription Factor K | Protein Coding | 41 | GC07P002226 | 0.247327179 |
| MKKS | MKKS Centrosomal Shuttling Protein | Protein Coding | 41 | GC20M010424 | 0.247327179 |
| NDST2 | N-Deacetylase And N-Sulfotransferase 2 | Protein Coding | 41 | GC10M073801 | 0.247327179 |
| NOVA1 | NOVA Alternative Splicing Regulator 1 | Protein Coding | 41 | GC14M026443 | 0.247327179 |
| NUMBL | NUMB Like Endocytic Adaptor Protein | Protein Coding | 41 | GC19M040665 | 0.247327179 |
| PLXNB3 | Plexin B3 | Protein Coding | 41 | GC0XP153764 | 0.247327179 |
| RAB25 | RAB25, Member RAS Oncogene Family | Protein Coding | 41 | GC01P156061 | 0.247327179 |
| SEC24A | SEC24 Homolog A, COPII Coat Complex Component | Protein Coding | 41 | GC05P134647 | 0.247327179 |
| SLC17A9 | Solute Carrier Family 17 Member 9 | Protein Coding | 41 | GC20P062952 | 0.247327179 |
| SNIP1 | Smad Nuclear Interacting Protein 1 | Protein Coding | 41 | GC01M037534 | 0.247327179 |
| TCL1B | TCL1 Family AKT Coactivator B | Protein Coding | 41 | GC14P095686 | 0.247327179 |
| TMEM126A | Transmembrane Protein 126A | Protein Coding | 41 | GC11P085647 | 0.247327179 |
| TMX3 | Thioredoxin Related Transmembrane Protein 3 | Protein Coding | 41 | GC18M068673 | 0.247327179 |
| TNIP1 | TNFAIP3 Interacting Protein 1 | Protein Coding | 41 | GC05M151029 | 0.247327179 |
| TSPAN15 | Tetraspanin 15 | Protein Coding | 41 | GC10P069451 | 0.247327179 |
| TSPAN9 | Tetraspanin 9 | Protein Coding | 41 | GC12P003078 | 0.247327179 |
| VGF | VGF Nerve Growth Factor Inducible | Protein Coding | 41 | GC07M101162 | 0.247327179 |
| ZC3H12A | Zinc Finger CCCH-Type Containing 12A | Protein Coding | 41 | GC01P037474 | 0.247327179 |
| ACKR4 | Atypical Chemokine Receptor 4 | Protein Coding | 40 | GC03P132597 | 0.247327179 |
| AFAP1L2 | Actin Filament Associated Protein 1 Like 2 | Protein Coding | 40 | GC10M114281 | 0.247327179 |
| ARSK | Arylsulfatase Family Member K | Protein Coding | 40 | GC05P095555 | 0.247327179 |
| CAPS | Calcyphosine | Protein Coding | 40 | GC19P005945 | 0.247327179 |
| CHPF2 | Chondroitin Polymerizing Factor 2 | Protein Coding | 40 | GC07P151232 | 0.247327179 |
| CHST7 | Carbohydrate Sulfotransferase 7 | Protein Coding | 40 | GC0XP046619 | 0.247327179 |
| CIRBP | Cold Inducible RNA Binding Protein | Protein Coding | 40 | GC19P001259 | 0.247327179 |
| CPEB1 | Cytoplasmic Polyadenylation Element Binding Protein 1 | Protein Coding | 40 | GC15M082543 | 0.247327179 |
| DNAJC10 | DnaJ Heat Shock Protein Family (Hsp40) Member C10 | Protein Coding | 40 | GC02P182716 | 0.247327179 |
| EPDR1 | Ependymin Related 1 | Protein Coding | 40 | GC07P037926 | 0.247327179 |
| ERGIC3 | ERGIC And Golgi 3 | Protein Coding | 40 | GC20P035542 | 0.247327179 |
| ERLEC1 | Endoplasmic Reticulum Lectin 1 | Protein Coding | 40 | GC02P053786 | 0.247327179 |
| ERP29 | Endoplasmic Reticulum Protein 29 | Protein Coding | 40 | GC12P112013 | 0.247327179 |
| EXOC8 | Exocyst Complex Component 8 | Protein Coding | 40 | GC01M231332 | 0.247327179 |
| FMN1 | Formin 1 | Protein Coding | 40 | GC15M032765 | 0.247327179 |
| GHRH | Growth Hormone Releasing Hormone | Protein Coding | 40 | GC20M037251 | 0.247327179 |
| HVCN1 | Hydrogen Voltage Gated Channel 1 | Protein Coding | 40 | GC12M110627 | 0.247327179 |
| ITFG1 | Integrin Alpha FG-GAP Repeat Containing 1 | Protein Coding | 40 | GC16M047156 | 0.247327179 |
| MCRS1 | Microspherule Protein 1 | Protein Coding | 40 | GC12M049808 | 0.247327179 |
| MOK | MOK Protein Kinase | Protein Coding | 40 | GC14M102214 | 0.247327179 |
| NKX3-2 | NK3 Homeobox 2 | Protein Coding | 40 | GC04M013542 | 0.247327179 |
| PCYOX1 | Prenylcysteine Oxidase 1 | Protein Coding | 40 | GC02P070257 | 0.247327179 |
| PI16 | Peptidase Inhibitor 16 | Protein Coding | 40 | GC06P087912 | 0.247327179 |
| PLA2G15 | Phospholipase A2 Group XV | Protein Coding | 40 | GC16P068245 | 0.247327179 |
| RASA4 | RAS P21 Protein Activator 4 | Protein Coding | 40 | GC07M103251 | 0.247327179 |
| SESN2 | Sestrin 2 | Protein Coding | 40 | GC01P028290 | 0.247327179 |
| SIAE | Sialic Acid Acetylesterase | Protein Coding | 40 | GC11M124633 | 0.247327179 |
| SPATA13 | Spermatogenesis Associated 13 | Protein Coding | 40 | GC13P024029 | 0.247327179 |
| SPRR3 | Small Proline Rich Protein 3 | Protein Coding | 40 | GC01P153001 | 0.247327179 |
| VSIG4 | V-Set And Immunoglobulin Domain Containing 4 | Protein Coding | 40 | GC0XM066021 | 0.247327179 |
| XPO4 | Exportin 4 | Protein Coding | 40 | GC13M020777 | 0.247327179 |
| ZKSCAN1 | Zinc Finger With KRAB And SCAN Domains 1 | Protein Coding | 40 | GC07P100015 | 0.247327179 |
| ARHGAP21 | Rho GTPase Activating Protein 21 | Protein Coding | 39 | GC10M024583 | 0.247327179 |
| CAPRIN1 | Cell Cycle Associated Protein 1 | Protein Coding | 39 | GC11P034051 | 0.247327179 |
| CNOT7 | CCR4-NOT Transcription Complex Subunit 7 | Protein Coding | 39 | GC08M017224 | 0.247327179 |
| MUC13 | Mucin 13, Cell Surface Associated | Protein Coding | 39 | GC03M124905 | 0.247327179 |
| NCLN | Nicalin | Protein Coding | 39 | GC19P003185 | 0.247327179 |
| POMGNT2 | Protein O-Linked Mannose N-Acetylglucosaminyltransferase 2 (Beta 1,4-) | Protein Coding | 39 | GC03M043121 | 0.247327179 |
| REC8 | REC8 Meiotic Recombination Protein | Protein Coding | 39 | GC14P024171 | 0.247327179 |
| RNF146 | Ring Finger Protein 146 | Protein Coding | 39 | GC06P127266 | 0.247327179 |
| TMEM135 | Transmembrane Protein 135 | Protein Coding | 39 | GC11P087037 | 0.247327179 |
| TRIM14 | Tripartite Motif Containing 14 | Protein Coding | 39 | GC09M098035 | 0.247327179 |
| TSPYL2 | TSPY Like 2 | Protein Coding | 39 | GC0XP053082 | 0.247327179 |
| UBE2Q1 | Ubiquitin Conjugating Enzyme E2 Q1 | Protein Coding | 39 | GC01M154548 | 0.247327179 |
| ADGRG2 | Adhesion G Protein-Coupled Receptor G2 | Protein Coding | 38 | GC0XM018989 | 0.247327179 |
| ARHGAP25 | Rho GTPase Activating Protein 25 | Protein Coding | 38 | GC02P068679 | 0.247327179 |
| ATAT1 | Alpha Tubulin Acetyltransferase 1 | Protein Coding | 38 | GC06P030626 | 0.247327179 |
| CBX6 | Chromobox 6 | Protein Coding | 38 | GC22M038861 | 0.247327179 |
| CDK2AP1 | Cyclin Dependent Kinase 2 Associated Protein 1 | Protein Coding | 38 | GC12M123250 | 0.247327179 |
| CLIC3 | Chloride Intracellular Channel 3 | Protein Coding | 38 | GC09M136994 | 0.247327179 |
| CMKLR2 | Chemerin Chemokine-Like Receptor 2 | Protein Coding | 38 | GC02M206176 | 0.247327179 |
| CYP4Z1 | Cytochrome P450 Family 4 Subfamily Z Member 1 | Protein Coding | 38 | GC01P047067 | 0.247327179 |
| DEFA3 | Defensin Alpha 3 | Protein Coding | 38 | GC08M007015 | 0.247327179 |
| GBP5 | Guanylate Binding Protein 5 | Protein Coding | 38 | GC01M089259 | 0.247327179 |
| IFT80 | Intraflagellar Transport 80 | Protein Coding | 38 | GC03M160256 | 0.247327179 |
| LARP4B | La Ribonucleoprotein 4B | Protein Coding | 38 | GC10M000806 | 0.247327179 |
| MBIP | MAP3K12 Binding Inhibitory Protein 1 | Protein Coding | 38 | GC14M036298 | 0.247327179 |
| METRNL | Meteorin Like, Glial Cell Differentiation Regulator | Protein Coding | 38 | GC17P083079 | 0.247327179 |
| OPN1MW | Opsin 1, Medium Wave Sensitive | Protein Coding | 38 | GC0XP154182 | 0.247327179 |
| PSG9 | Pregnancy Specific Beta-1-Glycoprotein 9 | Protein Coding | 38 | GC19M067944 | 0.247327179 |
| RABIF | RAB Interacting Factor | Protein Coding | 38 | GC01M202878 | 0.247327179 |
| RBMS3 | RNA Binding Motif Single Stranded Interacting Protein 3 | Protein Coding | 38 | GC03P028575 | 0.247327179 |
| RPRD1A | Regulation Of Nuclear Pre-MRNA Domain Containing 1A | Protein Coding | 38 | GC18M035984 | 0.247327179 |
| SYNM | Synemin | Protein Coding | 38 | GC15P099098 | 0.247327179 |
| SYTL3 | Synaptotagmin Like 3 | Protein Coding | 38 | GC06P158644 | 0.247327179 |
| TMTC3 | Transmembrane O-Mannosyltransferase Targeting Cadherins 3 | Protein Coding | 38 | GC12P088142 | 0.247327179 |
| ZFYVE27 | Zinc Finger FYVE-Type Containing 27 | Protein Coding | 38 | GC10P097739 | 0.247327179 |
| ZNF350 | Zinc Finger Protein 350 | Protein Coding | 38 | GC19M068330 | 0.247327179 |
| ADGRF1 | Adhesion G Protein-Coupled Receptor F1 | Protein Coding | 37 | GC06M046997 | 0.247327179 |
| AKIRIN2 | Akirin 2 | Protein Coding | 37 | GC06M087674 | 0.247327179 |
| BARX1 | BARX Homeobox 1 | Protein Coding | 37 | GC09M093951 | 0.247327179 |
| FBXO6 | F-Box Protein 6 | Protein Coding | 37 | GC01P011664 | 0.247327179 |
| FNDC3B | Fibronectin Type III Domain Containing 3B | Protein Coding | 37 | GC03P172039 | 0.247327179 |
| FXYD3 | FXYD Domain Containing Ion Transport Regulator 3 | Protein Coding | 37 | GC19P035115 | 0.247327179 |
| IL26 | Interleukin 26 | Protein Coding | 37 | GC12M068201 | 0.247327179 |
| KLHL6 | Kelch Like Family Member 6 | Protein Coding | 37 | GC03M183487 | 0.247327179 |
| KRT24 | Keratin 24 | Protein Coding | 37 | GC17M049374 | 0.247327179 |
| MTPN | Myotrophin | Protein Coding | 37 | GC07M135926 | 0.247327179 |
| MYORG | Myogenesis Regulating Glycosidase (Putative) | Protein Coding | 37 | GC09M034366 | 0.247327179 |
| PCYOX1L | Prenylcysteine Oxidase 1 Like | Protein Coding | 37 | GC05P149358 | 0.247327179 |
| TENM4 | Teneurin Transmembrane Protein 4 | Protein Coding | 37 | GC11M078652 | 0.247327179 |
| TM4SF4 | Transmembrane 4 L Six Family Member 4 | Protein Coding | 37 | GC03P149473 | 0.247327179 |
| TOX4 | TOX High Mobility Group Box Family Member 4 | Protein Coding | 37 | GC14P021476 | 0.247327179 |
| UNC50 | Unc-50 Inner Nuclear Membrane RNA Binding Protein | Protein Coding | 37 | GC02P098591 | 0.247327179 |
| ZBTB7C | Zinc Finger And BTB Domain Containing 7C | Protein Coding | 37 | GC18M048026 | 0.247327179 |
| BTBD7 | BTB Domain Containing 7 | Protein Coding | 36 | GC14M093237 | 0.247327179 |
| CERCAM | Cerebral Endothelial Cell Adhesion Molecule | Protein Coding | 36 | GC09P128411 | 0.247327179 |
| CGB5 | Chorionic Gonadotropin Subunit Beta 5 | Protein Coding | 36 | GC19P049043 | 0.247327179 |
| GPR75 | G Protein-Coupled Receptor 75 | Protein Coding | 36 | GC02M053852 | 0.247327179 |
| MAGEB2 | MAGE Family Member B2 | Protein Coding | 36 | GC0XP030215 | 0.247327179 |
| MLLT11 | MLLT11 Transcription Factor 7 Cofactor | Protein Coding | 36 | GC01P151291 | 0.247327179 |
| SCRG1 | Stimulator Of Chondrogenesis 1 | Protein Coding | 36 | GC04M173384 | 0.247327179 |
| TMEM131 | Transmembrane Protein 131 | Protein Coding | 36 | GC02M098546 | 0.247327179 |
| TMEM41A | Transmembrane Protein 41A | Protein Coding | 36 | GC03M185476 | 0.247327179 |
| POGLUT3 | Protein O-Glucosyltransferase 3 | Protein Coding | 35 | GC11M108473 | 0.247327179 |
| SHE | Src Homology 2 Domain Containing E | Protein Coding | 35 | GC01M154575 | 0.247327179 |
| SPHKAP | SPHK1 Interactor, AKAP Domain Containing | Protein Coding | 35 | GC02M227979 | 0.247327179 |
| CXCL17 | C-X-C Motif Chemokine Ligand 17 | Protein Coding | 34 | GC19M042428 | 0.247327179 |
| CYTL1 | Cytokine Like 1 | Protein Coding | 34 | GC04M005017 | 0.247327179 |
| LRP2BP | LRP2 Binding Protein | Protein Coding | 34 | GC04M185363 | 0.247327179 |
| TMEM106C | Transmembrane Protein 106C | Protein Coding | 34 | GC12P047963 | 0.247327179 |
| TRIM52 | Tripartite Motif Containing 52 | Protein Coding | 33 | GC05M181881 | 0.247327179 |
| C1QTNF12 | C1q And TNF Related 12 | Protein Coding | 31 | GC01M006972 | 0.247327179 |
| INKA2 | Inka Box Actin Regulator 2 | Protein Coding | 31 | GC01M111700 | 0.247327179 |
| TDRD10 | Tudor Domain Containing 10 | Protein Coding | 31 | GC01P154502 | 0.247327179 |
| TMPRSS11F | Transmembrane Serine Protease 11F | Protein Coding | 31 | GC04M068053 | 0.247327179 |
| IGHG3 | Immunoglobulin Heavy Constant Gamma 3 (G3m Marker) | Protein Coding | 30 | GC14M113020 | 0.247327179 |
| CDKN2B-AS1 | CDKN2B Antisense RNA 1 | RNA Gene | 25 | GC09P021994 | 0.247327179 |
| MIR155HG | MIR155 Host Gene | RNA Gene | 25 | GC21P025593 | 0.247327179 |
| TDRG1 | Testis Development Related 1 | RNA Gene | 24 | GC06P087952 | 0.247327179 |
| HAND2-AS1 | HAND2 Antisense RNA 1 | RNA Gene | 23 | GC04P173527 | 0.247327179 |
| LINC00305 | Long Intergenic Non-Protein Coding RNA 305 | RNA Gene | 23 | GC18M064080 | 0.247327179 |
| MIRLET7I | MicroRNA Let-7i | RNA Gene | 23 | GC12P062613 | 0.247327179 |
| DHRS4-AS1 | DHRS4 Antisense RNA 1 | RNA Gene | 22 | GC14M023938 | 0.247327179 |
| HOTAIRM1 | HOXA Transcript Antisense RNA, Myeloid-Specific 1 | RNA Gene | 22 | GC07P027095 | 0.247327179 |
| MIR339 | MicroRNA 339 | RNA Gene | 22 | GC07M001022 | 0.247327179 |
| MIR574 | MicroRNA 574 | RNA Gene | 22 | GC04P038962 | 0.247327179 |
| MIR423 | MicroRNA 423 | RNA Gene | 21 | GC17P030117 | 0.247327179 |
| MIR616 | MicroRNA 616 | RNA Gene | 21 | GC12M057519 | 0.247327179 |
| MIRLET7G | MicroRNA Let-7g | RNA Gene | 21 | GC03M052268 | 0.247327179 |
| MIR129-2 | MicroRNA 129-2 | RNA Gene | 20 | GC11P043786 | 0.247327179 |
| MIR31HG | MIR31 Host Gene | RNA Gene | 20 | GC09M021794 | 0.247327179 |
| MIR526B | MicroRNA 526b | RNA Gene | 20 | GC19P053694 | 0.247327179 |
| MIR770 | MicroRNA 770 | RNA Gene | 20 | GC14P110467 | 0.247327179 |
| LINC00426 | Long Intergenic Non-Protein Coding RNA 426 | RNA Gene | 19 | GC13M030385 | 0.247327179 |
| MIR103A1 | MicroRNA 103a-1 | RNA Gene | 19 | GC05M168560 | 0.247327179 |
| MIR135A2 | MicroRNA 135a-2 | RNA Gene | 19 | GC12P097563 | 0.247327179 |
| MIR517B | MicroRNA 517b | RNA Gene | 19 | GC19P053721 | 0.247327179 |
| MIR517C | MicroRNA 517c | RNA Gene | 19 | GC19P070433 | 0.247327179 |
| MIR520D | MicroRNA 520d | RNA Gene | 19 | GC19P053720 | 0.247327179 |
| MIR520G | MicroRNA 520g | RNA Gene | 19 | GC19P053722 | 0.247327179 |
| MIR608 | MicroRNA 608 | RNA Gene | 19 | GC10P100974 | 0.247327179 |
| MIR766 | MicroRNA 766 | RNA Gene | 19 | GC0XM119646 | 0.247327179 |
| ILF3-DT | ILF3 Divergent Transcript | RNA Gene | 18 | GC19M010652 | 0.247327179 |
| MIR508 | MicroRNA 508 | RNA Gene | 18 | GC0XM147236 | 0.247327179 |
| MIR517A | MicroRNA 517a | RNA Gene | 18 | GC19P053712 | 0.247327179 |
| MIR655 | MicroRNA 655 | RNA Gene | 18 | GC14P110462 | 0.247327179 |
| MIR1303 | MicroRNA 1303 | RNA Gene | 17 | GC05P154685 | 0.247327179 |
| MIR1908 | MicroRNA 1908 | RNA Gene | 17 | GC11M061815 | 0.247327179 |
| MIR2861 | MicroRNA 2861 | RNA Gene | 17 | GC09P127785 | 0.247327179 |
| MIR496 | MicroRNA 496 | RNA Gene | 17 | GC14P110457 | 0.247327179 |
| IL6-AS1 | IL6 Antisense RNA 1 | RNA Gene | 16 | GC07M022728 | 0.247327179 |
| LINC01139 | Long Intergenic Non-Protein Coding RNA 1139 | RNA Gene | 16 | GC01M238480 | 0.247327179 |
| MELTF-AS1 | MELTF Antisense RNA 1 | RNA Gene | 16 | GC03P197000 | 0.247327179 |
| MILIP | MYC Inducible LncRNA Inactivating P53 | RNA Gene | 16 | GC17P081929 | 0.247327179 |
| MIR1236 | MicroRNA 1236 | RNA Gene | 16 | GC06M069229 | 0.247327179 |
| MIR4484 | MicroRNA 4484 | RNA Gene | 16 | GC10P125819 | 0.247327179 |
| MIR450A1 | MicroRNA 450a-1 | RNA Gene | 16 | GC0XM134633 | 0.247327179 |
| MIR450A2 | MicroRNA 450a-2 | RNA Gene | 16 | GC0XM134637 | 0.247327179 |
| MIR599 | MicroRNA 599 | RNA Gene | 16 | GC08M099537 | 0.247327179 |
| CCR5AS | CCR5 Antisense RNA | RNA Gene | 15 | GC03M046365 | 0.247327179 |
| MIR1299 | MicroRNA 1299 | RNA Gene | 15 | GC09M040929 | 0.247327179 |
| MIR2467 | MicroRNA 2467 | RNA Gene | 15 | GC02M239352 | 0.247327179 |
| MIR466 | MicroRNA 466 | RNA Gene | 15 | GC03M031161 | 0.247327179 |
| MIR602 | MicroRNA 602 | RNA Gene | 15 | GC09P137838 | 0.247327179 |
| MIR613 | MicroRNA 613 | RNA Gene | 15 | GC12P022589 | 0.247327179 |
| MIR623 | MicroRNA 623 | RNA Gene | 14 | GC13P099356 | 0.247327179 |
| C2-AS1 | C2 Antisense RNA 1 | RNA Gene | 13 | GC06M069225 | 0.247327179 |
| MIR761 | MicroRNA 761 | RNA Gene | 13 | GC01M051836 | 0.247327179 |
| LINC02444 | Long Intergenic Non-Protein Coding RNA 2444 | RNA Gene | 11 | GC12P072987 | 0.247327179 |
| MIR6745 | MicroRNA 6745 | RNA Gene | 11 | GC11M096708 | 0.247327179 |
| LOC108281116 | SOX18 Promoter Region | Biological Region | 2 | GC20P065147 | 0.247327179 |
| IL4I1 | Interleukin 4 Induced 1 | Protein Coding | 42 | GC19M068198 | 0.24193871 |
| ATR | ATR Serine/Threonine Kinase | Protein Coding | 54 | GC03M142449 | 0.228804097 |
| ACAT1 | Acetyl-CoA Acetyltransferase 1 | Protein Coding | 52 | GC11P108121 | 0.228804097 |
| FBP1 | Fructose-Bisphosphatase 1 | Protein Coding | 52 | GC09M094603 | 0.228804097 |
| GCK | Glucokinase | Protein Coding | 52 | GC07M044528 | 0.228804097 |
| USP7 | Ubiquitin Specific Peptidase 7 | Protein Coding | 52 | GC16M008892 | 0.228804097 |
| ABCA3 | ATP Binding Cassette Subfamily A Member 3 | Protein Coding | 51 | GC16M002275 | 0.228804097 |
| DLL1 | Delta Like Canonical Notch Ligand 1 | Protein Coding | 51 | GC06M170282 | 0.228804097 |
| GYS1 | Glycogen Synthase 1 | Protein Coding | 51 | GC19M068148 | 0.228804097 |
| KCNJ11 | Potassium Inwardly Rectifying Channel Subfamily J Member 11 | Protein Coding | 51 | GC11M017627 | 0.228804097 |
| MAOA | Monoamine Oxidase A | Protein Coding | 51 | GC0XP043654 | 0.228804097 |
| STX1A | Syntaxin 1A | Protein Coding | 51 | GC07M073700 | 0.228804097 |
| ACLY | ATP Citrate Lyase | Protein Coding | 50 | GC17M041866 | 0.228804097 |
| ATP7B | ATPase Copper Transporting Beta | Protein Coding | 50 | GC13M051930 | 0.228804097 |
| GCGR | Glucagon Receptor | Protein Coding | 50 | GC17P081804 | 0.228804097 |
| GLRB | Glycine Receptor Beta | Protein Coding | 50 | GC04P157076 | 0.228804097 |
| GOT2 | Glutamic-Oxaloacetic Transaminase 2 | Protein Coding | 50 | GC16M058707 | 0.228804097 |
| PPM1D | Protein Phosphatase, Mg2+/Mn2+ Dependent 1D | Protein Coding | 50 | GC17P060600 | 0.228804097 |
| PTPN2 | Protein Tyrosine Phosphatase Non-Receptor Type 2 | Protein Coding | 50 | GC18M025513 | 0.228804097 |
| PTPRO | Protein Tyrosine Phosphatase Receptor Type O | Protein Coding | 50 | GC12P022665 | 0.228804097 |
| SPTLC2 | Serine Palmitoyltransferase Long Chain Base Subunit 2 | Protein Coding | 50 | GC14M077505 | 0.228804097 |
| TNNT2 | Troponin T2, Cardiac Type | Protein Coding | 50 | GC01M201359 | 0.228804097 |
| ABCC8 | ATP Binding Cassette Subfamily C Member 8 | Protein Coding | 49 | GC11M017392 | 0.228804097 |
| HTR1D | 5-Hydroxytryptamine Receptor 1D | Protein Coding | 49 | GC01M023191 | 0.228804097 |
| ITPR2 | Inositol 1,4,5-Trisphosphate Receptor Type 2 | Protein Coding | 49 | GC12M026336 | 0.228804097 |
| SMC3 | Structural Maintenance Of Chromosomes 3 | Protein Coding | 49 | GC10P110567 | 0.228804097 |
| WFS1 | Wolframin ER Transmembrane Glycoprotein | Protein Coding | 49 | GC04P006260 | 0.228804097 |
| ANTXR2 | ANTXR Cell Adhesion Molecule 2 | Protein Coding | 48 | GC04M079901 | 0.228804097 |
| ATP6AP2 | ATPase H+ Transporting Accessory Protein 2 | Protein Coding | 48 | GC0XP040582 | 0.228804097 |
| CLCN7 | Chloride Voltage-Gated Channel 7 | Protein Coding | 48 | GC16M001444 | 0.228804097 |
| EEF1A2 | Eukaryotic Translation Elongation Factor 1 Alpha 2 | Protein Coding | 48 | GC20M063488 | 0.228804097 |
| FZD7 | Frizzled Class Receptor 7 | Protein Coding | 48 | GC02P202034 | 0.228804097 |
| GABRA4 | Gamma-Aminobutyric Acid Type A Receptor Subunit Alpha4 | Protein Coding | 48 | GC04M046836 | 0.228804097 |
| HMOX2 | Heme Oxygenase 2 | Protein Coding | 48 | GC16P004474 | 0.228804097 |
| PDX1 | Pancreatic And Duodenal Homeobox 1 | Protein Coding | 48 | GC13P027921 | 0.228804097 |
| PHYH | Phytanoyl-CoA 2-Hydroxylase | Protein Coding | 48 | GC10M013277 | 0.228804097 |
| PTGDS | Prostaglandin D2 Synthase | Protein Coding | 48 | GC09P137112 | 0.228804097 |
| RALA | RAS Like Proto-Oncogene A | Protein Coding | 48 | GC07P039622 | 0.228804097 |
| SLC2A9 | Solute Carrier Family 2 Member 9 | Protein Coding | 48 | GC04M009772 | 0.228804097 |
| STEAP3 | STEAP3 Metalloreductase | Protein Coding | 48 | GC02P123196 | 0.228804097 |
| TBL1XR1 | TBL1X/Y Related 1 | Protein Coding | 48 | GC03M177019 | 0.228804097 |
| UBE2C | Ubiquitin Conjugating Enzyme E2 C | Protein Coding | 48 | GC20P045812 | 0.228804097 |
| WASF1 | WASP Family Member 1 | Protein Coding | 48 | GC06M110099 | 0.228804097 |
| APOA5 | Apolipoprotein A5 | Protein Coding | 47 | GC11M116789 | 0.228804097 |
| ATP6V0A2 | ATPase H+ Transporting V0 Subunit A2 | Protein Coding | 47 | GC12P123712 | 0.228804097 |
| DDB1 | Damage Specific DNA Binding Protein 1 | Protein Coding | 47 | GC11M096879 | 0.228804097 |
| FA2H | Fatty Acid 2-Hydroxylase | Protein Coding | 47 | GC16M074712 | 0.228804097 |
| GLO1 | Glyoxalase I | Protein Coding | 47 | GC06M069363 | 0.228804097 |
| GPC6 | Glypican 6 | Protein Coding | 47 | GC13P093226 | 0.228804097 |
| PANX1 | Pannexin 1 | Protein Coding | 47 | GC11P094128 | 0.228804097 |
| PAX7 | Paired Box 7 | Protein Coding | 47 | GC01P018631 | 0.228804097 |
| PGAM1 | Phosphoglycerate Mutase 1 | Protein Coding | 47 | GC10P097426 | 0.228804097 |
| PLK2 | Polo Like Kinase 2 | Protein Coding | 47 | GC05M058453 | 0.228804097 |
| PPP4C | Protein Phosphatase 4 Catalytic Subunit | Protein Coding | 47 | GC16P041865 | 0.228804097 |
| PRICKLE1 | Prickle Planar Cell Polarity Protein 1 | Protein Coding | 47 | GC12M042456 | 0.228804097 |
| PSMB4 | Proteasome 20S Subunit Beta 4 | Protein Coding | 47 | GC01P151372 | 0.228804097 |
| SDHD | Succinate Dehydrogenase Complex Subunit D | Protein Coding | 47 | GC11P112087 | 0.228804097 |
| SORT1 | Sortilin 1 | Protein Coding | 47 | GC01M109310 | 0.228804097 |
| SPG7 | SPG7 Matrix AAA Peptidase Subunit, Paraplegin | Protein Coding | 47 | GC16P091441 | 0.228804097 |
| TFG | Trafficking From ER To Golgi Regulator | Protein Coding | 47 | GC03P100709 | 0.228804097 |
| DROSHA | Drosha Ribonuclease III | Protein Coding | 46 | GC05M031401 | 0.228804097 |
| DTYMK | Deoxythymidylate Kinase | Protein Coding | 46 | GC02M241675 | 0.228804097 |
| LRP4 | LDL Receptor Related Protein 4 | Protein Coding | 46 | GC11M096698 | 0.228804097 |
| PDIA3 | Protein Disulfide Isomerase Family A Member 3 | Protein Coding | 46 | GC15P043746 | 0.228804097 |
| RICTOR | RPTOR Independent Companion Of MTOR Complex 2 | Protein Coding | 46 | GC05M038937 | 0.228804097 |
| RPL15 | Ribosomal Protein L15 | Protein Coding | 46 | GC03P023916 | 0.228804097 |
| SLC2A5 | Solute Carrier Family 2 Member 5 | Protein Coding | 46 | GC01M009036 | 0.228804097 |
| SLC33A1 | Solute Carrier Family 33 Member 1 | Protein Coding | 46 | GC03M155821 | 0.228804097 |
| ANXA6 | Annexin A6 | Protein Coding | 45 | GC05M151100 | 0.228804097 |
| B3GAT3 | Beta-1,3-Glucuronyltransferase 3 | Protein Coding | 45 | GC11M096926 | 0.228804097 |
| CDKL5 | Cyclin Dependent Kinase Like 5 | Protein Coding | 45 | GC0XP018425 | 0.228804097 |
| CHST6 | Carbohydrate Sulfotransferase 6 | Protein Coding | 45 | GC16M075472 | 0.228804097 |
| CYB5A | Cytochrome B5 Type A | Protein Coding | 45 | GC18M074250 | 0.228804097 |
| DIAPH3 | Diaphanous Related Formin 3 | Protein Coding | 45 | GC13M059665 | 0.228804097 |
| IFNA2 | Interferon Alpha 2 | Protein Coding | 45 | GC09M021384 | 0.228804097 |
| KDM4A | Lysine Demethylase 4A | Protein Coding | 45 | GC01P043650 | 0.228804097 |
| LCP1 | Lymphocyte Cytosolic Protein 1 | Protein Coding | 45 | GC13M046132 | 0.228804097 |
| LIPG | Lipase G, Endothelial Type | Protein Coding | 45 | GC18P049560 | 0.228804097 |
| MLC1 | Modulator Of VRAC Current 1 | Protein Coding | 45 | GC22M050059 | 0.228804097 |
| MYOC | Myocilin | Protein Coding | 45 | GC01M171604 | 0.228804097 |
| POLR1D | RNA Polymerase I And III Subunit D | Protein Coding | 45 | GC13P027620 | 0.228804097 |
| POU2F1 | POU Class 2 Homeobox 1 | Protein Coding | 45 | GC01P167190 | 0.228804097 |
| RIPK4 | Receptor Interacting Serine/Threonine Kinase 4 | Protein Coding | 45 | GC21M041739 | 0.228804097 |
| ST3GAL1 | ST3 Beta-Galactoside Alpha-2,3-Sialyltransferase 1 | Protein Coding | 45 | GC08M133454 | 0.228804097 |
| TGM3 | Transglutaminase 3 | Protein Coding | 45 | GC20P002296 | 0.228804097 |
| TRAF4 | TNF Receptor Associated Factor 4 | Protein Coding | 45 | GC17P057797 | 0.228804097 |
| TUSC3 | Tumor Suppressor Candidate 3 | Protein Coding | 45 | GC08P015417 | 0.228804097 |
| UCP1 | Uncoupling Protein 1 | Protein Coding | 45 | GC04M140559 | 0.228804097 |
| UCP3 | Uncoupling Protein 3 | Protein Coding | 45 | GC11M074000 | 0.228804097 |
| ARHGAP4 | Rho GTPase Activating Protein 4 | Protein Coding | 44 | GC0XM153907 | 0.228804097 |
| ATP6V1H | ATPase H+ Transporting V1 Subunit H | Protein Coding | 44 | GC08M053715 | 0.228804097 |
| FMO5 | Flavin Containing Dimethylaniline Monoxygenase 5 | Protein Coding | 44 | GC01M147175 | 0.228804097 |
| FOXE1 | Forkhead Box E1 | Protein Coding | 44 | GC09P097853 | 0.228804097 |
| GABRP | Gamma-Aminobutyric Acid Type A Receptor Subunit Pi | Protein Coding | 44 | GC05P170763 | 0.228804097 |
| GCLM | Glutamate-Cysteine Ligase Modifier Subunit | Protein Coding | 44 | GC01M093885 | 0.228804097 |
| GJB4 | Gap Junction Protein Beta 4 | Protein Coding | 44 | GC01P034759 | 0.228804097 |
| HACE1 | HECT Domain And Ankyrin Repeat Containing E3 Ubiquitin Protein Ligase 1 | Protein Coding | 44 | GC06M104728 | 0.228804097 |
| IFITM3 | Interferon Induced Transmembrane Protein 3 | Protein Coding | 44 | GC11M000319 | 0.228804097 |
| LEMD3 | LEM Domain Containing 3 | Protein Coding | 44 | GC12P065169 | 0.228804097 |
| MPC1 | Mitochondrial Pyruvate Carrier 1 | Protein Coding | 44 | GC06M166364 | 0.228804097 |
| NAB2 | NGFI-A Binding Protein 2 | Protein Coding | 44 | GC12P057437 | 0.228804097 |
| NCK2 | NCK Adaptor Protein 2 | Protein Coding | 44 | GC02P105744 | 0.228804097 |
| NDUFAF1 | NADH:Ubiquinone Oxidoreductase Complex Assembly Factor 1 | Protein Coding | 44 | GC15M041387 | 0.228804097 |
| NME4 | NME/NM23 Nucleoside Diphosphate Kinase 4 | Protein Coding | 44 | GC16P000396 | 0.228804097 |
| PDIA4 | Protein Disulfide Isomerase Family A Member 4 | Protein Coding | 44 | GC07M149003 | 0.228804097 |
| PPP1R3A | Protein Phosphatase 1 Regulatory Subunit 3A | Protein Coding | 44 | GC07M113876 | 0.228804097 |
| PROZ | Protein Z, Vitamin K Dependent Plasma Glycoprotein | Protein Coding | 44 | GC13P113158 | 0.228804097 |
| SLC26A8 | Solute Carrier Family 26 Member 8 | Protein Coding | 44 | GC06M069351 | 0.228804097 |
| SMAD5 | SMAD Family Member 5 | Protein Coding | 44 | GC05P136132 | 0.228804097 |
| TNFRSF10D | TNF Receptor Superfamily Member 10d | Protein Coding | 44 | GC08M023135 | 0.228804097 |
| VPS13A | Vacuolar Protein Sorting 13 Homolog A | Protein Coding | 44 | GC09P077177 | 0.228804097 |
| AATF | Apoptosis Antagonizing Transcription Factor | Protein Coding | 43 | GC17P036948 | 0.228804097 |
| ATP13A1 | ATPase 13A1 | Protein Coding | 43 | GC19M019645 | 0.228804097 |
| CAPN10 | Calpain 10 | Protein Coding | 43 | GC02P240586 | 0.228804097 |
| COX7A2L | Cytochrome C Oxidase Subunit 7A2 Like | Protein Coding | 43 | GC02M042312 | 0.228804097 |
| GLDN | Gliomedin | Protein Coding | 43 | GC15P051341 | 0.228804097 |
| MAGED1 | MAGE Family Member D1 | Protein Coding | 43 | GC0XP051803 | 0.228804097 |
| NCAPG | Non-SMC Condensin I Complex Subunit G | Protein Coding | 43 | GC04P018750 | 0.228804097 |
| NDUFC2 | NADH:Ubiquinone Oxidoreductase Subunit C2 | Protein Coding | 43 | GC11M078068 | 0.228804097 |
| OLFM4 | Olfactomedin 4 | Protein Coding | 43 | GC13P053028 | 0.228804097 |
| RAB6A | RAB6A, Member RAS Oncogene Family | Protein Coding | 43 | GC11M097360 | 0.228804097 |
| RGS6 | Regulator Of G Protein Signaling 6 | Protein Coding | 43 | GC14P071867 | 0.228804097 |
| SEMA4B | Semaphorin 4B | Protein Coding | 43 | GC15P090160 | 0.228804097 |
| SLC35A2 | Solute Carrier Family 35 Member A2 | Protein Coding | 43 | GC0XM048903 | 0.228804097 |
| SYVN1 | Synoviolin 1 | Protein Coding | 43 | GC11M097035 | 0.228804097 |
| TBL2 | Transducin Beta Like 2 | Protein Coding | 43 | GC07M073619 | 0.228804097 |
| TRA2B | Transformer 2 Beta Homolog | Protein Coding | 43 | GC03M185914 | 0.228804097 |
| UPF1 | UPF1 RNA Helicase And ATPase | Protein Coding | 43 | GC19P018831 | 0.228804097 |
| WDFY3 | WD Repeat And FYVE Domain Containing 3 | Protein Coding | 43 | GC04M084669 | 0.228804097 |
| AJUBA | Ajuba LIM Protein | Protein Coding | 42 | GC14M022971 | 0.228804097 |
| AMIGO2 | Adhesion Molecule With Ig Like Domain 2 | Protein Coding | 42 | GC12M047077 | 0.228804097 |
| CDKAL1 | CDK5 Regulatory Subunit Associated Protein 1 Like 1 | Protein Coding | 42 | GC06P020534 | 0.228804097 |
| EGFL7 | EGF Like Domain Multiple 7 | Protein Coding | 42 | GC09P136658 | 0.228804097 |
| GRSF1 | G-Rich RNA Sequence Binding Factor 1 | Protein Coding | 42 | GC04M070815 | 0.228804097 |
| HLX | H2.0 Like Homeobox | Protein Coding | 42 | GC01P220879 | 0.228804097 |
| LGALS2 | Galectin 2 | Protein Coding | 42 | GC22M037570 | 0.228804097 |
| LMO7 | LIM Domain 7 | Protein Coding | 42 | GC13P075620 | 0.228804097 |
| RPL28 | Ribosomal Protein L28 | Protein Coding | 42 | GC19P070489 | 0.228804097 |
| SCAMP2 | Secretory Carrier Membrane Protein 2 | Protein Coding | 42 | GC15M074843 | 0.228804097 |
| SLC38A2 | Solute Carrier Family 38 Member 2 | Protein Coding | 42 | GC12M046358 | 0.228804097 |
| SPN | Sialophorin | Protein Coding | 42 | GC16P029662 | 0.228804097 |
| CLCC1 | Chloride Channel CLIC Like 1 | Protein Coding | 41 | GC01M108881 | 0.228804097 |
| DLGAP4 | DLG Associated Protein 4 | Protein Coding | 41 | GC20P036308 | 0.228804097 |
| GFUS | GDP-L-Fucose Synthase | Protein Coding | 41 | GC08M143673 | 0.228804097 |
| GKN1 | Gastrokine 1 | Protein Coding | 41 | GC02P068974 | 0.228804097 |
| GPR61 | G Protein-Coupled Receptor 61 | Protein Coding | 41 | GC01P109539 | 0.228804097 |
| NACA | Nascent Polypeptide Associated Complex Subunit Alpha | Protein Coding | 41 | GC12M056712 | 0.228804097 |
| RASSF4 | Ras Association Domain Family Member 4 | Protein Coding | 41 | GC10P044959 | 0.228804097 |
| RPS18 | Ribosomal Protein S18 | Protein Coding | 41 | GC06P087803 | 0.228804097 |
| SHARPIN | SHANK Associated RH Domain Interactor | Protein Coding | 41 | GC08M144098 | 0.228804097 |
| SMPD4 | Sphingomyelin Phosphodiesterase 4 | Protein Coding | 41 | GC02M130151 | 0.228804097 |
| SRP68 | Signal Recognition Particle 68 | Protein Coding | 41 | GC17M076038 | 0.228804097 |
| STX7 | Syntaxin 7 | Protein Coding | 41 | GC06M132445 | 0.228804097 |
| TMX1 | Thioredoxin Related Transmembrane Protein 1 | Protein Coding | 41 | GC14P051240 | 0.228804097 |
| ACSM3 | Acyl-CoA Synthetase Medium Chain Family Member 3 | Protein Coding | 40 | GC16P020610 | 0.228804097 |
| ADGRA2 | Adhesion G Protein-Coupled Receptor A2 | Protein Coding | 40 | GC08P037785 | 0.228804097 |
| CLIC6 | Chloride Intracellular Channel 6 | Protein Coding | 40 | GC21P034671 | 0.228804097 |
| GDF7 | Growth Differentiation Factor 7 | Protein Coding | 40 | GC02P020666 | 0.228804097 |
| GPAT4 | Glycerol-3-Phosphate Acyltransferase 4 | Protein Coding | 40 | GC08P041577 | 0.228804097 |
| KIAA0319L | KIAA0319 Like | Protein Coding | 40 | GC01M035393 | 0.228804097 |
| LAPTM4B | Lysosomal Protein Transmembrane 4 Beta | Protein Coding | 40 | GC08P097775 | 0.228804097 |
| MT1E | Metallothionein 1E | Protein Coding | 40 | GC16P056625 | 0.228804097 |
| QSOX2 | Quiescin Sulfhydryl Oxidase 2 | Protein Coding | 40 | GC09M136206 | 0.228804097 |
| RHOBTB3 | Rho Related BTB Domain Containing 3 | Protein Coding | 40 | GC05P095713 | 0.228804097 |
| TAPT1 | Transmembrane Anterior Posterior Transformation 1 | Protein Coding | 40 | GC04M016162 | 0.228804097 |
| TMEM98 | Transmembrane Protein 98 | Protein Coding | 40 | GC17P032927 | 0.228804097 |
| TRIM31 | Tripartite Motif Containing 31 | Protein Coding | 40 | GC06M069109 | 0.228804097 |
| ZFX | Zinc Finger Protein X-Linked | Protein Coding | 40 | GC0XP024148 | 0.228804097 |
| ZMYND10 | Zinc Finger MYND-Type Containing 10 | Protein Coding | 40 | GC03M051481 | 0.228804097 |
| AKAP10 | A-Kinase Anchoring Protein 10 | Protein Coding | 39 | GC17M019904 | 0.228804097 |
| ATP5PB | ATP Synthase Peripheral Stalk-Membrane Subunit B | Protein Coding | 39 | GC01P111449 | 0.228804097 |
| CCAR2 | Cell Cycle And Apoptosis Regulator 2 | Protein Coding | 39 | GC08P022604 | 0.228804097 |
| MARCKSL1 | MARCKS Like 1 | Protein Coding | 39 | GC01M032334 | 0.228804097 |
| TREML1 | Triggering Receptor Expressed On Myeloid Cells Like 1 | Protein Coding | 39 | GC06M069399 | 0.228804097 |
| VMP1 | Vacuole Membrane Protein 1 | Protein Coding | 39 | GC17P059707 | 0.228804097 |
| WDR72 | WD Repeat Domain 72 | Protein Coding | 39 | GC15M121946 | 0.228804097 |
| BOP1 | BOP1 Ribosomal Biogenesis Factor | Protein Coding | 38 | GC08M144262 | 0.228804097 |
| CEMIP2 | Cell Migration Inducing Hyaluronidase 2 | Protein Coding | 38 | GC09M071684 | 0.228804097 |
| CNN3 | Calponin 3 | Protein Coding | 38 | GC01M094896 | 0.228804097 |
| DHRS7 | Dehydrogenase/Reductase 7 | Protein Coding | 38 | GC14M060144 | 0.228804097 |
| FAM172A | Family With Sequence Similarity 172 Member A | Protein Coding | 38 | GC05M093617 | 0.228804097 |
| LACTB | Lactamase Beta | Protein Coding | 38 | GC15P121579 | 0.228804097 |
| LIX1 | Limb And CNS Expressed 1 | Protein Coding | 38 | GC05M097091 | 0.228804097 |
| PHLDB2 | Pleckstrin Homology Like Domain Family B Member 2 | Protein Coding | 38 | GC03P111732 | 0.228804097 |
| RFTN1 | Raftlin, Lipid Raft Linker 1 | Protein Coding | 38 | GC03M021384 | 0.228804097 |
| SEC62 | SEC62 Homolog, Preprotein Translocation Factor | Protein Coding | 38 | GC03P169966 | 0.228804097 |
| SRPRA | SRP Receptor Subunit Alpha | Protein Coding | 38 | GC11M126255 | 0.228804097 |
| LRRC3B | Leucine Rich Repeat Containing 3B | Protein Coding | 37 | GC03P026640 | 0.228804097 |
| MACO1 | Macoilin 1 | Protein Coding | 37 | GC01P025430 | 0.228804097 |
| NDC1 | NDC1 Transmembrane Nucleoporin | Protein Coding | 37 | GC01M053765 | 0.228804097 |
| OCIAD2 | OCIA Domain Containing 2 | Protein Coding | 37 | GC04M048887 | 0.228804097 |
| RABL3 | RAB, Member Of RAS Oncogene Family Like 3 | Protein Coding | 37 | GC03M120686 | 0.228804097 |
| SPINK7 | Serine Peptidase Inhibitor Kazal Type 7 | Protein Coding | 37 | GC05P148312 | 0.228804097 |
| SRCIN1 | SRC Kinase Signaling Inhibitor 1 | Protein Coding | 37 | GC17M038530 | 0.228804097 |
| TMEM199 | Transmembrane Protein 199 | Protein Coding | 37 | GC17P057770 | 0.228804097 |
| TMEM214 | Transmembrane Protein 214 | Protein Coding | 37 | GC02P027032 | 0.228804097 |
| TOM1L1 | Target Of Myb1 Like 1 Membrane Trafficking Protein | Protein Coding | 37 | GC17P059117 | 0.228804097 |
| TRIM16 | Tripartite Motif Containing 16 | Protein Coding | 37 | GC17M015627 | 0.228804097 |
| ZNRF3 | Zinc And Ring Finger 3 | Protein Coding | 37 | GC22P028883 | 0.228804097 |
| C2CD2 | C2 Calcium Dependent Domain Containing 2 | Protein Coding | 36 | GC21M041885 | 0.228804097 |
| DIRAS3 | DIRAS Family GTPase 3 | Protein Coding | 36 | GC01M068045 | 0.228804097 |
| EMC2 | ER Membrane Protein Complex Subunit 2 | Protein Coding | 36 | GC08P108443 | 0.228804097 |
| IGSF9B | Immunoglobulin Superfamily Member 9B | Protein Coding | 36 | GC11M133990 | 0.228804097 |
| MT1B | Metallothionein 1B | Protein Coding | 36 | GC16P057084 | 0.228804097 |
| STX10 | Syntaxin 10 | Protein Coding | 36 | GC19M013144 | 0.228804097 |
| TTC27 | Tetratricopeptide Repeat Domain 27 | Protein Coding | 36 | GC02P032628 | 0.228804097 |
| VEZT | Vezatin, Adherens Junctions Transmembrane Protein | Protein Coding | 36 | GC12P095217 | 0.228804097 |
| FAM83F | Family With Sequence Similarity 83 Member F | Protein Coding | 35 | GC22P039994 | 0.228804097 |
| MTFP1 | Mitochondrial Fission Process 1 | Protein Coding | 35 | GC22P040535 | 0.228804097 |
| MXRA5 | Matrix Remodeling Associated 5 | Protein Coding | 35 | GC0XM003308 | 0.228804097 |
| NAIF1 | Nuclear Apoptosis Inducing Factor 1 | Protein Coding | 35 | GC09M128061 | 0.228804097 |
| TAC4 | Tachykinin Precursor 4 | Protein Coding | 35 | GC17M049838 | 0.228804097 |
| BEX2 | Brain Expressed X-Linked 2 | Protein Coding | 34 | GC0XM103309 | 0.228804097 |
| CAAP1 | Caspase Activity And Apoptosis Inhibitor 1 | Protein Coding | 34 | GC09M026840 | 0.228804097 |
| ELAPOR2 | Endosome-Lysosome Associated Apoptosis And Autophagy Regulator Family Member 2 | Protein Coding | 34 | GC07M086877 | 0.228804097 |
| FIBIN | Fin Bud Initiation Factor Homolog | Protein Coding | 34 | GC11P026994 | 0.228804097 |
| TMEM92 | Transmembrane Protein 92 | Protein Coding | 34 | GC17P059022 | 0.228804097 |
| RELL1 | RELT Like 1 | Protein Coding | 33 | GC04M037592 | 0.228804097 |
| GINM1 | Glycosylated Integral Membrane Protein 1 | Protein Coding | 32 | GC06P149566 | 0.228804097 |
| NPS | Neuropeptide S | Protein Coding | 32 | GC10P127549 | 0.228804097 |
| PBDC1 | Polysaccharide Biosynthesis Domain Containing 1 | Protein Coding | 32 | GC0XP076172 | 0.228804097 |
| RETREG2 | Reticulophagy Regulator Family Member 2 | Protein Coding | 32 | GC02P219177 | 0.228804097 |
| RUSF1 | RUS Family Member 1 | Protein Coding | 31 | GC16M037899 | 0.228804097 |
| SPRR2B | Small Proline Rich Protein 2B | Protein Coding | 30 | GC01M153070 | 0.228804097 |
| HOTTIP | HOXA Distal Transcript Antisense RNA | RNA Gene | 24 | GC07P027198 | 0.228804097 |
| CRNDE | Colorectal Neoplasia Differentially Expressed | RNA Gene | 23 | GC16M054845 | 0.228804097 |
| MIR615 | MicroRNA 615 | RNA Gene | 22 | GC12P054033 | 0.228804097 |
| ZEB2-AS1 | ZEB2 Antisense RNA 1 | RNA Gene | 22 | GC02P144519 | 0.228804097 |
| SNHG6 | Small Nucleolar RNA Host Gene 6 | RNA Gene | 21 | GC08M066921 | 0.228804097 |
| MIR124-2 | MicroRNA 124-2 | RNA Gene | 20 | GC08P064379 | 0.228804097 |
| LINC00958 | Long Intergenic Non-Protein Coding RNA 958 | RNA Gene | 19 | GC11M012877 | 0.228804097 |
| LINC01561 | Long Intergenic Non-Protein Coding RNA 1561 | RNA Gene | 19 | GC10P120597 | 0.228804097 |
| MIR520C | MicroRNA 520c | RNA Gene | 19 | GC19P070430 | 0.228804097 |
| MIR654 | MicroRNA 654 | RNA Gene | 19 | GC14P110461 | 0.228804097 |
| MIR802 | MicroRNA 802 | RNA Gene | 19 | GC21P035720 | 0.228804097 |
| LINC-ROR | Long Intergenic Non-Protein Coding RNA, Regulator Of Reprogramming | RNA Gene | 18 | GC18M057054 | 0.228804097 |
| LINC00857 | Long Intergenic Non-Protein Coding RNA 857 | RNA Gene | 18 | GC10P093451 | 0.228804097 |
| MIR3174 | MicroRNA 3174 | RNA Gene | 17 | GC15P090006 | 0.228804097 |
| LINC00968 | Long Intergenic Non-Protein Coding RNA 968 | RNA Gene | 16 | GC08M056496 | 0.228804097 |
| MIR1248 | MicroRNA 1248 | RNA Gene | 16 | GC03P187151 | 0.228804097 |
| MIR1258 | MicroRNA 1258 | RNA Gene | 15 | GC02M179860 | 0.228804097 |
| NKILA | NF-KappaB Interacting LncRNA | RNA Gene | 15 | GC20P057711 | 0.228804097 |
| COL6A4P1 | Collagen Type VI Alpha 4 Pseudogene 1 | Pseudogene | 14 | GC03M015151 | 0.228804097 |
| LINC00997 | Long Intergenic Non-Protein Coding RNA 997 | RNA Gene | 13 | GC07P033265 | 0.228804097 |
| PCBP2-OT1 | PCBP2 Overlapping Transcript 1 | RNA Gene | 12 | GC12P053464 | 0.228804097 |
| VIM2P | Vimentin 2, Pseudogene | Pseudogene | 5 | GC06M126602 | 0.228804097 |
| LEPQTL1 | Leptin, Serum Levels Of | Genetic Locus | 4 | GC02U903086 | 0.228804097 |
| FCGR3B | Fc Gamma Receptor IIIb | Protein Coding | 45 | GC01M161623 | 0.22631295 |
| IRF7 | Interferon Regulatory Factor 7 | Protein Coding | 50 | GC11M000612 | 0.209525079 |
| CHAD | Chondroadherin | Protein Coding | 40 | GC17M050464 | 0.209525079 |
| TCIM | Transcriptional And Immune Response Regulator | Protein Coding | 33 | GC08P040153 | 0.209525079 |
