# Supplementary material for: The role of intestinal immune cells and matrix metalloproteinases in inflammatory bowel disease
Source: Front Immunol. 2023 Jan 17;13:1067950. doi: 10.3389/fimmu.2022.1067950 (PMC9888429; doi:10.3389/fimmu.2022.1067950)
Supplement: Supplementary Table 2 — Scoring criteria for the histological examination of mice. [file Table_2.docx]

Supplementary Material

Table 2 Score criteria for histological examination of mice

| **Mice histopathology record score sheet** | | | | | |
| --- | --- | --- | --- | --- | --- |
| group | number | Histopathological changes of the colon | | |  |
|  |  | ulcer | Epithelial cell changes | Inflammatory infiltration | Lymph node |
| DSS | 1 | 0 | 4 | 4 | 0 |
| DSS | 2 | 0 | 3 | 4 | 2 |
| DSS | 3 | 0 | 3 | 2 | 0 |
| DSS | 4 | 0 | 3 | 4 | 0 |
| DSS | 5 | 0 | 3 | 4 | 1 |
| DSS | 6 | 0 | 3 | 4 | 0 |
| Control | 1 | 0 | 0 | 0 | 0 |
| Control | 2 | 0 | 0 | 0 | 0 |
| Control | 2 | 0 | 0 | 0 | 0 |
| Control | 4 | 0 | 0 | 0 | 0 |
| Control | 5 | 0 | 0 | 0 | 0 |
| Control | 6 | 0 | 0 | 0 | 0 |
